# Intermolecular Photocatalytic Chemo‐, Stereo‐ and Regioselective Thiol–Yne–Ene Coupling Reaction

**DOI:** 10.1002/anie.202116888

**Published:** 2022-03-02

**Authors:** Julia V. Burykina, Andrey D. Kobelev, Nikita S. Shlapakov, Alexander Yu. Kostyukovich, Artem N. Fakhrutdinov, Burkhard König, Valentine P. Ananikov

**Affiliations:** ^1^ Zelinsky Institute of Organic Chemistry Russian Academy of Sciences Leninsky Prospect, 47 Moscow 119991 Russia; ^2^ Lomonosov Moscow State University Leninskie Gory GSP-1, 1-3 Moscow 119991 Russia; ^3^ Institut für Organische Chemie Universität Regensburg Universitätstrasse 31 93053 Regensburg Germany

**Keywords:** Multicomponent Reactions, Photocatalysis, Reaction Mechanisms, Thiol–Yne–Ene, Visible Light

## Abstract

The first example of an intermolecular thiol–yne–ene coupling reaction is reported for the one‐pot construction of C−S and C−C bonds. Thiol–yne–ene coupling opens a new dimension in building molecular complexity to access densely functionalized products. The employment of Eosin Y/DBU/MeOH photocatalytic system suppresses hydrogen atom transfer (HAT) and associative reductant upconversion (via C−S three‐electron σ‐bond formation). Investigation of the reaction mechanism by combining online ESI‐UHRMS, EPR spectroscopy, isotope labeling, determination of quantum yield, cyclic voltammetry, Stern–Volmer measurements and computational modeling revealed a unique photoredox cycle with four radical‐involving stages. As a result, previously unavailable products of the thiol–yne–ene reaction were obtained in good yields with high selectivity. They can serve as stable precursors for synthesizing synthetically demanding activated 1,3‐dienes.

## Graphical abstract

An atom‐economical metal‐free photoredox three‐component coupling of thiols with readily available alkynes and α‐vinylsulfide has been developed. The reaction uses the easy‐to‐handle EosinY/DBU photocatalytic system, resulting in a one‐pot synthesis of C−S and C−C bonds. The reaction mechanism was investigated by combining mass spectrometry, EPR spectroscopy, isotope labeling, cyclic voltammetry, Stern–Volmer measurements and computational modeling, revealing a unique photoredox cycle.

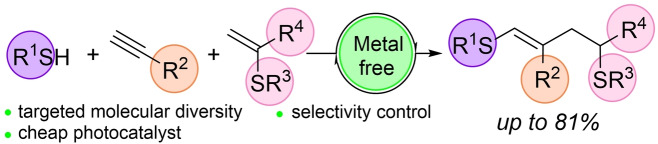

## Introduction

Alkyne and alkene moieties are ubiquitous building blocks for photochemical transformations, given their widespread natural and synthetic occurrence.[^1^, ^2^, ^3^, ^4^, ^5^, ^6^, ^7^] Therefore, reactions aimed at combining them by creating a new C−C bond are an integral part of the path towards molecular complexity. Along with the generally accepted C−C cross‐coupling reactions involving unsaturated hydrocarbons with leaving groups (Sonogashira, Suzuki, Heck and other reactions), alternative methods of direct atom‐economical ene–ene, ene–yne and yne–yne couplings were developed.[^8^, ^9^, ^10^, ^11^, ^12^] These methods provide a great variety of possible products; however, at the same time, the control of chemo‐, regio‐ and stereoselectivity in these reactions becomes challenging, especially in the case of intermolecular processes.[^13^, ^14^, ^15^, ^16^, ^17^, ^18^, ^19^, ^20^] In recent years, alkynes have also undergone a renaissance in the chemistry of radical photochemical processes involving metal complexes, since the triple bond is a convenient platform for selective bifunctionalization through C−C bond creation.[^21^, ^22^, ^23^, ^24^]

**Scheme 1:**
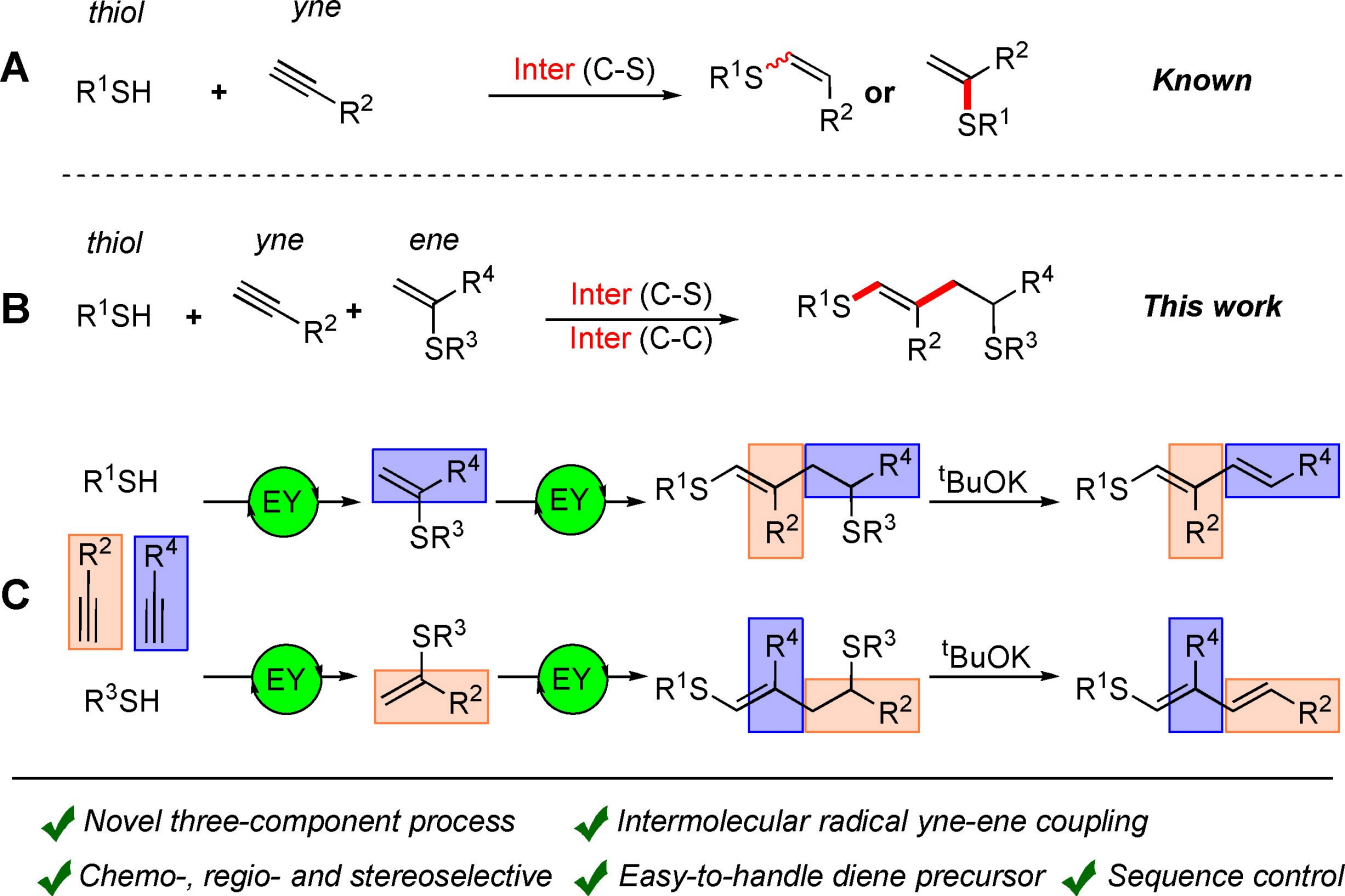
A) Photocatalyzed thiol–yne click reaction. B) Three‐component system for one‐pot intermolecular C−C and C−S bonds construction. C) Molecular diversity in the thiol–yne–ene reaction. EY‐Eosin Y.

The thiol–yne reaction is one of the most well‐known processes in radical chemistry (Scheme 1A). This addition process is called a click reaction and has several advantages in terms of straightforward and predictable transformations, atom economy and high yields.[^25^, ^26^] Thiol–yne click reactions are utilized in the modifications of peptides and proteins,[^27^, ^28^, ^29^] material synthesis,[^30^, ^31^, ^32^] polymerization,[^33^, ^34^, ^35^] and surface modification.[^36^, ^37^, ^38^] Currently, thiol–yne coupling is a core methodology of modern chemistry. Several intramolecular cascade reactions were developed based on thiol–yne coupling for radical late‐stage cyclization in total syntheses.^[39]^ In intramolecular transformations, the reaction selectivity and geometry of the product can be predefined by specifically installing σ‐bonds between the components.

The energetic parameters of the reaction are improved due to the elimination of the disfavoring entropy contribution (i.e., one reacting molecule instead of two). Intermolecular transformations are much more difficult to perform, although they are highly useful. A large variety of easily available and diverse small molecules can result in a cost‐efficient synthetic methodology. Each new component (i.e., changing from two‐component to three‐component intermolecular coupling) will open new opportunities in building molecular complexity and impose several difficulties for reaction design. Indeed, intermolecular reactions have to overcome the disfavoring entropy contribution, which is a serious limitation in the case of three components. Moreover, achieving chemo‐, stereo‐ and regioselectivities become a critical issue due to the absence of predefined bonds between the components or directing groups in starting compounds. Therefore, the design of radical‐based three‐component processes is highly challenging, particularly in the case of modern metal‐free catalytic systems.[^40^, ^41^, ^42^, ^43^]

In the present article, for the first time, we describe the methodology for photoredox three‐component thiol–yne–ene intermolecular coupling (Scheme 1B). A wide range of aromatic alkynes and thiols bearing various functional groups, including practically demanded fluorinated moieties,[^44^, ^45^, ^46^, ^47^, ^48^] are compatible with the reaction conditions and provide the target products in good yields.

α‐Vinylsulfides are particularly attractive alkene coupling partners produced under similar photocatalytic conditions.^[49]^ Three‐stage synthesis, including photocatalytic transformation for SR^3^ group elimination with the formation of corresponding dienes, can be considered a formal thiol–yne–yne coupling with controlled sequence order (Scheme 1C). To the best of our knowledge, such unprecedented selectivity with carbon chain sequence management is unique for radical reactions. Such an approach provides an opportunity for solving a challenging practical problem of obtaining and storing activated sulfur‐containing dienes.[^50^, ^51^]

Real‐time observation of the trapped radicals in the thiol–yne–ene reaction was performed by advanced ultra‐high resolution mass spectrometry (ESI‐UHRMS). Electron paramagnetic resonance (EPR), 1D and 2D NMR spectroscopy, cyclic voltammetry (CV), Stern–Volmer measurements and single‐crystal X‐ray diffraction were used to identify key reaction intermediates and products of the transformation. The reaction mechanism was proposed based on experimental findings and computational modeling.

## Results and Discussion

**Scheme 2:**
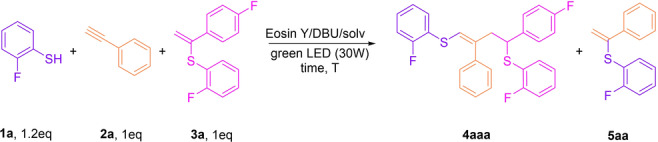
Eosin Y‐mediated intermolecular thiol–yne–ene reaction between compounds **1 a**, **2 a** and **3 a**.

Initial experiments were carried out using 2‐fluorobenzenethiol (**1 a**), phenylacetylene (**2 a**) and vinylsulfide (**3 a**) with Eosin Y as a catalyst (3 mol %) and DBU as a base under green light (530 nm) irradiation (Scheme 2). Under these conditions, desired product **4 aaa** was obtained in 7 % yield (entry 1, Table 1). Optimization of the reaction conditions revealed that it is important to gradually add reagent **1 a** to minimize the formation of side product **5 aa**. This byproduct results from an upconverted^[52]^ vinyldisulfide π*‐radical‐anion intermediate transformation (as described in our previous work).^[49]^ Therefore, reducing the relative thiolate anion amount or suppressing its nucleophilicity is a keystone of this multicomponent radical process. We built a custom‐made device for the controlled addition of reagents, thiol in particular to the reaction vessel (see Figure S10 in Supporting Information). The yield of the desired **4 aaa** product increased from 7 % to 48 % (entry 1 versus entry 7, Table 1). The use of other photocatalysts resulted in low **3 a** conversion (entries 2–4, Table 1), but two organic dyes, Bengal Rose and Fluorescein, gave good product **4 aaa** yields and can be considered as possible alternatives to Eosin Y (entries 5 and 6, Table 1). A more detailed study of the reaction conditions showed that the yield of product **4** depends on the rate of addition (RA) of the thiol: using conditions in which RA is higher at the beginning gave better yields than a constant rate. Varying the thiol addition rate, we obtained compound **4 aaa** in a yield of 66 % with Eosin Y as a photocatalyst and DMF as a solvent (entry 8, Table 1). The temperature increase to 40 °C decreased the yield of desired product **4 aaa** to 61 % (entry 9, Table 1). The application of MeOH as a solvent instead of DMF allows to achieve excellent selectivity with exclusive **4 aaa** formation (entry 10 vs entry 7, Table 1). Increasing the temperature to 40 °C and the initial addition of **1 a** improved the yield of **4 aaa** to 81 % (entry 11, Table 1). The yield dropped dramatically under these conditions when thiol was added at an exponential rate (entry 12, Table 1). Rose Bengal and Fluorescein gave 60 % and 75 % of product **4 aaa**, respectively, under optimized conditions. The control experiments revealed that the reaction does not proceed without irradiation of the reaction mixture **1 a**/**2 a**/**3 a**/Eosin Y; only a tiny amount of undesired disulfide was formed. Thus, Eosin Y/DBU/MeOH gave the highest yield of the desired product and provided the full suppression of **5 aa** formation (most likely via strong MeOH solvation of the thiolate anion), and we selected this system for further studies.

**Table 1:** Optimization of the intermolecular thiol–yne–ene reaction conditions.

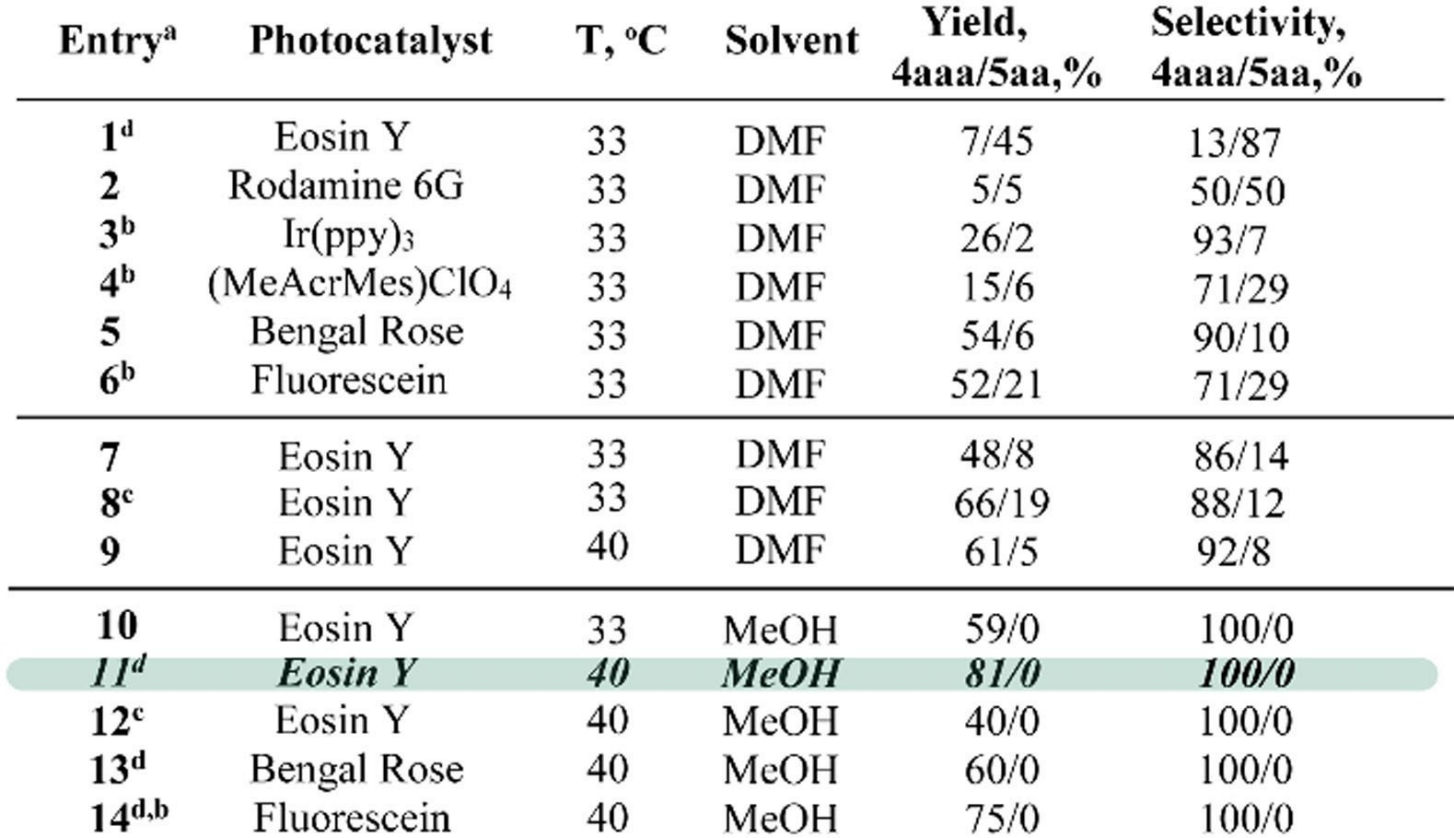

[a] **2 a** (0.12 mmol), **3 a** (0.1 mmol) and 3 mol % catalyst were added to 2 mL solvent. A 400 μL solution of **1 a** (0.15 mmol) and DBU (0.18 mmol) in solvent was added gradually by 1.33 μL within the reaction time. [b] LED 465 nm. [c] Rate of addition *r*=0.15 mmol*k *e* ^−*kt*^; *k*=1.71×10^−4^ s^−1^. [d] Initial addition of **1 a**.

**Scheme 3:**
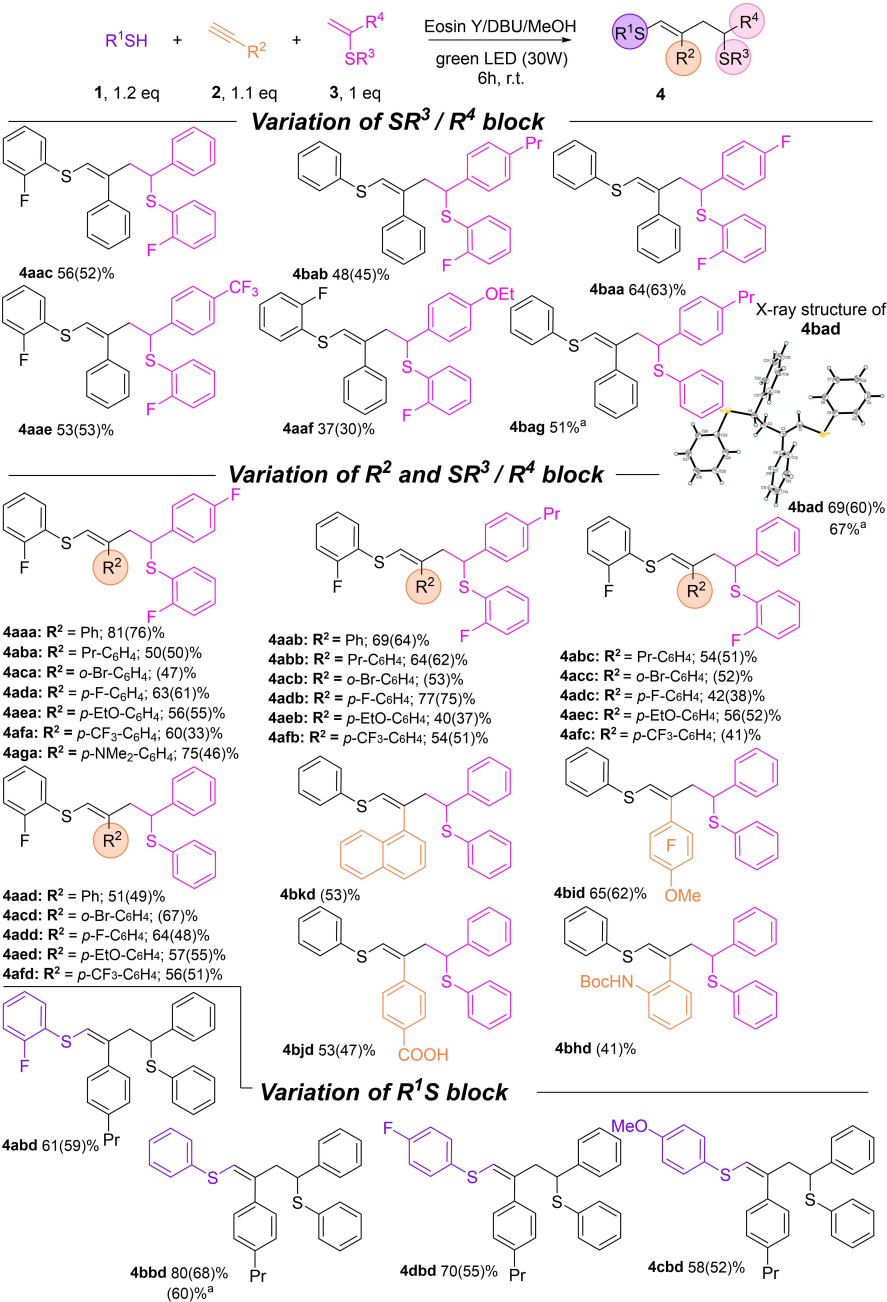
The scope of the three‐component thiol–yne–ene reaction. Yields were determined by ^1^H NMR, and isolated yields are shown in parentheses. [a] One‐pot approach: see the experimental part for detailed information.

Using the optimized synthetic conditions, we conducted three‐component photocatalytic reactions of different terminal alkynes with thiols and vinylsulfides. The scope of the photocatalyzed thiol–yne–ene reaction is summarized in Scheme 3. The yields of the products mostly range from good to high (50–81 %). Various terminal alkynes and vinylsulfides were used, and the reaction afforded the corresponding products (Scheme 3, variation of R^2^ and SR^3^/R^4^ block).

The use of aromatic alkynes gave products **4** in yields up to 81 %. The utilization of alkynes with electron‐donating (OEt—**4 aea**, **4 aeb**, **4 aec**, **4 aed**; NMe_2_—**4 aga**; NHBoc—**4 bhd**) and electron‐withdrawing groups (CF_3_—**4 afa**, **4 afb**, **4 afc**, **4 afd**; COOH—**4 bjd**, perfluoroaryl—**4 bid**) demonstrated good functional group tolerance in the studied reaction. The high catalytic activity of the Eosin Y/DBU system was observed for various aromatic thiols. The desired products were obtained in high yields with various substituents (Scheme 3, variation of R^1^S block). Different aromatic vinylsulfides were used in the thiol–yne–ene reaction, and products **4 aac**/**4 bab**/**4 baa**/**4 aae**/**4 bad** were obtained in 45–63 % isolated yields (Scheme 3, variation SR^3^/R^4^ block). The structure of ***Z*** **‐4 bad** was confirmed using a combination of 1D and 2D NMR experiments and X‐ray analysis.^[53]^ The synthetic procedure allowed the incorporation of fluorine substituents in various parts of the target molecule.

**Scheme 4:**
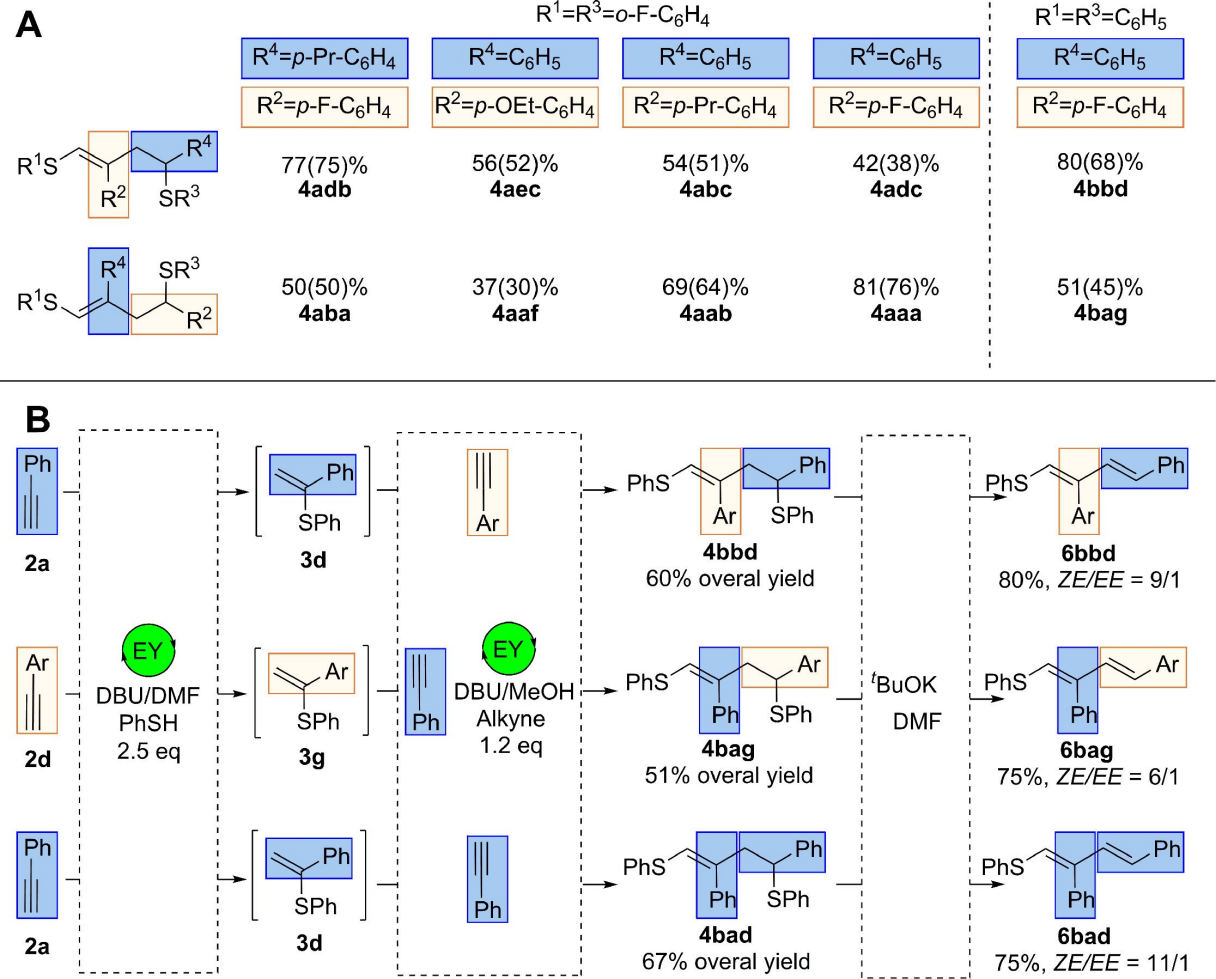
A) Pairs of products with controlled sequences of substituents. B) One‐pot synthesis and subsequent diene formation using strong base reaction conditions. Ar=*p*‐Pr‐C_6_H_4_.

Five pairs of products, listed in Scheme 4A, should be mentioned especially. These products have the same set of aryl substituents attached to the carbon skeleton, but in alternating order. For example, product **4 adb** differs from **4 aba** by substituents at the R^2^ and R^4^ positions (Scheme 4A). Developing this sequence control concept, we have undertaken the synthesis of three thiol–yne–ene coupling products starting from thiophenol and a pair of alkynes. Employing a previously published thiol–yne protocol,^[49]^ we obtained one of the components of the three‐component reaction, vinyl sulfides, in excellent yield (more than 90 %). Without further purification, the synthesized vinylsulfides were used in the studied reactions. As a result, the large‐scale two‐step photochemical syntheses of **4 bbd**, **4 bag** and **4 bad** were performed with high overall yields (Scheme 4B). Among the other possible transformations, elimination of thiol was chosen as a perspective route for product modification. It allows access to polyfunctionalized 1,3‐dienes, for which new methods of synthesis are in demand.[^54^, ^55^, ^56^]

Here, we demonstrate facile access to such S‐functionalized 1,3‐dienes—building blocks of high synthetic importance.[^50^, ^51^, ^57^, ^58^] As an illustrative example, the transformation of **4 bbd** under strongly basic conditions using ^t^BuOK‐DMF affords (**6 bbd**) phenyl((1*Z*,3*E*)‐4‐phenyl‐2‐(4‐propylphenyl)buta‐1,3‐dien‐1‐yl)sulfane in 80 % yield (Scheme 4B). The structure was confirmed by ^1^H and ^13^C{^1^H} NMR spectroscopy and NOE experiments. It should be noted that some activated dienes cannot be stored in pure form for a long time due to isomerization and decomposition. Therefore, it is important to have suitable precursors to easily generate such activated dienes. The present molecules match these requirements since compound **4** is stable and the RSH elimination process is easy to perform. We carried out the catalytic reaction at a larger scale to identify the generated byproducts. Product **4 bbd** was obtained in 60 % yield, and the E‐isomer of **4 bbd** was obtained in 8 %. This product was purified by column chromatography, and the structure was confirmed by ^1^H and ^13^C{^1^H} NMR spectroscopy and NOE experiments (see Figures S29, S30 for detailed information). Moreover, another side product was characterized by 1D and 2D NMR and MS/MS experiments and identified as a furan derivative (see Figure S8/S23–S27).

**Scheme 5:**
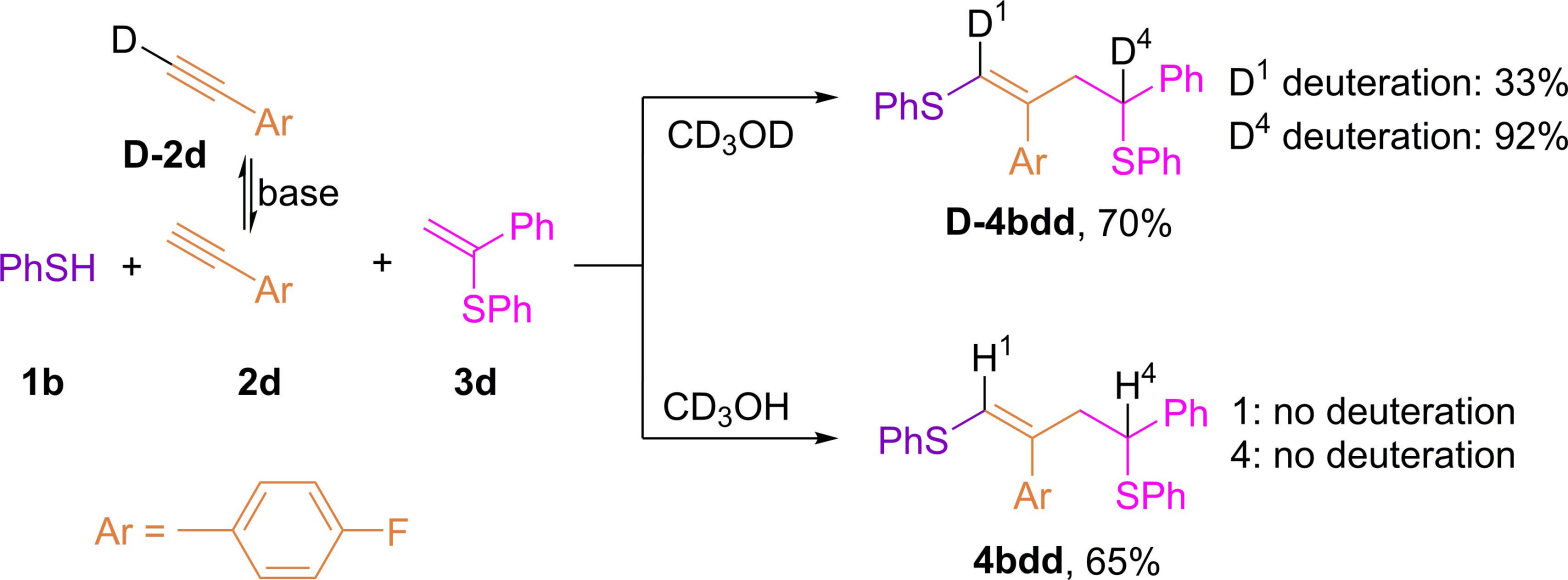
Three‐component thiol–yne–ene reaction in deuterated solvent. Ar=*p*‐F‐C_6_H_4_.

To elucidate the mechanism, the reaction was performed in deuterated methanol. (*Z*)‐(2‐(4‐fluorophenyl)‐4‐phenylbut‐1‐ene‐1,4‐diyl)bis(phenylsulfane) **D‐4 bdd** with 33 % deuteration at the first position (D^1^) and 92 % deuteration at the fourth position (D^4^) was produced in CD_3_OD and isolated in 70 % yield as determined by ^1^H, ^2^H and ^13^C{^1^H} NMR spectroscopy (Scheme 5, CD_3_OD). The reaction in CD_3_OH under the same conditions led to product **4 bdd** containing no deuterium atoms (Scheme 5, CD_3_OH). The reaction in CH_3_OH with alkyne, deuterated on its terminal position (**D‐2 d**), led to the product without deuterium atoms **4 bdd**. However, the same reaction in CD_3_OD gave **D‐4 bdd** with 100 % deuteration at positions 2 and 4. The results of these experiments showed that the solvent is the source of the hydrogen atom at the fourth position in the **4 bdd** skeleton. Thus, deuterium atoms were inserted through acid–base interactions with CD_3_OD rather than hydrogen atom transfer. Therefore, an almost stoichiometric (92 %) deuteration at the fourth position in the case of CD_3_OD can be explained by the formation of carbon‐centered benzyl anion **A2** (see Scheme 8), which may be the first piece of a mechanistic puzzle. The appearance of (D^1^) can be explained by the H/D exchange of weakly acidic hydrogen atom in alkyne under mild reaction conditions.

**Scheme 6:**
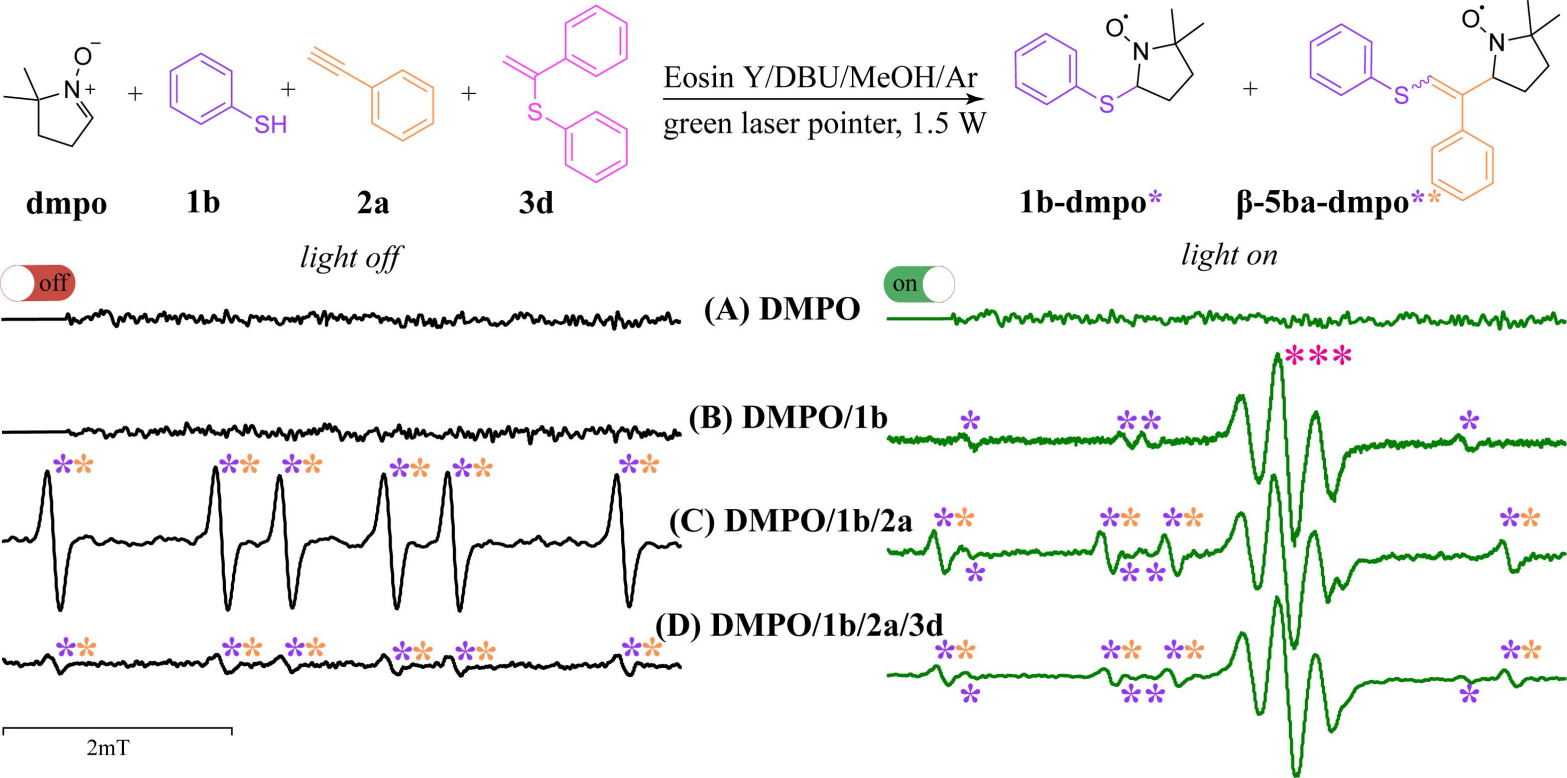
Series of EPR spectra for reaction mixtures with consecutive addition of reagents (black: without irradiation; green: with green light irradiation of the mixture). The signal of the radical anion Eosin Y^.−^ is marked as ***. Simulated spectra are presented in the Supporting Information.

To detect radical intermediates in situ during the reaction, we performed an electron paramagnetic resonance spectroscopy (EPR) investigation of the thiol–yne–ene reaction between thiophenol (**1 b**), alkyne (**2 a**), and vinylsulfide (**3 d**) under standard reaction conditions (Scheme 6). 5,5‐Dimethyl‐1‐pyrroline N‐oxide (DMPO) was used as a trapping reagent to detect free radicals by EPR.

As shown in Scheme 6, irradiation of reaction mixture **A** without reagents **1 b**/**2 a**/**3 d** did not lead to the formation of any detectable radicals. The signals of radical **1 b**‐dmpo* (*A*_H_=1.58 mT, *A*_N_=1.38 mT, g‐factor=2.0061)^[59]^ and Eosin Y^.−^ appear after oxidation of thiophenol by Eosin Y upon irradiation of mixture **B**. The presence of β‐5 ba‐dmpo** (*A*_H_=2.00 mT, *A*_N_=1.47 mT, g‐factor=2.0058)^[59]^ in mixture **C** without green light irradiation of the sample can be explained by the stability of this radical, formed in a dark equilibrium radical process. Green light irradiation of mixture **C** (**dmpo**/**1 b**/**2 a**) leads to a decrease in the intensity of the signal corresponding to β‐5 ba‐dmpo**, possibly due to consumption of β‐5 ba‐dmpo** in light‐mediated redox processes (for example, reduction of the nitroxyl radical to the corresponding O‐centered anion). No other radical species were observed in the EPR study of the final mixture **dmpo**/**1 b**/**2 a**/**3 d**. This may be explained by either no other radical species formation or some radicals being too bulky to react with the spin trap to be detected. On the other hand, the intensity of β‐5 ba‐dmpo** drops significantly in the presence of **3 d**, which implies additional channels of vinyl radical consumption, such as trapping by **3 d**.

In addition, radical trapping experiments were performed with γ‐terpinene and a **1 b**/**2 a**/**3 d** reaction mixture. Target product **4 bad** was not formed, indicating that radical intermediates are important for the transformation.

**Scheme 7:**
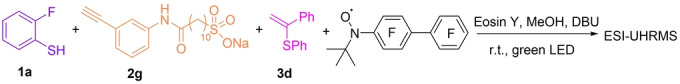
Reaction mixture studied by photo‐ESI‐UHRMS.

**Figure 1:**
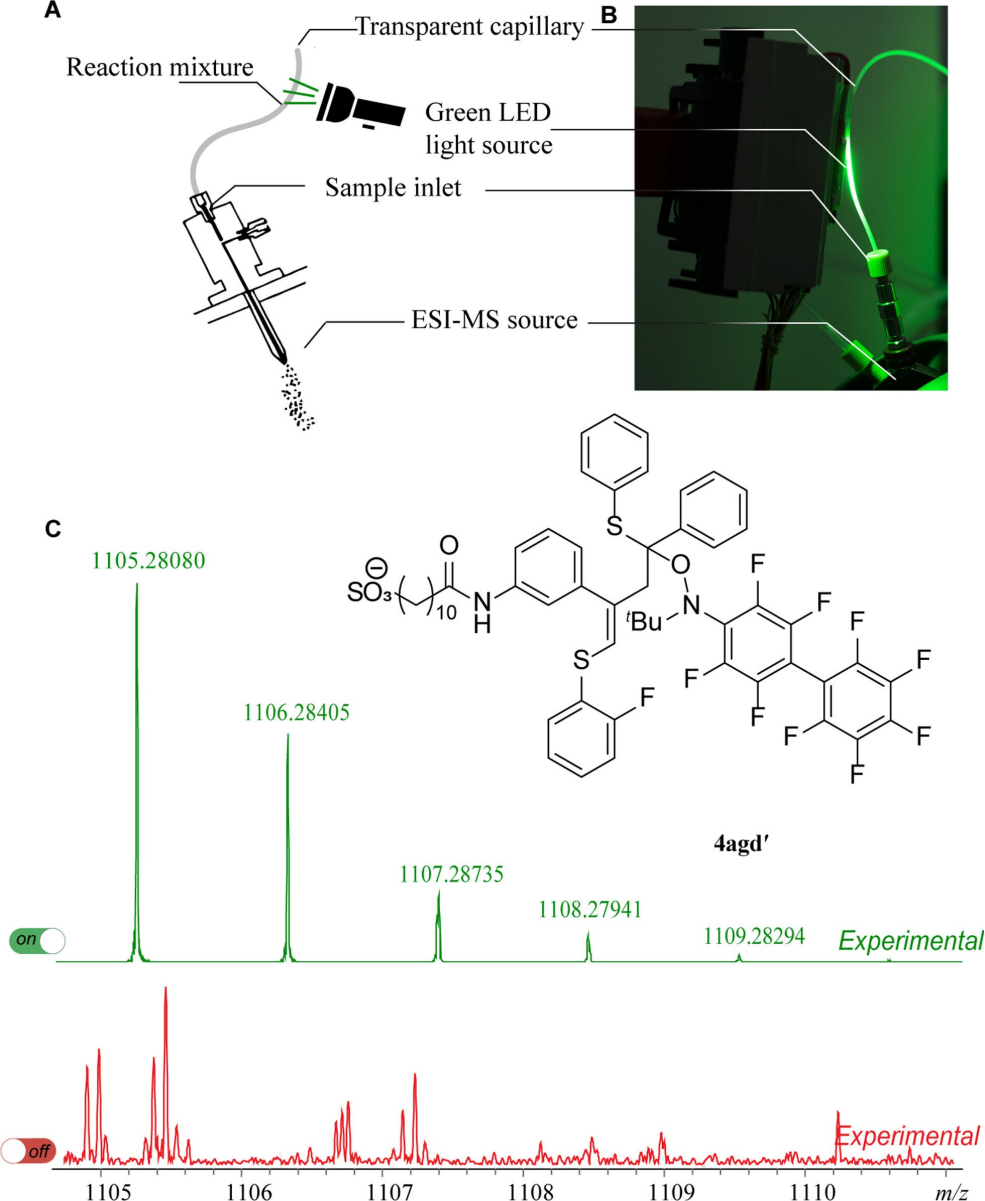
A) Custom‐made dedicated device for the ESI‐(−)UHRMS experiment. B) Photograph of the experimental setup: the reaction mixture was introduced into a transparent Teflon capillary, photoexcited by a green LED and passed into the mass spectrometer. C) Real‐time spectra under light irradiation (green colour) and the same region of MS spectra obtained without light irradiation (red colour) in negative‐ion mode.

Next, we applied a combination of ultrahigh resolution electrospray ionization mass spectrometry (ESI‐UHRMS) technique and a photochemistry approach.[^60^, ^61^, ^62^] FT‐ICR‐MS provides high accuracy, resolution and mass precision for the reliable identification of components.[^63^, ^64^] To reveal the nature of the observed intermediates, we performed the reaction inside a transparent capillary in the immediate vicinity of the ionization chamber of the mass spectrometer with the continuous pumping of the reaction mixture from the flask through the capillary. In the case of the thiol–yne–ene reaction, we placed a green LED close to the ion source of the mass spectrometer. The light beam was directed to the capillary connected to the ESI source where ionization occurs (Figure 1A; B). The thiol–yne–ene reaction between alkyne (**2 g**), 2‐fluorothiophenol (**1 a**), vinylsulfide **3 d** and N‐(*tert*‐butyl)‐N‐(perfluorobiphenyl‐4‐yl)oxylamine[^65^, ^66^] was chosen for ESI‐UHRMS investigation (Scheme 7). In the negative ion mode in the absence of green light irradiation, signals corresponding to alkyne **2 g**, 2‐fluoriothiophenol (**1 a**) and Eosin Y were dominant in the ESI mass spectra. Under green light irradiation of the capillary, molecular ions corresponding to product **4 agd** (accurate *m*/*z* 704.23477; exact *m*/*z* 704.23437 for [C_39_H_43_NO_4_S_3_F]^−^), trapped intermediate **4 agd′** (accurate *m*/*z* 1105.28080; exact *m*/*z* 1105.28059 for [C_55_H_51_N_2_O_5_S_3_F_10_]^−^) and trapped intermediate **β‐5 ag′** (accurate *m*/*z* 893.21447; exact *m*/*z* 893.21462 for [C_41_H_39_N_2_O_5_S_2_F_10_]^−^) began to appear in a couple of seconds after the start of irradiation (Figure 1C and Figure S7). When the light was switched off, the signals of **β‐5 ag′** and **4 agd′** decreased in intensity.

**Scheme 8:**
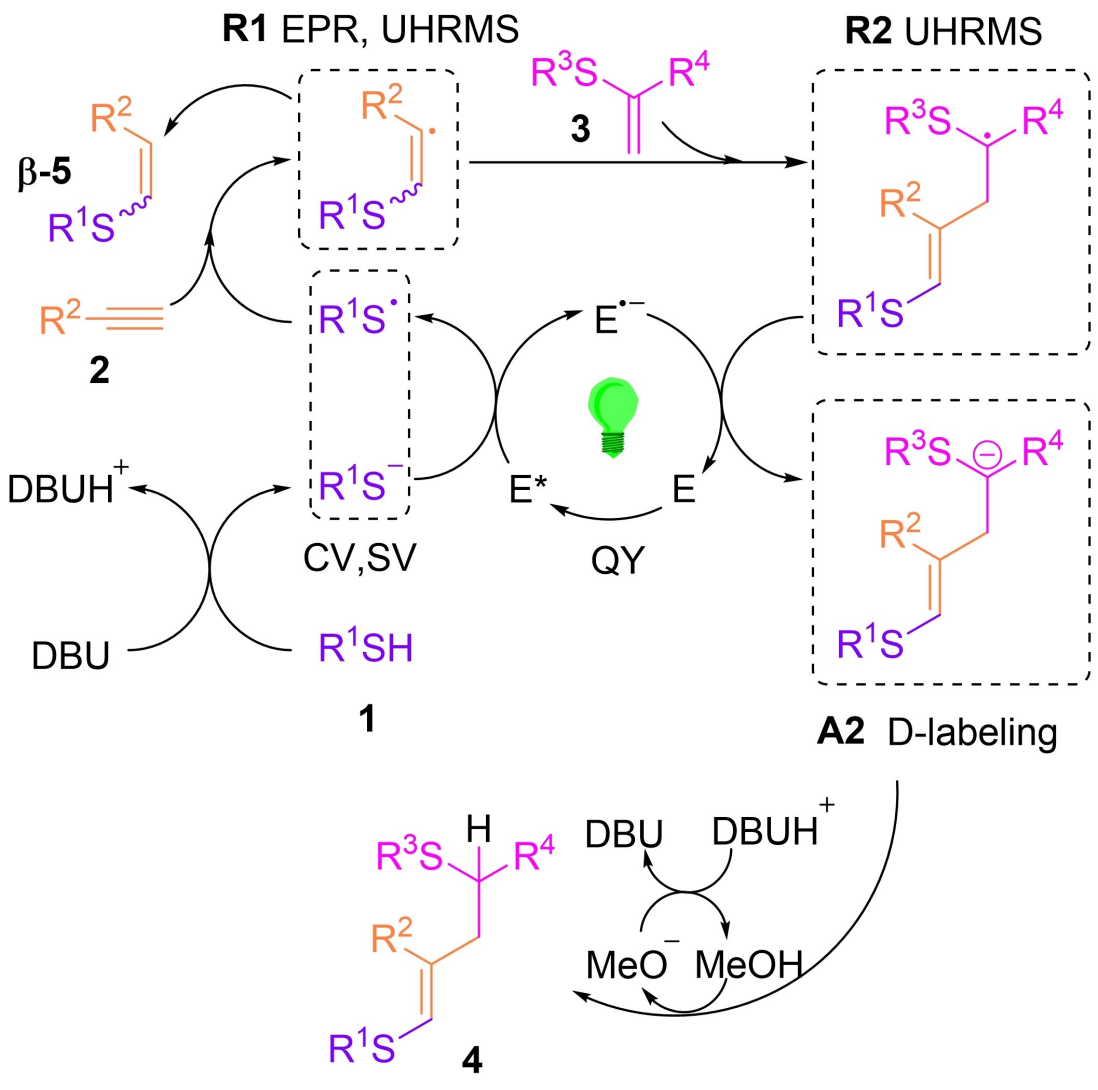
Plausible catalytic cycle of the photoredox thiol–yne–ene reaction. Dashed frames mark the experimentally identified intermediates.

The simultaneous appearance or disappearance of these peaks proved the key role of light in the formation of reactive intermediates **R1** and **R2** (Scheme 8). Next, continuous online ESI‐(−)UHRMS monitoring was conducted under green light irradiation of the Schlenk tube reaction vessel. For this purpose, we used alkyne **2 g** with an easily ionizable sulfonate group and carried out the thiol–yne–ene reaction between alkyne **2 g**, 2‐fluoriothiophenol (**1 a**) and vinylsulfide **3 d** under standard reaction conditions. This experiment allows real‐time monitoring of reagents (**2 g**, **1 a**), products and byproducts (see Figure S2 for details). To obtain additional information about the structure of the byproducts, collision‐induced dissociation (CID) was applied. The ions at *m*/*z*=546.1994, 492.1684, 626.1849, and 686.2206 were selected as precursor ions for ESI MS/MS CID experiments. In the MS/MS spectra, signals corresponding to loss of the alkyne **2 g** and thiol **1 a** fragments were detected. The ion at *m*/*z* 492.1684 was assigned as vinylsulfide **β‐5 ag** formed from the reaction between alkyne **2 g** and thiol **1 a**.

The following overall reaction mechanism can be proposed (Scheme 8). The catalytic cycle starts from Eosin Y, affording the thiyl radical to appear as the result of PET from thiolate‐anion R_1_S^−^ to the exited photocatalyst. The superiority of thiolate anions over another reactant in Eosin Y emission quenching supports the present hypothesis (*K*_SV_(PhS^−^)=13.8, *K*_SV_(PhSH)=3.7, *K*_SV_(vinylsulfide **3 d**)=1.7, for other reactants *K*_SV_≈0). Cyclic voltammetry (CV) measurements also demonstrate the thermodynamic feasibility exclusively of the discussed PET (*E*(eosin*(T_1_)/eosin^−^)=0.83 V,^[67]^ *E*(PhS⋅/PhS^−^)=0.28 V vs SCE). The corresponding CV and Stern–Volmer measurements are given in the Supporting Information (Figures S20, S21). Next, R^1^S⋅ interacts with alkyne **2**, producing **R1**, which can abstract a hydrogen atom yielding side product **β‐5**, and the corresponding ion was detected in the MS experiments. The addition of reagent **3** to **R1** results in the formation of **R2**. Reduction of **R2** with Eosin Y^.−^ led to anion formation. Anion **A2** could be protonated, which led to product **4**. To assess our hypothesis, the quantum yield (QY) was measured at an excitation wavelength of 528 nm for the reaction leading to **4 bad**. The QY of 1.9 % indicates a photocatalytic reaction without a significant contribution from radical chain processes. Therefore, radical **R2** has to be reduced to maintain the catalytic cycle. However, the only strong reductant in the system is the Eosin Y radical anion: *E*(eosin/eosin^.−^)=−1.06 V vs SCE.

**Figure 2:**
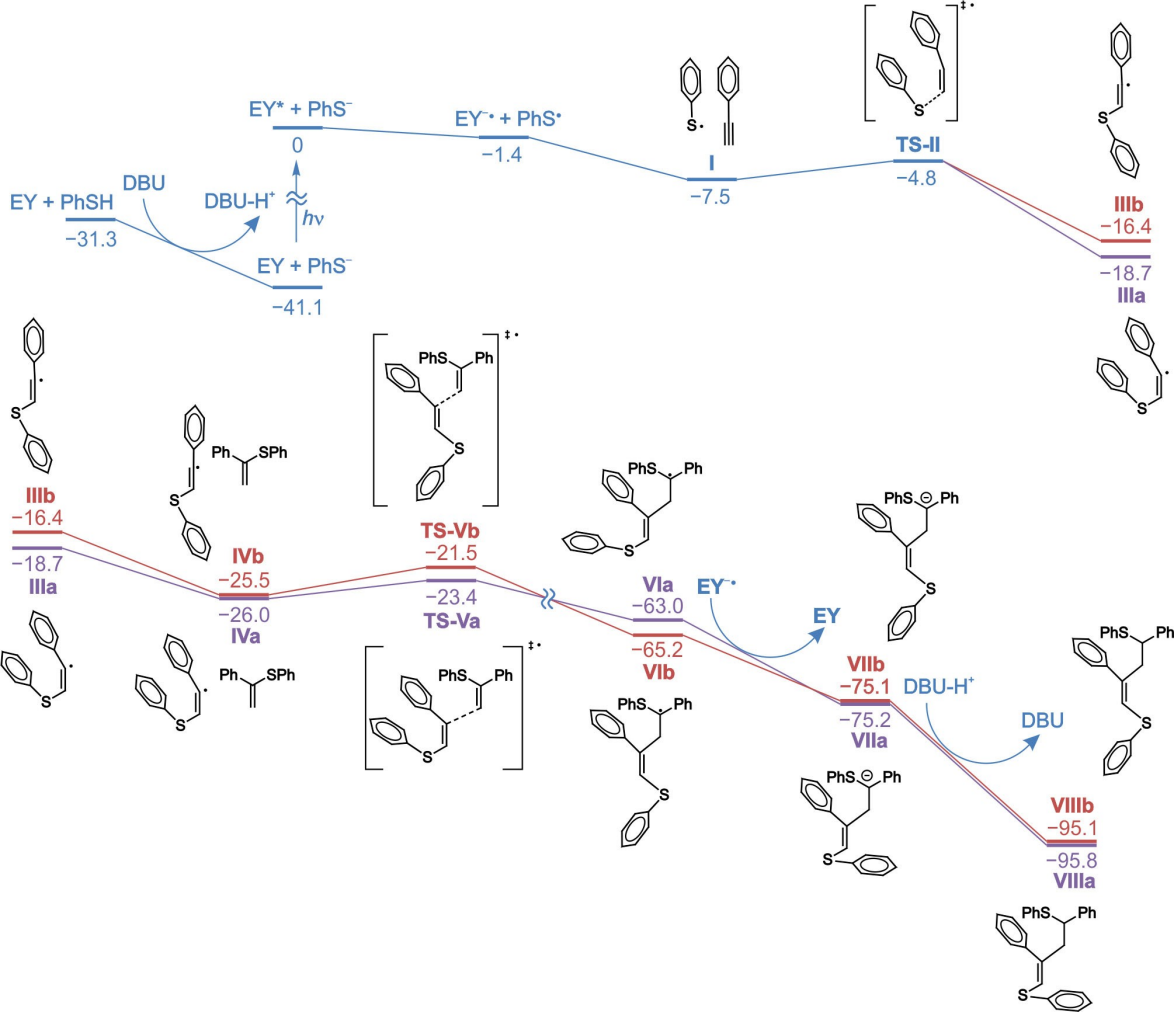
Energy profiles of three‐component reactions between **1 b**, **2 a** and **3 d**, leading to the *Z*‐isomer (path a; magenta colour) and the *E*‐isomer (path b; red colour) of product **VIII**. The calculations were performed at the PBE1PBE–D3BJ/6–311+G**&PCM (MeOH) level of theory.

To gain insight into the reaction mechanism and understand the reaction regioselectivity, the complete reaction path was modeled at the PBE1PBE‐D3BJ/6‐311+G**&PCM (MeOH) level of theory. In the first stage, DBU deprotonates thiophenol since the p*K*_a_ values for the acids DBU‐H^+^ (13.5 in water) and PhSH (6.62 in water) differ significantly. The energy of the thiophenol deprotonation step was calculated to be −9.8 kcal mol^−1^ (Figure 2). Then Eosin Y passed into an excited triplet state EY*. Singlet‐triplet excitation of Eosin Y destabilizes the system by 41.1 kcal mol^−1^, which is close to the value determined by the voltammetric method in a MeCN/H_2_O solvent mixture (43.6 kcal mol^−1^).^[67]^ The excited Eosin Y molecule absorbs an electron from the thiophenolate anion with the formation of a reactive radical PhS⋅. Electron transfer is an exergonic process (Δ*E*=−1.4 kcal mol^−1^). At the next stage, the radical PhS⋅ and phenylacetylene form associate **I**, followed by the formation of adduct **III**. The potential barrier of radical addition, as expected, has a very low value of Δ*E* (**I**→**TS‐II**)=2.7 kcal mol^−1^. No E‐isomer was found for compound **III**. Instead, there was a linear configuration of vinyl radical **IIIb** with a C=C−Ph angle close to 180°. *Z*‐isomer **IIIa** was 2.3 kcal mol^−1^ more stable than **IIIb**. Stabilization of **IIIa** is probably associated with the “through space” interaction of two phenyl substituents. Then, isomers **VI** were formed by adding vinyl radicals to the alkene. This step determines the *E*/*Z* selectivity, which is determined by the difference in the energies of transition states **TS‐V**. The energy of the **TS‐Va** structure is 1.9 kcal mol^−1^ lower than the energy of **TS‐Vb**. This means that the rate of **VIa** formation is approximately 33 times higher, which agrees with experimental data on the predominant formation of the *Z*‐isomer (Figure 2).

To examine the radical addition process to multiple bonds and radical center transfer to another carbon atom, we calculated how the spin population changes on the coupling fragments (Figure S19). During the reaction, a new C−C bond is formed, and the distance between carbon atoms decreases from 2.8 to 1.5 Å. The spin density, which reflects the localization of the unpaired electron, gradually flows from the vinyl radical (fragment A) to vinyl sulfide (fragment B). In the transition state, the distance between the combined carbon atoms is approximately 2.4 Å, and less than 20 % of the spin density is localized on fragment B. The spin populations of fragments **A** and **B** become equal when the distance between carbon atoms reaches 2.1–2.2 Å. With a further decrease in the distance between carbon atoms, the spin population completely shifts to fragment **B**. Note that the order of stability of isomers **VI** does not coincide with the orders of stability of compounds **IV** and **TS‐V**. The *E* isomer **VIb** is 2.2 kcal mol^−1^, which is more stable than the *Z* isomer **VIa**. Since the *Z*/*E* selectivity of the reaction under consideration is determined by the kinetic factor, the energy ratio of the **TS‐V** structures is of decisive importance. The reverse electron transfer from the Eosin Y doublet molecule to **VI** is an exergonic process (Δ*E*=−12.2 and−9.9 kcal mol^−1^ for paths **a** and **b**, respectively). As a result, Eosin Y returns to its initial state, and carbanions **VII** are formed. In addition, compound **VII** readily deprotonates the cation DBU‐H^+^ (Δ*E*=−20.6 and−20.0 kcal mol^−1^ for pathways **a** and **b**, respectively) to form products **VIIIa** and **VIIIb**.

To confirm the reliability of the theoretical modeling, calculations were also performed using the B3LYP and M062X functionals in both the condensed and the gas phases (Figures S14–S18). The obtained data concerning the *Z*/*E* selectivity of the process are consistent with each other (Table S9): in all cases, transition state **TS‐Va** is more stable by 1.4–2.6 kcal mol^−1^ (in methanol) and 1.9–2.1 kcal mol^−1^ (in the gas phase). Continuous medium, in this case, does not affect the selectivity of the process. Thus, the performed PBE1PBE, B3LYP, and M062X calculations yield similar results and correlate well with our experimental findings.

## Conclusion

In summary, the first example of an intermolecular thiol–yne–ene coupling reaction has been developed and studied in detail. A controlled three‐component transformation in thiol–yne chemistry has been realized, taking the reaction to a new level of molecular complexity. The desired products are obtained with good yields and selectivity under metal‐free photocatalytic conditions. The fundamental problem of three‐component radical processes has been solved by suppressing the most likely vinyl radical consumption pathways, such as HAT and associative reductant upconversion. Thus, the key role of such stabilization of the vinyl radical in the studied reaction opens up many possibilities for developing new three‐component processes. The mechanistic toolbox presented in this work has proven its value in investigating key steps of such transformations.

**Scheme 9:**
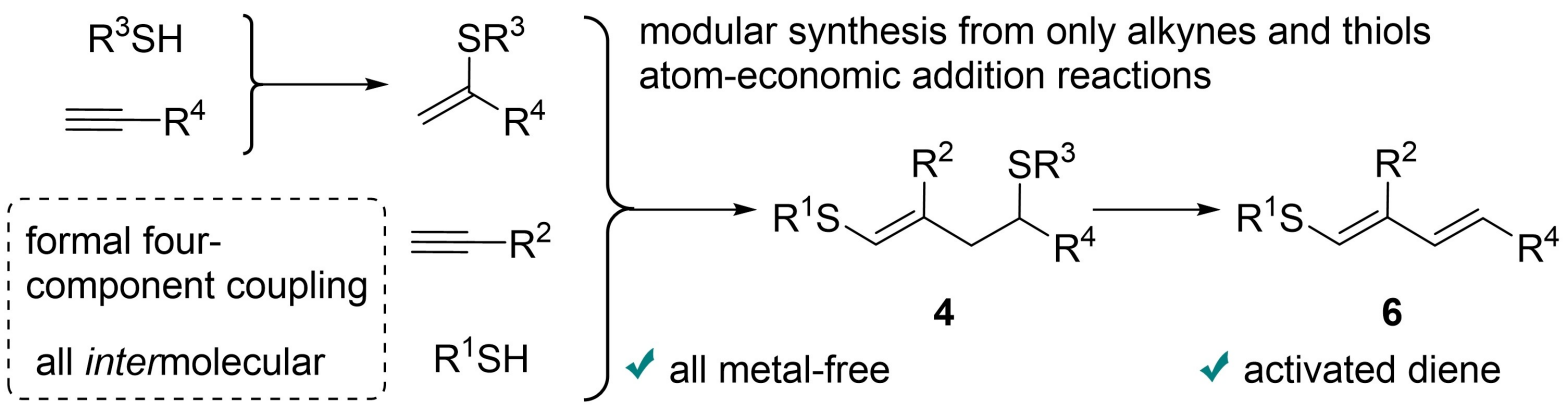
The overall approach demonstrated in this work.

Discussing the overall transformation, alkynes and thiols are the only starting materials used, and the process can be considered a formal four‐component coupling (Scheme 9). In the first stage, the reaction between the alkyne and thiol forms α‐vinylsulfide (thiol–yne coupling). In the next stage, a regioselective thiol–yne–ene reaction is carried out, where other alkyne and thiol molecules may be involved. Implementation of such a sequential transformation represents an ordered head‐to‐tale coupling of two different alkynes accompanied by heterofunctionalization. Amazingly, the described strategy of radical multicomponent coupling demonstrates even better selectivity than many metal‐based catalytic systems. The two‐step photoredox one‐pot synthesis of stable precursor **4** to access activated 1,3‐diene **6** is of great practical use. Atom‐economic addition reactions are involved in both steps. The synthesis of the intermediate vinylsulfide is a highly selective photoredox process, where the product does not require purification to enter the next reaction stage. Elimination of R^3^SH is easy to perform (Scheme 9), and the thiol can be recycled for use in the next synthetic sequence. Merging metal‐free photoredox transformations with a waste‐minimized synthetic strategy led to high selectivity in this new synthetic method.

## Conflict of interest

The authors declare no conflict of interest.

1

## Supporting information

As a service to our authors and readers, this journal provides supporting information supplied by the authors. Such materials are peer reviewed and may be re‐organized for online delivery, but are not copy‐edited or typeset. Technical support issues arising from supporting information (other than missing files) should be addressed to the authors.

Supporting Information

## Data Availability

The data that support the findings of this study are available in the Supporting Information of this article.
