# Supplementary material for: Intermolecular Photocatalytic Chemo‐, Stereo‐ and Regioselective Thiol–Yne–Ene Coupling Reaction
Source: Angew Chem Int Ed Engl. 2022 Mar 2;61(17):e202116888. doi: 10.1002/anie.202116888 (PMC9313788; doi:10.1002/anie.202116888)
Supplement: Supplementary file 1 — Supporting Information [file ANIE-61-0-s001.pdf]

Angewandte Chemie
Eine Zeitschrift der Gesellschaft Deutscher Chemiker

Supporting Information

Intermolecular Photocatalytic Chemo-, Stereo- and Regioselective Thiol–Yne–Ene Coupling Reaction

J. V. Burykina, A. D. Kobelev, N. S. Shlapakov, A. Y. Kostyukovich, A. N. Fakhrutdinov, B. König, V. P. Ananikov**

Supplementary Information

Intermolecular Photocatalytic Chemo-, Stereo- and Regioselective Thiol-Yne-Ene Coupling Reaction

Julia V. Burykina,[a]+ Andrey D. Kobelev,[a,b]+ Nikita S. Shlapakov,[a,c] Alexander Yu. Kostyukovich,[a] Artem N. Fakhrutdinov,[a] Burkhard König,[c]* Valentine P. Ananikov. [a,b]*

[a] *Zelinsky Institute of Organic Chemistry, Russian Academy of Sciences, Leninsky prospect 47, Moscow, 119991 Russia*
[b] *Lomonosov Moscow State University, Leninskie Gory GSP-1, 1-3, Moscow, 119991 Russia*
[c] *Institut für Organische Chemie, Universität Regensburg, Universitätstrasse 31, 93053 Regensburg, Germany.*
**e-mail: Burkhard.Koenig@chemie.uni-regensburg.de; val@ioc.ac.ru*
[+] *These authors contributed equally*

Table of Contents

Experimental section S2
Optimization of reaction conditions S5
Electrospray ionization mass spectrometry investigations S8
Design of the photoreactor S15
EPR investigation S16
DFT calculations S18
Cyclic voltammetry of selected components S25
Stern-Volmer measurements s25
X-ray Crystal Structure Determination of 4bad S27
Characterization data of products S37
Copies of NMR spectra of products S49
References S100

Experimental section

General considerations. Chemicals were obtained from commercial sources (Merck, Sigma-Aldrich, ABCR, and Alfa-Aesar) and used as received. Preparative column chromatography was performed using silica gel 60 (230–400 mesh, Carl Roth). A low-concentration tuning mix for MS calibration was obtained from Agilent Technologies. Reactions were performed in ROTILABO® flat-bottom vials with fine thread ND18, clear glass, 10 ml.
The samples for the ESI-TOF-MS experiments were prepared in 1.5 mL Eppendorf tubes (MeCN solutions). Aliquots were taken using Hamilton syringes.

NMR measurements were performed using a Bruker AVANCE 600 spectrometer operating at 600.1, 564.6, 150.0 MHz for ^{1}H, ^{19}F, and ^{13}C, respectively, and a Bruker AVANCE 500 spectrometer operating at 500.1, 125.8 MHz for ^{1}H and ^{13}C, respectively, Bruker Avance III 400 spectrometer operating at 400.1, 100.6 MHz for ^{1}H and ^{13}C, respectively, or using a Bruker Fourier 300HD spectrometer at 300.1, 75.5 MHz for ^{1}H and ^{13}C, respectively, in $CDCl_3$, DMF-d_8, and acetone-d_6. All the measurements were performed at room temperature and processed using the TopSpin 3.5 or TopSpin 4.0 software package. The ^{1}H and ^{13}C chemical shifts were referenced to residual solvent signals, relative to TMS as an external standard for spectra in $CDCl_3$, or relative to C_6F_6 as an external standard for ^{19}F spectra.[1]

ESI-MS TOF experiments. High-resolution mass spectra were recorded on a Bruker maXis Q-TOF instrument (Bruker Daltonik) equipped with an electrospray ionization (ESI) source. The measurements were performed in positive (+) MS ion mode (HV capillary: 4.5 kV; spray shield: –0.5 kV) and (–) MS ion mode (HV capillary: 3.0 kV; spray shield: −0.5 kV) with a scan range of *m/z* 50 − 1500. External calibration of the mass spectrometer was performed using a low-concentration tuning mix solution (Agilent Technologies). Direct syringe injection was applied to the solution in MeCN (flow rate: 5 μL×min^{-1}). Nitrogen was used as the nebulizing (1.0 bar) and drying gas (200 °C, 4.0 L×min^{-1}). All spectra were recorded at 1 Hz frequency and processed using the Bruker Data Analysis 5.1 software package.

X-ray crystallographic data and refinement details X-ray diffraction data were collected at 100 K on a Bruker Quest D8 diffractometer equipped with a Photon-III area-detector (graphite monochromator, shutterless ω-scan technique) using Mo Kα radiation. The intensity data were integrated by the SAINT program (Bruker. APEX-III. Bruker AXS Inc., Madison, Wisconsin, USA, 2019) and were corrected for absorption and decay using SADABS.[2] The structure was solved by direct methods using SHELXT[3] and refined on F2 using SHELXL-2018.63 All nonhydrogen atoms were refined with anisotropic displacement parameters. Hydrogen atoms were placed in ideal calculated positions and refined as riding atoms with relative isotropic displacement parameters; a rotating group model was applied for methyl groups. The SHELXTL program suite (Bruker. APEX-III. Bruker AXS Inc., Madison, Wisconsin, USA, 2019) was used for molecular graphics.

Computational details. Calculations were carried out within the framework of density functional theory in the Gaussian program.[4] The PBE1PBE,[5] B3LYP[6] and M062X[7] functionals were used for the calculation of unrestricted open-shell wave functions. Empirical Grimme dispersion correction (D3BJ)[8] was added for the PBE1PBE and B3LYP functionals. Geometry optimization and calculation of molecular vibration frequencies were calculated using the 6-311+G** basis set.[9,10] The calculations were performed both in the gas phase and in a continuous solvent medium (PCM, MeOH).[11] All transition states had one imaginary vibrational frequency, and their correctness was confirmed by IRC calculations.[12] Conformational analyses were carried out for radical intermediates **VIa** and **VIb**. It consisted of four sequential scans of

the potential energy surface along dihedral angles (Figure S13). Furthermore, the reaction pathways were calculated for the most stable conformers found.

Synthesis of product 4 in DMF. Vinylsulfide **3** (0.1 mmol), 1,8-diazabicyclo[5.4.0]undec-7-ene (0.18 mmol, 27 µl) and Eosin Y (3 mol. %, 2 mg) were dissolved in 2 ml DMF. Argon was bubbled through the reaction mixture for 5 min. Alkyne **2** (0.12 mmol) was added in an argon flow. The vial with the reaction mixture was capped, and a syringe with 400 µl of a stock solution of thiol **1** (0.15 mmol) in DMF was inserted (needle was immersed into the reaction mixture). After the reaction mixture was irradiated by green light (525 nm, 30 W diode) for 6 h at 40 °C, 400 µl of stock solution was added during the reaction time according to the chosen program.

Synthesis of product 4 in MeOH. Vinylsulfide 3 (0.1 mmol), 1,8-diazabicyclo[5.4.0]undec-7-ene (0.15 mmol, 23 µl) and Eosin Y (3 mol. %, 2 mg) were dissolved in 2 ml MeOH. Argon was bubbled through the reaction mixture for 5 min. Alkyne **2** (0.12 mmol) and thiol **1** (0.15 mmol) were added in argon flow. Vial with the reaction mixture was irradiated by green light (525 nm, 30 W diode) for 6 h at 40 ºC. After completion of the reaction, the solvent was evaporated, and the residue was purified by column chromatography (petroleum ether/DCM/triethylamine 4:1:0.02).

H/D exchange reaction. Vinylsulfide **3d** (0.1 mmol), 1,8-diazabicyclo[5.4.0]undec-7-ene (0.15 mmol, 23 µl) and Eosin Y (3 mol. %, 2 mg) were dissolved in 2 ml of CD_3OD (or CD_3OH, or CH_3OH). Argon was bubbled through the reaction mixture for 5 min. Alkyne **2d** or **D-2d** (0.11 mmol) and thiol **1b** (0.12 mmol) were added to the argon flow. Vial with the reaction mixture was irradiated by green light (525 nm, 30 W diode) for 6 h at 40 ºC. After completion of the reaction, the solvent was evaporated, and the residue was purified by column chromatography (petroleum ether/DCM/triethylamine 4:1:0.02).

Synthesis of product 6bbd. To a solution of 0.06 mmol **4bbd** in 2 ml DMF, 0.3 mmol of ^{t}BuOK was added. The reaction mixture was stirred for 2 days at room temperature. Thirty milliliters of Et_2O was added to the reaction mixture, and the organic phase was washed 3 times with a 30 ml concentrated solution of NaOH in water. Further purification of the product by column chromatography was not employed due to decomposition of substances on silica gel and aluminum oxide.

One-pot synthesis. DBU (7 mmol) and Eosin Y (0.03 mmol) were dissolved in 31 ml of DMF in a 250 ml flask to maximize the area of irradiation. The mixture was degassed under low pressure and filled with argon 3 times. Then, 2.6 mmol of phenylacetylene **2a** and 5.2 mmol of thiophenol **1a** were added to the mixture. The reaction was carried out overnight at 40 °C with four 30 W green LEDs. Then, 2.6 mmol of phenylacetylene **2a**, 2 mmol of thiophenol **1a**, 2 mmol of DBU and 20 ml of bubbled argon MeOH were added to the reaction flask. The reaction was carried out overnight at 40 °C with four 30 W green LEDs.

Gram-scale synthesis of 4bbd. Vinylsulfide **3d** (1.5 mmol), 1,8-diazabicyclo[5.4.0]undec-7-ene (3.7 mmol, 550 µl) and Eosin Y (1 mol. %, 10 mg) were dissolved in 35 ml MeOH. Argon was bubbled through the reaction mixture for 5 min. Alkyne **2b** (2.3 mmol) and thiol **1b** (2.5 mmol) were added in argon flow. The reaction was carried out overnight at 40 °C with four 30 W green LEDs. After completion of the reaction, the white precipitate was filtered and washed with MeOH. Then, the solvent was evaporated from the filtrate, and the residue was purified by column chromatography (petroleum ether/DCM/triethylamine 4:1:0.02).

General procedure of the ESI-UHRMS study. Solution of N-(tert-butyl)-N-(perfluorobiphenyl-4-yl)oxylamine (2.5 µmol, 1 mg), **1a** (5.1 µmol, 0.66 mg), DBU (5.6 µmol, 0.86 mg), **2g** (1.6 µmol, 0.6 mg), **3d** (12.7 µmol, 2.7 mg), Eosin Y (0.04 µmol, 0.025 mg) in 1 ml of MeOH. Argon was bubbled through a nontransparent vial with the reaction mixture for 5 min. Three hundred microliters of the mixture was taken with a Hamilton syringe and wrapped in aluminum foil. A syringe was inserted into the dispenser and connected to the nebulizer through a transparent Teflon capillary. Ultrahigh resolution mass spectra (ESI-UHRMS) were acquired on a Bruker SolariX XR ion cyclotron resonance Fourier transform MS (Bremen, Germany) equipped with a 15 Tesla superconducting magnet and an Apollo II source in negative electrospray ionization mode. The sample was injected with a constant flow rate of 120 µl/h, nebulizer gas pressure of 1 bar and drying gas (200 °C, 4.0 L×min^{-1}). The accumulation time was 0.1 sec, the number of scans was 256, and the *m/z* range was 150–3000. The applied ESI voltage was a 3.6 kV capillary voltage and a −0.5 kV end plate offset. All spectra were acquired using a time transient of 2 MW. Transfer optic parameters were therefore ToF 1.3 msec, frequency 4 MHz and RF amplitude of 350 Vpp.

EPR measurements of the four mixtures were performed using a SPINSCAN X EPR spectrometer (ADANI). Mixture **A**: DMPO (1 mg, 9 µmol), Eosin Y (1 mg, 1.4 µmol), DBU (12 µl, 80 µmol) in 1 ml of MeOH. Mixture **B**: components of mixture **A** and PhSH (7 µl, 70 µmol). Mixture **C**: components of mixture **B** and phenylacetylene **2a** (10 µl, 90 µmol). Mixture **D**: components of mixture **C** and **3d** (17 mg, 80 µmol). Each mixture was bubbled with argon and taken into a glass capillary, which was inserted into the EPR tube. The first scanning was performed without irradiation. Then, the capillary was irradiated with 1.5 W green laser pointer for 20 seconds before the next scan and during it. Scanning parameters: Modulation amplitude: 1G. Microwave power: 117.957 mW. Microwave frequency: 9.435 GHz. The obtained spectra were smoothed using the scipy.signal.lfilter python library. Simulated and experimental spectra are presented in Figures S11 and S12.

Cyclic voltammetry experiments were performed using IKA ElectraSyn 2.0. The working electrode was a glassy carbon electrode; a carbon electrode was used as the counter electrode. Ag wire in 3 M KCl water solution was used as the reference electrode. PhSH + DBU (15 mM + 22.5 mM) solution was prepared by dissolving thiophenol (8.3 mg) and DBU (17.1 mg) in 5 ml of methanol. PhSH (15 mM) solution was prepared by dissolving thiophenol (8.3 mg) in 5 ml of methanol. DBU (22.5 mM) solution was prepared by dissolving DBU (17.1 mg) in 5 ml of methanol. 171 mg (0.1 M) of $(n\text{-Bu})_4NClO_4$ was added to each mixture to increase the conductivity of the solutions. Each sample was bubbled with argon.

Optimization of reaction conditions

The following procedure was chosen for optimization of the photocatalyst:

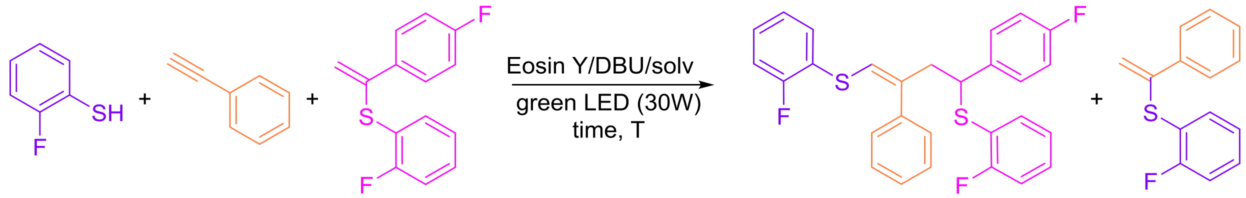

1a **2a** **3a** **4aaa** **5aa**

Table S1. Time optimization.

Time	4aaa, %	5aa, %	Conversion of 3a, %
2h	34	1	92
4h	29	5	88
6h	49	5	84

Reaction conditions: alkyne **2a** (0.15 mmol), vinylsulfide **3a** (0.15 mmol) and 3 mg eosin Y were added to 2 ml DMF. A 400 µl solution of **1a** (0.18 mmol) and DBU (0.24 mmol) in DMF was added gradually by 1.33 µl within the reaction time. (The reaction was carried out in an Ar atmosphere under 30 W green LED irradiation). T = 33 ˚C. The reaction time was 6 h because longer reaction times did not further improve the yield. (2-Fluorophenyl)(1-(4-fluorophenyl)vinyl)sulfane **3a** was synthesized under a previously published procedure and used in further synthesis without purification.[13]

Table S2. Photocatalyst optimization.

Photocatalyst/light	4aaa, %	5aa, %	Conversion of 3a, %
Eosin Y/green	**48**	8	89
Fluorescein/blue	**52**	21	81
Bengal Rose/green	**54**	6	78
Rhodamine 6G/green	5	5	33
Rhodamine 6G (20 mg)/blue	0	0	0
$Ir(ppy)_3$/blue	26	2	100
$(MeAcrMes)ClO_4$/blue	14.5	5	48
$[Ir(CF_3\text{-}ppy)_2(dtbbpy)]PF_6$/blue	15.4	24	32

The photocatalyst was optimized under the following reaction conditions: alkyne **2a** (0.12 mmol), vinylsulfide **3a** (0.1 mmol) and 3 µmol of catalyst were added to 2 ml DMF, and the solution was bubbled with argon. Four hundred microliters of bubbled argon solution of **1a** (0.15 mmol) and DBU (0.18 mmol) in DMF was added gradually by 1.33 µl within the reaction time. The reaction was carried out under 30 W LED irradiation and 33 °C for 6 hours.

Table S3. Temperature optimization.

Temperature, °C	4aaa, %	5aa, %	Conversion of 3a, %
25	32	3	61

33	48	8	89
40	61	5	93
50	44	7	88

The temperature was optimized under the following reaction conditions: alkyne **2a** (0.12 mmol), vinylsulfide **3a** (0.1 mmol) and 3 μmol of eosin Y were added to 2 ml DMF, and the solution was bubbled with argon. Four hundred microliters of bubbled argon solution of **1a** (0.15 mmol) and DBU (0.18 mmol) in DMF was added gradually by 1.33 μl within the reaction time. The reaction was carried out under 30 W LED irradiation for 6 hours.

Table S4. Optimization of thiol addition.

Rate law	Time, h	4aaa, %	5aa, %	Conversion of 3a, %
$r \rightarrow \infty$	6	7	44	51
r = 0.025 mmol/h	6	48	8	89
$\Delta n/\Delta t = 5*10^{-4}$mmol/Δt<br>Δt=10s+0.415t	6	55	6	93
r = 0.15mmol * ke^{-kt};<br>$k=1.71*10^{-4}\ s^{-1}$	6	**66**	19	93
r = 0.15mmol * ke^{-kt};<br>$k=7.1*10^{-5}\ s^{-1}$	10	60%	20%	82

The rate of addition of thiol **1a** was optimized under the following reaction conditions: alkyne **2a** (0.12 mmol), vinylsulfide **3a** (0.1 mmol) and 3 μmol of eosin Y were added to 2 ml DMF, and the solution was bubbled with argon. Four hundred microliters of bubbled argon solution of **1a** (0.15 mmol) and DBU (0.18 mmol) in DMF was added gradually as a function of rate over time. The reaction was carried out under 30 W LED irradiation for 6 hours at 33 °C.

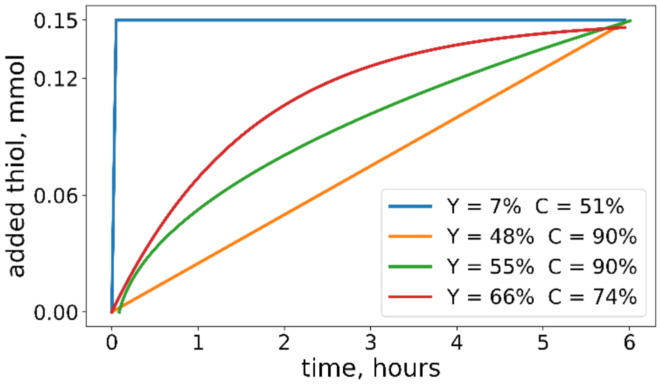

Figure S1. Influence of the law of **1a** addition on the product yield (Y) and conversion (C) of **3a**.

Table S5. Solvent optimization.

Solvent	4aaa, %	5aa, %	Conversion of 3a, %
DMF	6	20	80
DCE	0	0	25
NMP	9	5	83
MeOH	**59**	0	92
DMA	19	7	70
MeCN	30	traces	9

The solvent was optimized under the following reaction conditions: alkyne **2a** (0.12 mmol), vinylsulfide **3a** (0.1 mmol) and 3 µmol of eosin Y were added to 2 ml of solvent, and the solution was bubbled with argon. Eighty microliters of bubbled argon solution of **1a** (0.03 mmol) and DBU (0.04 mmol) in the chosen solvent was added gradually by 1.33 µl for 3 hours. Then, 320 µl of bubbled argon solution of **1a** (0.12 mmol) and DBU (0.15 mmol) in the chosen solvent was added to the reaction mixture. The reaction was carried out under 30 W LED irradiation for 6 hours at 33 °C.

Table S6. Additional optimization in protic solvents.

T, ˚C	Solvent	Addition mode	Catalyst	4aaa, %	5aa, %	Conversion of 3a, %
40	MeOH	Initial addition	Eosin Y	**81**	0	100
40	MeOH	Initial addition	Without catalyst	**-**	-	-
40	MeOH	Initial addition	Eosin Y/without light	**-**	-	-
21	MeOH	Initial addition	Eosin Y	69	0	100
40	MeOH	Initial addition	3DPA2FBN	43	0	100
40	MeOH	Initial addition	Fluoresceine	75	0	100
40	MeOH	Initial addition	Bengal Rose	60	0	100
40	EtOH	Initial addition	Eosin Y	73	0	100
40	MeOH	r(**3a**) = const	Eosin Y	19	0	60
40	MeOH	r(**1a**) = 0.15mmol * ke^{-kt}, $k=1.71*10^{-4}$ s^{-1}	Eosin Y	40	0	95

Then, final optimization was performed for protic solvents under the following reaction conditions: alkyne **2a** (0.12 mmol), DBU (0.18 mmol), and 3 µmol of photocatalyst were added to 2 ml of solvent, and the solution was bubbled with argon. vinylsulfide **3a** (0.1 mmol) and thiol **1a** (0.15 mmol) were added according to the addition mode. The reaction was carried out under 30 W LED (green for Eosin Y and blue for 3DPA2FBN) irradiation for an overall 6 hours at the chosen temperature.

The controlled experiments revealed that the reaction does not proceed without irradiation of reaction mixture **1a**/**2a**/**3a**/Eosin Y, and only a tiny amount of undesired disulfide was formed.

Electrospray ionization mass spectrometry investigations

The synthesis of sodium 11-((3-ethynylphenyl)amino)-11-oxoundecane-1-sulfonate (**2g**) was performed according to a published procedure[13]

ESI-(–)MS online monitoring of the photocatalytic thiol-yne click reaction

Alkyne (**2g**) (7.7 mg, 2*10^{-5} mol), 1,8-diazabicyclo[5.4.0]undec-7-ene (DBU) (4.6 mg, 3*10^{-5} mol), arylthiol (**1a**) (3.2 mg, 2.5*10^{-5} mol), vinylsulfide **3d** (6.3 mg, 3*10^{-5} mol) and 0.4 ml of methanol were mixed in a round-bottom flask. A Schleck tube (10 ml) equipped with a magnetic stir bar was filled with 400 µl of the prepared solution, and 0.1 mg (1.4*10^{-7} mol) of eosin Y was added followed by 3.6 ml of MeOH. A neck was closed with a silicon septum, and the side tap was connected to an argon double balloon. A PEEK capillary connected to the ESI source was pulled into the flask through the septum and immersed into the reaction mixture. The tube was placed into a setup equipped with a green LED 1.25 W (λ_{max}=533 nm), and reaction monitoring was started after the stabilization of the total ion current. The reaction was stirred at room temperature for 0.5 h, and spectra were recorded in negative ion mode for 4.30 min without light irradiation, followed by green LED light on with continuous recording over 0.5 h.

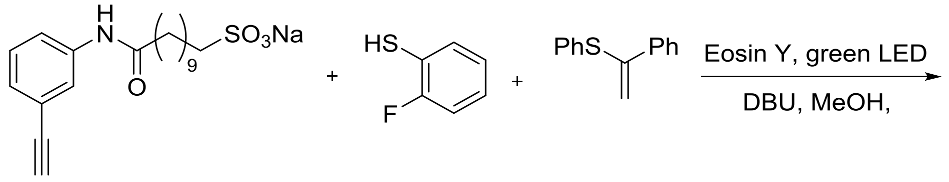

2g **1a** **3d**

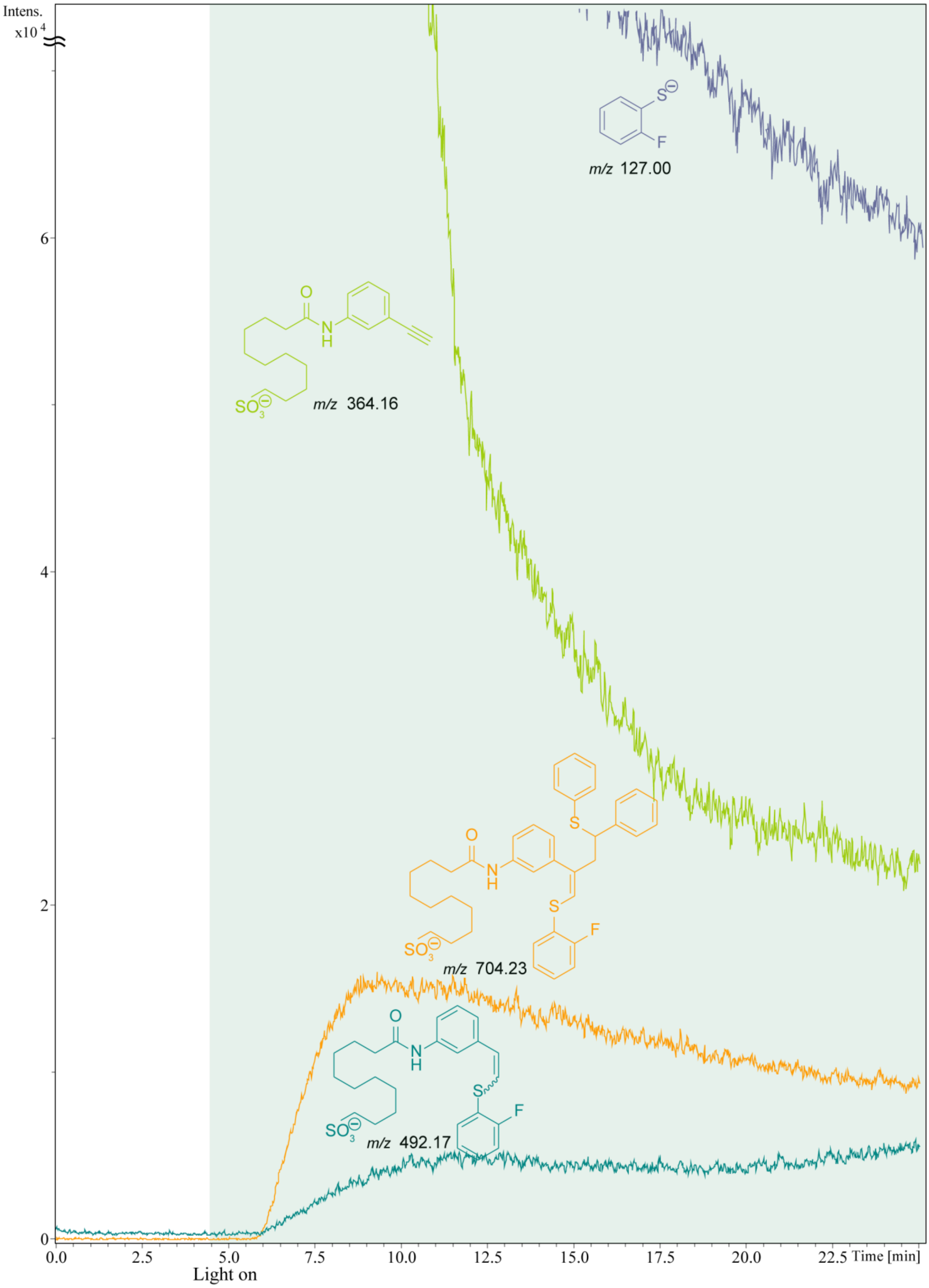

Figure S2. Real-time abundance of initial reagents (**1a**) (**2g**), product (**4agd**) with *m/z* 704.23 and byproduct (**β-5ag**) *m/z* 492.17 of photocatalytic reaction in the presence of eosin Y. Green light was turned on at 4.30 min from the beginning.

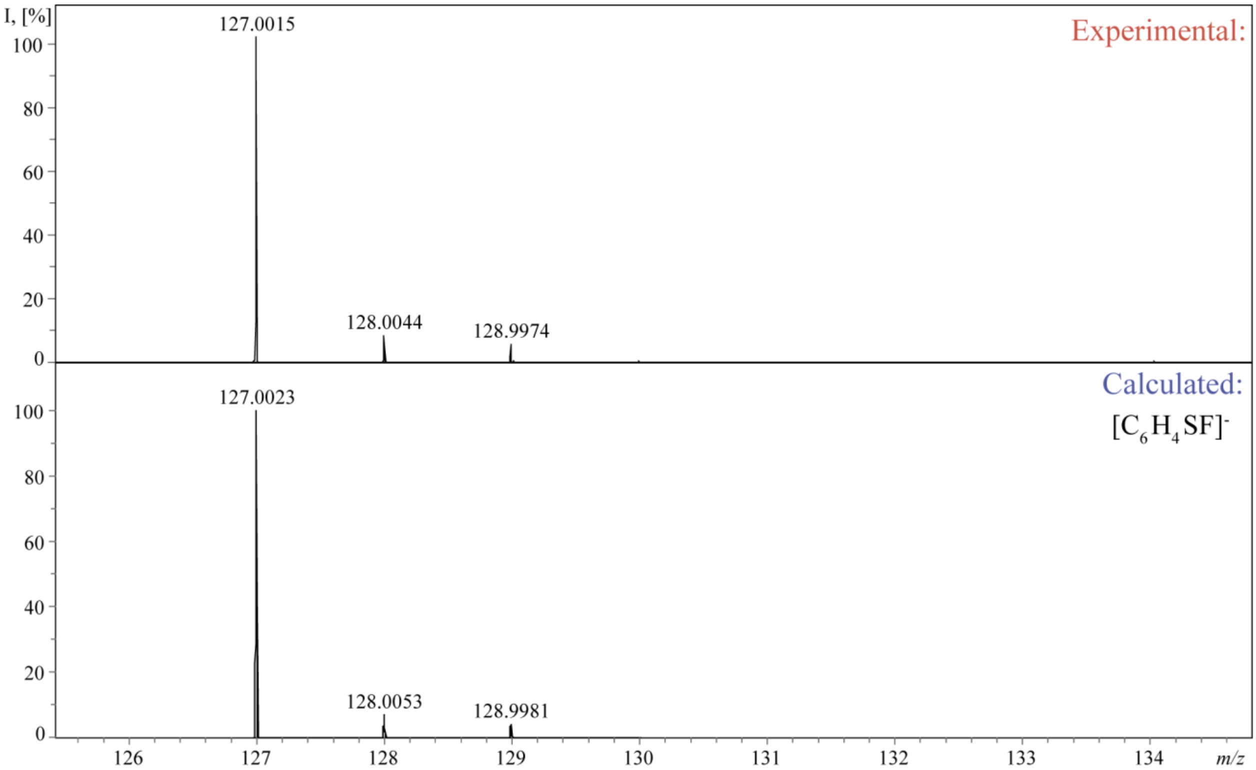

Figure S3. Experimentally detected and theoretical ESI-(-)MS spectrum of **1a** from the reaction mixture under online monitoring; main experimental peak [M-H]$^-$ = 127.0015 Da, calculated for C_6H_4SF = 127.0023 Da, Δ = 6.3 ppm.

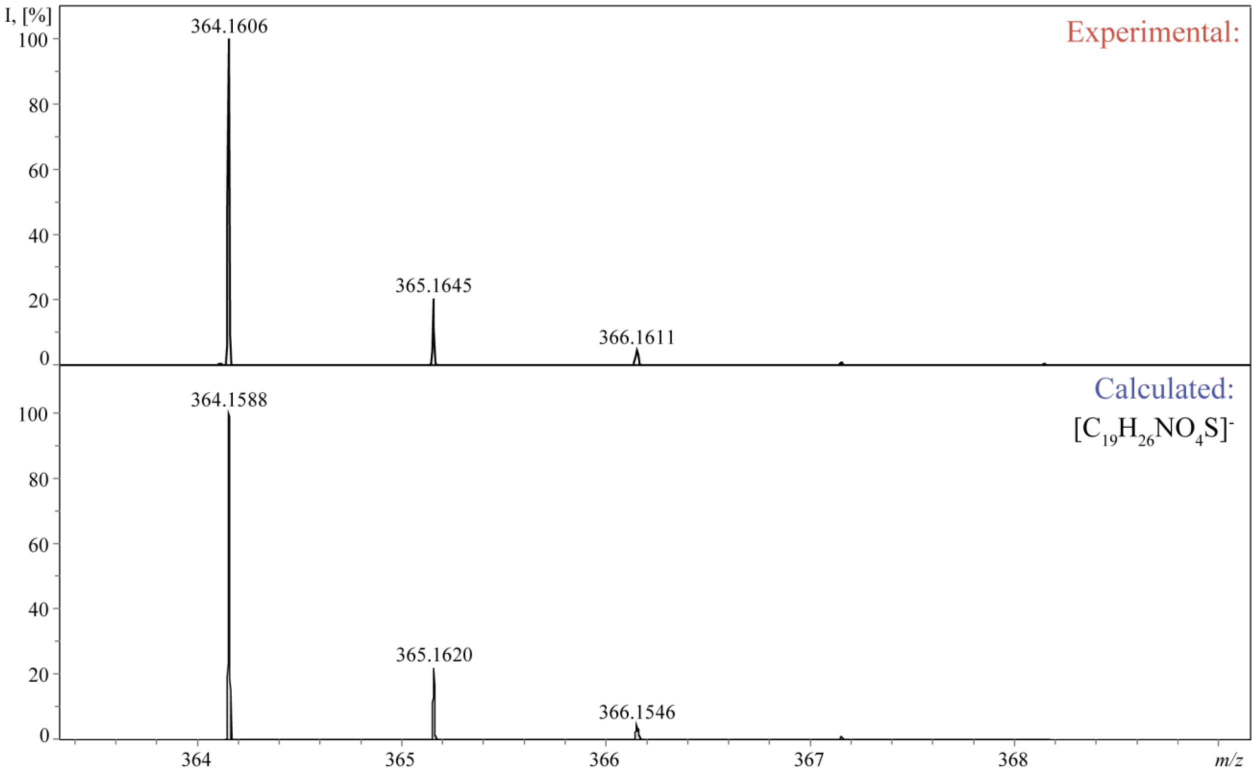

Figure S4. Experimentally detected and theoretical ESI-(-)MS spectrum of **2g** from the reaction mixture under online monitoring; main experimental peak [M]$^-$ = 364.1606 Da, calculated for $C_{19}H_{26}NO_4S$ = 364.1588 Da, Δ = 4.9 ppm.

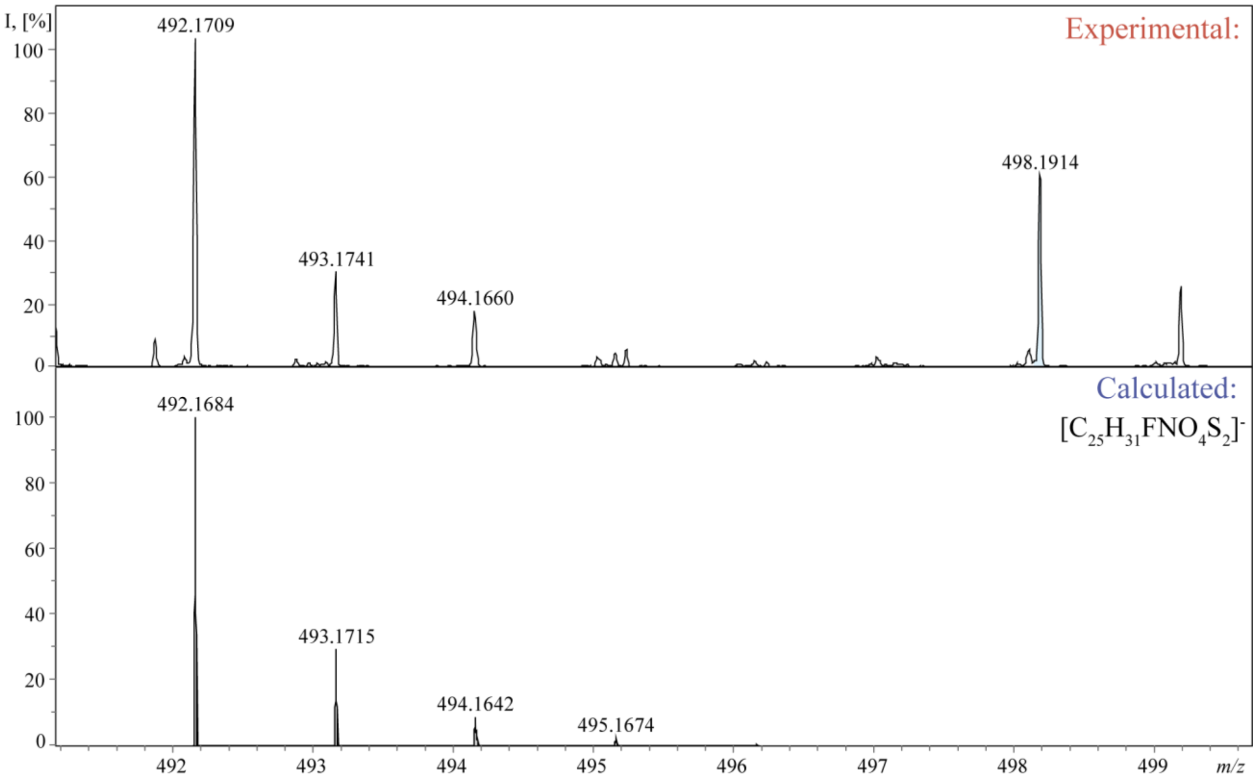

Figure S5. Experimentally detected and theoretical ESI-(-)MS spectrum of **β-5ag** from the reaction mixture under online monitoring; main experimental peak $[M]^-$ =492.1709 Da, calculated for $C_{25}H_{31}FNO_4S_2$ = 492.1684 Da, Δ = 5.0 ppm.

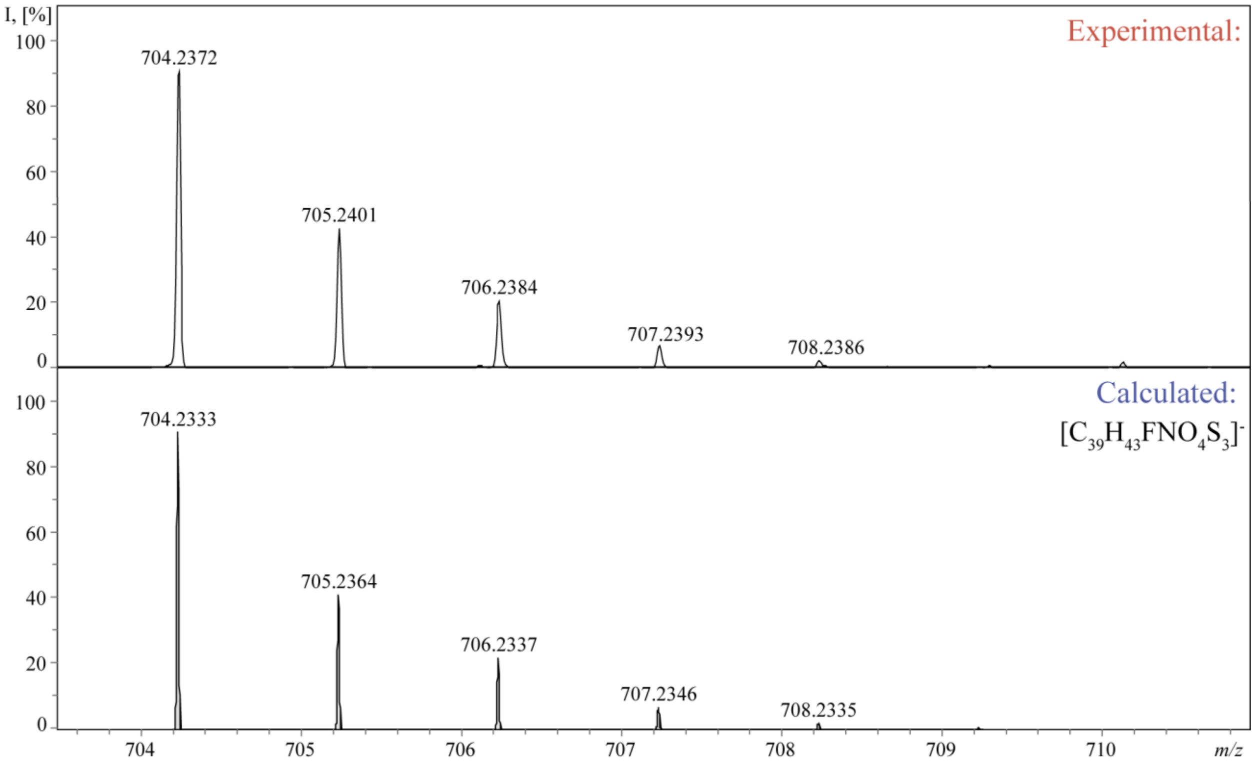

Figure S6. Experimentally detected and theoretical ESI-(-)MS spectrum of **4agd** from the reaction mixture under online monitoring; main experimental peak $[M]^-$ = 704.2372 Da, calculated for $C_{39}H_{43}FNO_4S_3$= 704.2344 Da, Δ = 4 ppm.

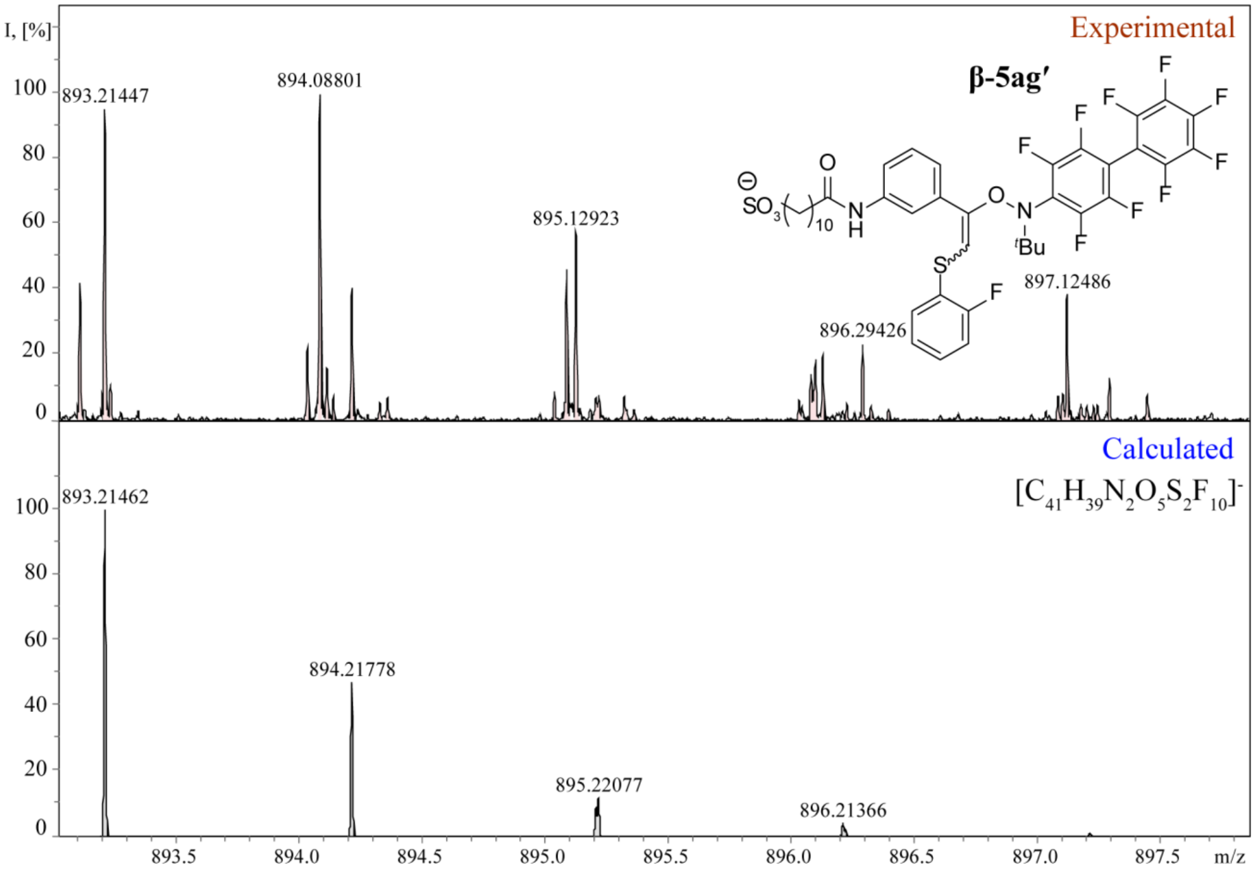

Figure S7. Experimentally detected and theoretical ESI-(-)MS spectrum of **β-5ag′** from the reaction mixture under light; main experimental peak $[M]^-$ = 893.21447 Da, calculated for $C_{41}H_{39}N_2O_5S_2F_{10}$ = 893.21462 Da, Δ = 0.2 ppm.

A white precipitate was isolated from the reaction mixture, purified by semipreparative TLC and analyzed by NMR spectroscopy and ESI-MS/MS mass spectrometry. Primary mass spectrometry analysis revealed the formation of a significant portion of the side products, including a major component ion with *m/z* 479.04344. To examine the structure of this molecule, tandem mass spectrometry (MS/MS) was applied (see **Figure S8** for detailed information). The value of choice for collision energy was 25 eV. Analysis of the MS2 spectrum signals corresponding to the loss of different fragments of the molecule allowed us to suppose 5-phenyl-2-(phenylthio)-3-(4-propylphenyl)furan formation. To confirm the structure of individual compounds, a number of 1D and 2D NMR experiments were performed (see **Figure S8 and Figures S23-S27** for detailed information).

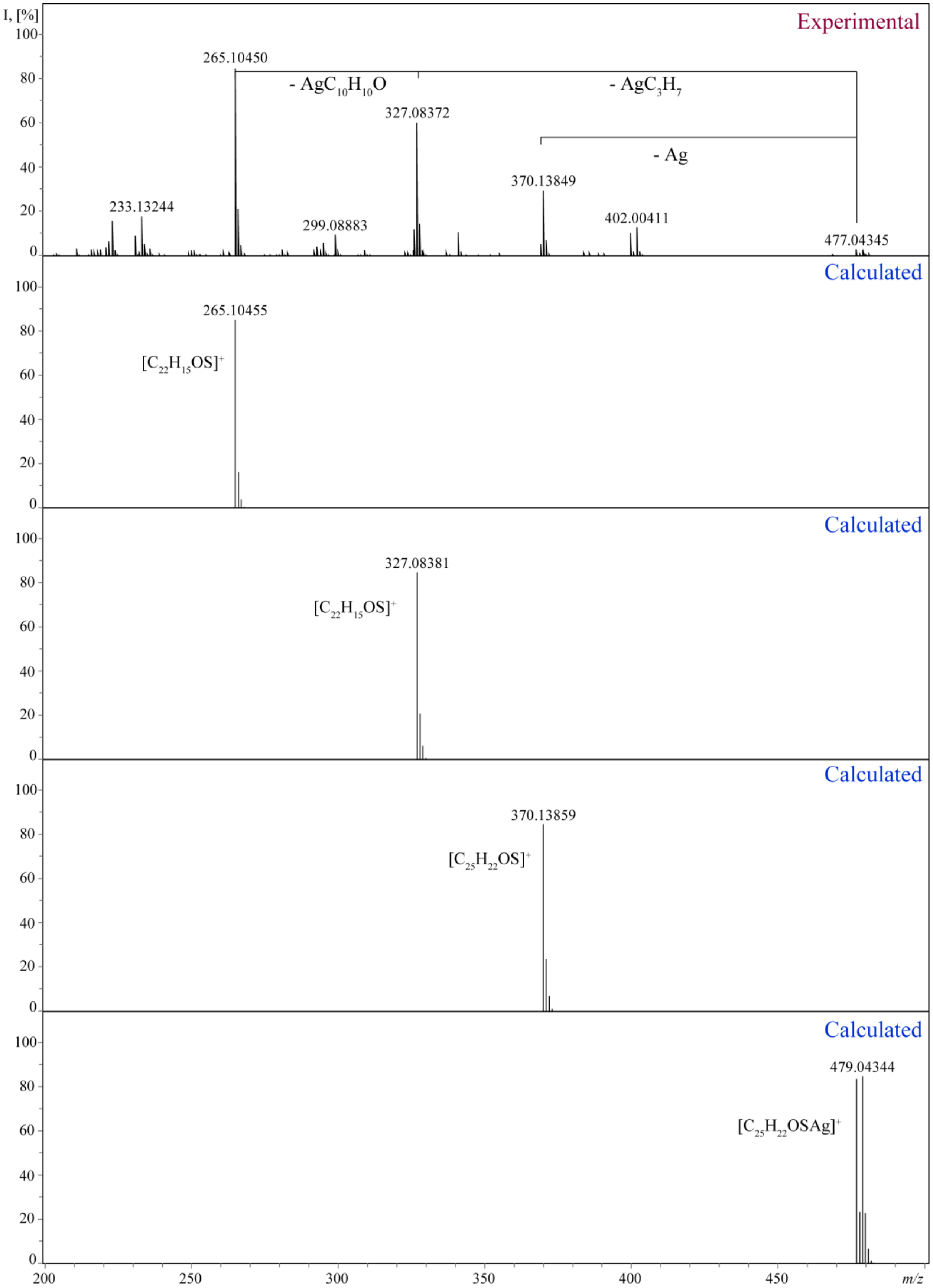

Figure S8. Experimentally detected ESI-(+)MS/MS (CID) spectrum of the side product 5-phenyl-2-(phenylthio)-3-(4-propylphenyl)furan; collision energy 25 eV. Calculated MS of parent and fragment ions.

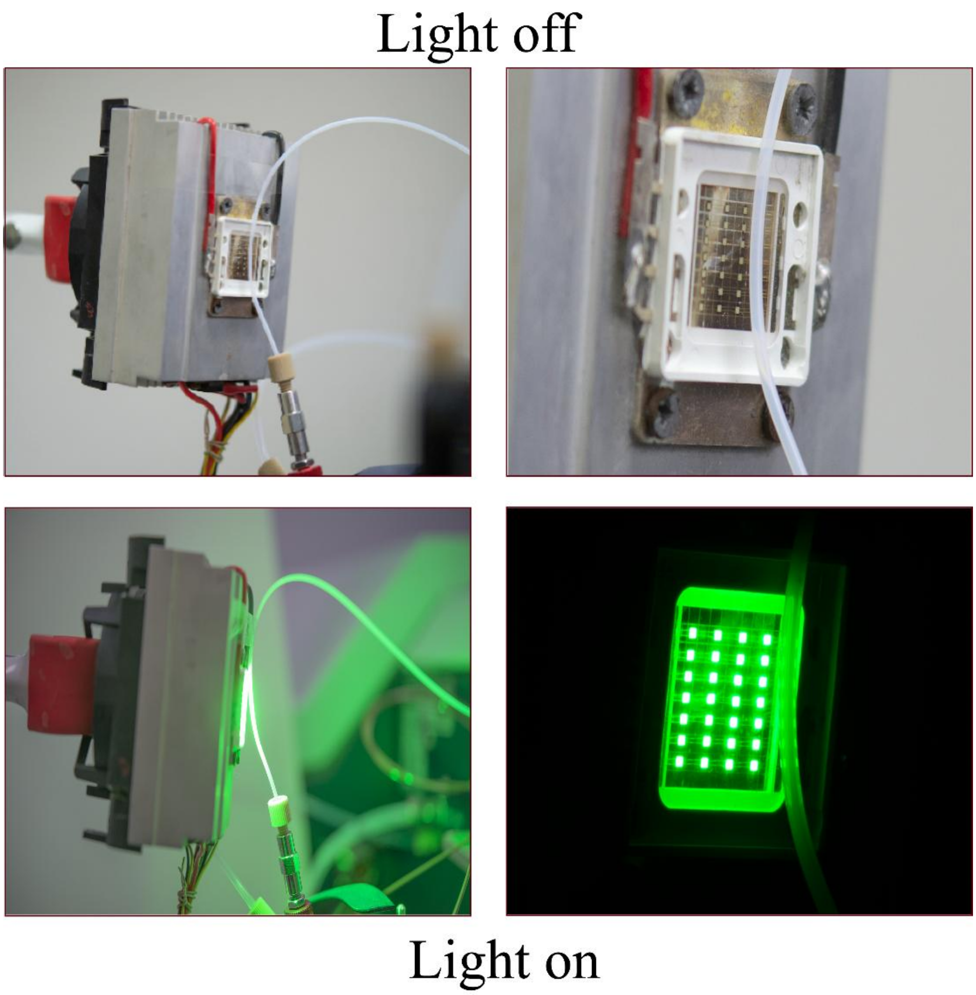

Figure S9. MS reaction setup for photocatalytic reaction investigation.

Design of the photoreactor

The created photoreactor consists of four 3D-printed syringe pumps, a programmed LCD display and a water bath connected to a thermostat (**Figure S10**). The developed concept allows up to 8 reactions to be carried out simultaneously, which is extremely important for accelerating the optimization process. Light irradiance was approximately measured in the reaction vessel as $730*10^3$ lux.

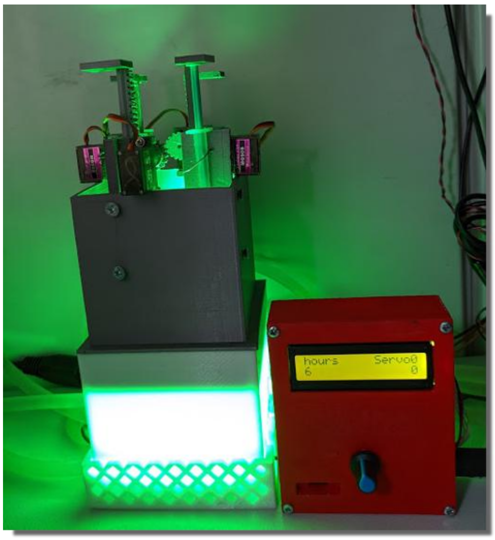

Figure S10. Custom build 3D-printed photoreactor.

EPR investigation

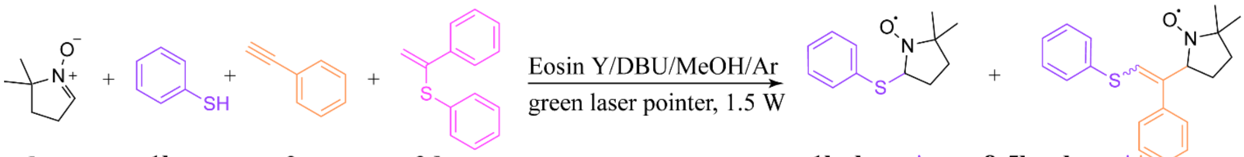

dmpo **1b** **2a** **3d** **1b-dmpo*** **β-5ba-dmpo****

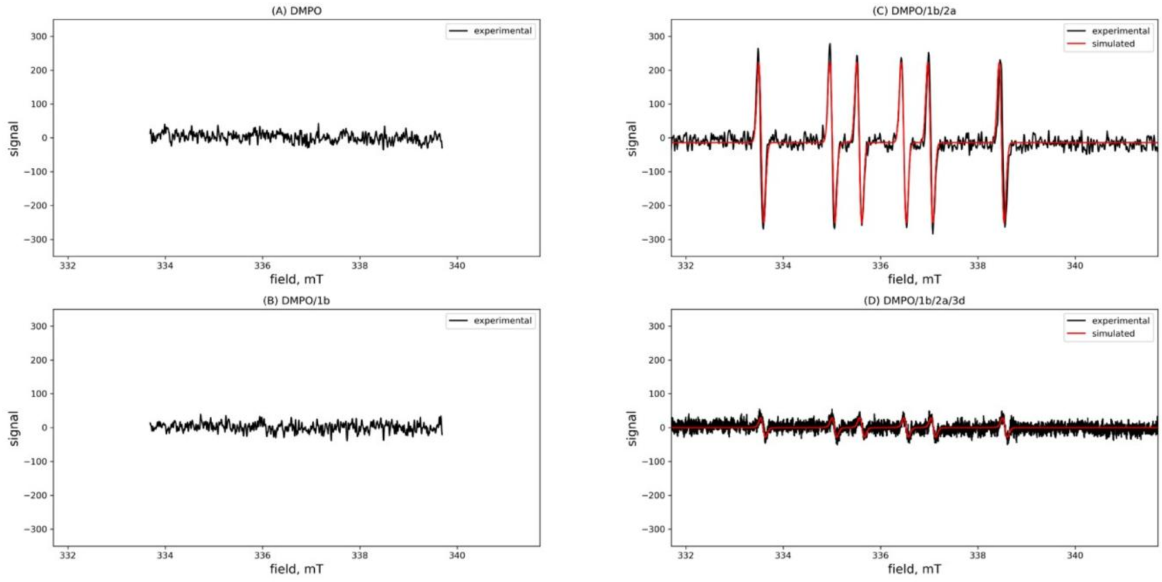

Figure S11. Experimental and simulated EPR spectra of mixtures without irradiation. The parameters of the simulation are listed in **Table S7**.

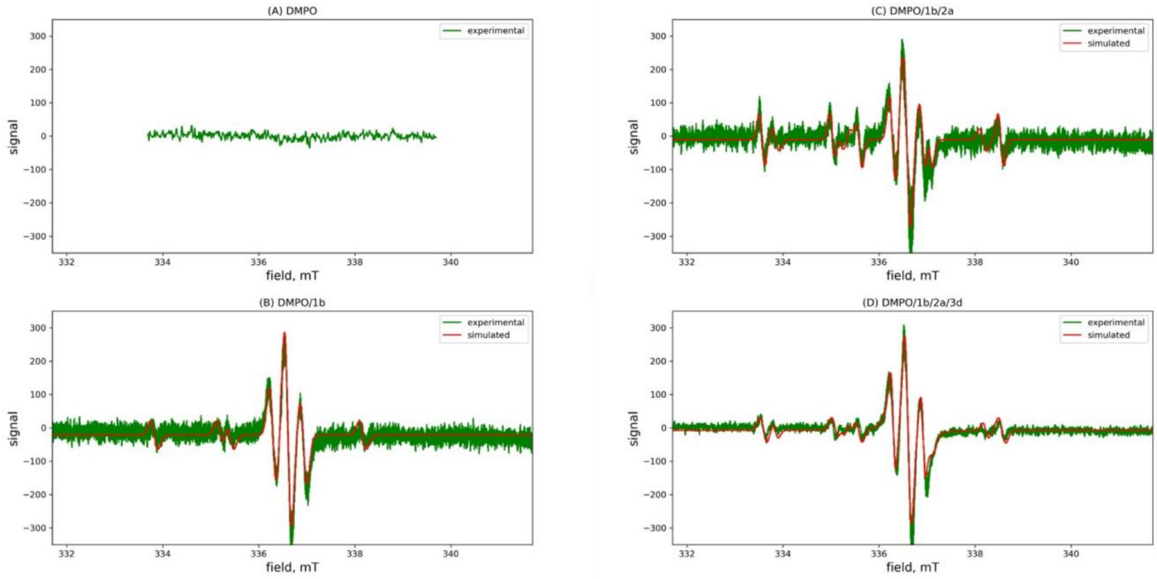

Figure S12. Experimental and simulated EPR spectra of mixtures under green irradiation. The parameters of the simulation are listed in **Table S8**.

Table S7. Fitted parameters for the simulation of EPR spectra for experiments without green irradiation.

Experiment	g-factor	A_H, MHz	A_H, mT	A_N, MHz	A_N, mT
C) β-5ba-dmpo	2.0060	56.0	2.00	40.8	1.46
D) β-5ba-dmpo	2.0058	56.7	2.02	40.9	1.46

Table S8. Fitted parameters for the simulation of EPR spectra for experiments under green irradiation. *Values of A_H.

Experiment	g-factor	A_H, MHz	A_H, mT	A_N, MHz	A_N, mT
B) Eosin $Y^{\cdot -}$	2.0025	9.2	0.32	8.6*	0.30*
B) 1b-dmpo	2.0061	44.0	1.57	38.5	1.37
C) Eosin $Y^{\cdot -}$	2.0026	9.3	0.33	8.3*	0.30*
C) 1b-dmpo	2.0061	45.1	1.61	37.7	1.36
C) β-5ba-dmpo	2.0058	56.5	2.02	41.1	1.47
D) Eosin $Y^{\cdot -}$	2.0027	9.6	0.34	7.8*	0.28*
D) 1b-dmpo	2.0060	43.5	1.55	39.3	1.40
D) β-5ba-dmpo	2.0057	55.0	1.96	41.7	1.49

Simulation parameters were obtained by fitting the garlic function and experimental data in the Easypin v5.2.33 program.

DFT calculations

Table S9. Energy parameters E(TS-Vb) − E(TS-Va) (ΔE) and E(TS-Vb-NMe_2) − E(TS-Va-NMe_2) (ΔE-NMe_2).

**level of theory**	**ΔE, kcal/mol**	**ΔE-NMe_2, kcal/mol**
PBE1PBE/6-311+G** gas phase	2.1	−0.3
PBE1PBE/6-311+G** PCM(MeOH)	1.9	0.3
B3LYP/6-311+G** gas phase	2.0	−0.4
B3LYP/6-311+G** PCM(MeOH)	2.6	1.0
M062X/6-311+G** gas phase	1.9	−1.4
M062X/6-311+G** PCM(MeOH)	1.4	−0.9

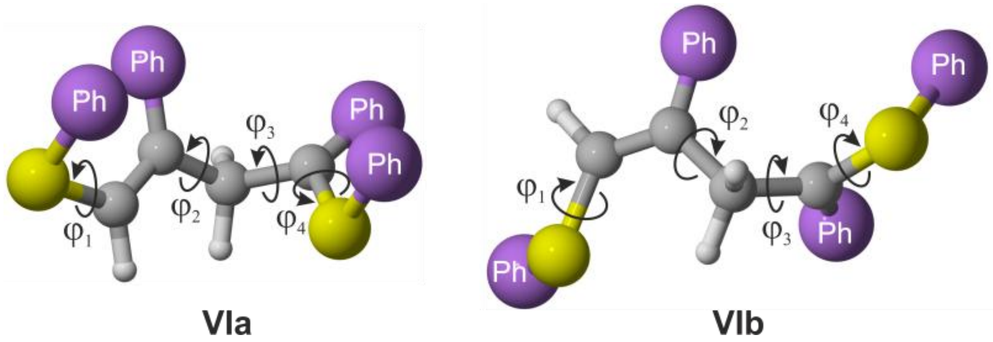

Figure S13. Conformational search for compounds **VIa** и **VIb**. The most stable conformations are shown.

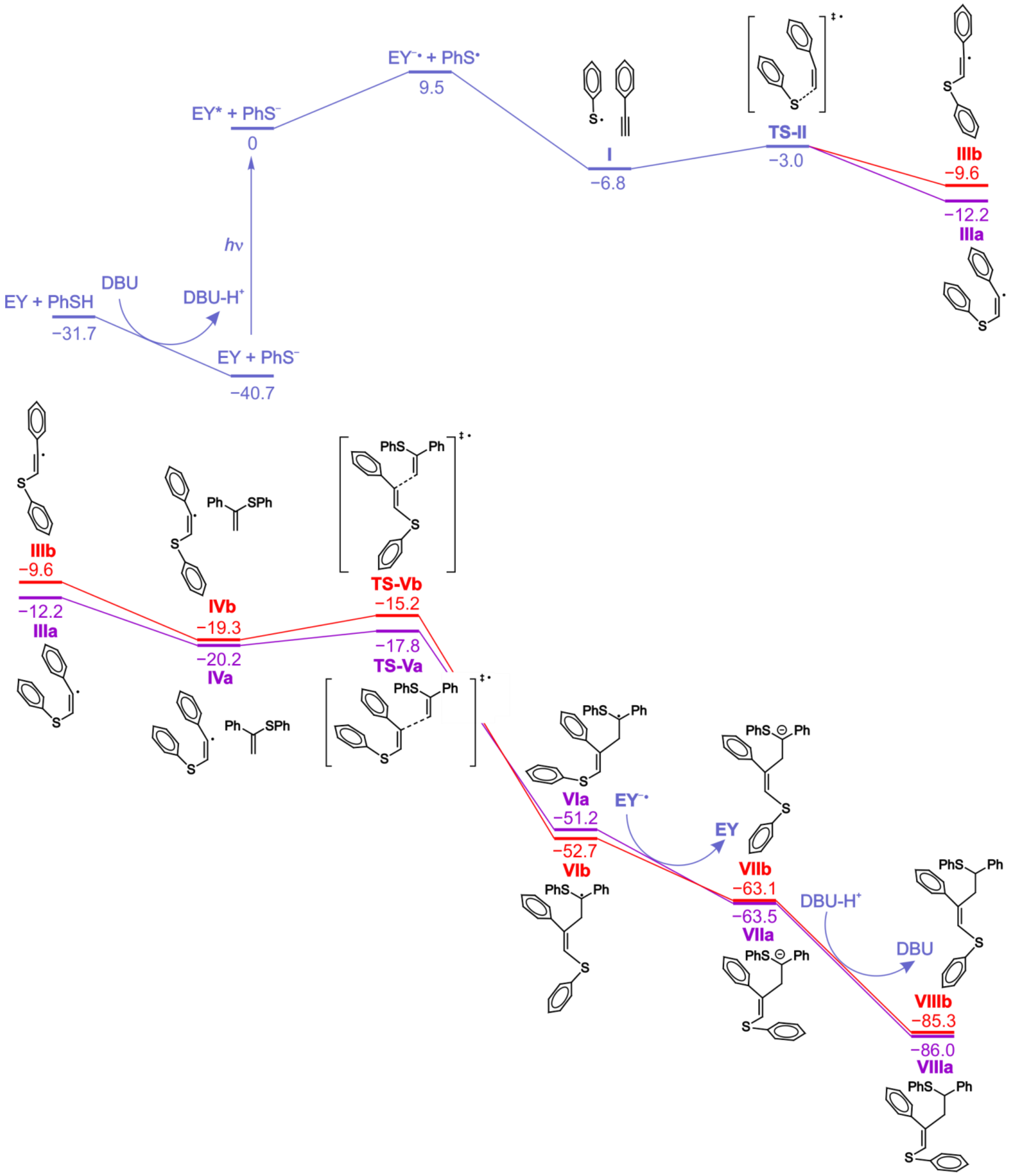

Figure S14. Energy profiles of three-component reactions between **1b**, **2a** and **3d**, leading to the Z-isomer (path a) and the E-isomer (path b) of product **VIII**. The values of potential energies ΔE are given in kcal/mol. B3LYP-D3BJ/6-311+G**&PCM(MeOH).

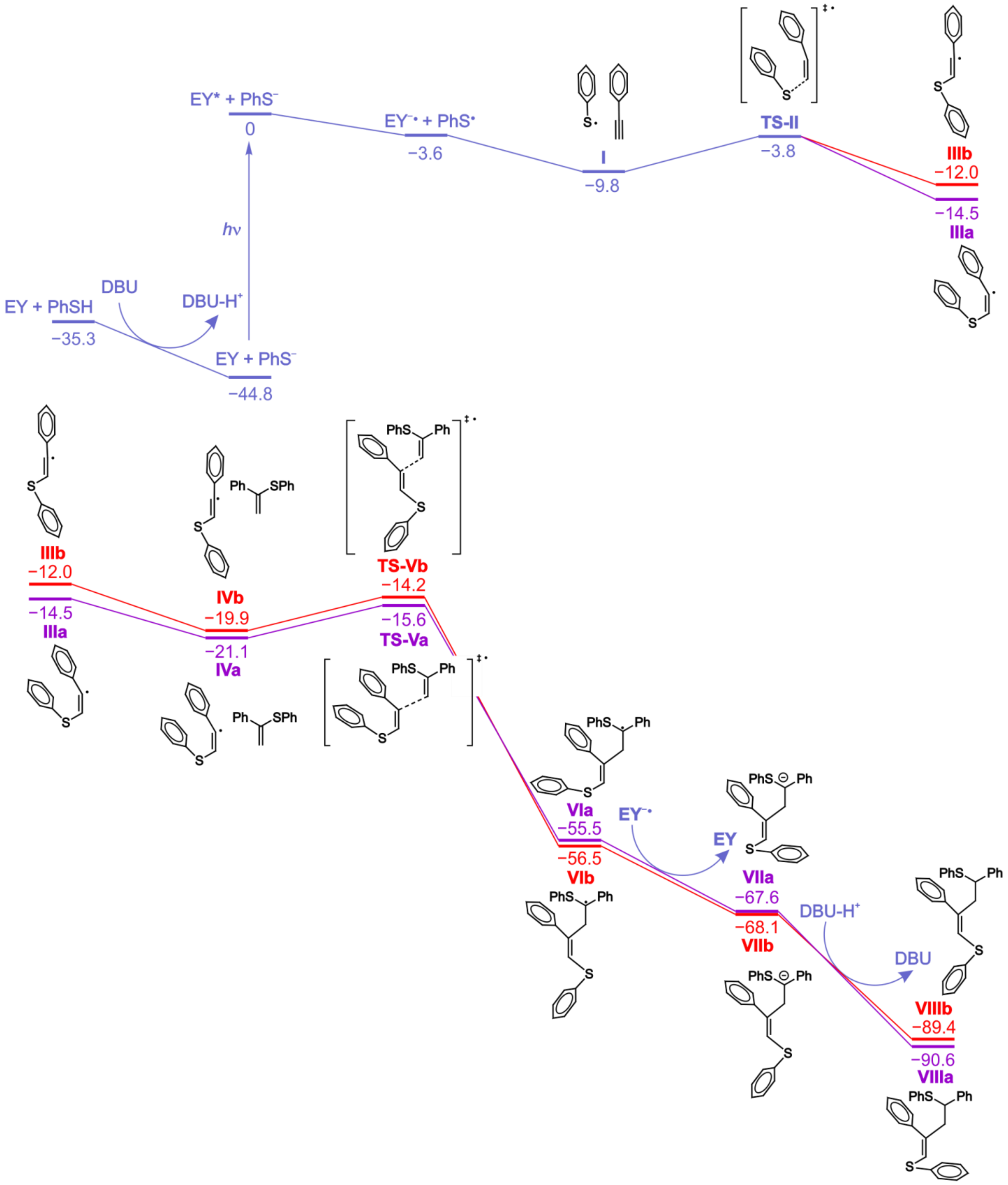

Figure S15. Energy profiles of three-component reactions between **1b**, **2a** and **3d**, leading to the Z-isomer (path a) and the E-isomer (path b) of product **VIII**. The values of potential energies ΔE are given in kcal/mol. M062X/6-311+G**&PCM(MeOH).

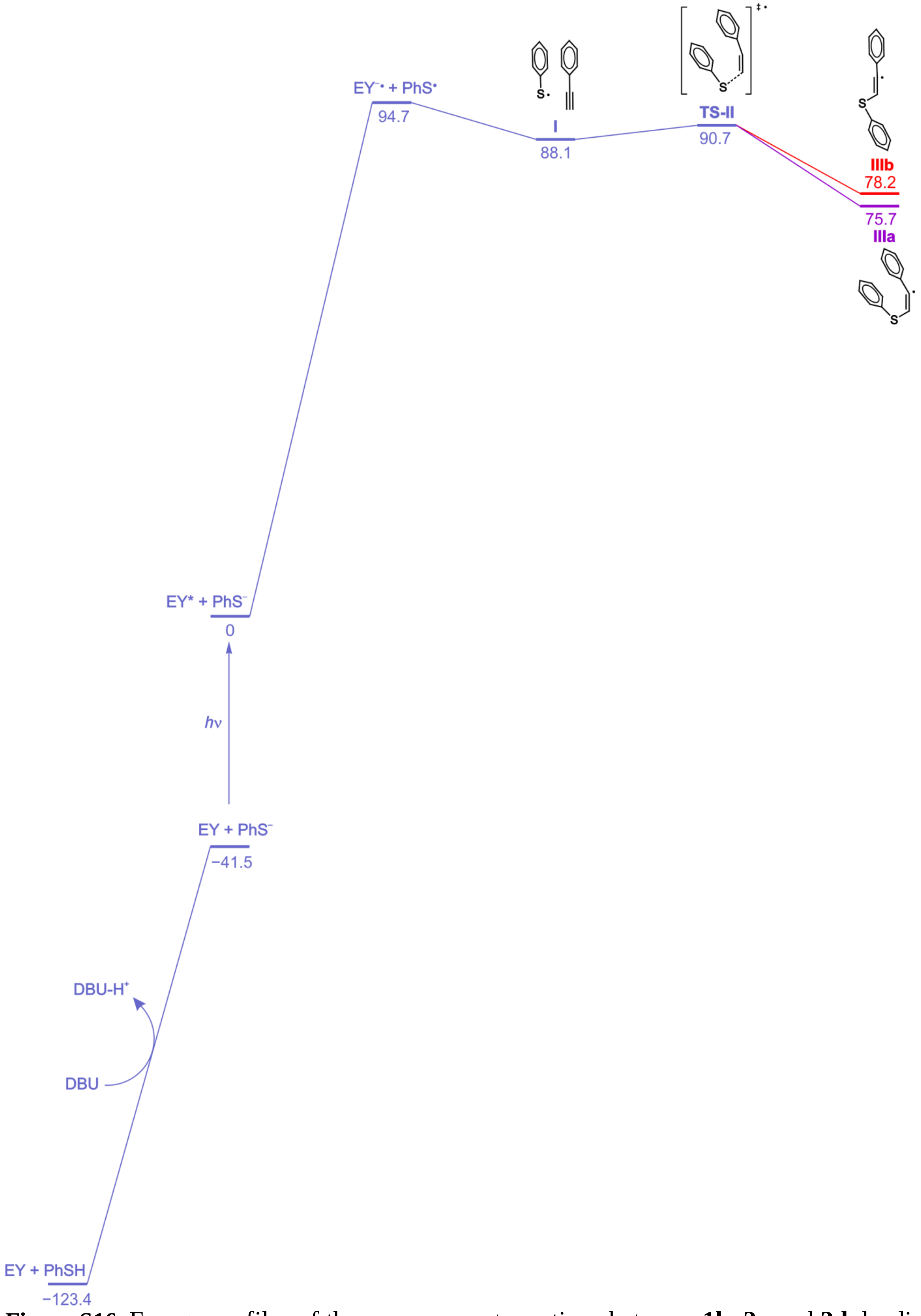

Figure S16. Energy profiles of three-component reactions between **1b**, **2a** and **3d**, leading to the Z-isomer (path a) and the E-isomer (path b) of product **VIII**. The values of potential energies ΔE are given in kcal/mol. PBE1PBE-D3BJ/6-311+G** gas phase. Part 1.

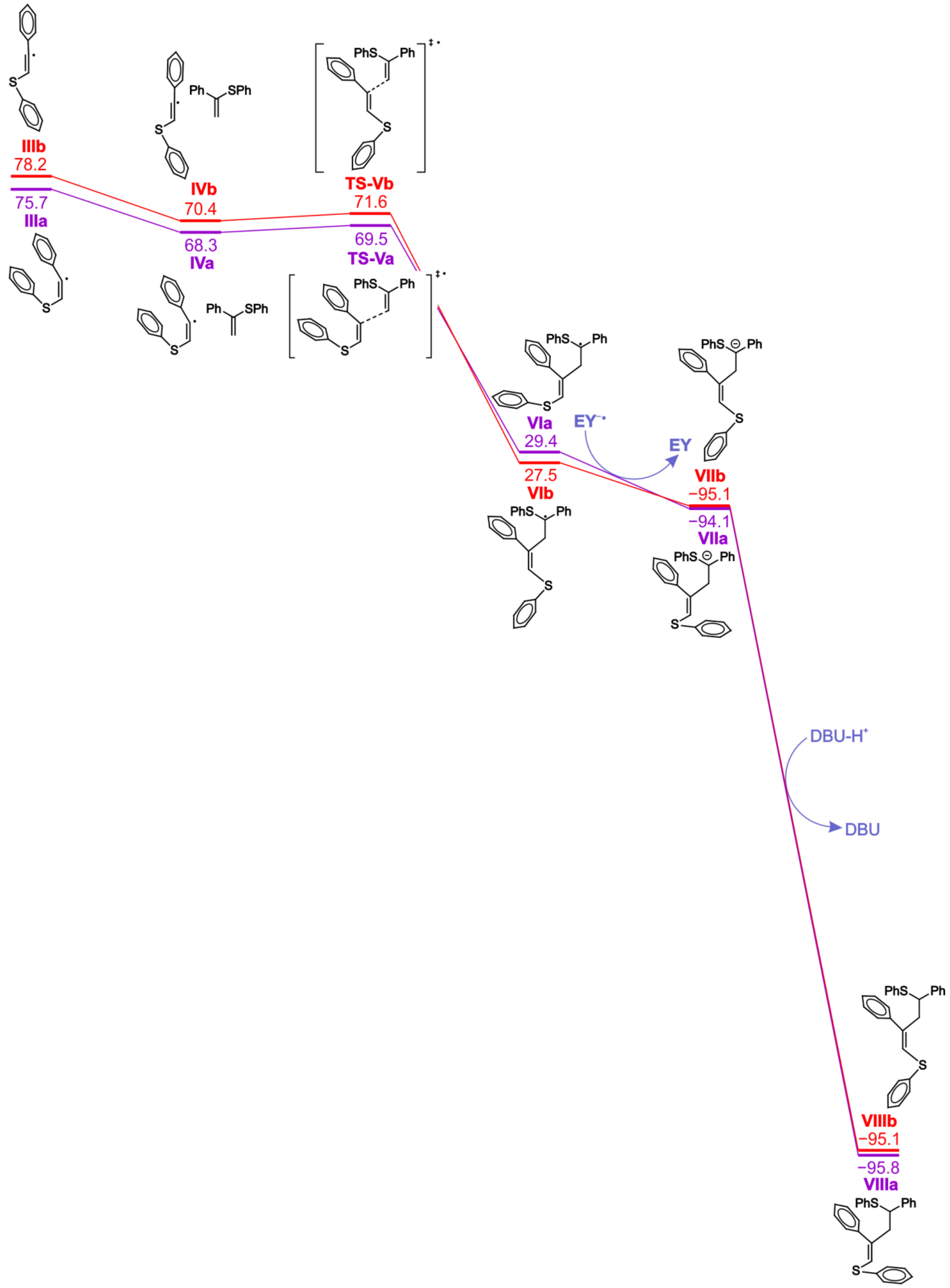

Figure S17. Energy profiles of three-component reactions between **1b**, **2a** and **3d**, leading to the Z-isomer (path a) and the E-isomer (path b) of product **VIII**. The values of potential energies ΔE are given in kcal/mol. PBE1PBE-D3BJ/6-311+G** gas phase. Part 2.

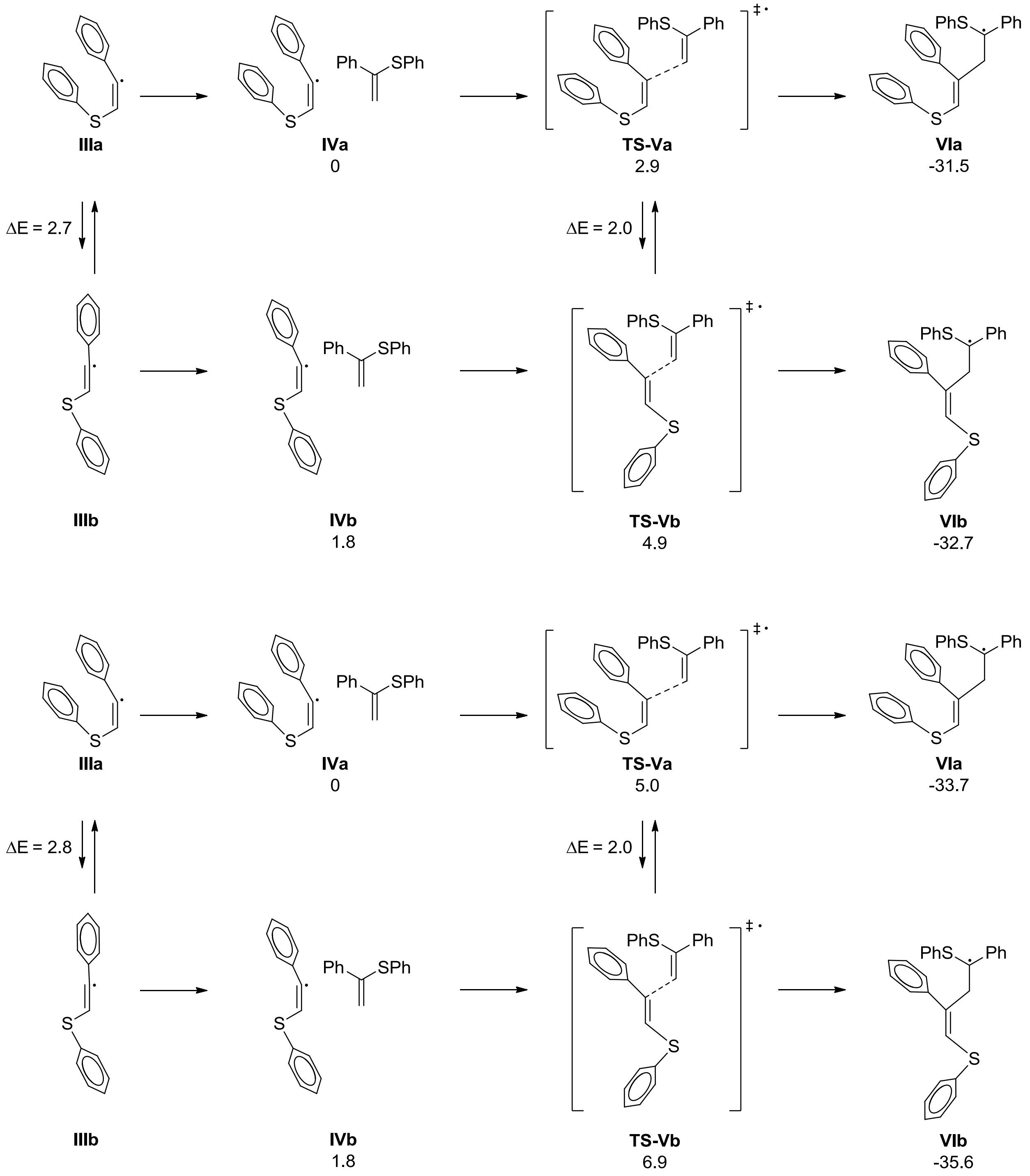

Figure S18. Top: stage of carboradical addition **III** → **VI**. B3LYP-D3BJ/6-311+G** gas phase; bottom: stage of carboradical addition **III** → **VI**. M062X/6-311+G** gas phase.

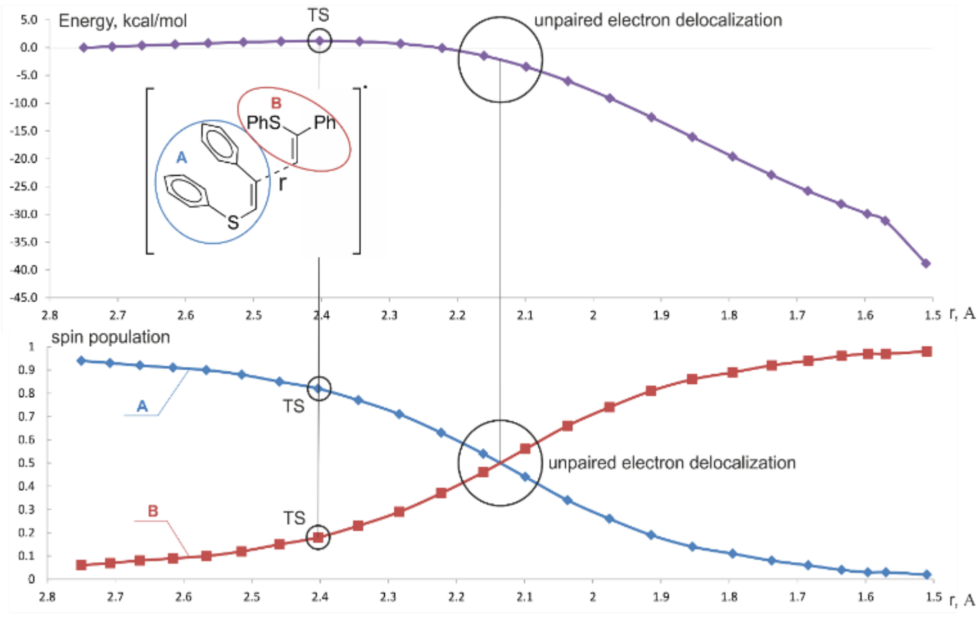

Figure S19. Energy profile of the **IVa** → **VIa** (top) stage; transfer of an unpaired electron during the formation of a C-C bond (below). The calculation was carried out at the PBE1PBE-D3BJ/6-311+G** level of theory.

Cyclic voltammetry of selected components

The PhS^- anion has a smaller oxidation potential in comparison to its acidic form PhSH, which has no peaks on CV in the range of solvent stability (**Figure S20**). The exact value of $E^o_{PhS^{\cdot}/PhS^-}$ can be estimated in a quasi-static process with a small scan rate. $E^o_{PhS^{\cdot}/PhS^-}$ = -93 mV vs Fc^+/Fc or $E^o_{PhS^{\cdot}/PhS^-}$ = 0.281 V vs SCE.[14]

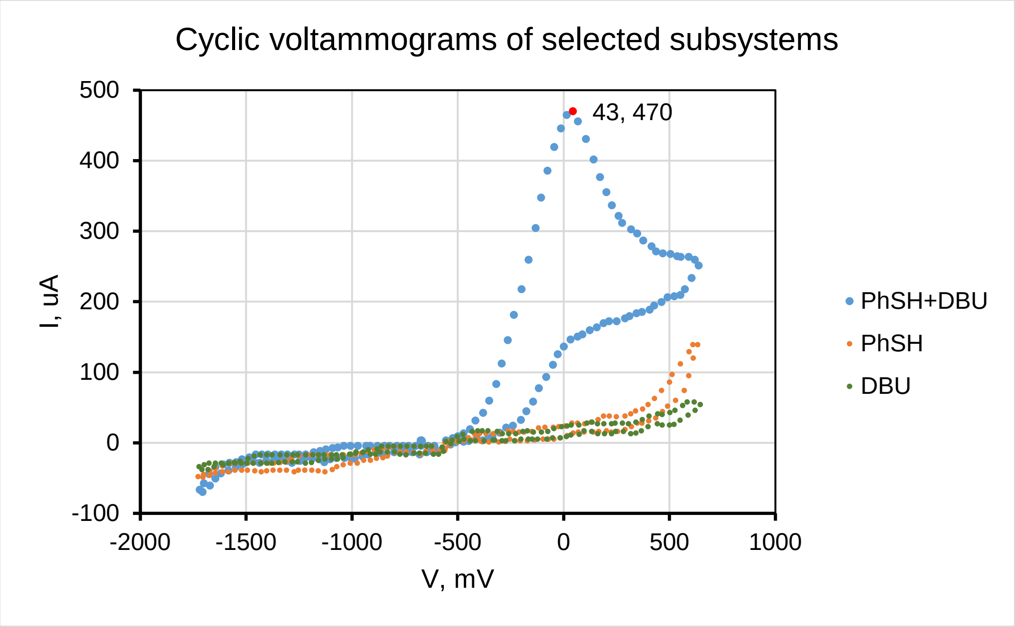

Figure S20. Cyclic voltammograms of thiophenol + DBU (15 mM and 22.5 mM) blue line, thiophenol (15 mM) – orange line and DBU (22.5 mM) – green line vs Fc^+/Fc; scan rate 300 mV/s.

Stern-Volmer measurements

A Spectrofluorometer Cary Eclipse (Agilent) was used for all measurements. Corrected emission spectra were recorded with the same excitation and emission slit sizes of 5 and 5 nm, respectively, and an integration time of 0.1 seconds was used for all quenching measurements.

Stock solutions were prepared as follows. Eosin Y (0.7 mg) was weighed into a 5 ml volumetric flask and dissolved in MeOH (**SOL0**) to obtain the photocatalyst solution.250 µl of **SOL0** was added to a 250 ml volumetric flask and diluted in MeOH. Thus, the final Eosin Y solution (**SOL1**) had a concentration equal to $2*10^{-7}$ M. Thiophenol (**1b**) 55 mg was weighed into a 5 ml volumetric flask and dissolved in **SOL1** for the thiol solution (0.1 M). Phenylacetylene (**2a**) (51 mg) was weighed into a 5 ml volumetric flask and dissolved in **SOL1** for the alkyne solution (0.1 M). DBU (76 mg) was weighed into a 5 ml volumetric flask and dissolved in **SOL1** for the DBU solution (0.1 M). In the case of an alkyne (0.1 M) + DBU (0.1 M) solution 51 mg of phenylacetylene (**2a**) and 76 mg of DBU were weighed into a 5 ml volumetric flask and dissolved in **SOL1**. For the thiol (0.1 M) + DBU (0.15 M) solution, 51 mg of phenylacetylene and 114 mg of DBU were weighed into a 5 ml volumetric flask and dissolved in **SOL1**. Phenyl(1-phenylvinyl)sulfane (212 mg) was weighed into a 5 ml volumetric flask and dissolved in **SOL1** for the preparation of alkene (**3d**) solution (0.2 M). Diphenyldisulphide (109 mg) was weighed into a 5 ml volumetric flask and dissolved in **SOL1** for the preparation of a disulphide solution (0.1 M).

Zero point measurements of the photocatalyst fluorescence intensity were performed 10 times for **SOL1** and averaged. Then, in the cuvette, corresponding amounts (**Table S10**) of **SOL1** and quencher solution were mixed, and the fluorescence intensity was immediately measured.

Table S10. Amounts of the stock solutions mixed in the cuvette for steady state Stern-Volmer measurements

Thiol		Aklyne		DBU		Alkyne + DBU		Thiol + DBU		Alkene		Disulphide	
V(Eosin)	V(sol)	V(Eosin)	V(sol)	V(Eosin)	V(sol)	V(Eosin)	V(sol)	V(Eosin)	V(sol)	V(Eosin)	V(sol)	V(Eosin)	V(sol)
3000	10	3000	50	3000	50	3000	100	3000	50	3000	200	3000	200
3000	50	3000	100	3000	100	3000	300	3000	100	3000	300	3000	400
3000	100	3000	200	3000	200	3000	500	3000	150	3000	400	2500	500
3000	200	3000	400	3000	500	3000	1000	3000	200	2500	500	2500	1000
3000	400	2000	1000	2000	1000			3000	300	2500	1000		
3000	600	2000	1500	2000	1500			3000	400				
3000	800							2500	500				
3000	1000							2500	1000				
3000	1200												

In all measurements, λ_{ex} = 529 nm. The intensity values were taken at λ_{em}=541 nm for steady state Stern-Volmer plotting (**Figure S21**). A quenching factor $I_0/I-1$ was applied.

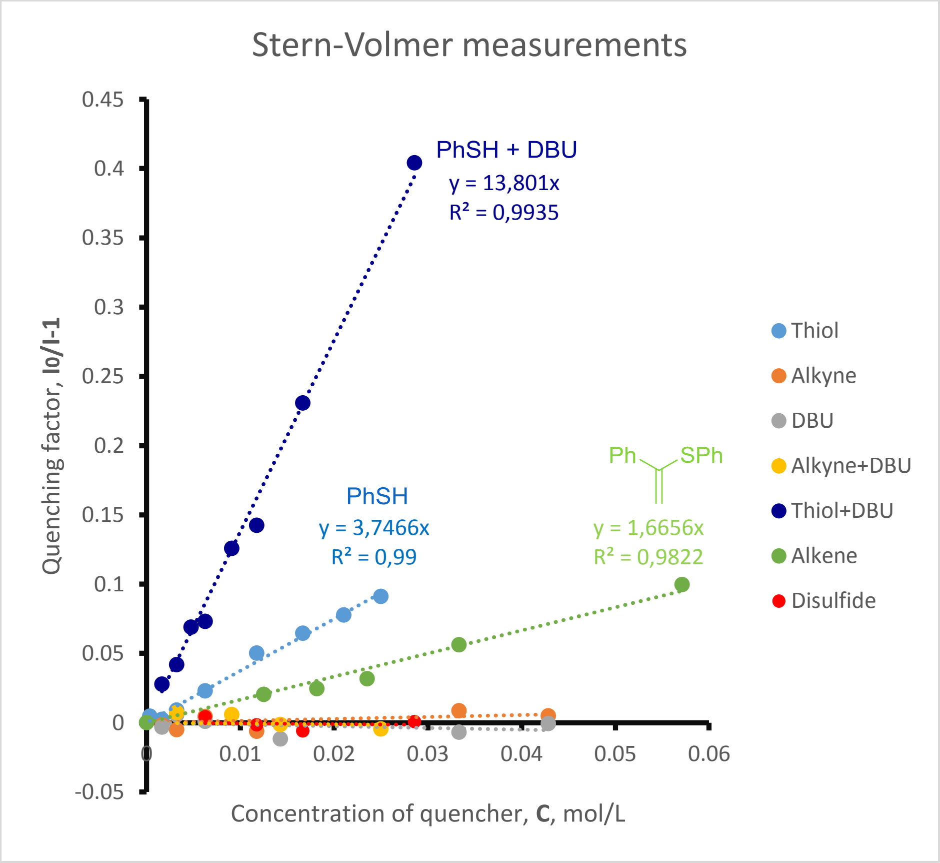

Figure S21. Steady state Stern-Volmer measurements of the photocatalyst fluorescence quenching.

X-ray Crystal Structure Determination of 4bad

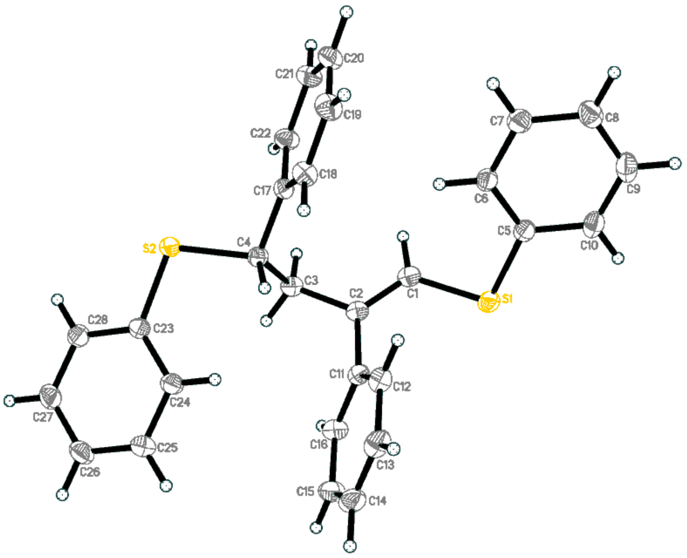

Figure S22. Crystal structure of **4bad**.

Table S 11. Crystal data and structure refinement for **4bad**.

Identification code	**4bad**	
CCDC number	2093791	
Empirical formula	C28 H24 S2	
Formula weight	424.59	
Temperature	100(2) K	
Wavelength	0.71073 Å	
Crystal system	Triclinic	
Space group	P-1	
Unit cell dimensions	a = 6.6567(2) Å	α= 86.4610(10)°.
	b = 10.7436(3) Å	β= 87.5340(10)°.
	c = 15.6159(5) Å	γ = 89.6150(10)°.
Volume	1113.63(6) Å^3	
Z	2	
Density (calculated)	1.266 g/cm^3	
Absorption coefficient	0.252 mm^{-1}	
F(000)	448	
Crystal size	0.60 x 0.47 x 0.38 mm^3	
Theta range for data collection	2.239 to 32.498°.	
Index ranges	-10<=h<=10, -16<=k<=16, -23<=l<=23	
Reflections collected	51448	
Independent reflections	8033 [R(int) = 0.0301]	

Observed reflections	7076	
Completeness to theta = 25.242°	99.7 %	
Absorption correction	Semi-empirical from equivalents	
Max. and min. transmission	0.7471 and 0.7067	
Refinement method	Full-matrix least-squares on F^2	
Data / restraints / parameters	8033 / 0 / 271	
Goodness-of-fit on F^2	1.067	
Final R indices [I>2sigma(I)]	R1 = 0.0387, wR2 = 0.0946	
R indices (all data)	R1 = 0.0456, wR2 = 0.0996	
Largest diff. peak and hole	0.437 and -0.437 e.Å^{-3}	

Table S12. Atomic coordinates (x 10^4) and equivalent isotropic displacement parameters (Å^2 x 10^3) for **4bad**. U(eq) is defined as one-third of the trace of the orthogonalized U^{ij} tensor.

Atom	x	y	z	U(eq)
S(1)	9572(1)	4803(1)	1350(1)	23(1)
S(2)	1989(1)	5289(1)	4263(1)	21(1)
C(1)	8264(2)	4905(1)	2348(1)	21(1)
C(2)	6730(2)	5689(1)	2483(1)	19(1)
C(3)	5697(2)	5728(1)	3365(1)	20(1)
C(4)	3746(2)	4954(1)	3376(1)	18(1)
C(5)	9640(2)	3171(1)	1259(1)	20(1)
C(6)	8322(2)	2354(1)	1726(1)	21(1)
C(7)	8476(2)	1078(1)	1625(1)	25(1)
C(8)	9912(2)	609(1)	1058(1)	28(1)
C(9)	11211(2)	1428(1)	590(1)	29(1)
C(10)	11089(2)	2700(1)	687(1)	25(1)
C(11)	5806(2)	6480(1)	1787(1)	19(1)
C(12)	4851(2)	5940(1)	1123(1)	22(1)
C(13)	3866(2)	6675(1)	509(1)	27(1)
C(14)	3823(2)	7962(1)	548(1)	30(1)
C(15)	4766(2)	8514(1)	1202(1)	29(1)
C(16)	5752(2)	7781(1)	1822(1)	24(1)
C(17)	4093(2)	3555(1)	3447(1)	18(1)
C(18)	3250(2)	2820(1)	2854(1)	22(1)
C(19)	3466(2)	1528(1)	2913(1)	27(1)
C(20)	4556(2)	958(1)	3566(1)	28(1)
C(21)	5417(2)	1679(1)	4158(1)	27(1)
C(22)	5183(2)	2972(1)	4104(1)	24(1)
C(23)	1128(2)	6822(1)	3998(1)	20(1)

C(24)	1296(2)	7406(1)	3174(1)	22(1)
C(25)	533(2)	8604(1)	3025(1)	26(1)
C(26)	-439(2)	9221(1)	3680(1)	28(1)
C(27)	-636(2)	8631(1)	4496(1)	28(1)
C(28)	160(2)	7448(1)	4660(1)	24(1)

Table S13. Bond lengths [Å] and angles [°] for **4bad**.

S(1)-C(1)	1.7604(12)
S(1)-C(5)	1.7675(12)
S(2)-C(23)	1.7704(11)
S(2)-C(4)	1.8260(11)
C(1)-C(2)	1.3370(15)
C(1)-H(1)	0.9500
C(2)-C(11)	1.4900(14)
C(2)-C(3)	1.5149(15)
C(3)-C(4)	1.5462(15)
C(3)-H(3A)	0.9900
C(3)-H(3B)	0.9900
C(4)-C(17)	1.5174(14)
C(4)-H(4)	1.0000
C(5)-C(6)	1.3953(15)
C(5)-C(10)	1.4002(15)
C(6)-C(7)	1.3921(16)
C(6)-H(6)	0.9500
C(7)-C(8)	1.3888(17)
C(7)-H(7)	0.9500
C(8)-C(9)	1.3903(19)
C(8)-H(8)	0.9500
C(9)-C(10)	1.3852(19)
C(9)-H(9)	0.9500
C(10)-H(10)	0.9500
C(11)-C(12)	1.3962(15)
C(11)-C(16)	1.4025(16)
C(12)-C(13)	1.3876(16)
C(12)-H(12)	0.9500
C(13)-C(14)	1.388(2)
C(13)-H(13)	0.9500
C(14)-C(15)	1.3871(19)
C(14)-H(14)	0.9500

C(15)-C(16)	1.3941(16)
C(15)-H(15)	0.9500
C(16)-H(16)	0.9500
C(17)-C(18)	1.3907(15)
C(17)-C(22)	1.3960(15)
C(18)-C(19)	1.3922(16)
C(18)-H(18)	0.9500
C(19)-C(20)	1.3868(19)
C(19)-H(19)	0.9500
C(20)-C(21)	1.3865(18)
C(20)-H(20)	0.9500
C(21)-C(22)	1.3956(16)
C(21)-H(21)	0.9500
C(22)-H(22)	0.9500
C(23)-C(24)	1.3952(15)
C(23)-C(28)	1.4006(15)
C(24)-C(25)	1.3911(15)
C(24)-H(24)	0.9500
C(25)-C(26)	1.3871(17)
C(25)-H(25)	0.9500
C(26)-C(27)	1.3880(19)
C(26)-H(26)	0.9500
C(27)-C(28)	1.3876(16)
C(27)-H(27)	0.9500
C(28)-H(28)	0.9500

C(1)-S(1)-C(5)	101.19(5)
C(23)-S(2)-C(4)	104.19(5)
C(2)-C(1)-S(1)	123.87(9)
C(2)-C(1)-H(1)	118.1
S(1)-C(1)-H(1)	118.1
C(1)-C(2)-C(11)	123.52(10)
C(1)-C(2)-C(3)	120.56(10)
C(11)-C(2)-C(3)	115.68(9)
C(2)-C(3)-C(4)	108.35(8)
C(2)-C(3)-H(3A)	110.0
C(4)-C(3)-H(3A)	110.0
C(2)-C(3)-H(3B)	110.0
C(4)-C(3)-H(3B)	110.0
H(3A)-C(3)-H(3B)	108.4

C(17)-C(4)-C(3)	113.89(9)
C(17)-C(4)-S(2)	106.01(7)
C(3)-C(4)-S(2)	112.89(7)
C(17)-C(4)-H(4)	107.9
C(3)-C(4)-H(4)	107.9
S(2)-C(4)-H(4)	107.9
C(6)-C(5)-C(10)	119.62(11)
C(6)-C(5)-S(1)	122.93(8)
C(10)-C(5)-S(1)	117.45(9)
C(7)-C(6)-C(5)	119.70(10)
C(7)-C(6)-H(6)	120.2
C(5)-C(6)-H(6)	120.2
C(8)-C(7)-C(6)	120.75(11)
C(8)-C(7)-H(7)	119.6
C(6)-C(7)-H(7)	119.6
C(7)-C(8)-C(9)	119.31(12)
C(7)-C(8)-H(8)	120.3
C(9)-C(8)-H(8)	120.3
C(10)-C(9)-C(8)	120.68(11)
C(10)-C(9)-H(9)	119.7
C(8)-C(9)-H(9)	119.7
C(9)-C(10)-C(5)	119.94(11)
C(9)-C(10)-H(10)	120.0
C(5)-C(10)-H(10)	120.0
C(12)-C(11)-C(16)	118.74(10)
C(12)-C(11)-C(2)	120.84(10)
C(16)-C(11)-C(2)	120.25(10)
C(13)-C(12)-C(11)	120.77(11)
C(13)-C(12)-H(12)	119.6
C(11)-C(12)-H(12)	119.6
C(12)-C(13)-C(14)	120.16(11)
C(12)-C(13)-H(13)	119.9
C(14)-C(13)-H(13)	119.9
C(15)-C(14)-C(13)	119.84(11)
C(15)-C(14)-H(14)	120.1
C(13)-C(14)-H(14)	120.1
C(14)-C(15)-C(16)	120.28(12)
C(14)-C(15)-H(15)	119.9
C(16)-C(15)-H(15)	119.9
C(15)-C(16)-C(11)	120.21(11)

C(15)-C(16)-H(16)	119.9
C(11)-C(16)-H(16)	119.9
C(18)-C(17)-C(22)	118.69(10)
C(18)-C(17)-C(4)	119.12(9)
C(22)-C(17)-C(4)	122.16(9)
C(17)-C(18)-C(19)	121.14(11)
C(17)-C(18)-H(18)	119.4
C(19)-C(18)-H(18)	119.4
C(20)-C(19)-C(18)	119.79(11)
C(20)-C(19)-H(19)	120.1
C(18)-C(19)-H(19)	120.1
C(21)-C(20)-C(19)	119.72(11)
C(21)-C(20)-H(20)	120.1
C(19)-C(20)-H(20)	120.1
C(20)-C(21)-C(22)	120.45(11)
C(20)-C(21)-H(21)	119.8
C(22)-C(21)-H(21)	119.8
C(21)-C(22)-C(17)	120.20(11)
C(21)-C(22)-H(22)	119.9
C(17)-C(22)-H(22)	119.9
C(24)-C(23)-C(28)	119.28(10)
C(24)-C(23)-S(2)	124.06(8)
C(28)-C(23)-S(2)	116.61(8)
C(25)-C(24)-C(23)	119.80(10)
C(25)-C(24)-H(24)	120.1
C(23)-C(24)-H(24)	120.1
C(26)-C(25)-C(24)	120.92(11)
C(26)-C(25)-H(25)	119.5
C(24)-C(25)-H(25)	119.5
C(25)-C(26)-C(27)	119.23(11)
C(25)-C(26)-H(26)	120.4
C(27)-C(26)-H(26)	120.4
C(28)-C(27)-C(26)	120.59(11)
C(28)-C(27)-H(27)	119.7
C(26)-C(27)-H(27)	119.7
C(27)-C(28)-C(23)	120.14(11)
C(27)-C(28)-H(28)	119.9
C(23)-C(28)-H(28)	119.9

Table S14. Anisotropic displacement parameters ($Å^2 \times 10^3$) for **4bad**. The anisotropic displacement factor exponent takes the form: $-2\pi^2[h^2 a^{*2}U^{11} + ... + 2 h k a^* b^* U^{12}]$.

	U^{11}	U^{22}	U^{33}	U^{23}	U^{13}	U^{12}
S(1)	18(1)	24(1)	27(1)	5(1)	3(1)	1(1)
S(2)	27(1)	17(1)	18(1)	0(1)	3(1)	3(1)
C(1)	20(1)	22(1)	22(1)	2(1)	-3(1)	-2(1)
C(2)	19(1)	18(1)	20(1)	0(1)	-4(1)	-3(1)
C(3)	24(1)	18(1)	18(1)	-2(1)	-4(1)	-1(1)
C(4)	22(1)	17(1)	16(1)	-2(1)	-1(1)	0(1)
C(5)	16(1)	25(1)	19(1)	2(1)	-2(1)	2(1)
C(6)	20(1)	24(1)	20(1)	0(1)	1(1)	2(1)
C(7)	29(1)	24(1)	21(1)	-1(1)	-2(1)	1(1)
C(8)	34(1)	28(1)	22(1)	-5(1)	-5(1)	7(1)
C(9)	27(1)	39(1)	21(1)	-6(1)	0(1)	9(1)
C(10)	20(1)	36(1)	20(1)	0(1)	1(1)	4(1)
C(11)	16(1)	22(1)	19(1)	2(1)	-2(1)	-1(1)
C(12)	20(1)	26(1)	20(1)	-2(1)	-2(1)	-2(1)
C(13)	21(1)	40(1)	20(1)	1(1)	-4(1)	-1(1)
C(14)	24(1)	38(1)	26(1)	10(1)	-3(1)	5(1)
C(15)	29(1)	24(1)	32(1)	7(1)	-1(1)	3(1)
C(16)	26(1)	22(1)	25(1)	2(1)	-4(1)	-2(1)
C(17)	20(1)	18(1)	18(1)	-3(1)	1(1)	0(1)
C(18)	21(1)	21(1)	25(1)	-4(1)	-4(1)	-1(1)
C(19)	27(1)	22(1)	34(1)	-9(1)	-2(1)	-4(1)
C(20)	28(1)	18(1)	36(1)	-3(1)	5(1)	2(1)
C(21)	30(1)	22(1)	29(1)	2(1)	-2(1)	5(1)
C(22)	30(1)	20(1)	21(1)	-1(1)	-4(1)	1(1)
C(23)	21(1)	16(1)	21(1)	-2(1)	0(1)	0(1)
C(24)	25(1)	20(1)	22(1)	-1(1)	-1(1)	3(1)
C(25)	28(1)	21(1)	28(1)	2(1)	-2(1)	3(1)
C(26)	27(1)	18(1)	39(1)	-1(1)	1(1)	4(1)
C(27)	29(1)	20(1)	35(1)	-6(1)	7(1)	1(1)
C(28)	29(1)	19(1)	25(1)	-3(1)	5(1)	-1(1)

Table S15. Hydrogen coordinates (x 104) and isotropic displacement parameters (Å^2 x 10^3) for **4bad**.

	x	y	z	U(eq)
H(1)	8671	4372	2817	25
H(3A)	5375	6601	3490	24
H(3B)	6595	5376	3808	24
H(4)	3065	5166	2827	22
H(6)	7324	2668	2110	26
H(7)	7588	522	1949	30
H(8)	10006	-262	991	33
H(9)	12192	1112	199	35
H(10)	11988	3251	367	30
H(12)	4875	5060	1092	27
H(13)	3219	6296	62	33
H(14)	3149	8464	126	36
H(15)	4740	9395	1227	34
H(16)	6389	8164	2270	29
H(18)	2515	3207	2402	27
H(19)	2867	1039	2507	33
H(20)	4713	77	3608	33
H(21)	6171	1289	4604	32
H(22)	5768	3459	4515	28
H(24)	1929	6985	2717	27
H(25)	680	9006	2467	31
H(26)	-963	10037	3572	34
H(27)	-1322	9041	4945	34
H(28)	48	7061	5224	29

Table S16. Torsion angles [°] for **4bad**.

C(5)-S(1)-C(1)-C(2)
S(1)-C(1)-C(2)-C(11)
S(1)-C(1)-C(2)-C(3)
C(1)-C(2)-C(3)-C(4)
C(11)-C(2)-C(3)-C(4)
C(2)-C(3)-C(4)-C(17)
C(2)-C(3)-C(4)-S(2)
C(23)-S(2)-C(4)-C(17)
C(23)-S(2)-C(4)-C(3)
C(1)-S(1)-C(5)-C(6)
C(1)-S(1)-C(5)-C(10)
C(10)-C(5)-C(6)-C(7)
S(1)-C(5)-C(6)-C(7)
C(5)-C(6)-C(7)-C(8)
C(6)-C(7)-C(8)-C(9)
C(7)-C(8)-C(9)-C(10)
C(8)-C(9)-C(10)-C(5)
C(6)-C(5)-C(10)-C(9)
S(1)-C(5)-C(10)-C(9)
C(1)-C(2)-C(11)-C(12)
C(3)-C(2)-C(11)-C(12)
C(1)-C(2)-C(11)-C(16)
C(3)-C(2)-C(11)-C(16)
C(16)-C(11)-C(12)-C(13)
C(2)-C(11)-C(12)-C(13)
C(11)-C(12)-C(13)-C(14)
C(12)-C(13)-C(14)-C(15)
C(13)-C(14)-C(15)-C(16)
C(14)-C(15)-C(16)-C(11)
C(12)-C(11)-C(16)-C(15)
C(2)-C(11)-C(16)-C(15)
C(3)-C(4)-C(17)-C(18)
S(2)-C(4)-C(17)-C(18)
C(3)-C(4)-C(17)-C(22)
S(2)-C(4)-C(17)-C(22)
C(22)-C(17)-C(18)-C(19)

C(4)-C(17)-C(18)-C(19)

C(17)-C(18)-C(19)-C(20)

C(18)-C(19)-C(20)-C(21)

C(19)-C(20)-C(21)-C(22)

C(20)-C(21)-C(22)-C(17)

C(18)-C(17)-C(22)-C(21)

C(4)-C(17)-C(22)-C(21)

C(4)-S(2)-C(23)-C(24)

C(4)-S(2)-C(23)-C(28)

C(28)-C(23)-C(24)-C(25)

S(2)-C(23)-C(24)-C(25)

C(23)-C(24)-C(25)-C(26)

C(24)-C(25)-C(26)-C(27)

C(25)-C(26)-C(27)-C(28)

C(26)-C(27)-C(28)-C(23)

C(24)-C(23)-C(28)-C(27)

S(2)-C(23)-C(28)-C(27)

Characterization data of products

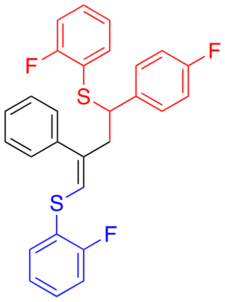

(*Z*)-(4-(4-fluorophenyl)-2-phenylbut-1-ene-1,4-diyl)bis((2-fluorophenyl)sulfane) (4aaa).
^{1}H NMR ($CDCl_3$, 300 MHz): 7.41-7.31 (3H, m), 7.22-7.07 (7H, m), 7.05-6.87 (7H, m), 6.05 (1H, s), 4.18 (1H, dd, J = 9.3, 6.1 Hz), 3.19 (1H, ddd, J = 14.2, 6.0, 0.8 Hz), 3.05 (1H, dd, J = 14.2, 9.2 Hz). **^{13}C{^{1}H} NMR** ($CDCl_3$, 75 MHz): 162.6 (d, J(C-F) = 246.3 Hz), 162.1 (d, J(C-F) = 245.9 Hz), 160.3 (d, J(C-F) = 245.6 Hz), 140.5, 138.1, 136.4 (d, J(C-F) = 3.1 Hz), 135.5 (d, J(C-F) = 1.0 Hz), 130.8 (d, J(C-F) = 2.2 Hz), 130.0 (d, J(C-F) = 8.2 Hz), 129.7 (d, J(C-F) = 8.1 Hz), 128.5, 128.3 (d, J(C-F) = 7.6 Hz), 128.2, 128.1, 124.7 (d, J(C-F) = 3.8 Hz), 124.4 (d, J(C-F) = 3.8 Hz), 123.8 (d, J(C-F) = 17.1 Hz), 122.0 (d, J(C-F) = 1.6 Hz), 121.1 (d, J(C-F) = 18.1 Hz), 115.8 (d, J(C-F) = 23.4 Hz), 115.7 (d, J(C-F) = 21.7 Hz), 115.4 (d, J(C-F) = 21.8 Hz), 49.9 (d, J(C-F) = 2.2 Hz), 45.7. **^{19}F NMR** ($CDCl_3$, 283 MHz): -102.6 (1F, s), -105.7 (1F, s), -109.9 (1F, s). **HRMS** (ESI): *m/z* = 545.0824 calcd for $C_{29}H_{20}S_2F_6$ $[M-H]^+$ = 545.0827 (Δ = 0.6 ppm).

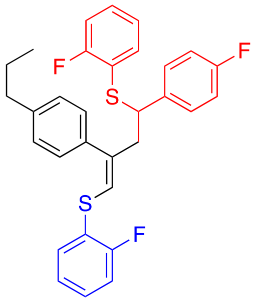

(Z)-(4-(4-fluorophenyl)-2-(4-propylphenyl)but-1-ene-1,4-diyl)bis((2-fluorophenyl)sulfane) (4aba).
^{1}H NMR ($CDCl_3$, 300 MHz): 7.21-7.06 (9H, m), 7.03-6.86 (7H, m), 6.00 (1H, s), 4.20 (1H, dd, J = 9.4, 5.9 Hz), 3.18 (1H, dd, J = 14.3, 6.0 Hz), 3.03 (1H, dd, J = 14.0, 9.4 Hz). 2.61 (2H, t, J = 7.6 Hz), 1.75-1.61 (2H, sext, J = 7.5 Hz), 0.98 (3H, t, J = 7.3 Hz). **^{13}C{^{1}H} NMR** (Acetone-*d*$_6$, 75.5 MHz): 163.0 (d, J(C-F) = 244.6 Hz), 162.8 (d, J(C-F) = 244.1 Hz), 160.7 (d, J(C-F) = 243.4 Hz), 143.2, 142.2, 137.6 (d, J(C-F) = 3.1 Hz), 136.4, 135.7 (d, J(C-F) = 1.6 Hz), 130.92 (d, J(C-F) = 7.9 Hz), 130.90 (d, J(C-F) = 1.6 Hz), 130.8 (d, J(C-F) = 8.4 Hz), 129.2, 129.0 (d, J(C-F) = 7.7 Hz), 128.9, 125.8 (d, J(C-F) = 3.6 Hz), 125.4 (d, J(C-F) = 3.7 Hz), 124.6 (d, J(C-F) = 17.0 Hz), 122.1 (d, J(C-F) = 17.9 Hz), 121.2 (d, J(C-F) = 0.9 Hz), 116.4 (d, J(C-F) = 22.9 Hz), 116.2 (d, J(C-F) = 21.6 Hz), 115.9 (d, J(C-F) = 21.6 Hz), 50.4 (d, J(C-F) = 1.7 Hz), 45.8, 38.3, 25.2, 14.1. **^{19}F{^{1}H} NMR** ($CDCl_3$, 283 MHz): -102.6 (1F, s), -105.8 (1F, s), -109.9 (1F, s) **HRMS** (ESI): *m/z* = 521.1573 calcd for $C_{31}H_{28}F_3S_2$ $[M+H]^+$ = 521.1579 (Δ = 1.2 ppm).

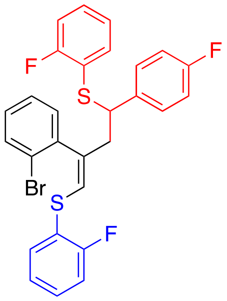

(Z)-(2-(2-bromophenyl)-4-(4-fluorophenyl)but-1-ene-1,4-diyl)bis((2-fluorophenyl)sulfane) (4aca).
^{1}H NMR (DMSO-*d*$_6$, 400 MHz, 373 K): 7.64 (1H, d, J = 7.9 Hz), 7.36-7.21 (7H, m), 7.21-7.09 (4H, m), 7.09-7.00 (4H, m), 6.43 (1H, s), 4.42 (1H, dd, J = 8.7, 6.4 Hz), 3.22 (1H, dd, J = 15.0, 8.9 Hz), 3.15 (1H, dd, J = 15.0, 6.1 Hz). **^{13}C{^{1}H} NMR** (DMSO-*d*$_6$, 100 MHz, 373 K): 161.1 (d, J(C-F) = 244.6 Hz), 160.9 (d, J(C-F) = 244.3 Hz), 159.0 (d, J(C-F) = 244.1 Hz), 139.8, 138.6, 135.9, 134.3 (d, J(C-F) = 1.1 Hz), 132.2, 130.14, 130.07 (d, J(C-F) = 1.8 Hz), 129.5 (d, J(C-F) = 8.1 Hz), 129.4 (d, J(C-F) = 8.2 Hz), 129.1, 128.1 (d, J(C-F) = 7.7 Hz), 127.0, 124.4 (d, J(C-F) = 3.3 Hz), 124.1 (d, J(C-F) = 3.7 Hz), 122.7 (d, J(C-F) = 1.8 Hz), 121.7 (d, J(C-F) = 17.2 Hz), 121.2, 120.1 (d, J(C-F) = 18.3 Hz), 115.1 (d, J(C-F) = 22.7 Hz), 115.0 (d, J(C-F) = 21.7 Hz), 114.4 (d, J(C-F) = 21.5 Hz), 48.6, 42.4. **^{19}F NMR** (DMSO-*d*$_6$, 376 MHz, 373 K): -103.5 – -103.6 (1F, m), -106.5 – -106.7 (1F, m), -110.4 – -110.5 (1F, m). **HRMS** (ESI): *m/z* = 596.9746 calcd for $C_{28}H_{20}F_3S_2BrK$ $[M+K]^+$ = 596.9755 (Δ = 1.8 ppm).

(Z)-(2,4-bis(4-fluorophenyl)but-1-ene-1,4-diyl)bis((2-fluorophenyl)sulfane) (4ada).

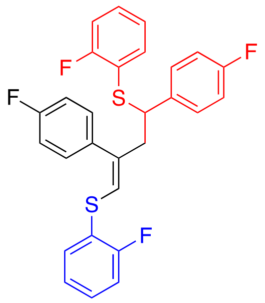

^{1}H NMR ($CDCl_3$, 300 MHz): 7.23-7.13 (4H, m), 7.13-6.99 (8H, m), 6.99-6.87 (4H, m), 6.07 (1H, s), 4.14 (1H, dd, J = 9.0, 6.4 Hz), 3.15 (1H, dd, J = 13.9, 6.1 Hz), 3.03 (1H, dd, J = 14.2, 9.2 Hz). **^{13}C{^{1}H} NMR** ($CDCl_3$, 75.5 MHz): 162.6 (d, J(C-F) = 246.5 Hz), 162.3 (d, J(C-F) = 247.4 Hz), 162.1 (d, J(C-F) = 246.1 Hz), 160.4 (d, J(C-F) = 245.8 Hz), 139.3, 136.3 (d, J(C-F) = 3.4 Hz), 135.7 (d, J(C-F) = 1.1 Hz), 133.9 (d, J(C-F) = 3.3 Hz), 130.9 (d, J(C-F) = 2.2 Hz), 130.1 (d, J(C-F) = 8.0 Hz), 130.0 (d, J(C-F) = 8.0 Hz), 129.7 (d, J(C-F) = 8.1 Hz), 128.5 (d, J(C-F) = 7.6 Hz), 124.7 (d, J(C-F) = 3.8 Hz), 124.4 (d, J(C-F) = 3.8 Hz), 123.4 (d, J(C-F) = 16.9 Hz), 122.5-122.4 (br. s), 121.0 (d, J(C-F) = 18.2 Hz), 115.8 (d, J(C-F) = 22.4 Hz), 115.75 (d, J(C-F) = 21.6 Hz), 115.55 (d, J(C-F) = 21.4 Hz), 115.4 (d, J(C-F) = 21.5 Hz), 49.9 (d, J(C-F) = 2.4 Hz), 45.7. **^{19}F{^{1}H} NMR** ($CDCl_3$, 283 MHz): -102.5 (1F, s), -105.5 (1F, s), -108.6 (1F, s), -109.7 (1F, s). **HRMS** (ESI): *m/z* = 535.0569 calcd for $C_{28}H_{20}F_4S_2K$ $[M+K]^+$ = 535.0574 (Δ = 0.9 ppm).

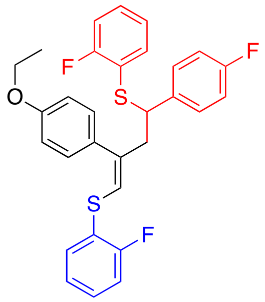

(Z)-(2-(4-ethoxyphenyl)-4-(4-fluorophenyl)but-1-ene-1,4-diyl)bis((2-fluorophenyl)sulfane) (4aea).

1H NMR ($CDCl_3$, 300 MHz): 7.22-7.07 (7H, m), 7.04-6.85 (9H, m), 5.98 (1H, s), 4.18 (1H, dd, J_1 = 9.3, 6.0 Hz), 4.07 (2H, q, J = 7.0 Hz), 3.18 (1H, dd, J = 14.0, 5.8 Hz), 3.02 (1H, dd, J = 14.1, 9.4 Hz), 1.44 (3H, t, J = 7.0 Hz). **^{13}C{^{1}H} NMR** ($CDCl_3$, 75.5 MHz): 162.5 (d, J(C-F) = 246.4 Hz), 162.1 (d, J(C-F) = 245.8 Hz), 160.3 (d, J(C-F) = 245.5 Hz), 158.7, 140.4, 136.4 (d, J(C-F) = 3.3 Hz), 135.5 (d, J(C-F) = 1.6 Hz), 130.6 (d, J(C-F) = 1.6 Hz), 130.0 (d, J(C-F) = 1.1 Hz), 129.9 (d, J(C-F) = 7.6 Hz), 129.7 (d, J(C-F) = 8.2 Hz), 129.5, 128.1 (d, J(C-F) = 7.7 Hz), 124.7 (d, J(C-F) = 3.8 Hz), 124.4 (d, J(C-F) = 3.9 Hz), 124.1 (d, J(C-F) = 16.9 Hz), 121.2 (d, J(C-F) = 18.0 Hz), 120.8 (d, J(C-F) = 1.6 Hz), 115.8 (d, J(C-F) = 23.5 Hz), 115.6 (d, J(C-F) = 21.6 Hz), 115.3 (d, J(C-F) = 21.4 Hz), 114.4, 63.6, 49.9 (d, J(C-F) = 2.0 Hz), 45.8, 15.0. **^{19}F{^{1}H} NMR** ($CDCl_3$, 283 MHz): -102.5 (1F, s), -105.9 (1F, s), -109.9 (1F, s). **HRMS** (ESI): *m/z* = 629.0344 calcd for $C_{30}H_{25}F_3S_2OAg$ $[M+Ag]^+$ = 629.0344 (Δ = 0 ppm).

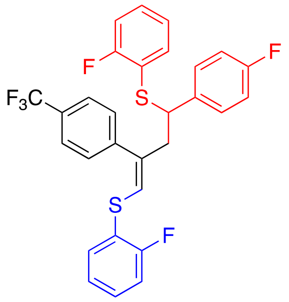

(Z)-(4-(4-fluorophenyl)-2-(4-(trifluoromethyl) phenyl)but-1-ene-1,4-diyl)bis((2-fluorophenyl)sulfane) (4afa).

^{1}H NMR ($CDCl_3$, 300 MHz): 7.61 (2H, d, J = 8.2 Hz), 7.31 (2H, d, J = 8.1 Hz), 7.24-7.16 (2H, m), 7.13-6.98 (6H, m), 6.98-6.85 (4H, m), 6.16 (1H, s), 4.13 (1H, dd, J = 9.0, 6.5 Hz), 3.17 (1H, dd, J = 14.4, 6.5 Hz), 3.06 (1H, dd, J =14.2, 9.1 Hz). **^{13}C{^{1}H} NMR** ($CDCl_3$, 75 MHz): 162.7 (d, J(C-F) = 246.4 Hz), 162.1 (d, J(C-F) = 246.4 Hz), 160.5 (d, J(C-F) = 245.3 Hz), 141.9 (d, J(C-F) = 1.4 Hz), 138.5, 136.2 (d, J(C-F) = 3.3 Hz), 135.8 (d, J(C-F) = 1.0 Hz), 131.3 (d, J(C-F) = 1.1 Hz), 130.3 (d, J(C-F) = 8.1 Hz), 130.0 (q J(C-F) = 32.6 Hz), 129.7 (d, J(C-F) = 8.1 Hz), 128.9 (d, J(C-F) = 7.6 Hz), 128.7, 125.6 (q, J(C-F) = 3.8 Hz), 124.8 (d, J(C-F) = 3.7 Hz), 124.4 (d, J(C-F) = 3.8 Hz), 124.2 (q, J(C-F) = 272.1 Hz), 124.1 (d, J(C-F) = 1.0 Hz), 122.9 (d, J(C-F) = 17.2 Hz), 120.8 (d, J(C-F) = 18.0 Hz), 115.88 (d, J(C-F) = 23.4 Hz), 115.87 (d, J(C-F) = 22.2 Hz), 115.5 (d, J(C-F) = 21.4 Hz), 50.0 (d, J(C-F) = 2.4 Hz), 45.5. **^{19}F{^{1}H} NMR** ($CDCl_3$, 283 MHz): -57.7 (3F, s), -102.4 (1F, s), -105.2 (1F, s), -109.6 (1F, s). **HRMS** (ESI): *m/z* = 654.9954 calcd for $C_{29}H_{20}F_6S_2Ag$ $[M+Ag]^+$ = 654.9953 (Δ = 0.2 ppm).

(Z)-4-(4-(4-fluorophenyl)-1,4-bis((2-fluorophenyl)thio)but-1-en-2-yl)-*N*,*N*-dimethylaniline) (4aga).

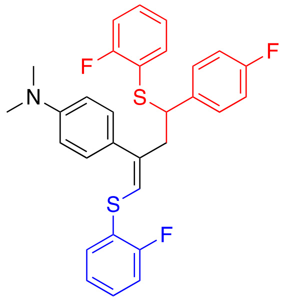

^{1}H NMR ($CDCl_3$, 300 MHz): 7.19-7.07 (7H, m), 7.02-6.89 (7H, m), 6.78-6.66 (2H, d, J = 8.0 Hz), 5.90 (1H, s), 4.24 (1H, dd, J = 9.3, 5.9 Hz), 3.21 (1H, dd, J = 14.0, 5.9 Hz), 3.07-2.96 (7H, m). **^{13}C{^{1}H} NMR** ($CDCl_3$, 75 MHz): 162.5 (d, J(C-F) = 246.3 Hz), 162.0 (d, J(C-F) = 245.6 Hz), 160.1 (d, J(C-F) = 245.6 Hz), 150.1, 141.2, 136.6 (d, J(C-F) = 3.2 Hz), 135.4 (d, J(C-F) = 1.0 Hz), 130.3 (d, J(C-F) = 1.9 Hz), 129.79 (d, J(C-F) = 8.0 Hz), 129.77 (d, J(C-F) = 8.1 Hz), 129.2, 127.8 (d, J(C-F) = 7.6 Hz), 127.4, 124.6 (d, J(C-F) = 3.7 Hz), 124.5 (d, J(C-F) = 17.4 Hz), 124.4 (d, J(C-F) = 3.8 Hz), 121.4 (d, J(C-F) = 18.1 Hz), 119.1, 115.8 (d, J(C-F) = 23.0 Hz), 115.5 (d, J(C-F) = 21.7 Hz), 115.3 (d, J(C-F) = 21.4 Hz), 112.0, 50.0 (d, J(C-F) = 2.0 Hz), 45.7, 40.6. **^{19}F{^{1}H} NMR** ($CDCl_3$, 283 MHz): -102.6 (1F, s), -106.2 (1F, s), -110.1 (1F, s). **HRMS (ESI)**: *m/z* = 630.0501 calcd for $C_{30}H_{26}F_3NS_2Ag$ $[M+Ag]^+$ = 630.0501 (Δ = 0.0 ppm).

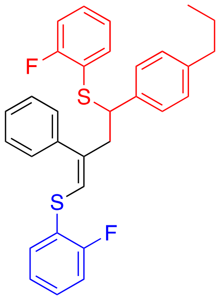

(Z)-(2-phenyl-4-(4-propylphenyl)but-1-ene-1,4-diyl)bis((2-fluorophenyl)sulfane) (4aab).

^{1}H NMR ($CDCl_3$, 300 MHz): 7.40-7.30 (3H, m), 7.22-6.93 (14H, m), 6.07 (1H, s), 4.18 (1H, dd, J = 8.6, 5.6 Hz), 3.22-3.06 (2H, m), 2.59-2.51 (2H, m), 1.69-1.55 (2H, sext, J = 7.5 Hz), 0.91 (3H, t, 7.3 Hz). **^{13}C{^{1}H} NMR** (acetone-*d*$_6$, 75 MHz): 162.9 (d, J(C-F) = 244.5 Hz), 160.6 (d, J(C-F) = 244.5 Hz), 142.7, 142.5, 139.2, 138.6, 135.4 (d, J(C-F) = 1.0 Hz), 130.9 (d, J(C-F) = 1.7 Hz), 130.5 (d, J(C-F) = 8.1 Hz), 129.3, 129.2, 129.98 128.95 (d, J(C-F) = 7.5 Hz), 128.9, 128.7, 125.8, (d, J(C-F) = 3.5 Hz), 125.4, (d, J(C-F) = 3.5 Hz), 124.6 (d, J(C-F) = 17.1 Hz), 122.6 (d, J(C-F) = 18.0 Hz), 121.4 (d, J(C-F) = 1.4 Hz), 116.4 (d, J(C-F) = 23.0 Hz), 116.2 (d, J(C-F) = 21.6 Hz), 50.7 (d, J(C-F) = 2.2 Hz), 45.9, 38.2, 25.2, 14.0. **^{19}F{^{1}H} NMR** ($CDCl_3$, 283 MHz): -102.7 (1F, s), -105.9 (1F, s). **HRMS** (ESI): *m/z* = 503.1667 calcd for $C_{31}H_{29}F_2S_2$ $[M+H]^+$ = 503.1673 (Δ = 1.2 ppm).

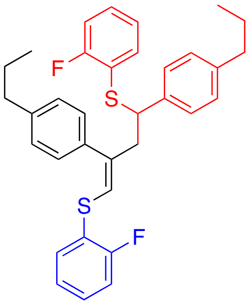

(Z)-(2,4-bis(4-propylphenyl)but-1-ene-1,4-diyl)bis((2-fluorophenyl)sulfane) (4abb).

^{1}H NMR ($CDCl_3$, 300 MHz): 7.20-7.02 (11H, m), 7.02-6.89 (5H, m), 6.01 (1H, s), 4.20 (1H, dd, J = 8.8, 6.4 Hz), 3.20-3.04 (2H, m), 2.71-2.54 (4H, m), 1.76-1.55 (4H, m), 0.97 (3H, t, J = 7.3 Hz), 0.91 (3H, t, J = 7.3 Hz). **^{13}C{^{1}H} NMR** ($CDCl_3$, 75 MHz): 162.4 (d, J(C-F) = 246.0 Hz), 160.2 (d, J(C-F) = 245.8 Hz), 142.4, 141.9, 141.0, 137.7, 135.5, 135.2 (d, J(C-F) = 0.8 Hz), 130.5 (d, J(C-F) = 1.6 Hz), 129.5 (d, J(C-F) = 8.1 Hz), 128.6, 128.5, 128.1, 128.0, 127.8 (d, J(C-F) = 7.7 Hz), 124.6 (d, J(C-F) = 3.6 Hz), 124.3 (d, J(C-F) = 16.6 Hz), 124.2 (d, J(C-F) = 3.8 Hz), 121.9 (d, J(C-F) = 18.1 Hz), 120.8, 115.7 (d, J(C-F) = 23.0 Hz), 115.5 (d, J(C-F) = 21.8 Hz), 50.3 (d, J(C-F) = 1.6 Hz), 45.7, 38.0, 37.8, 24.54, 24.49, 14.0, 13.9. **^{19}F{^{1}H} NMR** ($CDCl_3$, 283 MHz): -102.7 (1F, s), -106.0 (1F, s). **HRMS** (ESI): *m/z* = 651.1115 calcd for $C_{34}H_{34}F_2S_2Ag$ $[M+Ag]^+$ = 651.1115 (Δ = 0.0 ppm).

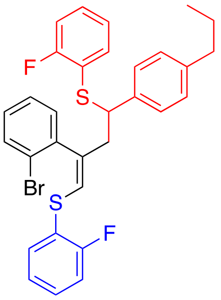

(Z)-(2-(2-bromophenyl)-4-(4-propylphenyl)but-1-ene-1,4-diyl)bis((2-fluorophenyl)sulfane) (4acb).

^{1}H NMR (DMSO-*d*$_6$, 400 MHz, 373 K): 7.63 (1H, d, J = 7.5 Hz), 7.33 (1H, t, J = 7.5 Hz), 7.31-7.17 (6H, m), 7.17-7.05 (6H, m), 7.04-6.98 (2H, m), 6.39 (1H, s), 4.39 (1H, dd, J = 8.9, 6.1 Hz), 3.22 (1H, dd, J = 14.9, 9.1 Hz), 3.12 (1H, dd, J = 14.9, 5.9 Hz), 2.54 (2H, t, J = 7.4 Hz), 1.56 (2H, sext, J = 7.4 Hz), 0.88 (3H, t, J = 7.3 Hz). **^{13}C{^{1}H} NMR** (DMSO-*d*$_6$, 100 MHz, 373 K): 161.0 (d, J(C-F) = 247.4 Hz), 158.9 (d, J(C-F) = 244.1 Hz), 140.9, 140.2, 138.7, 136.8, 133.9, 132.2, 130.1, 129.9, 129.2 (d, J(C-F) = 8.1 Hz), 129.0, 128.0 (d, J(C-F) = 7.3 Hz), 127.6, 127.3, 126.9, 124.4 (d, J(C-F) = 3.7 Hz), 124.0 (d, J(C-F) =

3.6 Hz), 122.3, 121.8 (d, J(C-F) = 16.9 Hz), 121.1, 120.6 (d, J(C-F) = 18.3 Hz), 115.0 (d, J(C-F) = 23.5 Hz), 114.9 (d, J(C-F) = 21.6 Hz), 49.0, 42.4, 36.3, 23.0, 12.7. ^{19}**F NMR** (DMSO-d_6, 376 MHz, 373 K): -108.2 (1F, m), -111.2 (1F, m). **HRMS** (ESI): *m/z* = 621.0314 calcd for $C_{31}H_{27}F_2BrS_2K$ $[M+K]^+$ = 621.0318 (Δ = 0.6 ppm).

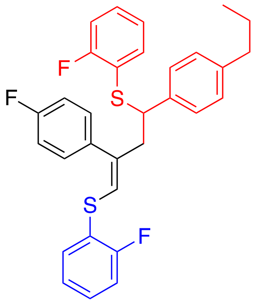

(Z)-(2-(4-fluorophenyl)-4-(4-propylphenyl)but-1-ene-1,4-diyl)bis((2-fluorophenyl)sulfane) (4adb).
^{1}**H NMR** ($CDCl_3$, 300 MHz): 7.24-7.09 (5H, m), 7.09-6.88 (11H, m), 6.08 (1H, s), 4.14 (1H, dd, J = 8.3, 7.0 Hz), 3.19-3.02 (2H, m), 2.59-2.51 (2H, m), 1.68-1.54 (2H, sext, J = 7.5 Hz), 0.90 (3H, t, J = 7.3 Hz). ^{13}**C{^{1}H} NMR** ($CDCl_3$, 75 MHz): 162.5 (d, J(C-F) = 246.0 Hz), 162.3 (d, J(C-F) = 247.1 Hz), 160.3 (d, J(C-F) = 245.9 Hz), 142.1, 139.9, 137.6, 135.4 (d, J(C-F) = 0.9 Hz), 134.2 (d, J(C-F) = 3.3 Hz), 130.8 (d, J(C-F) = 1.4 Hz), 130.1 (d, J(C-F) = 8.3 Hz), 129.7 (d, J(C-F) = 7.7 Hz), 128.6, 128.2 (d, J(C-F) = 7.4 Hz), 127.9, 124.7 (d, J(C-F) = 3.6 Hz), 124.3 (d, J(C-F) = 3.7 Hz), 123.7 (d, J(C-F) = 17.1 Hz), 122.0, 121.6 (d, J(C-F) = 18.2 Hz), 115.7 (d, J(C-F) = 23.4 Hz), 115.6 (d, J(C-F) = 21.7 Hz), 115.4 (d, J(C-F) = 21.6 Hz), 50.4 (d, J(C-F) = 1.8 Hz), 45.7, 37.8, 24.5, 13.9. ^{19}**F{^{1}H} NMR** ($CDCl_3$, 283 MHz): -102.6 (1F, s), -105.7 (1F, s), -108.9 (1F, s). **HRMS** (ESI): *m/z* = 627.0526 calcd for $C_{31}H_{27}F_3S_2Ag$ $[M+Ag]^+$ = 627.0552 (Δ = 4.1 ppm).

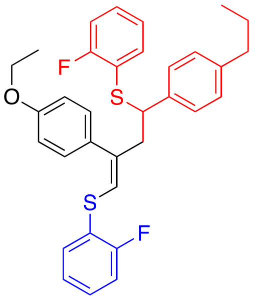

(Z)-(2-(4-ethoxyphenyl)-4-(4-propylphenyl)but-1-ene-1,4-diyl)bis((2-fluorophenyl)sulfane) (4aeb).
^{1}**H NMR** ($CDCl_3$, 300 MHz): 7.20-7.01 (9H, m), 7.01-6.83 (7H, m), 6.00 (1H, s), 4.17 (1H, dd, J = 8.7, 6.4 Hz), 4.06 (2H, q, J = 7.0 Hz), 3.20-3.02 (2H, m), 2.59-2.51 (2H, m), 1.70-1.54 (2H, sext, J = 7.5 Hz), 1.44 (3H, t, J = 7.0 Hz), 0.91 (3H, t, J = 7.3 Hz). ^{13}**C{^{1}H} NMR** ($CDCl_3$, 75 MHz): 162.4 (d, J(C-F) = 245.9 Hz), 160.1 (d, J(C-F) = 245.4 Hz), 158.6, 142.0, 140.9, 137.7, 135.2 (d, J(C-F) = 1.1 Hz), 130.4 (d, J(C-F) = 1.6 Hz), 130.3, 129.53, 129.52 (d, J(C-F) = 7.9 Hz), 128.6, 127.9, 127.8 (d, J(C-F) = 7.6 Hz), 124.6 (d, J(C-F) = 3.3 Hz), 124.3 (d, J(C-F) = 16.9 Hz), 124.3 (d, J(C-F) = 3.8 Hz), 121.9 (d, J(C-F) = 18.0 Hz), 120.4-120.3 (br s), 115.7 (d, J(C-F) = 22.9 Hz), 115.5 (d, J(C-F) = 21.7 Hz), 114.3, 63.5, 50.4 (d, J(C-F) = 1.9 Hz), 45.8, 24.6, 37.8, 15.0, 13.9. ^{19}**F{^{1}H} NMR** ($CDCl_3$, 283 MHz): -103.2 (1F, s), -106.5 (1F, s). **HRMS (ESI)**: *m/z* = 655.0907 calcd for $C_{33}H_{32}F_2OS_2Ag$ $[M+Ag]^+$ = 655.0905 (Δ = 0.3 ppm).

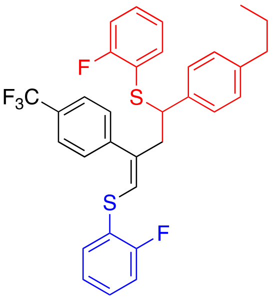

(Z)-(4-(4-propylphenyl)-2-(4-(trifluoromethyl)phenyl)but-1-ene-1,4-diyl)bis((2-fluorophenyl)sulfane) (4afb).
^{1}**H NMR** ($CDCl_3$, 300 MHz): 7.58 (2H, d, J = 8.2 Hz), 7.28 (2H, d, J = 8.2 Hz), 7.22-7.09 (3H, m) 7.09-6.88 (9H, m), 6.17 (1H, s), 4.20-4.06 (1H, m), 3.21-3.05 (2H, m), 2.58-2.50 (2H, m), 1.68-1.54 (2H, sext, J = 7.5 Hz), 0.91 (3H, t, J = 7.3 Hz). ^{13}**C{^{1}H} NMR** ($CDCl_3$, 75 MHz): 162.6 (d, J(C-F) = 246.3 Hz), 160.4 (d, J(C-F) = 246.2 Hz), 142.2, 142.1 (q, J(C-F) = 1.1 Hz), 139.1, 137.4, 135.6 (d, J(C-F) = 1.4 Hz), 131.1 (d, J(C-F) = 1.7 Hz), 129.9 (d, J(C-F) = 8.0 Hz), 129.8 (q J(C-F) = 32.4 Hz), 128.7, 128.65, 128.6 (d, J(C-F) = 7.7 Hz), 127.9, 125.4 (d, J(C-F) = 3.7 Hz), 124.8 (d, J(C-F) = 3.6 Hz), 124.3 (d, J(C-F) = 3.7 Hz), 124.25 (q J(C-F) = 272.1 Hz), 123.6 (d, J(C-F) = 1.7 Hz), 123.2 (d, J(C-F) = 17.1 Hz), 121.5 (d, J(C-F) = 18.2 Hz), 115.79 (d, J(C-F) = 23.7 Hz), 115.77 (d, J(C-F) = 21.7 Hz), 50.5 (d, J(C-F) = 2.2 Hz), 45.5, 37.8, 24.5, 13.9. ^{19}**F{^{1}H} NMR** ($CDCl_3$, 283 MHz): -57.7 (3F, s), -102.6 (1F, s), -105.4 (1F, s). **HRMS** (ESI): *m/z* = 679.0513 calcd for $C_{32}H_{27}F_5S_2Ag$ $[M+Ag]^+$ = 679.0517 (Δ = 0.6 ppm).

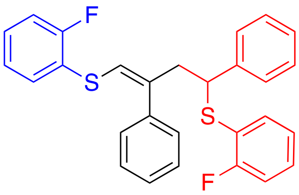

(Z)-(2,4-diphenylbut-1-ene-1,4-diyl)bis((2-fluorophenyl)sulfane) (4aac).
^{1}H NMR ($CDCl_3$, 300 MHz): 7.40-7.30 (3H, m), 7.25-7.08 (10H, m), 7.03-6.87 (5H, m), 6.06 (1H, s), 4.20 (1H, dd, J = 8.9, 6.4 Hz), 3.24-3.06 (2H, m). **^{13}C{^{1}H} NMR** (Acetone-d_6, 100 MHz): 162.9 (d, J(C-F) = 244.5 Hz), 160.5 (d, J(C-F) = 243.7 Hz), 142.6, 141.4, 139.1, 135.5 (d, J(C-F) = 0.9 Hz), 130.7 (d, J(C-F) = 1.7 Hz), 130.6 (d, J(C-F) = 8.0 Hz), 129.3, 129.2, 129.01, 128.97, 128.9 (d, J(C-F) = 7.7 Hz), 128.7, 128.3, 125.9 (d, J(C-F) = 3.7 Hz), 125.4 (d, J(C-F) = 3.6 Hz), 124.6 (d, J(C-F) = 16.8 Hz), 122.4 (d, J(C-F) = 18.0 Hz), 121.3, 116.4 (d, J(C-F) = 23.1 Hz), 116.1 (d, J(C-F) = 21.9 Hz), 50.9, 45.9. **^{19}F{^{1}H} NMR** ($CDCl_3$, 283 MHz): -102.7 (1F, s), -106.0 (1F, s). **HRMS** (ESI): *m/z* = 459.1037 calcd for $C_{28}H_{21}S_2$ $[M\text{-}H]^+$ = 459.1047 (Δ = 2.2 ppm).

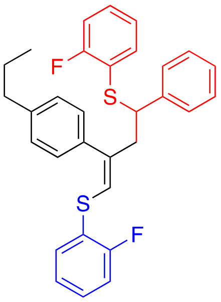

(Z)-(4-phenyl-2-(4-propylphenyl)but-1-ene-1,4-diyl)bis((2-fluorophenyl)sulfane) (4abc).
^{1}H NMR ($CDCl_3$, 300 MHz): 7.25-7.07 (12H, m), 7.02-6.85 (5H, m), 6.00 (1H, s), 4.21 (dd, J = 9.0, 6.2 Hz), 3.24-3.04 (2H, m), 2.65-2.57 (2H, m), 1.76-1.58 (2H, sext, J = 7.5 Hz), 0.98 (3H, t, J = 7.3 Hz). **^{13}C{^{1}H} NMR** ($CDCl_3$, 75 MHz): 162.4 (d, J(C-F) = 246.3 Hz), 160.1 (d, J(C-F) = 245.5 Hz), 142.5, 141.1, 140.6, 135.4, 135.2 (d, J(C-F) = 1.3 Hz), 130.4 (d, J(C-F) = 1.8 Hz), 129.6 (d, J(C-F) = 8.1 Hz), 128.54, 128.53, 128.2, 128.1, 127.8 (d, J(C-F) = 7.6 Hz), 127.5, 124.7 (d, J(C-F) = 3.6 Hz), 124.3 (d, J(C-F) = 3.7 Hz), 124.25 (d, J(C-F) = 17.4 Hz), 121.6 (d, J(C-F) = 18.0 Hz), 120.9 (d, J(C-F) = 1.0 Hz), 115.7 (d, J(C-F) = 22.9 Hz), 115.5 (d, J(C-F) = 21.7 Hz), 50.5 (d, J(C-F) = 2.2 Hz), 45.7, 38.0, 24.5, 14.1. **^{19}F{^{1}H} NMR** ($CDCl_3$, 283 MHz): -102.7 (1F, s), -106.1 (1F, s) **HRMS** (ESI): *m/z* = 609.0649 calcd for $C_{31}H_{28}F_2S_2Ag$ $[M+Ag]^+$ = 609.0646 (Δ = 0.5 ppm).

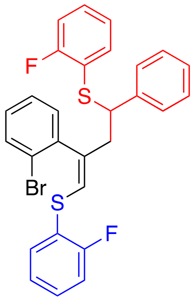

(Z)-(2-(2-bromophenyl)-4-phenylbut-1-ene-1,4-diyl)bis((2-fluorophenyl)sulfane) (4acc).
^{1}H NMR (DMSO-d_6, 400 MHz, 373 K): 7.66-7.61 (1H, m), 7.36-7.32 (1H, m), 7.31-7.22 (9H, m), 7.15-7.06 (4H, m), 7.06-6.99 (2H, m), 6.40 (1H, s), 4.41 (1H, dd, J = 8.9, 6.1 Hz), 3.24 (1H, dd, J = 15.0, 9.1 Hz), 3.15 (1H, dd, J = 15.0, 6.0 Hz). **^{13}C{^{1}H} NMR** (DMSO-d_6, 100 MHz, 373 K): 161.1 (d, J(C-F) = 244.6 Hz), 158.9 (d, J(C-F) = 244.2 Hz), 140.1, 139.7, 138.7, 134.0, 132.2, 130.1, 129.9, 129.3 (d, J(C-F) = 8.1 Hz), 129.1, 128.0 (d, J(C-F) = 7.3 Hz), 127.7, 127.4, 127.0, 126.8, 124.4 (d, J(C-F) = 3.7 Hz), 124.0 (d, J(C-F) = 3.7 Hz), 122.4 (d, J(C-F) = 1.5 Hz), 121.8 (d, J(C-F) = 17.2 Hz), 121.1, 120.4 (d, J(C-F) = 18.0 Hz), 115.0 (d, J(C-F) = 22.9 Hz), 114.9 (d, J(C-F) = 21.6 Hz), 49.3, 42.4. **^{19}F{^{1}H} NMR** (DMSO-d_6, 376 MHz, 373 K): -108.2 (1F, m), -111.3 (1F, m). **HRMS** (ESI): *m/z* = 646.9276 calcd for $C_{28}H_{21}F_2BrS_2Ag$ $[M+Ag]^+$ = 646.9264 (Δ = 1.9 ppm).

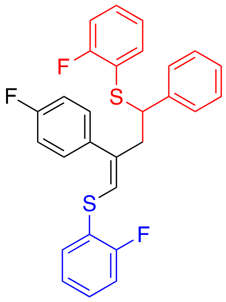

(Z)-(2-(4-fluorophenyl)-4-phenylbut-1-ene-1,4-diyl)bis((2-fluorophenyl)sulfane) (4adc).
^{1}H NMR ($CDCl_3$, 300 MHz): 7.25-7.09 (10H, m), 7.09-6.88 (7H, m), 6.07 (1H, s), 4.16 (1H, dd, J = 8.7, 6.7 Hz), 3.20-3.04 (2H, m). **^{13}C{^{1}H} NMR** ($CDCl_3$, 75 MHz): 162.6 (d, J(C-F) = 246.3 Hz), 162.3 (d, J(C-F) = 247.2 Hz), 160.3 (d, J(C-F) = 245.9 Hz), 140.5, 139.9, 135.5 (d, J(C-F) = 1.6 Hz), 134.1 (d, J(C-F) = 3.3 Hz), 130.7 (d, J(C-F) = 1.6 Hz), 130.1 (d, J(C-F) = 8.2 Hz), 129.9 (d, J(C-F) = 8.1 Hz), 128.6, 128.2 (d, J(C-F) = 7.6 Hz), 128.1, 127.6, 124.8 (d, J(C-F) = 3.8 Hz), 124.3 (d, J(C-F) = 3.8 Hz), 123.7 (d, J(C-F) = 16.8 Hz), 122.0, 121.4 (d, J(C-F) = 18.0 Hz), 115.8 (d, J(C-F) = 21.8 Hz), 115.6 (d, J(C-F) = 22.1 Hz), 115.5 (d, J(C-F) = 20.6 Hz), 50.6 (d, J(C-F) = 2.2 Hz), 45.7. **^{19}F{^{1}H} NMR** ($CDCl_3$,

282.5 MHz): -102.6 (1F, s), -105.8 (1F, s), -108.8 (1F, s). **HRMS** (ESI): *m/z* = 587.0078 calcd for $C_{28}H_{21}F_3S_2Ag$ $[M+Ag]^+$ = 587.0079 (Δ = 0.2 ppm).

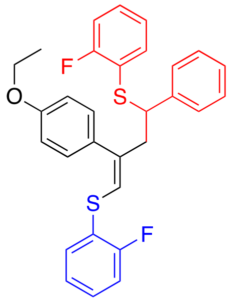

(Z)-(2-(4-ethoxyphenyl)-4-phenylbut-1-ene-1,4-diyl)bis((2-fluorophenyl)sulfane) (4aec) containing 17% **(E)-(2-(4-ethoxyphenyl)-4-phenylbut-1-ene-1,4-diyl)bis((2-fluorophenyl)sulfane)**
^{1}H NMR ($CDCl_3$, 300 MHz): 7.25-6.78 (17H, m), 5.99 (1H, s), 4.20 (1H, dd, J = 9.0, 6.3 Hz), 4.07 (2H, q, J = 6.9 Hz), 3.18 (1H, dd, J = 14.2, 6.0 Hz), 3.08 (1H, dd, J = 14.2, 9.1 Hz), 1.44 (3H, t, J = 7.0 Hz). **^{13}C{^{1}H} NMR** ($CDCl_3$, 75 MHz): 162.5 (d, J(C-F) = 246.2 Hz), 160.1 (d, J(C-F) = 246.0 Hz), 158.7, 140.9, 140.7, 135.3 (d, J(C-F) = 1.2 Hz), 130.3 (d, J(C-F) = 1.5 Hz), 130.1, 129.7 (d, J(C-F) = 8.0 Hz), 129.5, 128.5, 128.1, 127.8 (d, J(C-F) = 7.4 Hz), 127.5, 124.7 (d, J(C-F) = 3.6 Hz), 124.32 (d, J(C-F) = 3.8 Hz), 124.27 (d, J(C-F) = 16.9 Hz), 121.6 (d, J(C-F) = 17.7 Hz), 120.4 (d, J(C-F) = 1.0 Hz), 115.7 (d, J(C-F) = 22.9 Hz), 115.5 (d, J(C-F) = 21.6 Hz), 114.4, 63.5, 50.6 (d, J(C-F) = 2.2 Hz), 45.8, 15.0. **^{19}F{^{1}H} NMR** ($CDCl_3$, 283 MHz): -102.6 (1F, s), -106.1 (1F, s). **HRMS** (ESI): *m/z* = 543.1031 calcd for $C_{30}H_{26}S_2F_2OK$ $[M+K]^+$ = 543.1025 (Δ = 1.1 ppm).

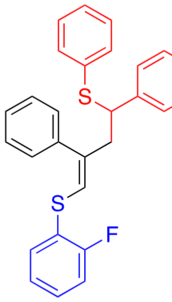

(Z)-(2,4-diphenyl-4-(phenylthio)but-1-en-1-yl)(2-fluorophenyl)sulfane (4aad), containing 8% **4bad**.
^{1}H NMR ($CDCl_3$, 300 MHz): 7.41-7.27 (4H, m), 7.24-7.09 (12H, m), 7.06-6.83 (3H, m), 6.02 (1H, s), 4.08 (1H, dd, J = 9.3, 6.0 Hz), 3.21 (1H, ddd, J = 14.2, 6.0, 0.9 Hz), 3.08 (1H, dd, J = 14.2, 9.3 Hz). **^{13}C{^{1}H} NMR** ($CDCl_3$, 75 MHz): 160.2 (d, J(C-F) = 245.9 Hz), 141.3, 141.0, 138.3, 134.7, 132.5, 130.5 (d, 1.4 Hz), 128.8, 128.6, 128.5, 128.4, 128.2, 128.0, 127.9 (d, J(C-F) = 7.8 Hz), 127.4, 127.3, 124.7 (d, J(C-F) = 3.6 Hz), 124.1 (d, J(C-F) = 17.1 Hz), 121.3 (d, J(C-F) = 1.5 Hz), 115.5 (d, J(C-F) = 21.7 Hz), 51.4, 45.7. **^{19}F{^{1}H} NMR** ($CDCl_3$, 283 MHz): -106.0 (1F, s). **HRMS** (ESI): *m/z* = 443.1292 calcd for $C_{28}H_{24}FS_2$ $[M+H]^+$ = 443.1298 (Δ = 1.4 ppm).

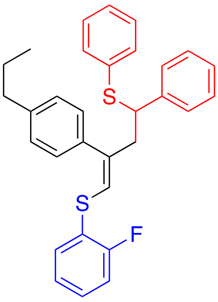

(Z)-(2-fluorophenyl)(4-phenyl-4-(phenylthio)-2-(4-propylphenyl)but-1-en-1-yl)sulfane (4abd), containing 13% **4bbd.**
^{1}H NMR ($CDCl_3$, 300 MHz): 7.32-7.27 (1H, m), 7.25-7.08 (14H, m), 7.05-6.92 (2H, m), 6.91-6.82 (1H, m), 5.96 (1H, s), 4.10 (1H, dd, J = 9.4, 5.9 Hz), 3.20 (1H, dd, J = 14.2, 5.9 Hz), 3.07 (1H, dd, J = 14.1, 9.4 Hz), 2.66-2.57 (2H, m), 1.76-1.61 (2H, sext, J = 7.3 Hz), 0.98 (3H, t, J = 7.3 Hz). **^{13}C{^{1}H} NMR** ($CDCl_3$, 75 MHz): 160.1 (d, J(C-F) = 245.6 Hz), 142.5, 141.4, 141.1, 135.4, 134.8, 132.4, 130.4 (d, J(C-F) = 1.6 Hz), 128.8, 128.6, 128.5, 128.24, 128.18, 127.8 (d, J(C-F) = 7.6 Hz), 127.4, 127.2, 124.7 (d, J(C-F) = 3.6 Hz), 124.3 (d, J(C-F) = 17.2 Hz), 120.7 (d, J(C-F) = 1.4 Hz), 115.5 (d, J(C-F) = 21.7 Hz), 51.4, 45.7, 38.0, 24.5, 14.1. **^{19}F{^{1}H} NMR** ($CDCl_3$, 283 MHz): -106.1 (1F, s). **HRMS** (ESI): *m/z* = 485.1751 calcd for $C_{31}H_{30}FS_2$ $[M+H]^+$ = 485.1767 (Δ = 3.3 ppm).

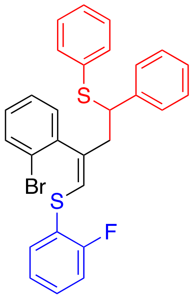

(Z)-(3-(2-bromophenyl)-4-((2-fluorophenyl)thio)-1-phenylbut-3-en-1-yl)(phenyl)sulfane (4acd) containing 15% **4bcd**.
^{1}H NMR (DMSO-d_6, 400 MHz, 373 K): 7.68-7.61 (1H, m), 7.39-7.08 (16H, m), 7.07-7.00 (1H, m), 6.38 (1H, s), 4.38 (1H, dd, J = 8.7, 6.3 Hz), 3.22 (1H, dd, J = 15.0, 8.9 Hz), 3.15 (1H, ddd, J = 15.1, 6.2, 1.1 Hz). **^{13}C{^{1}H} NMR** (DMSO-d_6, 100 MHz, 373 K): 158.9 (d, J(C-F) = 244.0 Hz), 140.4, 140.2, 138.8, 133.9, 132.2, 131.0, 130.1, 129.9, 129.0, 128.2, 127.9 (d, J(C-F) = 7.7 Hz), 127.7, 127.5, 127.0, 126.6, 126.5, 124.5 (d, J(C-F) =3.7 Hz), 122.2 (d, J(C-F) = 1.5 Hz), 121.8 (d, J(C-F) = 17.0 Hz), 121.2, 114.9 (d, J(C-F) = 21.6 Hz),

49.8, 42.5. **^{19}F NMR** (DMSO-d_6, 376 MHz, 373 K): -111.3 (1F, m). **HRMS** (ESI): *m/z* = 521.0392 calcd for $C_{28}H_{23}FS_2Br$ $[M+H]^+$ = 521.0403 (Δ = 2.1 ppm).

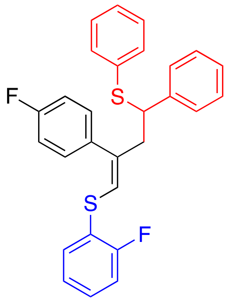

(Z)-(2-fluorophenyl)(2-(4-fluorophenyl)-4-phenyl-4-(phenylthio)but-1-en-1-yl)sulfane (4add), containing 15% **4bdd**.
^{1}H NMR ($CDCl_3$, 300 MHz): 7.31-7.27 (1, m), 7.26-7.12 (12H, m), 7.09-7.00 (3H, m), 7.00-6.95 (1H, m), 6.94-6.87 (1H, m), 6.03 (1H, s), 4.04 (1H, dd, J = 9.2, 6.2 Hz), 3.17 (1H, ddd, J = 14.2, 6.2, 1.0 Hz), 3.06 (1H, dd, J = 14.2, 9.2 Hz). **^{13}C{^{1}H} NMR** ($CDCl_3$, 75 MHz): 162.4 (d, J(C-F) = 247.3 Hz), 160.3 (d, J(C-F) = 246.0 Hz), 140.9, 140.2, 134.6, 134.1 (d, J(C-F) = 3.3 Hz), 132.7, 130.6 (d, J(C-F) = 1.2 Hz), 130.1 (d, J(C-F) = 8.1 Hz), 128.9, 128.6, 128.21, 128.15 (d, J(C-F) = 7.7 Hz), 127.5, 127.4, 124.8 (d, J(C-F) = 3.8 Hz), 123.7 (d, J(C-F) = 17.3 Hz), 121.8, 115.6 (d, J(C-F) = 21.7 Hz), 115.6 (d, J(C-F) = 21.4 Hz), 51.6, 45.7. **^{19}F{^{1}H} NMR** ($CDCl_3$, 283 MHz): -105.8 (1F, s), -108.7 (1F, s). **HRMS** (ESI): *m/z* = 461.1199 calcd for $C_{28}H_{23}F_2S_2$ $[M+H]^+$ = 461.1204 (Δ = 1.1 ppm).

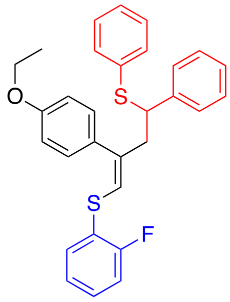

(Z)-(3-(4-ethoxyphenyl)-4-((2-fluorophenyl)thio)-1-phenylbut-3-en-1-yl)(phenyl)sulfane (4aed) containing 18% **4bed**
^{1}H NMR ($CDCl_3$, 300 MHz): 7.26-7.08 (13H, m), 7.07-6.93 (2H, m), 6.92-6.85 (3H, m), 5.94 (1H, s), 4.12-4.02 (3H, m), 3.20 (1H, dd, J = 14.1, 5.7 Hz), 3.06 (1H, dd, J = 14.1, 9.4 Hz), 1.45 (3H, t, J = 7.0 Hz). **^{13}C{^{1}H}** NMR ($CDCl_3$, 75 MHz): 160.1 (d, J(C-F) = 245.6 Hz), 158.7, 141.3, 141.0, 134.8, 132.5, 130.2 (d, J(C-F) = 1.8 Hz), 130.1, 129.6, 128.8, 128.6, 128.2, 127.7 (d, J(C-F) = 8.0 Hz), 127.4, 127.2, 124.7 (d, J(C-F) = 3.8 Hz), 124.3 (d, J(C-F) = 17.3 Hz), 120.2, 115.5 (d, J(C-F) = 21.8 Hz), 114.3, 63.5, 51.5, 45.8, 15.0. **^{19}F{^{1}H}** NMR ($CDCl_3$, 283 MHz): -106.2 (1F, s). **HRMS** (ESI): *m/z* = 525.1112 calcd for $C_{30}H_{27}FOS_2K$ $[M+K]^+$ = 525.1119 (Δ = 1.5 ppm)

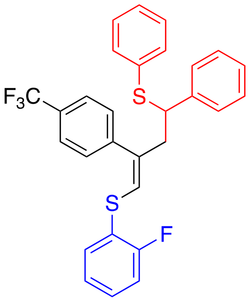

(Z)-(2-fluorophenyl)(4-phenyl-4-(phenylthio)-2-(4-(trifluoromethyl)phenyl)but-1-en-1-yl)sulfane (4afd), containing 5% **4bfd**.
^{1}H NMR ($CDCl_3$, 300 MHz): 7.60 (2H, d, J = 8.1 Hz), 7.33-7.27 (3H, m), 7.25-7.13 (10H, m), 7.05-6.97 (2H, m), 6.96-6.88 (1H, m), 6.11 (1H, s), 4.02 (1H, dd, J = 9.2, 6.2 Hz), 3.20 (1H, dd, J = 14.3, 6.2 Hz), 3.10 (1H, dd, J = 14.3, 9.2 Hz). **^{13}C{^{1}H} NMR** ($CDCl_3$, 75 MHz): 160.4 (d, J(C-F) = 246.2 Hz), 142.1 (q, J(C-F) = 1.4 Hz), 140.7, 139.3, 134.4, 132.80, 132.75, 130.9 (d, J(C-F) = 1.7 Hz), 129.9 (q J(C-F) = 32.5 Hz), 128.9, 128.8, 128.7, 128.5 (d, J(C-F) = 7.7 Hz), 128.2, 127.6 (d, J(C-F) = 2.8 Hz), 125.5 (q, J(C-F) = 3.8 Hz), 124.8 (d, J(C-F) = 3.7 Hz), 124,2 (q J(C-F) = 272.0 Hz), 123.45 (d, J(C-F) = 1.4 Hz), 123.2 (d, J(C-F) = 17.2 Hz), 115.7 (d, J(C-F) = 22.0 Hz), 51.7, 45.4. **^{19}F{^{1}H} NMR** ($CDCl_3$, 283 MHz): -57.6 (3F, s), -105.5 (1F, s). **HRMS** (ESI): *m/z* = 619.0141 calcd for $C_{29}H_{22}F_4S_2Ag$ $[M+Ag]^+$ = 619.0141 (Δ = 0.0 ppm).

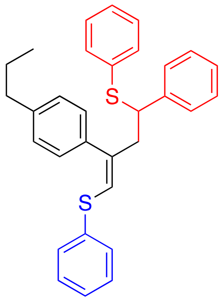

(Z)-(4-phenyl-2-(4-propylphenyl)but-1-ene-1,4-diyl)bis(phenylsulfane) (4bbd).
^{1}H NMR ($CDCl_3$, 300 MHz): 7.32-7.27 (1H, m), 7.26-7.09 (16H, m), 7.05-6.99 (2H, m), 6.05 (1H, s), 4.11 (1H, dd, J = 9.4, 5.9 Hz), 3.20 (1H, dd J = 14.2, 5.8 Hz), 3.06 (1H, dd, J = 14.2, 9.4 Hz), 2.61 (2H, t, J = 7.7 Hz), 1.76-1.61 (2H, sext, J = 7.5 Hz), 0.98 (3H, t, J = 7.3 Hz). **^{13}C{^{1}H} NMR** ($CDCl_3$, 75 MHz): 142.4, 141.2, 139.7, 137.1, 135.7, 134.9, 132.4, 129.0, 128.8, 128.58, 128.55, 128.5, 128.3, 128.2, 127.4, 127.2, 126.1, 122.6, 51.5, 45.7, 38.0, 24.5, 14.1. **HRMS** (ESI): *m/z* = 467.1856 calcd for $C_{31}H_{31}S_2$ $[M+H]^+$ =

467.1862 (Δ = 1.3 ppm).

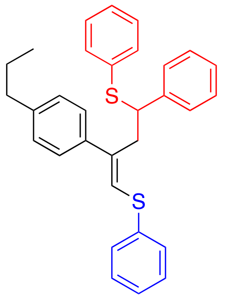

(E)-(4-phenyl-2-(4-propylphenyl)but-1-ene-1,4-diyl)bis(phenylsulfane) (E-4bbd).
^{1}H NMR ($CDCl_3$, 300 MHz): 7.25-7.15 (15H, m), 7.12-7.05 (4H, m), 6.32 (1H, s), 4.25 (1H, dd, J = 8.6, 6.9 Hz), 3.46-3.29 (2H, m), 2.63-2.55 (2H, m), 1.73-1.59 (2H, sext, J = 7.5 Hz), 0.96 (3H, t, J = 7.3 Hz). **^{13}C{^{1}H} NMR** ($CDCl_3$, 75 MHz): 142.2, 141.0, 140.7, 138.0, 136.7, 136.5, 135.2, 132.6, 129.0, 128.8, 128.7, 128.6, 128.3, 128.2, 127.4, 127.2, 126.4, 123.2, 52.0, 38.7, 37.8, 24.6, 14.0. **HRMS** (ESI): *m/z* = 505.1440 calcd for $C_{31}H_{30}S_2K$ $[M+K]^+$ = 505.1421 (Δ = 3.9 ppm).

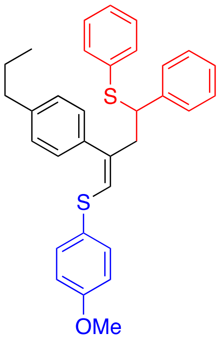

(Z)-(4-methoxyphenyl)(4-phenyl-4-(phenylthio)-2-(4-propylphenyl)but-1-en-1-yl)sulfane (4cbd).
^{1}H NMR ($CDCl_3$, 300 MHz): 7.30-7.24 (3H, m), 7.23-7.16 (11H, m), 7.07 (2H, d, J = 8.7 Hz), 6.80 (2H, d, J = 8.8 Hz), 6.01 (1H, s), 4.13 (1H, dd, J = 9.2, 6.0 Hz), 3.80 (3H, s), 3.19 (1H, dd, J = 14.0, 6.2 Hz), 3.04 (1H, dd, J = 14.1, 9.2 Hz), 2.69-2.06 (2H, m), 1.80-1.64 (2H, sext, 7.6 Hz), 1.01 (3H, t, J = 7.3 Hz). **^{13}C{^{1}H} NMR** ($CDCl_3$, 75 MHz): 158.9, 142.2, 141.3, 137.2, 135.8, 135.0, 132.8, 132.4, 131.5, 128.8, 128.5, 128.24, 128.18, 127.5, 127.3, 127.1, 124.9, 114.7, 55.5, 51.6, 45.5, 38.0, 24.5, 14.1. **HRMS** (ESI): *m/z* = 605.0936 calcd for $C_{32}H_{32}OS_2Ag$ $[M+Ag]^+$ = 605.0937 (Δ = 0.2 ppm).

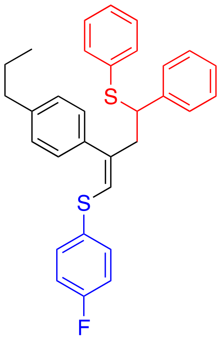

(Z)-(4-fluorophenyl)(4-phenyl-4-(phenylthio)-2-(4-propylphenyl)but-1-en-1-yl)sulfane (4dbd).
^{1}H NMR ($CDCl_3$, 300 MHz): 7.35-7.28 (4H, m), 7.24-7.18 (10H, m), 7.08-7.00 (2H, m), 6.99-6.91 (2H, m), 6.02 (1H, s), 4.16 (1H, dd, J = 9.4, 5.9 Hz), 3.24 (1H, ddd, J = 14.2, 5.9, 1.1 Hz), 3.09 (1H, ddd, J = 14.2, 9.4, 0.7 Hz), 2.67 (2H, t, J = 7.7 Hz), 1.81-1.66 (2H, sext, J = 7.4 Hz), 1.03 (3H, t, J = 7.3 Hz). **^{13}C{^{1}H} NMR** ($CDCl_3$, 75 MHz): 161.8 (d, J(C-F) = 246.0 Hz), 142.5, 141.2, 139.5, 135.6, 134.9, 132.4, 132.1 (d, J(C-F) = 2.9 Hz), 130.9 (d, J(C-F) = 8.0 Hz), 128.8, 128.6, 128.5, 128.3, 128.1, 127.4, 127.2, 123.1, 116.1 (d, J(C-F) = 21.9 Hz), 51.5, 45.6, 38.0, 24.5, 14.1. **^{19}F{^{1}H} NMR** ($CDCl_3$, 282.5 MHz): -111.1 (1F, s). **HRMS** (ESI): *m/z* = 593.0725 calcd for $C_{31}H_{29}FS_2Ag$ $[M+Ag]^+$ = 593.0737 (Δ = 2.0 ppm)

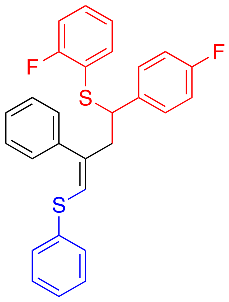

(Z)-(2-fluorophenyl)(1-(4-fluorophenyl)-3-phenyl-4-(phenylthio)but-3-en-1-yl)sulfane (4baa) containing 9% **4aaa**.
^{1}H NMR ($CDCl_3$, 300 MHz): 7.41-7.30 (3H, m), 7.25-7.06 (11H, m), 7.02-6.88 (4H, m), 6.14 (1H, s), 4.20 (1H, dd, J = 9.3, 6.1 Hz), 3.23 (1H, ddd, J = 14.2, 6.0, 0.8 Hz), 3.11 (1H, dd, J = 14.2, 9.3 Hz). **^{13}C{^{1}H} NMR** ($CDCl_3$, 75 MHz): 162.5 (d, J(C-F) = 246.3 Hz), 162.1 (d, J(C-F) = 245.9 Hz), 139.1, 138.3, 136.7, 136.5 (d, J(C-F) = 3.3 Hz), 135.5 (d, J(C-F) = 1.1 Hz), 129.9 (d, J(C-F) = 8.2 Hz), 129.8 (d, J(C-F) = 8.1 Hz), 129.0, 128.8, 128.5, 128.3, 127.9, 126.4, 124.4 (d, J(C-F) = 3.8 Hz), 123.7, 121.2 (d, J(C-F) = 18.5 Hz), 115.8 (d, J(C-F) = 22.9 Hz), 115.4 (d, J(C-F) = 21.5 Hz), 49.9 (d, J(C-F) = 2.2 Hz), 45.7. **^{19}F{^{1}H} NMR** ($CDCl_3$, 282.5 MHz): -102.6 (1F, s), -109.9 (1F, s). **HRMS** (ESI): *m/z* = 569.0173 calcd for $C_{28}H_{22}F_2S_2Ag$ $[M+Ag]^+$ = 569.0173 (Δ = 0.0 ppm).

(Z)-(2-fluorophenyl)(3-phenyl-4-(phenylthio)-1-(4-propylphenyl)but-3-en-1-yl)sulfane (4bab) containing 6% **4aab**.

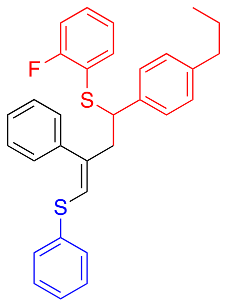

^{1}H NMR ($CDCl_3$, 300 MHz): 7.40-7.29 (3H, m), 7.23-7.02 (13H, m), 7.02-6.86 (2H, m), 6.15 (1H, s), 4.19 (1H, dd, J = 8.8, 6.5 Hz), 3.23-3.03 (2H, m), 2.60-2.51 (2H, m), 1.69-1.54 (2H, sext, J = 7.5 Hz), 0.91 (3H, t, J = 7.3 Hz). **^{13}C{^{1}H} NMR** ($CDCl_3$, 75 MHz): 162.4 (d, J(C-F) = 245.8 Hz), 142.0, 139.4, 138.6, 137.8, 136.9, 135.2 (d, J(C-F) = 1.1 Hz), 129.5 (d, J(C-F) = 7.6 Hz), 129.0, 128.8, 128.6, 128.4, 128.3, 128.0, 127.8, 126.2, 124.3 (d, J(C-F) = 3.8 Hz), 123.3, 121.9 (d, J(C-F) = 18.0 Hz), 115.7 (d, J(C-F) = 22.9 Hz), 50.4 (d, J(C-F) = 2.2 Hz), 45.6, 37.8, 24.6, 13.9. **^{19}F{^{1}H} NMR** ($CDCl_3$, 283 MHz): -103.1 (1F, s). **HRMS** (ESI): *m/z* = 593.0723 calcd for $C_{31}H_{29}FS_2Ag$ $[M+Ag]^+$ = 593.0737 (Δ = 2.4 ppm).

(Z)-(2,4-diphenylbut-1-ene-1,4-diyl)bis(phenylsulfane) (4bad).

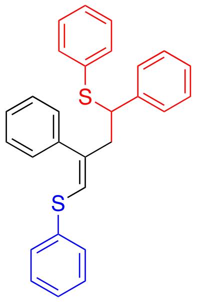

^{1}H NMR ($CDCl_3$, 300 MHz): 7.41-7.27 (5H, m), 7.25-7.13 (13H, m), 7.07-7.00 (2H, m), 6.10 (1H, s), 4.09 (1H, dd, J = 9.3, 5.9 Hz), 3.21 (1H, ddd, J = 14.2, 5.9, 6.0 Hz), 3.07 (1H, dd, J = 13.9, 9.2 Hz). **^{13}C{^{1}H} NMR** ($CDCl_3$, 75 MHz): 141.1, 139.7, 138.5, 136.9, 134.8, 132.5, 129.0, 128.8, 128.63, 128.59, 128.5, 128.4, 128.3, 127.9, 127.4, 127.3, 126.2, 123.2, 51.5, 45.6. **HRMS** (ESI): *m/z* = 533.0359 calcd for $C_{28}H_{24}S_2Ag$ $[M+Ag]^+$ = 533.0361 (Δ = 0.4 ppm).

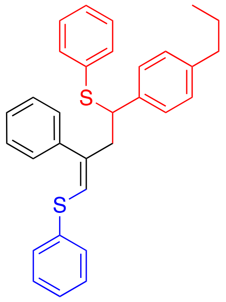

(Z)-(2-phenyl-4-(4-propylphenyl)but-1-ene-1,4-diyl)bis(phenylsulfane) (4bag).

^{1}H NMR ($CDCl_3$, 300 MHz): 7.35-7.27 (3H, m), 7.21-7.16 (4H, m), 7.16-7.12 (6H, m), 7.10-7.03 (6H, m), 6.10 (1H, s), 4.08 (1H, dd, J = 9.2, 6.0 Hz), 3.18 (1H, ddd, J = 14.2, 6.0, 1.0 Hz), 3.05 (1H, dd, J = 14.4, 9.3 Hz), 2.60-2.53 (2H, m), 1.69-1.56 (2H, sext, J = 7.6 Hz), 0.92 (3H, t, J = 7.4 Hz). **^{13}C{^{1}H} NMR** ($CDCl_3$, 75 MHz): 141.8, 139.7, 138.6, 138.2, 137.0, 135.1, 132.4, 129.0, 128.8, 128.7, 128.6, 128.4, 128.4, 128.1, 127.8, 127.1, 126.2, 123.1, 51.2, 45.6, 37.8, 24.6, 13.9. **HRMS** (ESI): *m/z* = 467.1862 calcd for $C_{31}H_{30}S_2H$ $[M+H]^+$ = 467.1862 (Δ = 0.0 ppm).

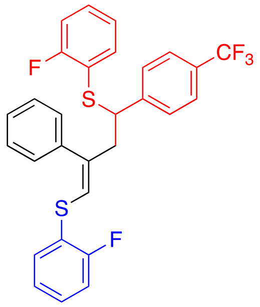

(Z)-(2-phenyl-4-(4-(trifluoromethyl)phenyl)but-1-ene-1,4-diyl)bis((2-fluorophenyl)sulfane) (4aae).

^{1}H NMR (Acetone-d_6, 300 MHz): 7.61 (2H, d, 8.1 Hz), 7.48 (2H, d, 8.1 Hz), 7.42-7.34 (3H, m), 7.31-7.22 (5H, m), 7.15-6.98 (5H, m), 6.32 (1H, s), 4.39 (1H, t, J = 7.8 Hz), 3.32 (2H, d, J = 7.7 Hz).

^{13}C{^{1}H} NMR (Acetone-d_6, 75 MHz): 163.0 (d, J(C-F) = 244.9 Hz), 160.8 (d, J(C-F) = 243.9 Hz), 146.4 (d, J(C-F) = 1.4 Hz), 141.3, 139.0, 135.9 (d, J(C-F) = 1.4 Hz), 131.2 (d, J(C-F) = 1.7 Hz), 131.1 (d, J(C-F) = 8.0 Hz), 129.77 (q J(C-F) = 31.2 Hz), 129.75, 129.31 (d, J(C-F) = 7.8 Hz), 129.27, 129.0, 128.8, 126.1 (q, J(C-F) = 3.9 Hz), 125.8 (d, J(C-F) = 3.6 Hz), 125.5 (d, J(C-F) = 3.7 Hz), 125.3 (q, J(C-F) = 271.0 Hz), 124.2 (d, J(C-F) = 17.3 Hz), 122.3 (d, J(C-F) = 1.0 Hz), 121.6 (d, J(C-F) = 18.0 Hz), 116.5 (d, J(C-F) = 23.0 Hz), 116.3 (d, J(C-F) = 21.7 Hz), 50.7 (d, J(C-F) = 1.6 Hz), 45.4. **^{19}F{^{1}H} NMR** ($CDCl_3$, 283 MHz): -57.5 (3F, s), -102.4 (1F, s), -105.4 (1F, s). **HRMS** (ESI): *m/z* = 529.1058 calcd for $C_{29}H_{22}S_2F_5$ $[M+H]^+$ 529.1078 (Δ = 3.8 ppm).

(Z)-(4-(4-ethoxyphenyl)-2-phenylbut-1-ene-1,4-diyl)bis((2-fluorophenyl)sulfane) (4aaf) containing 5% **(E)-(4-(4-ethoxyphenyl)-2-phenylbut-1-ene-1,4-diyl)bis((2-fluorophenyl)sulfane)**.

^{1}H NMR ($CDCl_3$, 300 MHz): 7.39-7.30 (3H, m), 7.21-7.05 (7H, m), 7.03-6.87 (5H, m), 6.80-6.73 (2H, d, J = 8.6 Hz), 6.03 (1H, s), 4.17 (1H, dd, J = 9.3, 6.1 Hz), 4.00 (2H, q, J = 7.0 Hz),

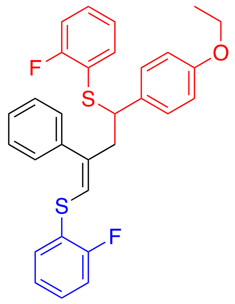

3.16 (1H, dd, J = 14.3, 5.9 Hz), 3.06 (1H, dd, J = 14.1, 9.4 Hz), 1.41 (3H, t, J = 7.0 Hz). **^{13}C{^{1}H} NMR** ($CDCl_3$, 75 MHz): 162.4 (d, J(C-F) = 246.0 Hz), 160.1 (d, J(C-F) = 245.4 Hz), 158.4, 141.4, 138.3, 135.2 (d, J(C-F) = 1.6 Hz), 132.3, 130.5 (d, J(C-F) = 1.9 Hz), 129.6 (d, J(C-F) = 8.0 Hz), 129.2, 128.5, 128.3, 128.0, 127.9 (d, J(C-F) = 7.6 Hz), 124.6 (d, J(C-F) = 3.8 Hz), 124.3 (d, J(C-F) = 3.8 Hz), 124.1 (d, J(C-F) = 16.7 Hz), 121.7 (d, J(C-F) = 18.5 Hz), 121.3 (d, J(C-F) = 1.7 Hz), 115.7 (d, J(C-F) = 23.4 Hz), 115.5 (d, J(C-F) = 21.7 Hz), 114.5, 63.5, 49.9 (d, J(C-F) = 2.2 Hz), 45.8, 15.0. **^{19}F{^{1}H} NMR** ($CDCl_3$, 283 MHz): -102.8 (1F, s), -106.1 (1F, s). **HRMS** (ESI): *m/z* = 613.0439 calcd for $C_{31}H_{28}F_2S_2Ag$ $[M+Ag]^+$ = 613.0435 (Δ = 0.7 ppm).

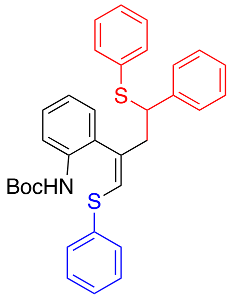

tert-butyl (Z)-(2-(4-phenyl-1,4-bis(phenylthio)but-1-en-2-yl)phenyl)carbamate (4bhd).

^{1}H NMR (DMSO-d_6, 400 MHz, 373 K): 7.80 (1H, d, J = 8.2 Hz), 7.32-7.25 (8H m), 7.21-7.10 (9H, m), 7.07 (2H, d, J = 4 Hz), 6.53 (1H, s), 4.29 (1H, t, J = 7.7 Hz), 3.20 (1H, ddd, J = 14.9, 8.2, 0.9 Hz), 3.13 (1H, ddd, J = 14.9, 7.3, 1.0 Hz), 1.36 (9H, s). **^{13}C{^{1}H} NMR** (DMSO-d_6, 100 MHz, 373 K): 152.0, 140.4, 135.3, 134.9, 134.8, 133.7, 130.9, 128.9, 128.4, 128.1, 128.0, 127.8, 127.7, 127.5, 127.3, 126.6, 126.4, 125.8, 125.0, 122.6, 120.6, 78.7, 50.0, 43.6, 27.4. **HRMS** (ESI): *m/z* = 648.0994 calcd for $C_{30}H_{26}F_3NS_2Ag$ $[M+Ag]^+$ = 648.0995 (Δ = 0.1 ppm).

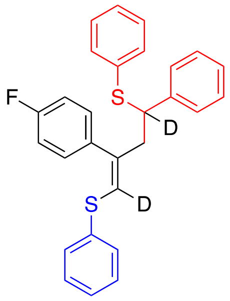

(*Z*)-(2-(4-fluorophenyl)-4-phenylbut-1-ene-1,4-diyl-1,4-*d*2)bis(phenylsulfane) (D-4bdd).

^{1}H NMR ($CDCl_3$, 300 MHz): 7.33-7.25 (4H, m), 7.22-7.15 (11H, m), 7.10-7.02 (4H, m), 6.13 (0.66H, s), 4.07 (0.08H, dd, J = 9.3, 6.1 Hz), 3.19 (1H, d, J = 14.2 Hz), 3.07 (1H, d, J = 14.2 Hz). **^{13}C{^{1}H}** NMR ($CDCl_3$, 75 MHz, 323 K): 162.2 (d, J(C-F) = 247.1 Hz), 140.9, 138.7, 136.6, 134.7, 134.4 (d, J(C-F) = 3.3 Hz), 132.6, 130.1 (d, J(C-F) = 7.6 Hz), 129.1, 128.8, 128.7, 128.6, 128.2, 127.5, 127.4, 126.3, 123.5, 115.4 (d, J(C-F) = 21.8 Hz), 51.2 (t, J(C-D) = 21.0 Hz), 45.6. **^{19}F{^{1}H}** NMR ($CDCl_3$, 282.5 MHz): -108.9 (1F, s). **^{2}H NMR** (Acetone-H_6, 46 MHz): 6.3 (0.3D, s), 4.24 (1D, s). **HRMS** (ESI): *m/z* = 552.0343 calcd for $C_{28}H_{22}D_1FS_2Ag$ = 552.0330 (Δ = 2.5 ppm), *m/z* = 553.0383calcd for $C_{28}H_{21}D_2FS_2Ag$ $[M+Ag]^+$ = 553.0393 (Δ = 1.8 ppm).

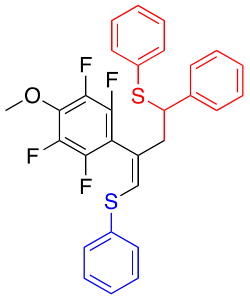

(Z)-(4-phenyl-2-(2,3,5,6-tetrafluoro-4-methoxyphenyl)but-1-ene-1,4-diyl)bis(phenylsulfane) (4bid).

^{1}H NMR ($CDCl_3$, 300 MHz): 7.25-7.16 (13H, m), 7.09-7.02 (2H, m), 6.36 (1H, s), 4.13 (1H, dd, J = 9.3, 5.5 Hz), 4.05 (3H, s), 3.20-3.01 (2H, m). **^{13}C{^{1}H} NMR** ($CDCl_3$, 75 MHz): 145.8-145.4 (m), 142.8-142.1 (m, first components of 2 C-F doublets), 140.4, 139.5-139.1 (m, second components of 2 C-F doublets), 138.2-137.7 (m), 135.0, 134.3, 132.9, 130.7, 129.4, 129.1, 128.9, 128.4, 128.2, 127.7, 127.5, 126.9, 126.6, 62.2 (t, J(C-F) = 3.7 Hz), 52.3, 43.6. **^{19}F{^{1}H} NMR** ($CDCl_3$, 283 MHz): -135.0: -135.7 (2F, m), -153.1 (2F, dd, J = 22.7, 8.7 Hz). **HRMS** (ESI): *m/z* = 635.0092 calcd for $C_{29}H_{22}S_2F_4OAg$ $[M+Ag]^+$ = 635.0090 (Δ = 0.3 ppm).

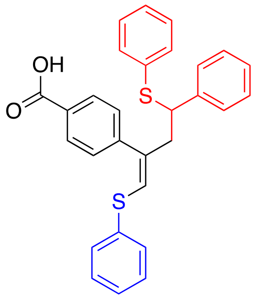

(Z)-4-(4-phenyl-1,4-bis(phenylthio)but-1-en-2-yl)benzoic acid (4bjd).

^{1}H NMR ($CDCl_3$, 300 MHz): 8.14-8.09 (2H, m), 7.36-7.28 (4, m), 7.24-7.14 (11H, m), 7.09-7.03 (2H, m), 6.21 (1H, s), 4.04 (1H, dd, J = 9.2, 6.1 Hz), 3.24 (1H, dd, J = 14.2, 5.9 Hz), 3.11 (1H, dd, J = 14.3, 9.4 Hz).

^{13}C{^{1}H} NMR ($CDCl_3$, 75 MHz): 172.0, 144.4, 140.8, 138.1, 136.3, 134.5, 132.7, 130.4, 129.1, 128.93, 128.90, 128.7, 128.6, 128.5, 128.2, 127.6, 127.5, 126.6, 125.3, 51.8, 45.4. **HRMS** (ESI): *m/z* = 577.0263 calcd for $C_{29}H_{24}O_2S_2Ag$ $[M+Ag]^+$ = 577.0260 (Δ = 0.5 ppm).

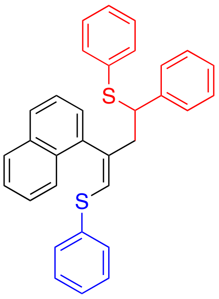

(Z)-(2-(naphthalen-1-yl)-4-phenylbut-1-ene-1,4-diyl)bis(phenylsulfane) (4bkd).
^{1}H NMR (DMSO-d_6, 400 MHz): 7.93 (1H, d, J = 8.1 Hz), 7.88 (1H, d, J = 8.2 Hz), 7.72 (1H, d, J = 8.3 Hz), 7.52-7.46 (2H, m), 7.45-7.37 (1H, m), 7.31-7.17 (9H, m), 7.17-7.07 (7H, m), 7.57-7.00 (19H, m), 6.63 (1H, s), 4.35 (1H, dd, J = 8.4, 6.5 Hz), 3.36-3.18 (2H, m). **^{13}C{^{1}H} NMR** (DMSO-d_6, 100 MHz): 140.4, 137.9, 136.4, 135.3, 134.0, 133.0, 130.8, 129.6, 128.5, 128.1, 127.8, 127.7, 127.6, 127.5, 127.3, 126.6, 126.3, 125.6, 125.5, 125.28, 125.26, 124.8, 124.4, 123.9, 50.1, 44.3. **HRMS** (ESI): *m/z* = 583.0524 calcd for $C_{32}H_{26}S_2Ag$ $[M+Ag]^+$ = 583.0518 (Δ = 1.0 ppm).

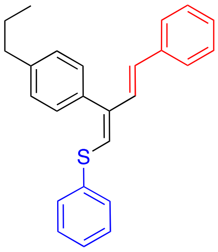

Phenyl((1Z,3E)-4-phenyl-2-(4-propylphenyl)buta-1,3-dien-1-yl)sulfane (6bbd) containing 17% **phenyl((1E,3E)-4-phenyl-2-(4-propylphenyl)buta-1,3-dien-1-yl)sulfane.**
^{1}H NMR ($CDCl_3$, 300 MHz): 7.47-7.29 (12H, m (**1Z, 3E**); 12H*0.2, m (**1E, 3E**)), 7.27-7.20 (2H, m (**1Z, 3E**)); 2H*0.2, m (**1E, 3E**)), 7.08 (1H, d, J = 15.8 Hz (**1Z, 3E**)), 6.70 (1H, s (**1Z, 3E**)), 6.62 (1*0.21H, d, J = 16.0 Hz (**1E, 3E**)), 6.42 (1*0.18H, s (**1E, 3E**)) 6.22 (1H, d, J = 15.8 Hz (**1Z, 3E**)), 2.74-2.66 (2H, m), 1.84-1.70 (2H, sext, J = 7.5 Hz), 1.04 (3H, t, J = 7.3 Hz). **^{13}C{^{1}H} NMR** ($CDCl_3$, 75 MHz): 142.5, 140.9, 137.6, 136.4, 134.4, 131.0, 129.6, 129.40, 129.35, 129.2, 128.71, 128.70, 128.2, 127.4, 126.9, 126.5, 38.1, 24.5, 14.1.

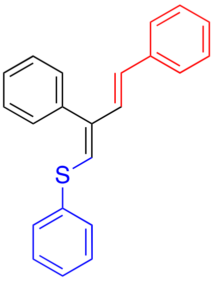

((1Z,3E)-2,4-diphenylbuta-1,3-dien-1-yl)(phenyl)sulfane (6bad), containing 8% **((1E,3E)-2,4-diphenylbuta-1,3-dien-1-yl)(phenyl)sulfane.**
^{1}H NMR ($CDCl_3$, 300 MHz): 7.50-7.45 (2H, m), 7.44-7.37 (4H, m), 7.37-7.31 (6H, m), 7.30-7.27 (2H, m), 7.25-7.23 (1H, m), 7.05 (1H, d, J = 15.9 Hz), 6.68 (1H, s), 6.56 (1*0.09, d, J = 16.0 Hz (**1E, 3E**)), 6.40 (1*0.09, s (**1E, 3E**)) 6.14 (1H, d, J = 15.9 Hz). **^{13}C{^{1}H} NMR** ($CDCl_3$, 75 MHz): 140.9, 137.5, 137.3, 136.3, 130.9, 129.7, 129.6, 129.6, 129.2, 128.7, 128.7, 128.5, 128.0, 127.5, 127.0, 126.5. **HRMS** (ESI): *m/z* = 423.0169 calcd for $C_{22}H_{18}SAg$ $[M+Ag]^+$ = 423.0171 (Δ = 0.5 ppm).

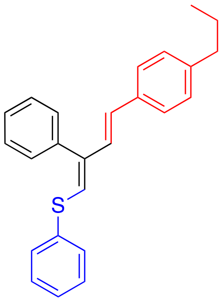

Phenyl((1Z,3E)-2-phenyl-4-(4-propylphenyl)buta-1,3-dien-1-yl)sulfane (6bag) containing 15% **phenyl((1E,3E)-2-phenyl-4-(4-propylphenyl)buta-1,3-dien-1-yl)sulfane**.
^{1}H NMR ($CDCl_3$, 300 MHz): 7.48-7.42 (2H, m), 7.41-7.36 (4H, m), 7.35-7.29 (4H, m), 7.24-7.21 (2H, m), 7.08 (2H, d, J = 7.9 Hz), 7.01 (1H, d, J = 15.8 Hz), 6.63 (1H, s), 6.52 (1H*0.17, d, J = 16.1 Hz (**1E, 3E**)), 6.35 (1H*0.17, s (**1E, 3E**)), 6.11 (1H, d, J = 15.9 Hz), 2.57-2.50 (2H, m), 1.67-1.53 (2H, sext, J = 7.4 Hz), 0.91 (3H, t, J = 7.3 Hz). **^{13}C{^{1}H} NMR** ($CDCl_3$, 75 MHz): 142.2, 141.2, 137.4, 136.4, 134.9, 130.0, 129.6, 129.6, 129.5, 129.2, 128.9, 128.6, 127.9, 127.6, 126.9, 126.4, 37.9, 24.6, 13.9. **HRMS** (ESI): *m/z* = 465.0662 calcd for $C_{25}H_{24}SAg$ $[M+Ag]^+$ = 465.0642 (Δ = 4.3 ppm).

5-phenyl-2-(phenylthio)-3-(4-propylphenyl)furan

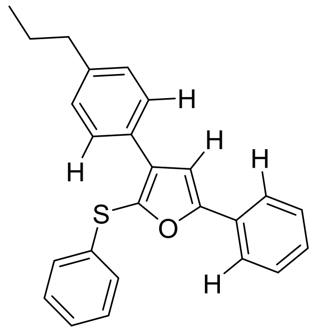

^{1}H NMR (600 MHz, CD_2Cl_2) δ 7.76 – 7.74 (m, 2H), 7.63 (d, *J* = 8.0 Hz, 2H), 7.43 (t, *J* = 7.9 Hz, 2H), 7.35 – 7.31 (m, 1H), 7.28 – 7.24 (m, 2H), 7.24 – 7.21 (m, 2H), 7.21 – 7.19 (m, 2H), 7.18 – 7.15 (m, 1H), 7.06 (s, 1H), 2.63 – 2.59 (m, 2H), 1.67 – 1.63 (m, 2H), 0.95 (t, *J* = 7.3 Hz, 3H). **^{13}C{^{1}H} NMR** (151 MHz, CD_2Cl_2) δ 157.4, 143.3, 137.9, 137.8, 137.5, 135.9, 130.0, 129.8, 129.4, 129.2, 128.9, 128.1, 127.3, 126.7, 124.7, 107.9, 38.3, 25.1, 14.2.

^{19}F NMR spectra of **4aca**, **4acb**, **4acc**, and **4acd** were obtained without referring to external standards due to high temperatures.

Copies of NMR spectra of products

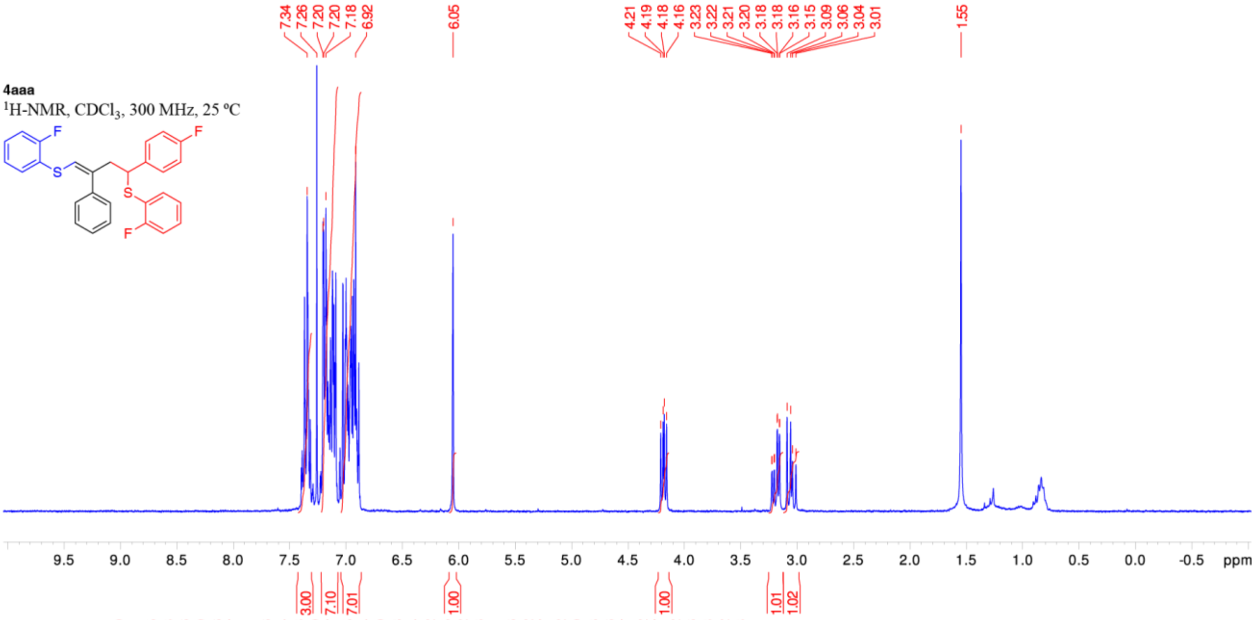

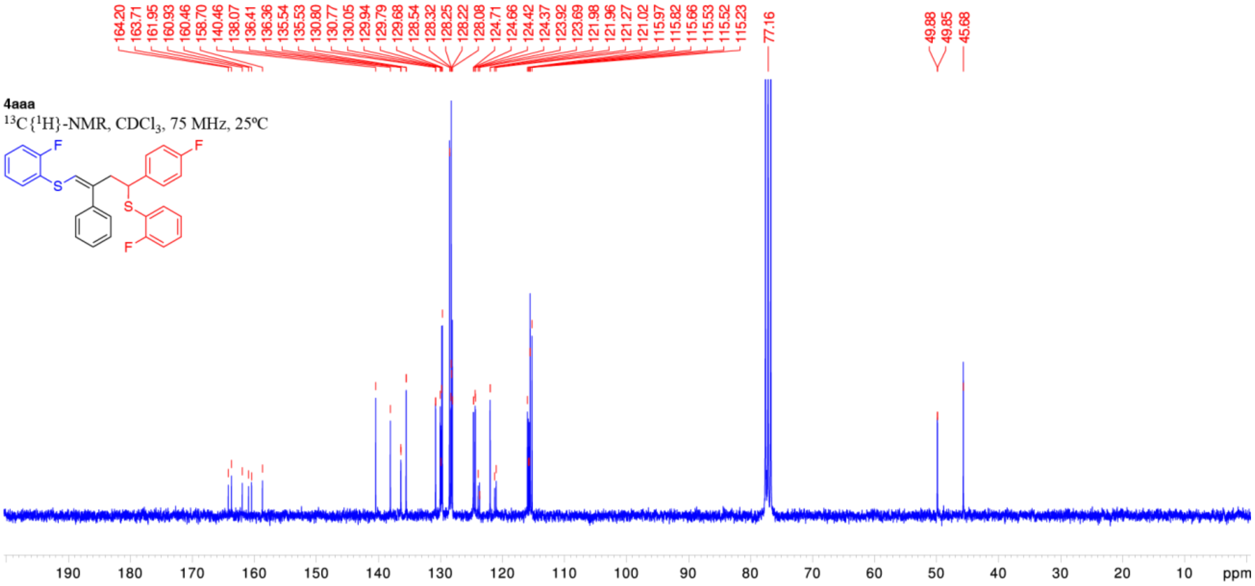

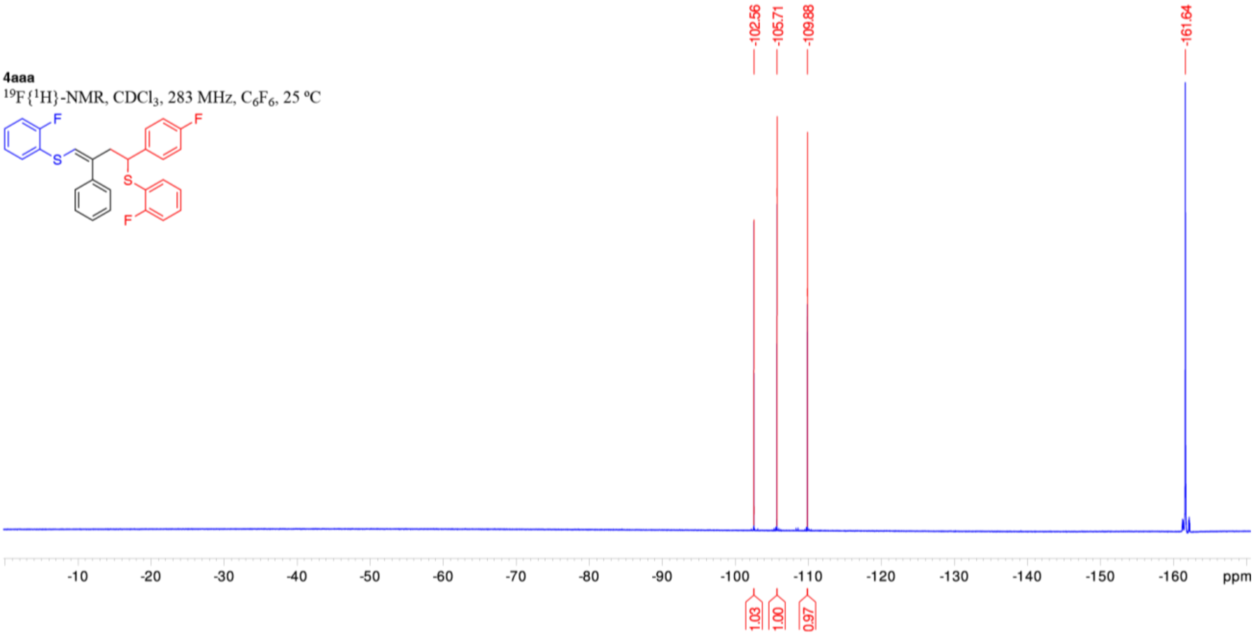

4aba

4aba
^{1}H-NMR, $CDCl_3$, 300 MHz, 25 ºC

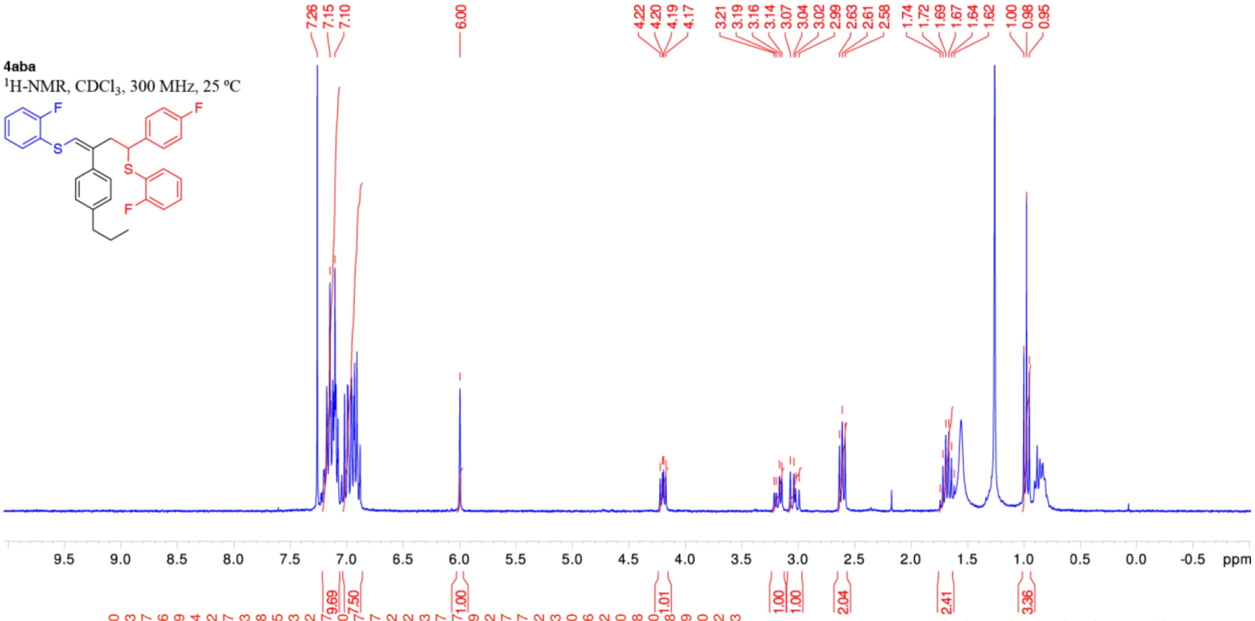

4aba
$^{13}C\{^{1}H\}$-NMR, Acetone-d_6, 75.5 MHz, 25ºC

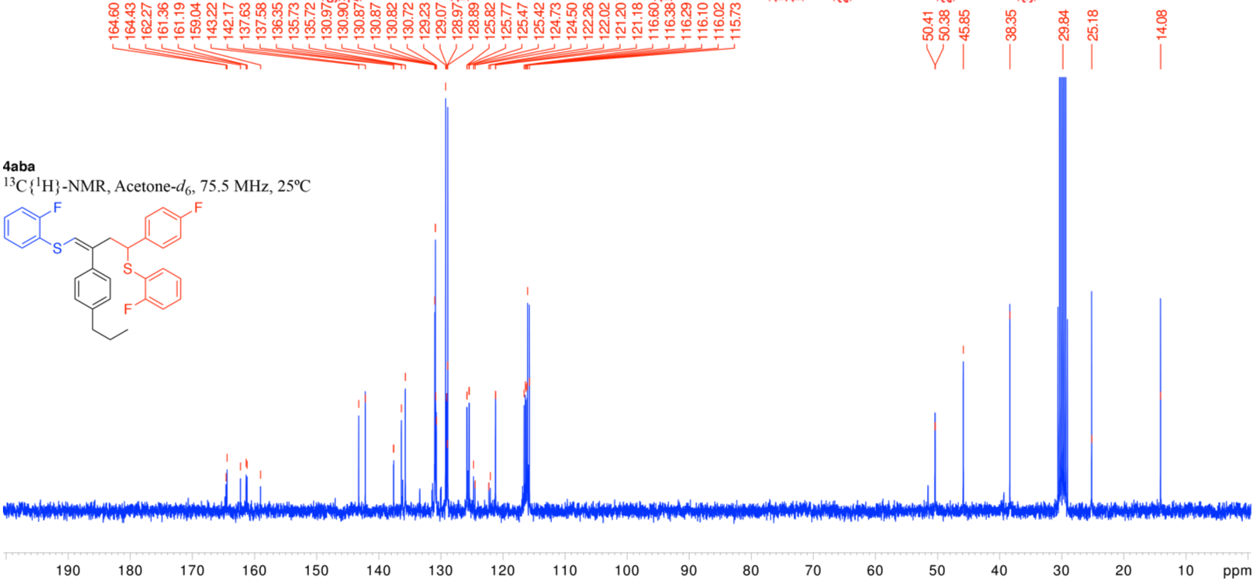

4aba
$^{19}F\{^{1}H\}$-NMR, $CDCl_3$, 283 MHz, C_6F_6, 25 ºC

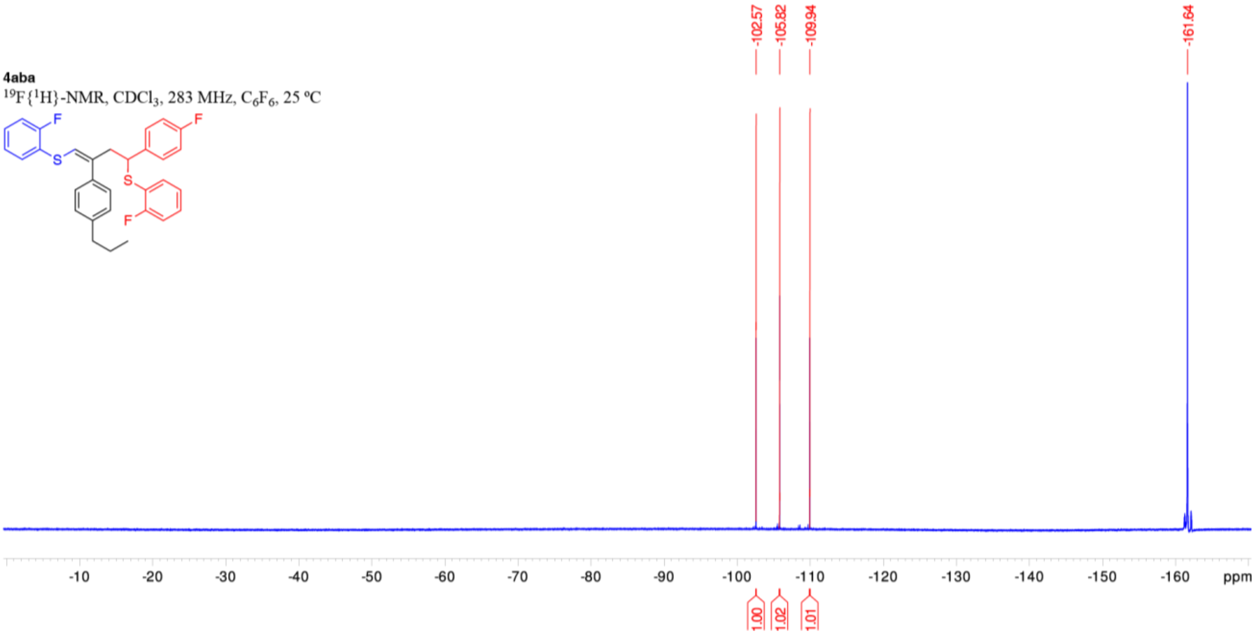

4aca

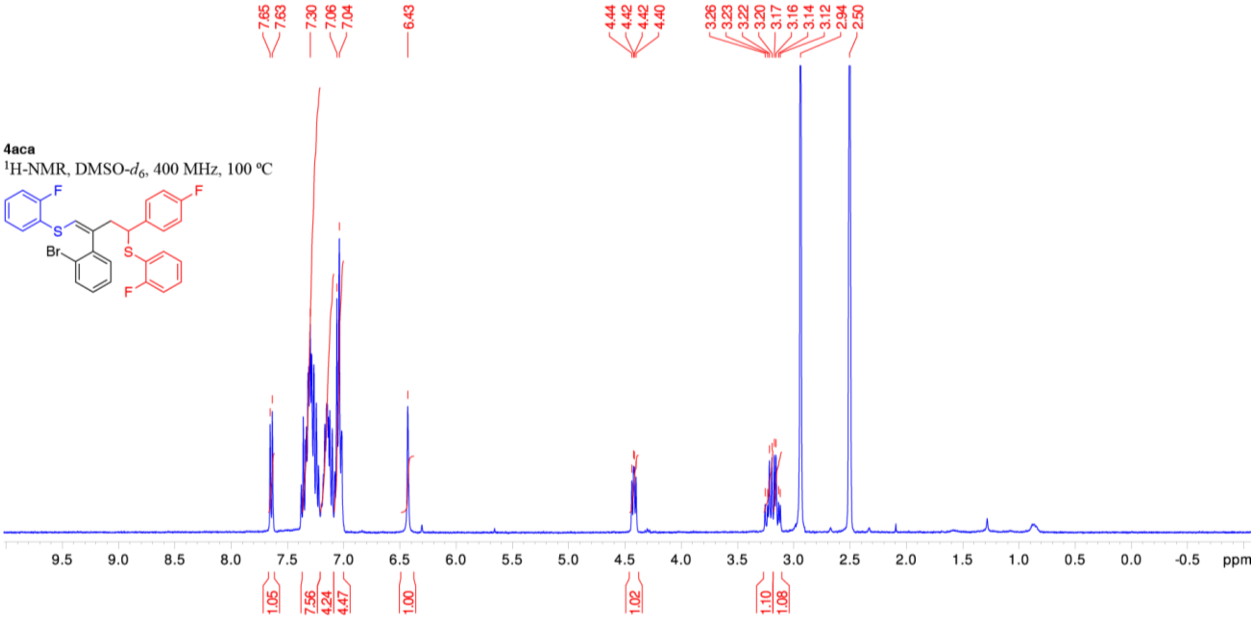
4aca
^{1}H-NMR, DMSO-d_6, 400 MHz, 100 °C
F
S
F
Br
S
F
7.65
7.63
7.30
7.06
7.04
6.43
4.44
4.42
4.42
4.40
3.26
3.23
3.22
3.20
3.17
3.16
3.14
3.12
2.94
2.50
9.5
9.0
8.5
8.0
7.5
7.0
6.5
6.0
5.5
5.0
4.5
4.0
3.5
3.0
2.5
2.0
1.5
1.0
0.5
0.0
-0.5
ppm
1.05
7.56
4.24
4.47
1.00
1.02
1.10
1.08

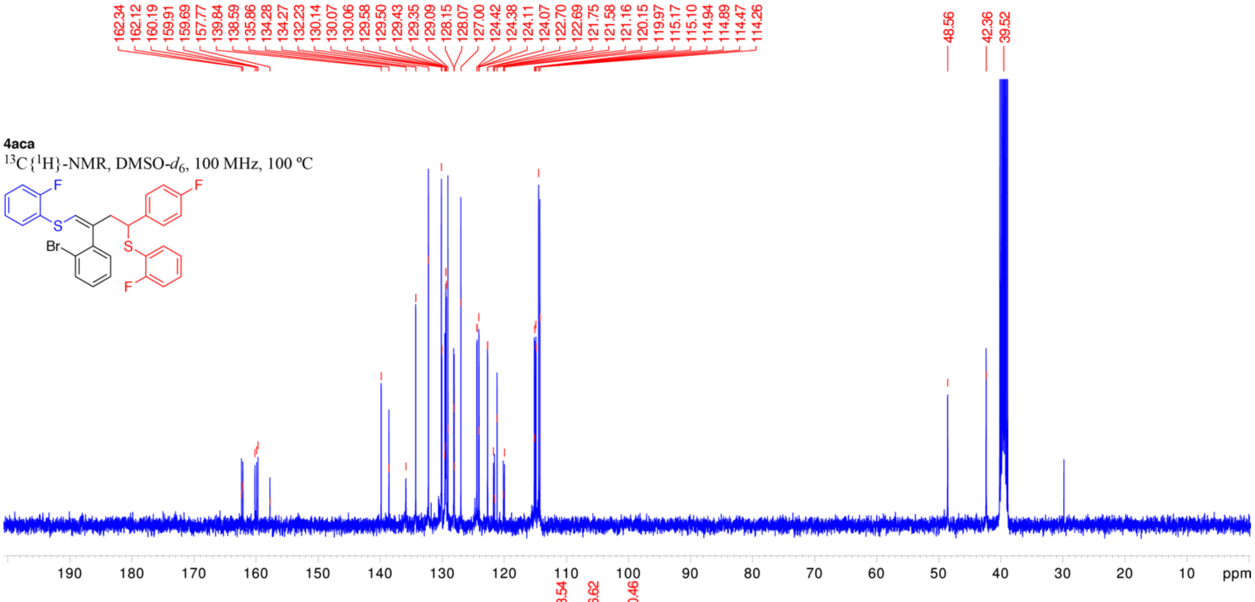
4aca
^{13}C{^{1}H}-NMR, DMSO-d_6, 100 MHz, 100 °C
F
S
F
Br
S
F
162.34
162.12
160.19
159.91
159.69
157.77
139.84
138.59
135.86
134.28
134.27
132.23
130.14
130.07
130.06
129.58
129.50
129.43
129.35
129.09
128.15
128.07
127.00
124.42
124.38
124.11
124.07
122.70
122.69
121.75
121.58
121.16
120.15
119.97
115.17
115.10
114.94
114.89
114.47
114.26
48.56
42.36
39.52
190
180
170
160
150
140
130
120
110
100
90
80
70
60
50
40
30
20
10
ppm

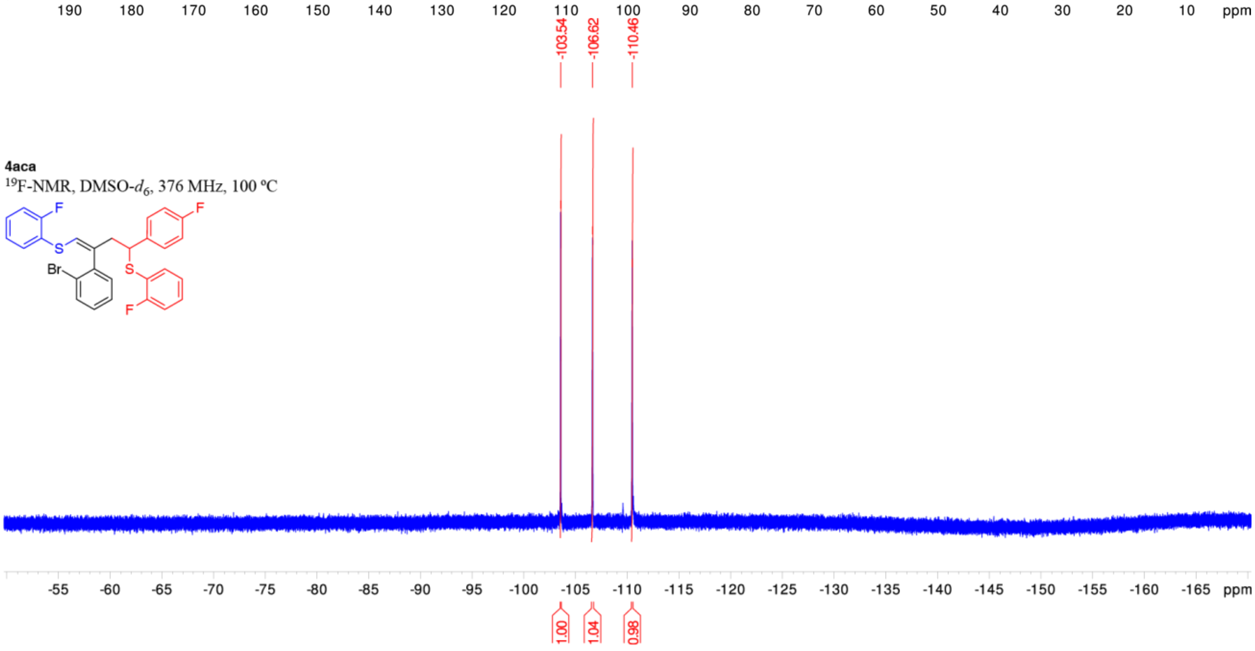
-103.54
-106.62
-110.46
4aca
^{19}F-NMR, DMSO-d_6, 376 MHz, 100 °C
F
S
F
Br
S
F
-55
-60
-65
-70
-75
-80
-85
-90
-95
-100
-105
-110
-115
-120
-125
-130
-135
-140
-145
-150
-155
-160
-165
ppm
1.00
1.04
0.98

4ada

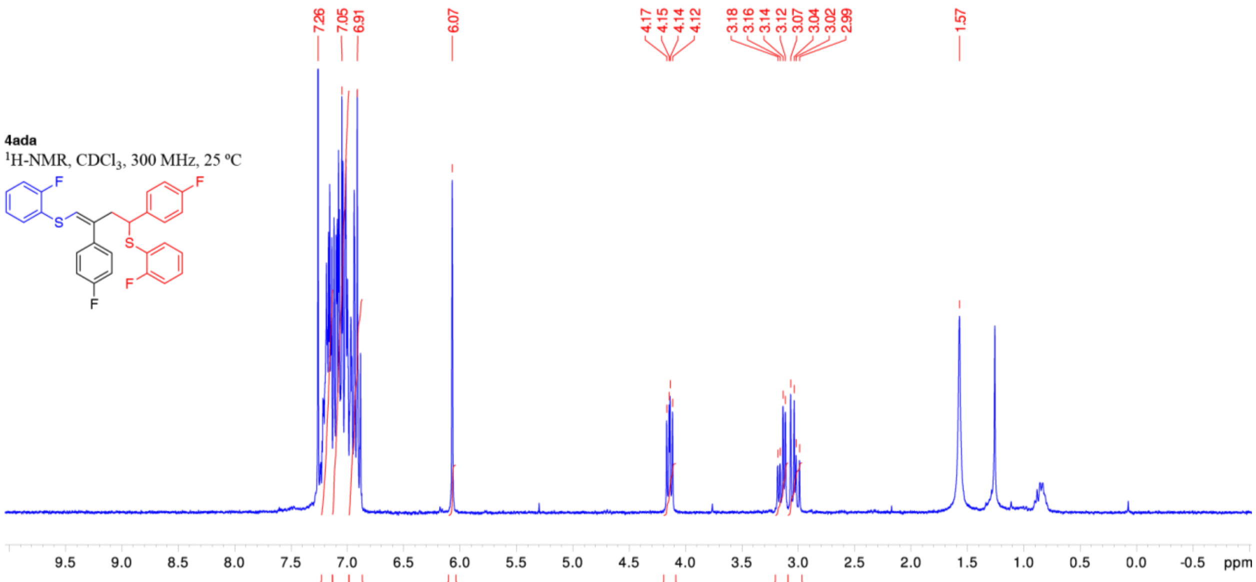

4ada
^{1}H-NMR, CDCl$_3$, 300 MHz, 25 °C
7.26
7.05
6.91
6.07
4.17
4.15
4.14
4.12
3.18
3.16
3.14
3.12
3.07
3.04
3.02
2.99
1.57
ppm

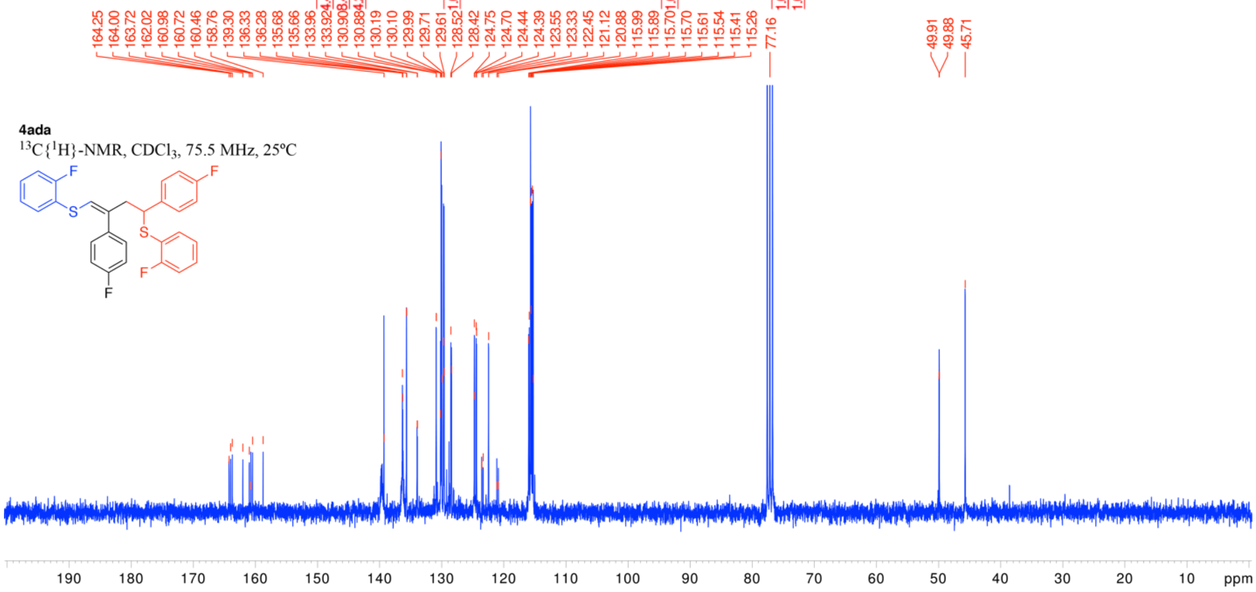

4ada
^{13}C{^{1}H}-NMR, CDCl$_3$, 75.5 MHz, 25°C
77.16
49.91
49.88
45.71
ppm

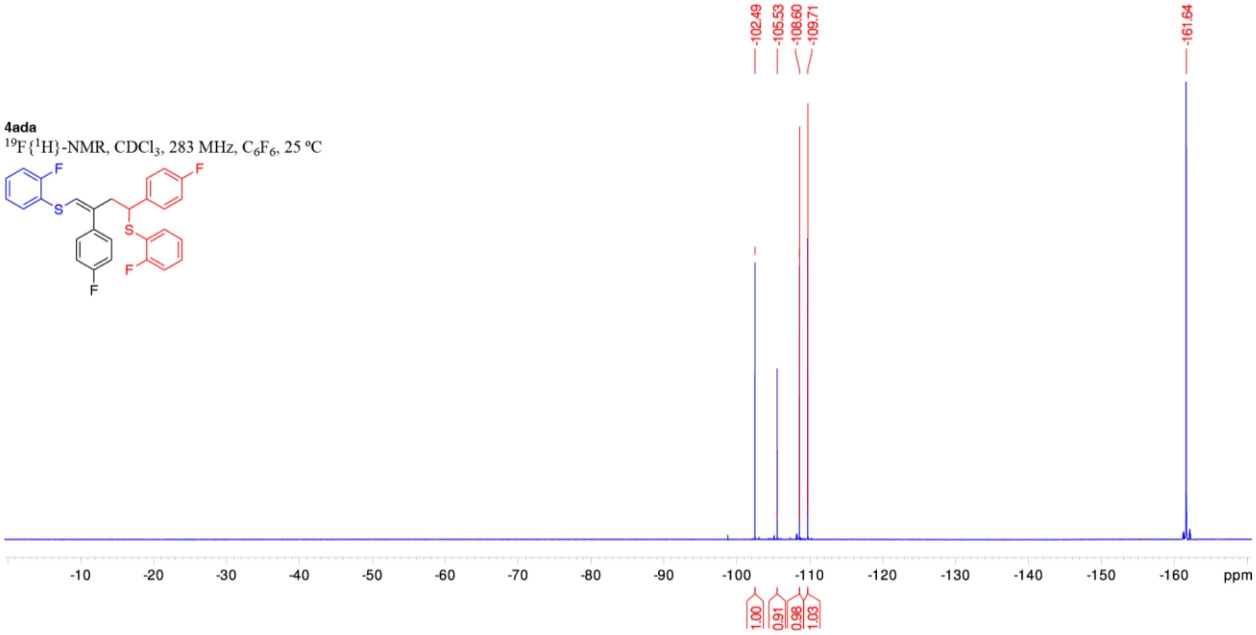

4ada
^{19}F{^{1}H}-NMR, CDCl$_3$, 283 MHz, C$_6$F$_6$, 25 °C
-102.49
-105.53
-108.60
-109.71
-161.64
1.00
0.91
0.98
1.03
ppm

4aea

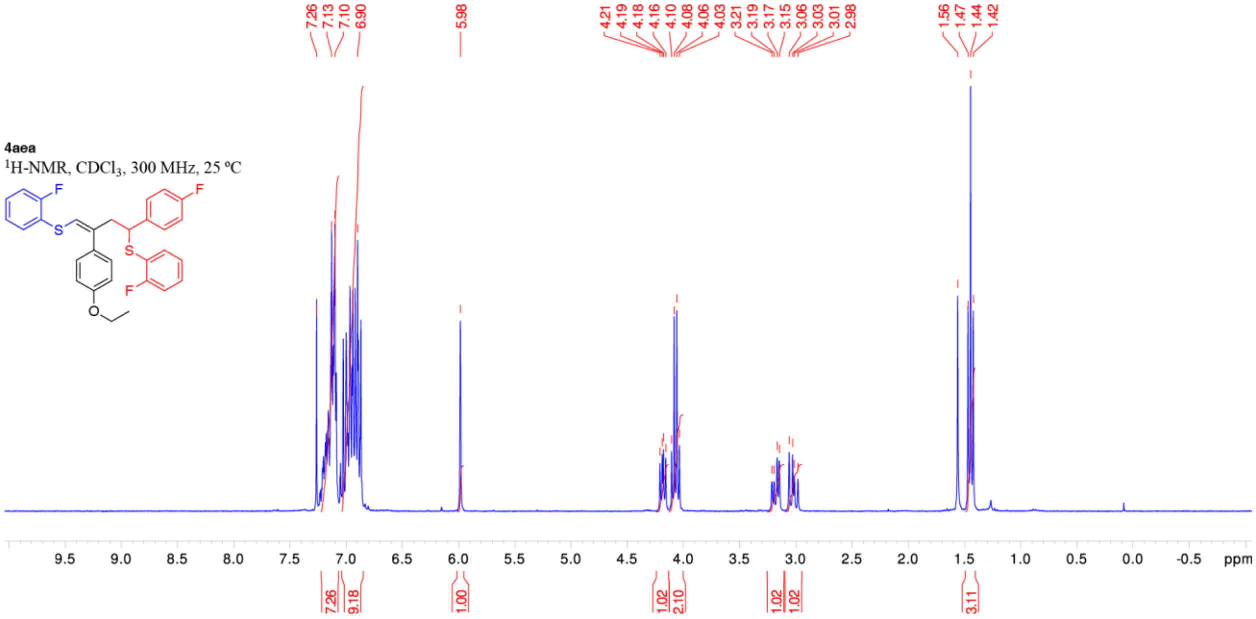
4aea
^{1}H-NMR, CDCl$_3$, 300 MHz, 25 ºC
7.26
7.13
7.10
6.90
5.98
4.21
4.19
4.18
4.16
4.10
4.08
4.06
4.03
3.21
3.19
3.17
3.15
3.06
3.03
3.01
2.98
1.56
1.47
1.44
1.42
7.26
9.18
1.00
1.02
2.10
1.02
1.02
3.11
ppm

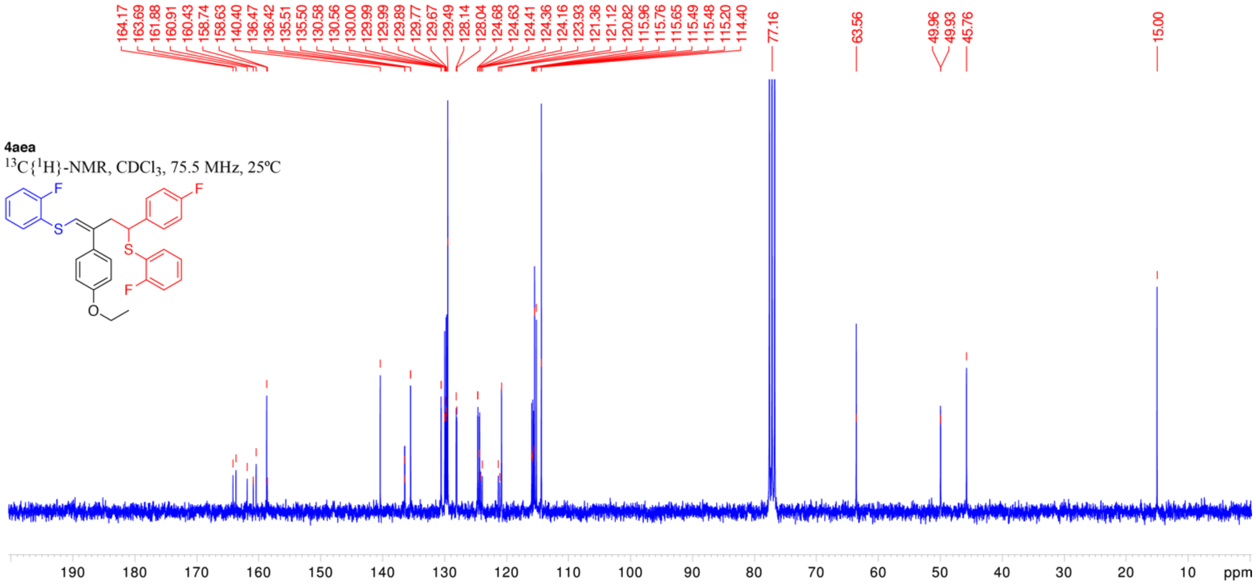
4aea
^{13}C{^{1}H}-NMR, CDCl$_3$, 75.5 MHz, 25ºC
164.17
163.69
161.88
160.91
160.43
158.74
158.63
140.40
136.47
136.42
135.51
135.50
130.58
130.56
130.00
129.99
129.99
129.89
129.77
129.67
129.49
128.14
128.04
124.68
124.63
124.41
124.36
124.16
123.93
121.36
121.12
120.82
115.96
115.76
115.65
115.49
115.48
115.20
114.40
77.16
63.56
49.96
49.93
45.76
15.00
ppm

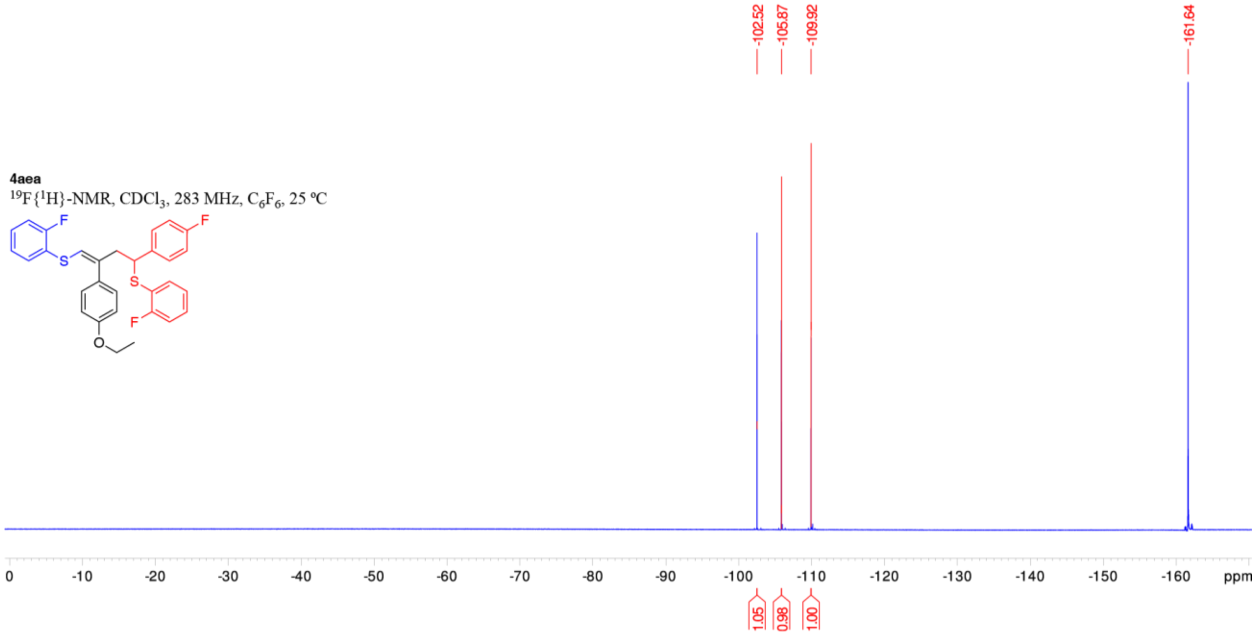
4aea
^{19}F{^{1}H}-NMR, CDCl$_3$, 283 MHz, C$_6$F$_6$, 25 ºC
-102.52
-105.87
-109.92
-161.64
1.05
0.98
1.00
ppm

4afa

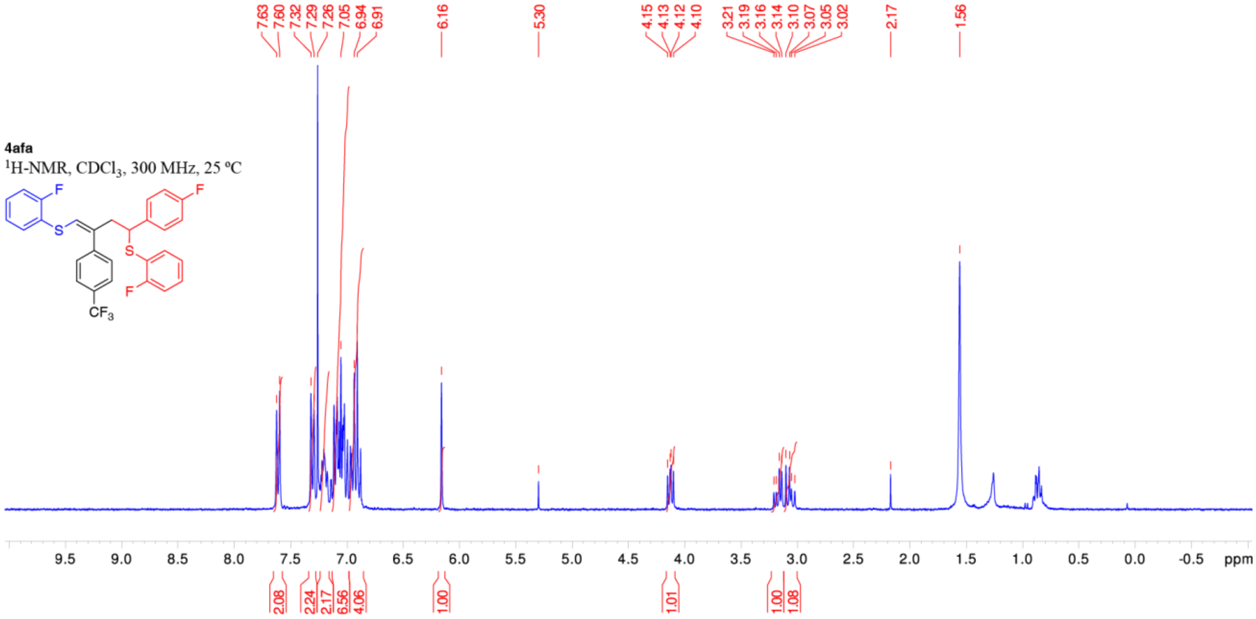
4afa
^{1}H-NMR, $CDCl_3$, 300 MHz, 25 ºC
ppm

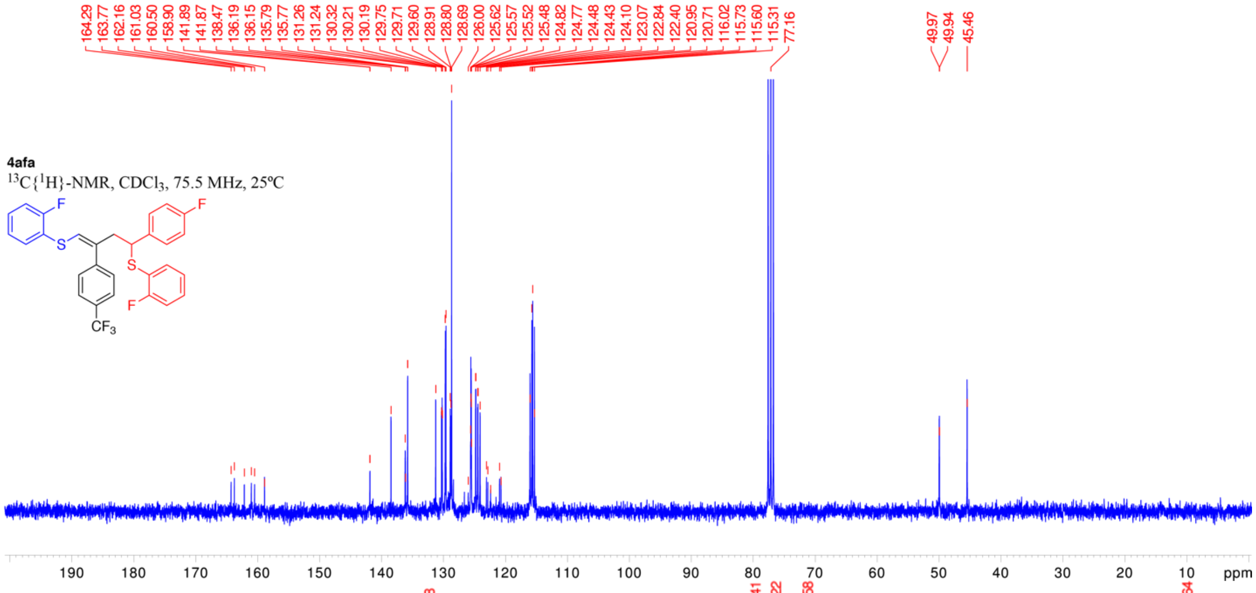
4afa
^{13}C{^{1}H}-NMR, $CDCl_3$, 75.5 MHz, 25ºC
ppm

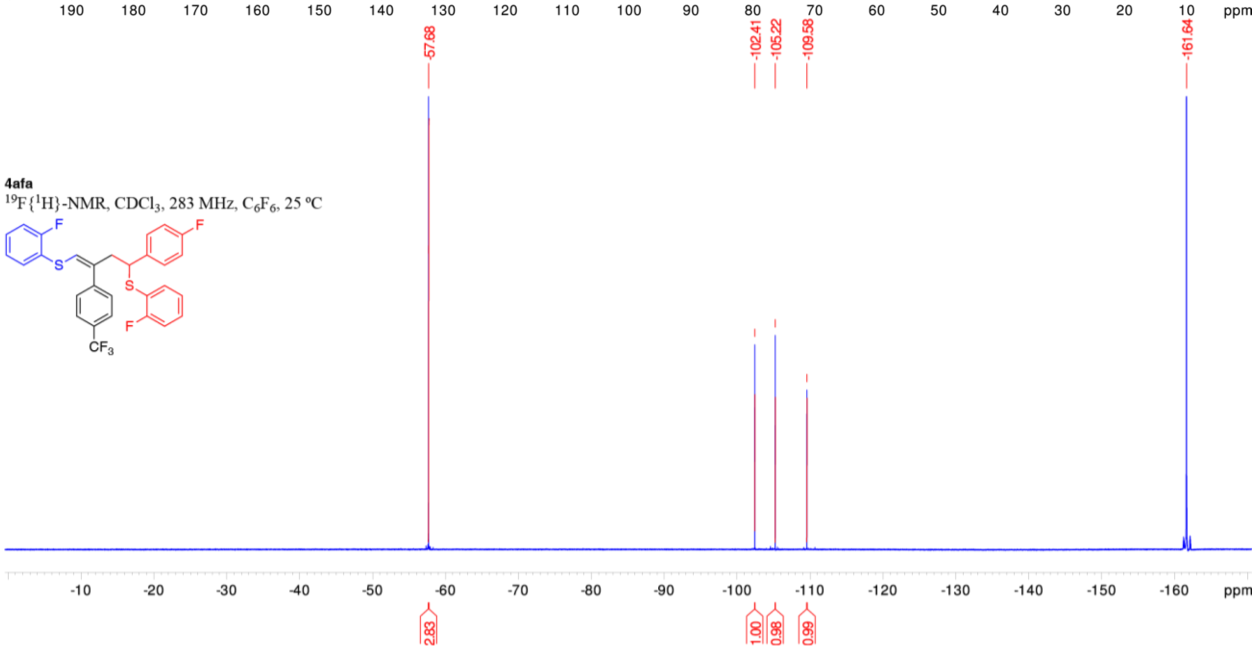
4afa
^{19}F{^{1}H}-NMR, $CDCl_3$, 283 MHz, C_6F_6, 25 ºC
ppm

4aga

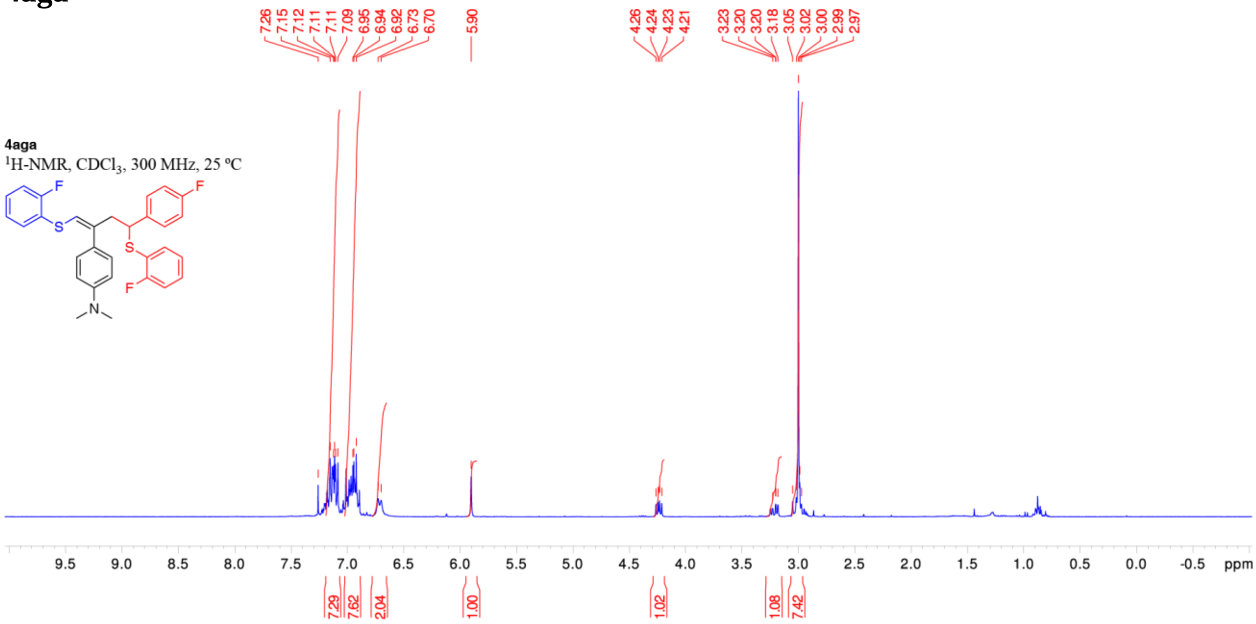

4aga
1H-NMR, CDCl3, 300 MHz, 25 ºC
7.26 7.15 7.12 7.11 7.09 6.95 6.94 6.92 6.73 6.70
5.90
4.26 4.24 4.23 4.21
3.23 3.20 3.20 3.18 3.05 3.02 3.00 2.99 2.97
7.29 7.62 2.04 1.00 1.02 1.08 7.42
ppm

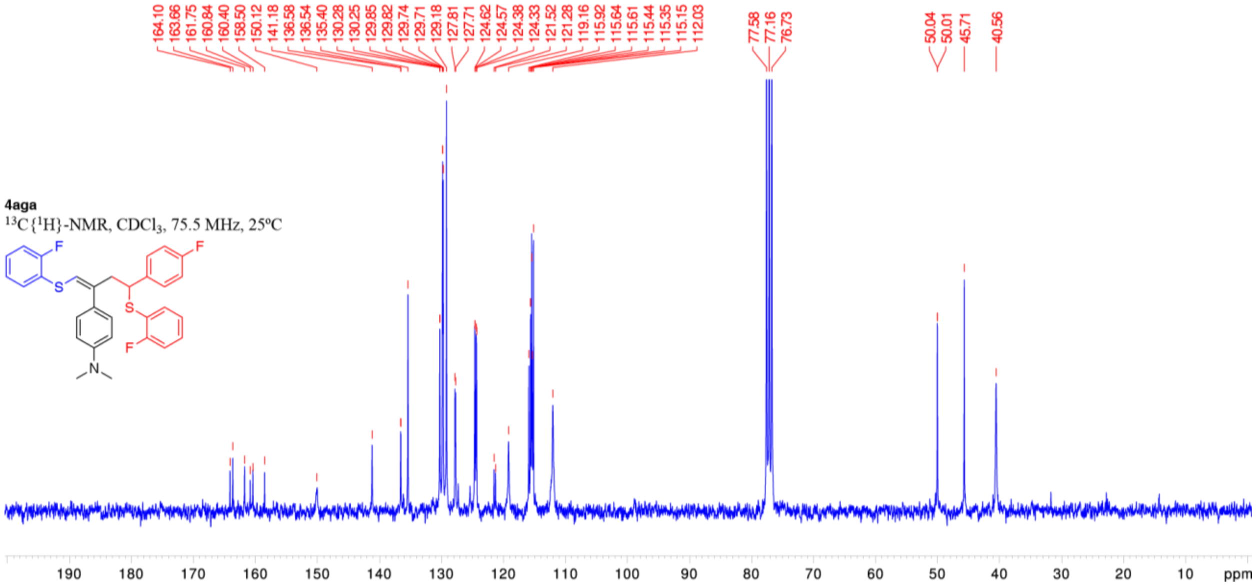

4aga
13C{1H}-NMR, CDCl3, 75.5 MHz, 25ºC
164.10 163.66 161.75 160.84 160.40 158.50 150.12 141.18 136.58 136.54 135.40 130.28 130.25 129.85 129.82 129.74 129.71 129.18 127.81 127.71 124.62 124.57 124.38 124.33 121.52 121.28 119.16 115.92 115.64 115.61 115.44 115.35 115.15 112.03
77.58 77.16 76.73
50.04 50.01 45.71 40.56
ppm

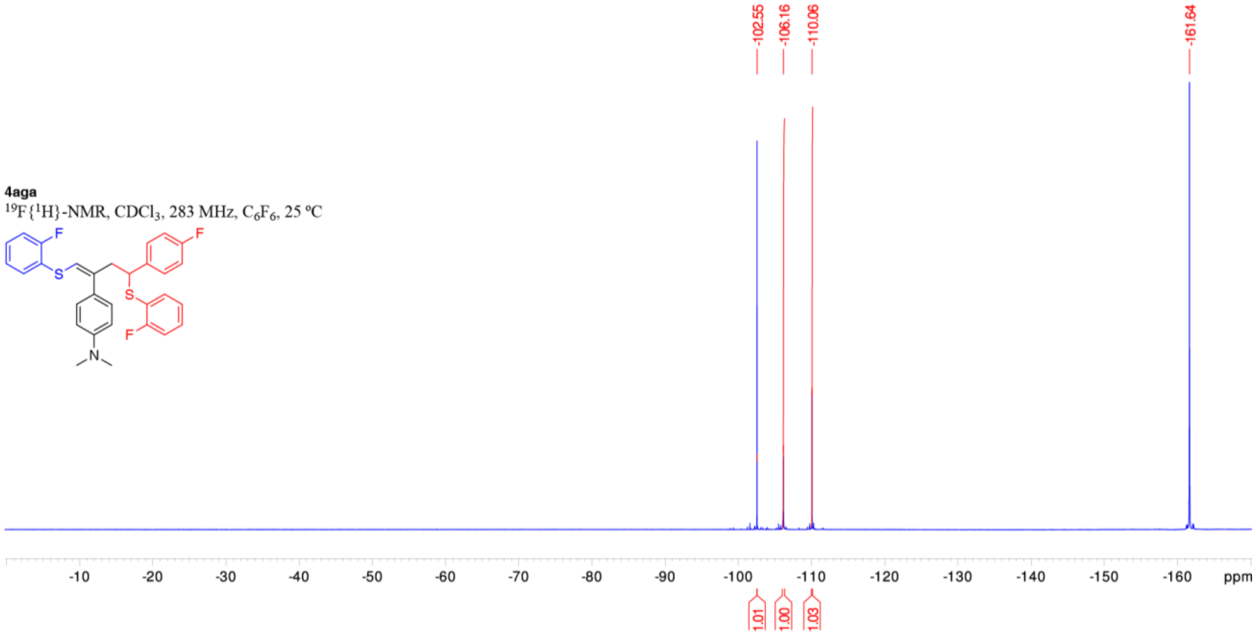

4aga
19F{1H}-NMR, CDCl3, 283 MHz, C6F6, 25 ºC
-102.55 -106.16 -110.06 -161.64
1.01 1.00 1.03
ppm

4aab

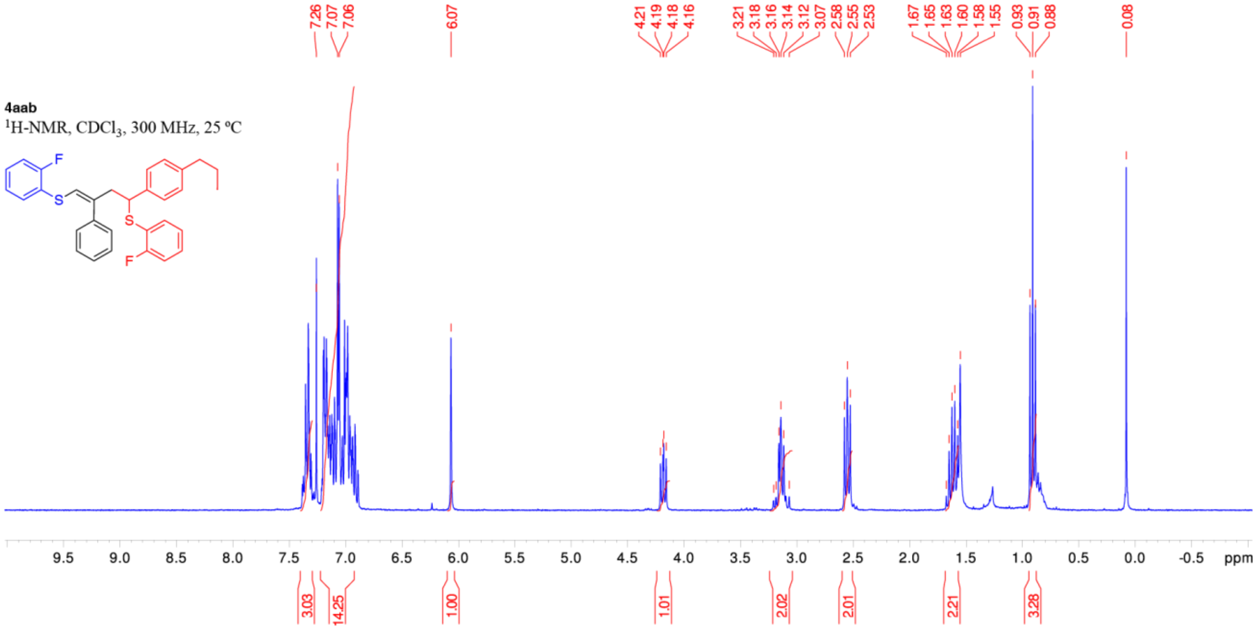
4aab
^{1}H-NMR, CDCl$_3$, 300 MHz, 25 ºC

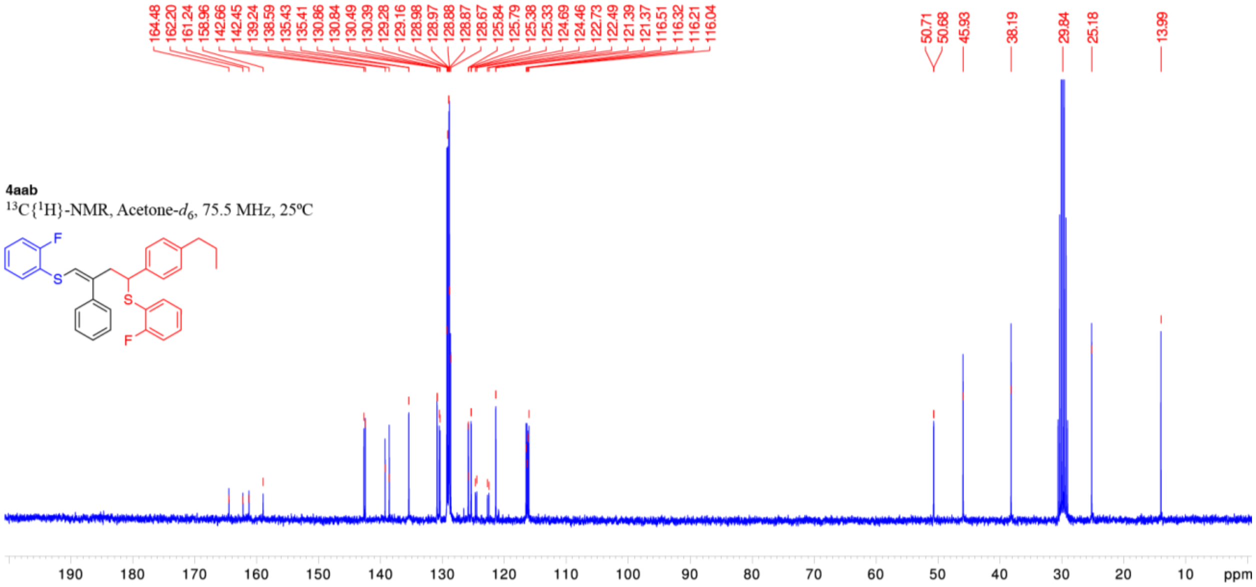
4aab
^{13}C{^{1}H}-NMR, Acetone-d_6, 75.5 MHz, 25ºC

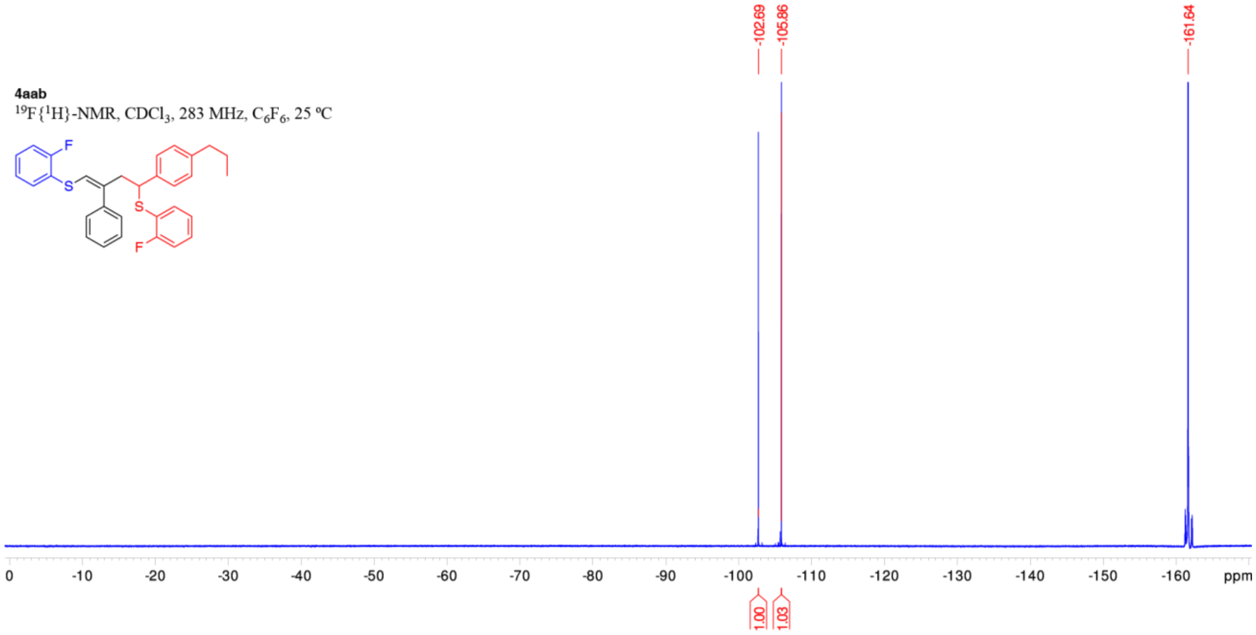
4aab
^{19}F{^{1}H}-NMR, CDCl$_3$, 283 MHz, C$_6$F$_6$, 25 ºC
-102.69
-105.86
-161.64

4abb

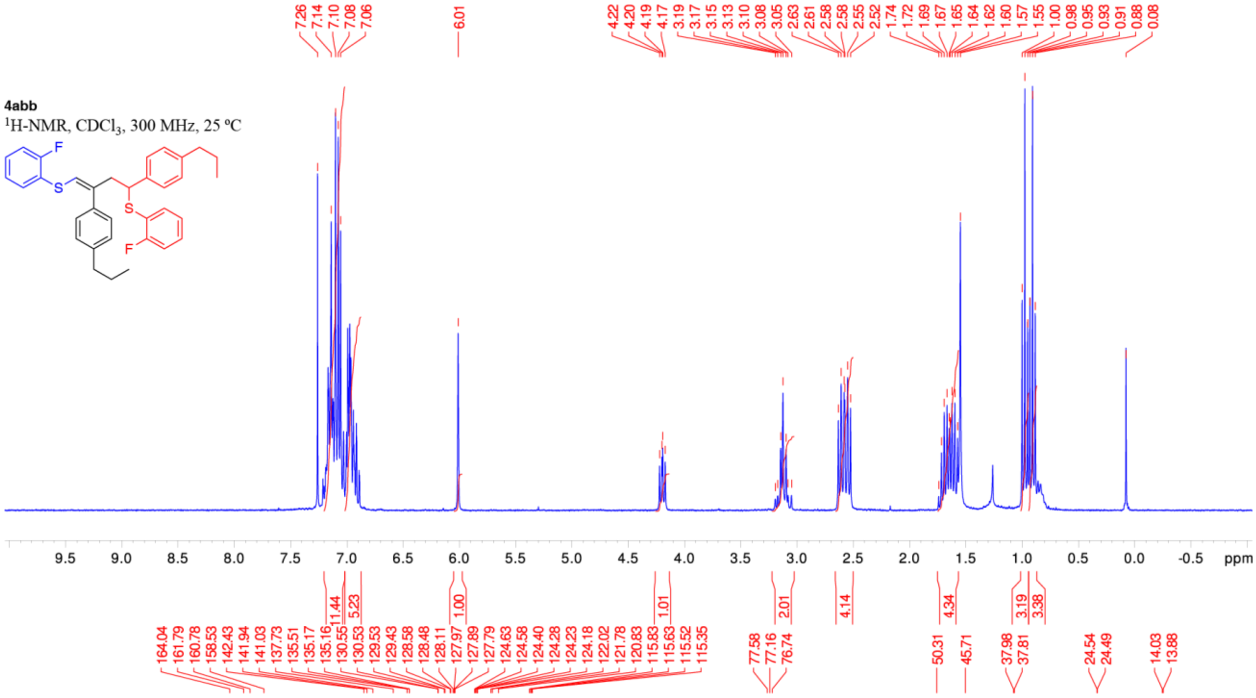
4abb
¹H-NMR, CDCl₃, 300 MHz, 25 ºC
7.26 7.14 7.10 7.08 7.06
6.01
4.22 4.20 4.19 4.17 3.19 3.17 3.15 3.13 3.10 3.08 3.05 2.63 2.61 2.58 2.58 2.55 2.52 1.74 1.72 1.69 1.67 1.65 1.64 1.62 1.60 1.57 1.55 1.00 0.98 0.95 0.93 0.91 0.88 0.08
11.44 5.23 1.00 1.01 2.01 4.14 4.34 3.19 3.38
ppm

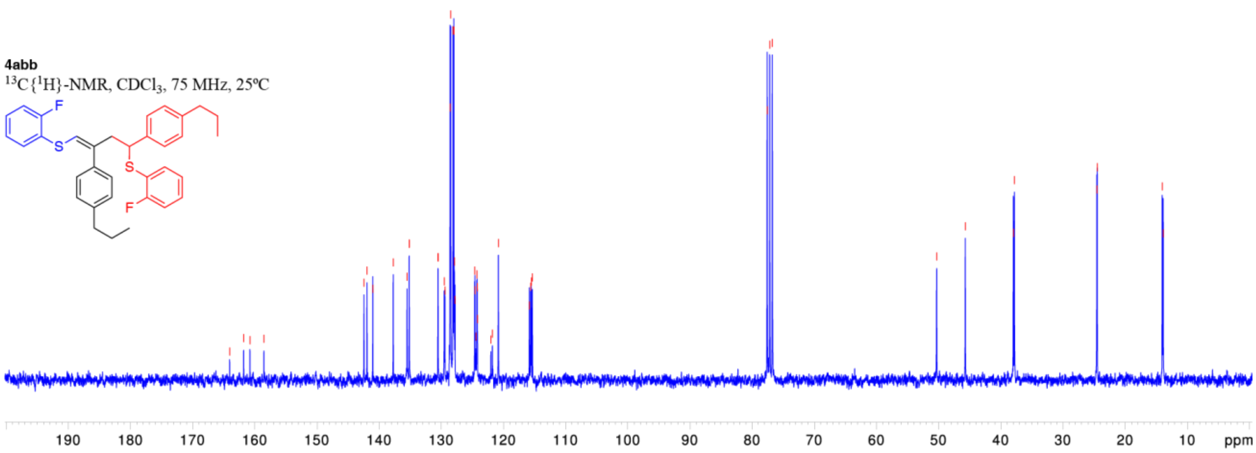
164.04 161.79 160.78 158.53 142.43 141.94 141.03 137.73 135.51 135.17 135.16 130.55 130.53 129.53 129.43 128.58 128.48 128.11 127.97 127.89 127.79 124.63 124.58 124.40 124.28 124.23 124.18 122.02 121.78 120.83 115.83 115.63 115.52 115.35
77.58 77.16 76.74
50.31 45.71 37.98 37.81 24.54 24.49 14.03 13.88
4abb
¹³C{¹H}-NMR, CDCl₃, 75 MHz, 25ºC
ppm

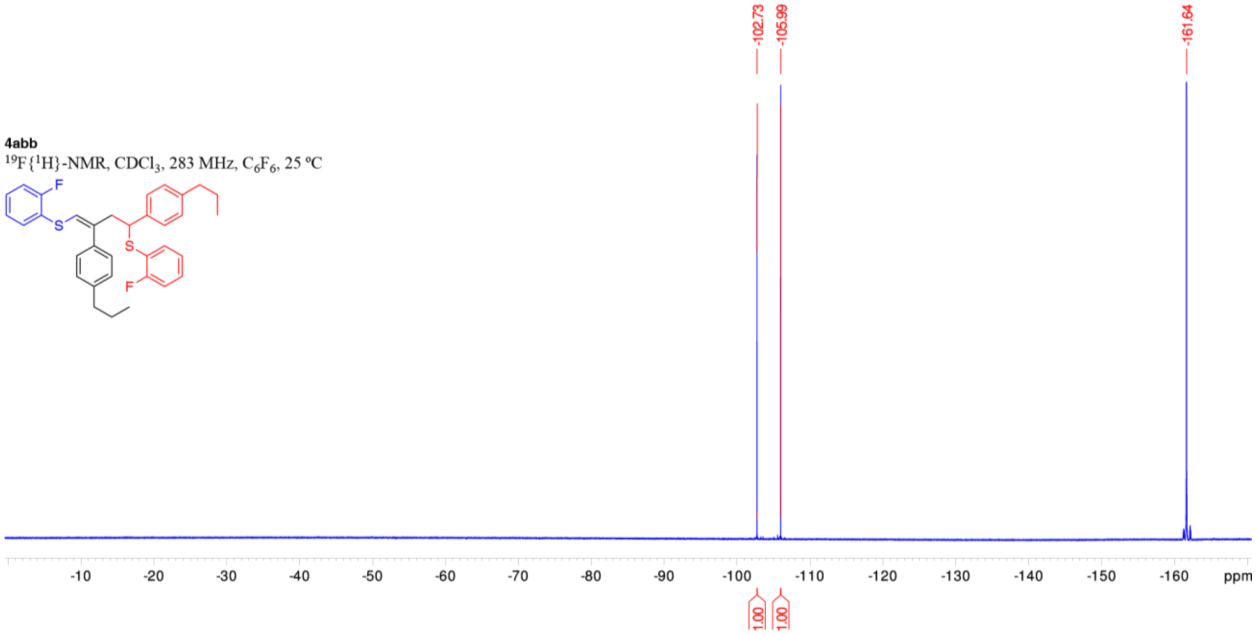
-102.73 -105.99 -161.64
4abb
¹⁹F{¹H}-NMR, CDCl₃, 283 MHz, C₆F₆, 25 ºC
ppm
1.00 1.00

4acb

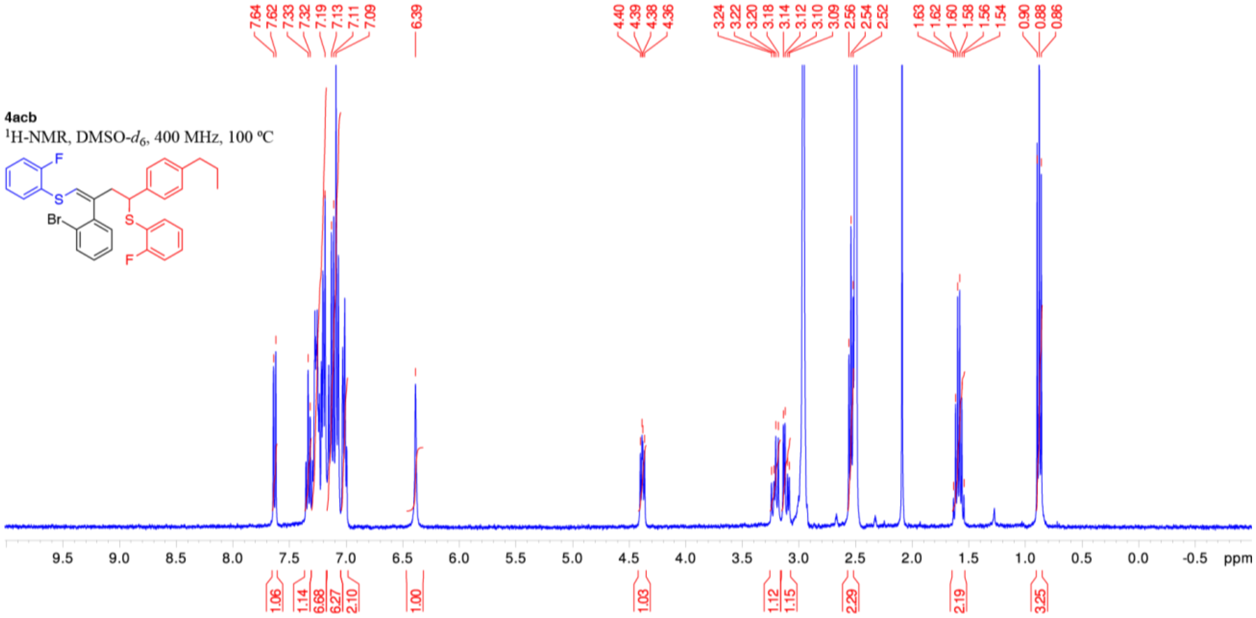
4acb
1H-NMR, DMSO-d6, 400 MHz, 100 ºC

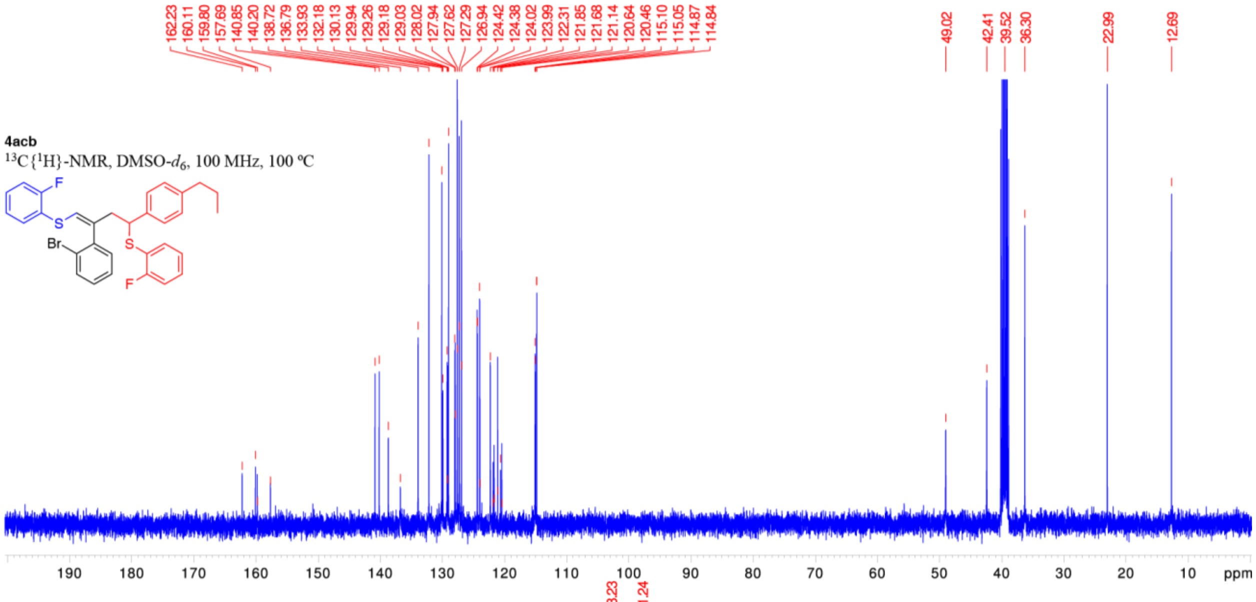
4acb
13C{1H}-NMR, DMSO-d6, 100 MHz, 100 ºC

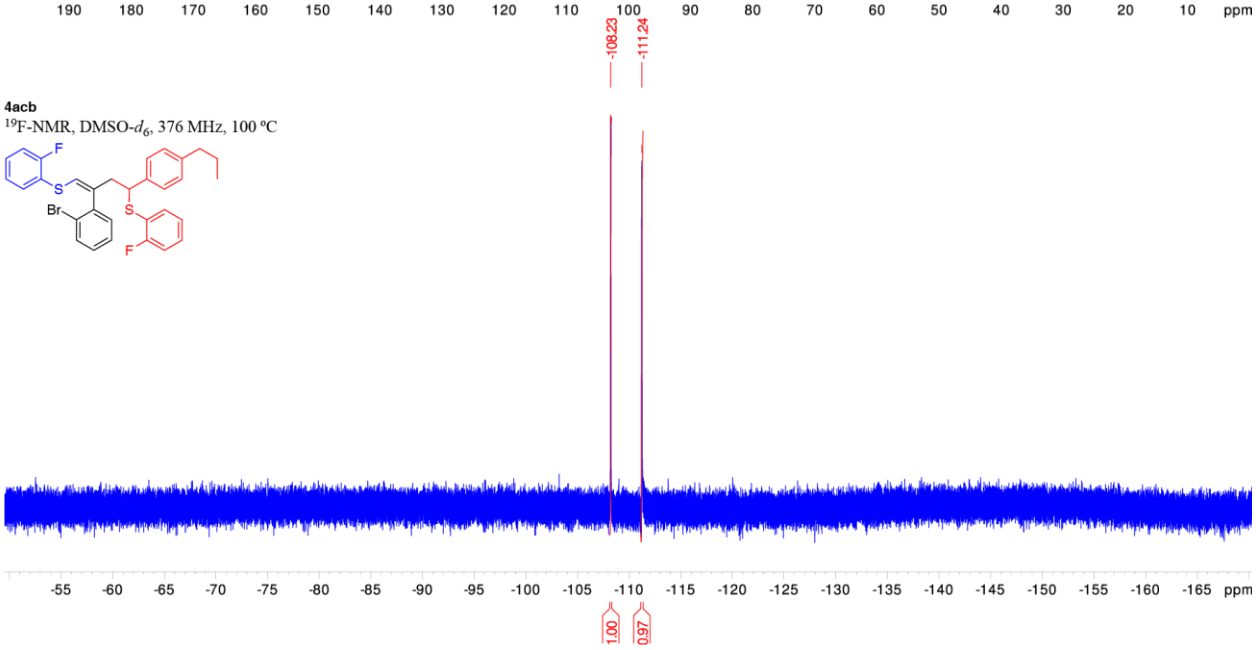
4acb
19F-NMR, DMSO-d6, 376 MHz, 100 ºC
-108.23
-111.24

4adb

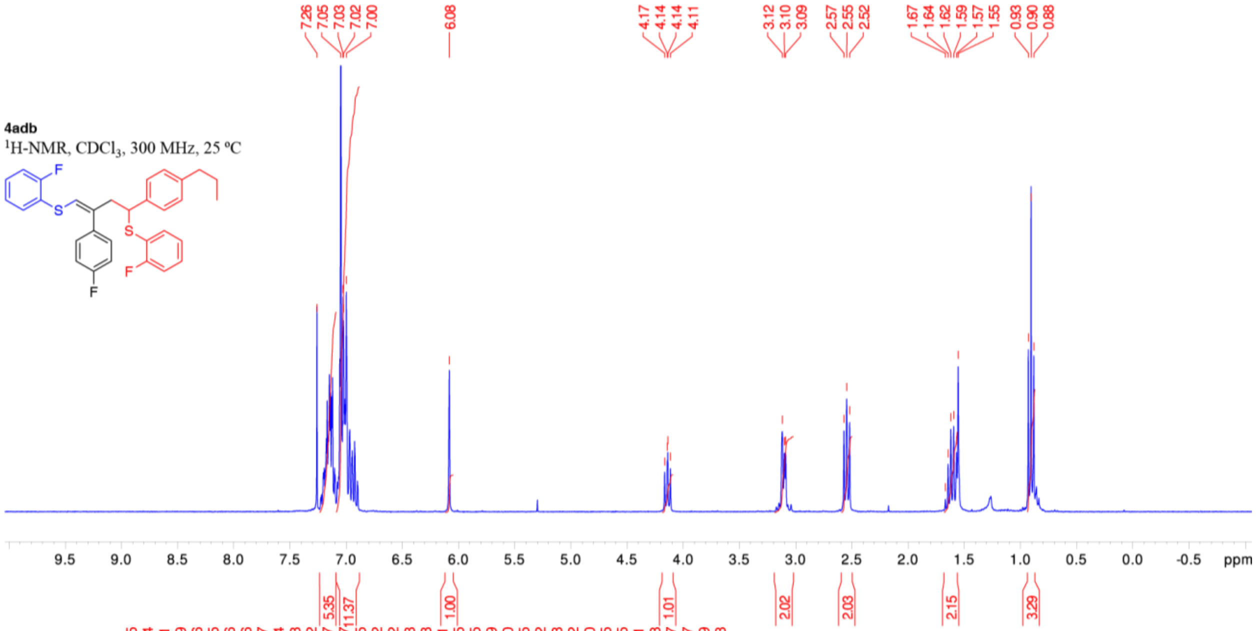
4adb
1H-NMR, CDCl3, 300 MHz, 25 °C
ppm

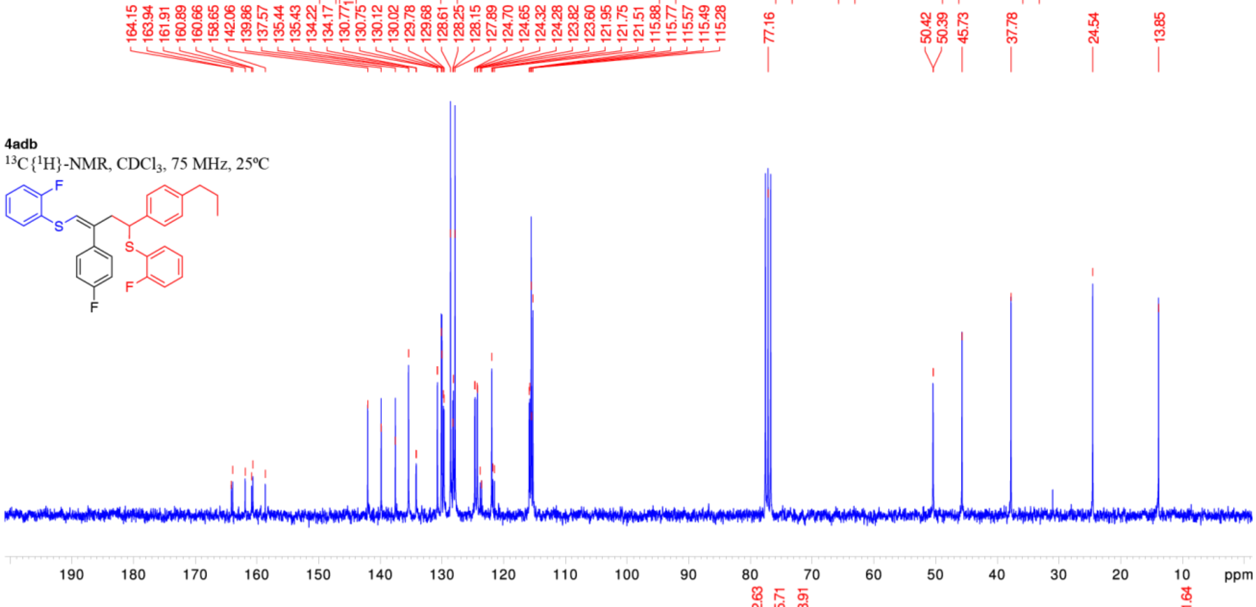
4adb
13C{1H}-NMR, CDCl3, 75 MHz, 25ºC
ppm

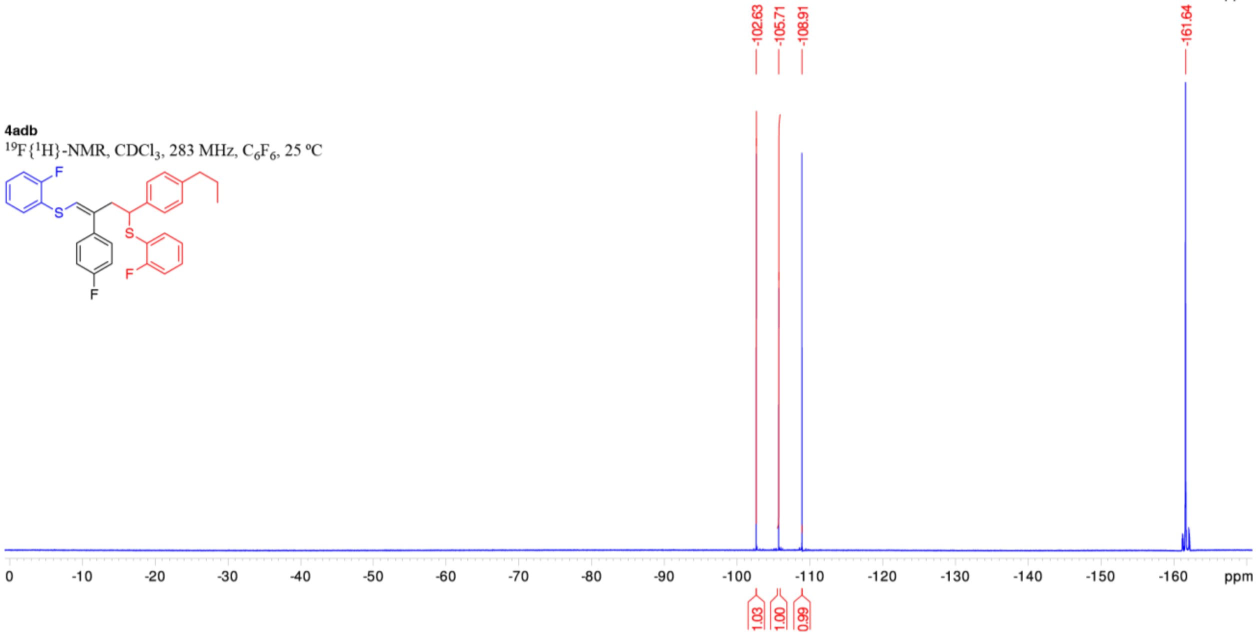
4adb
19F{1H}-NMR, CDCl3, 283 MHz, C6F6, 25 °C
-102.63
-105.71
-108.91
-161.64
ppm

4aeb

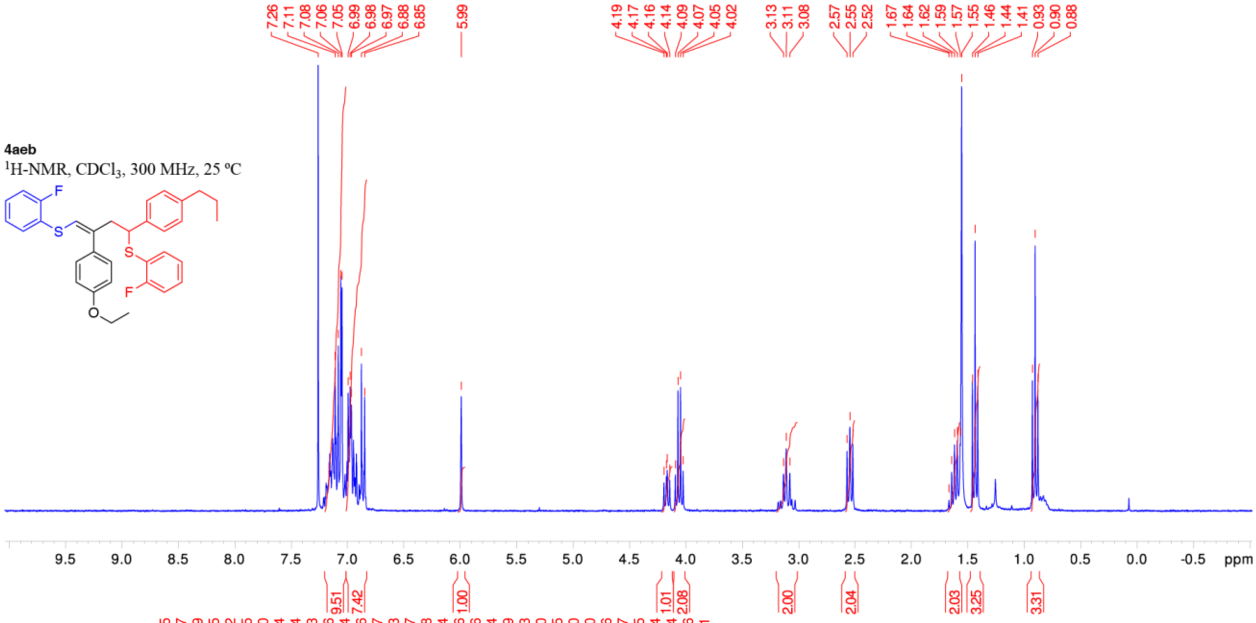
4aeb
^{1}H-NMR, $CDCl_3$, 300 MHz, 25 ºC

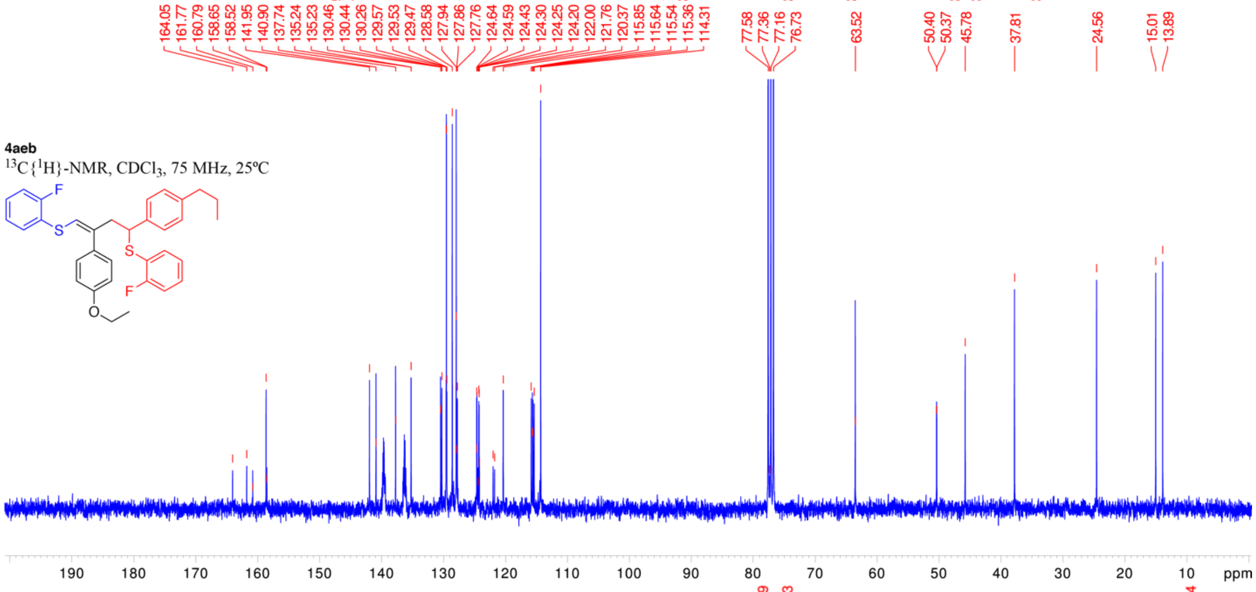
4aeb
^{13}C{^{1}H}-NMR, $CDCl_3$, 75 MHz, 25ºC

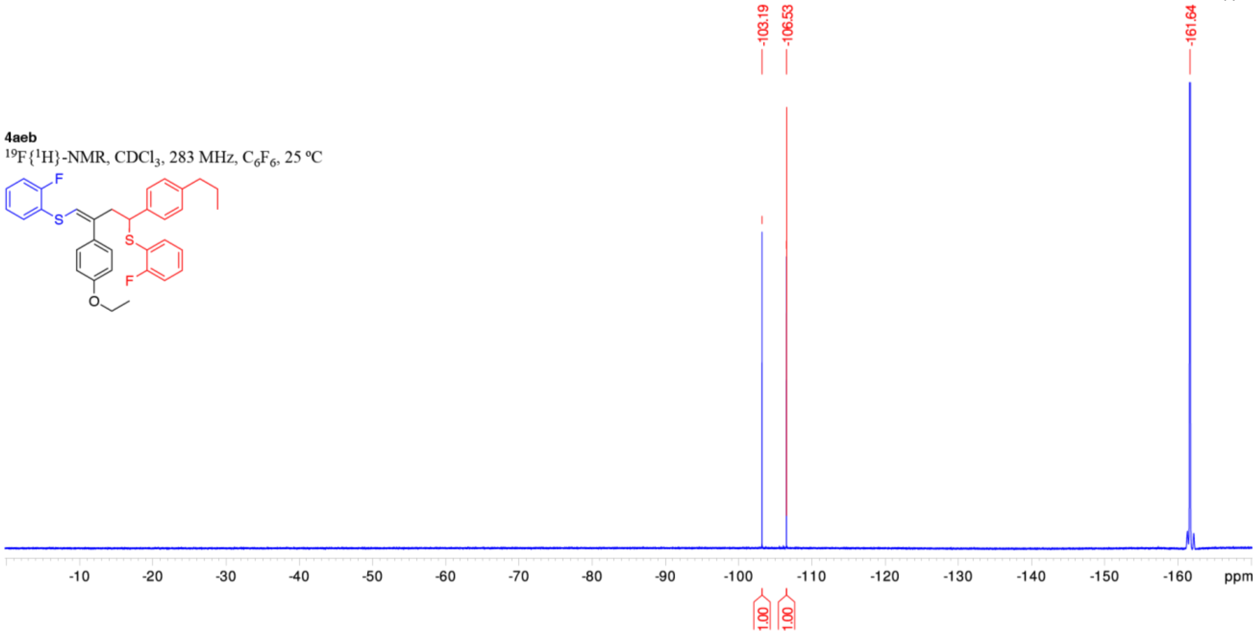
4aeb
^{19}F{^{1}H}-NMR, $CDCl_3$, 283 MHz, C_6F_6, 25 ºC

4afb

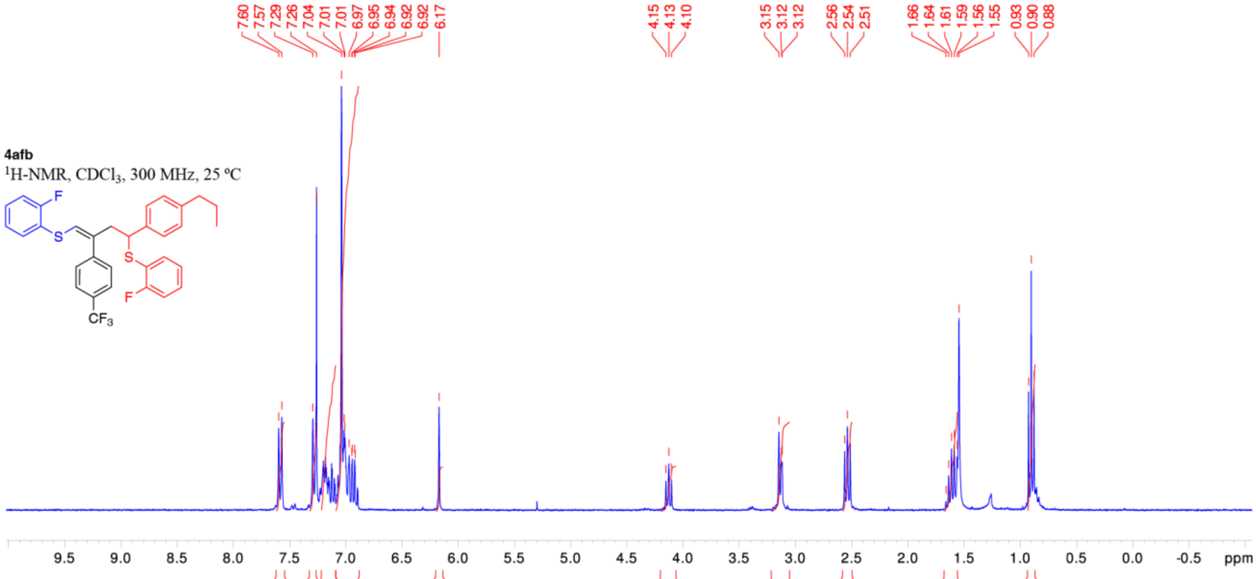
4afb
^{1}H-NMR, $CDCl_3$, 300 MHz, 25 ºC
ppm

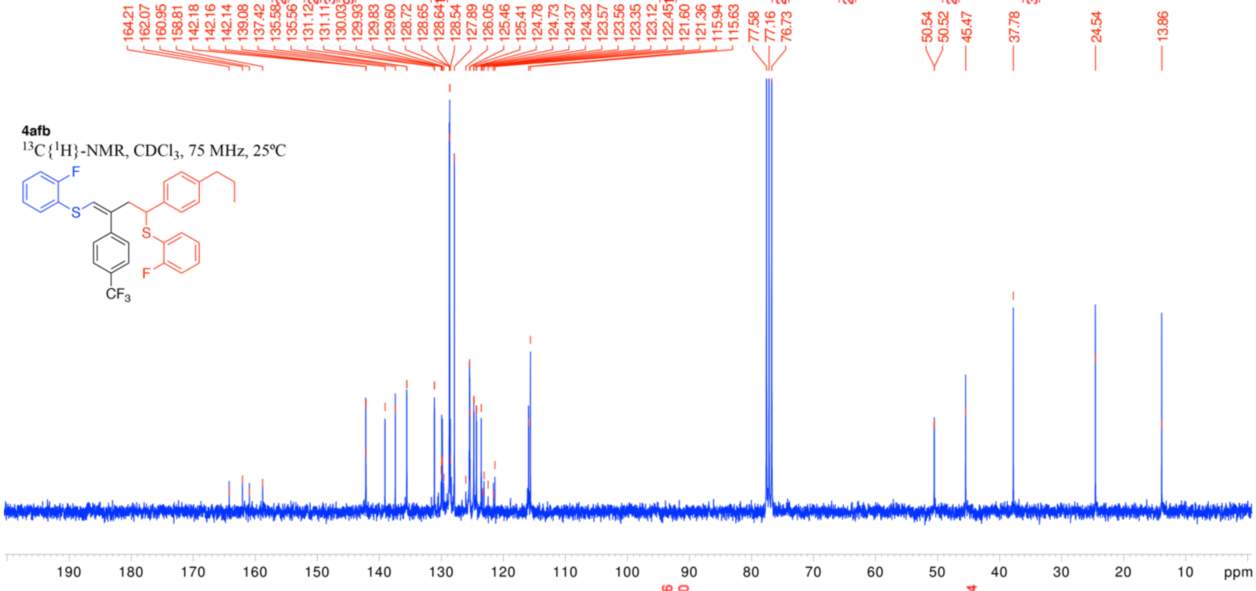
4afb
^{13}C{^{1}H}-NMR, $CDCl_3$, 75 MHz, 25ºC
ppm

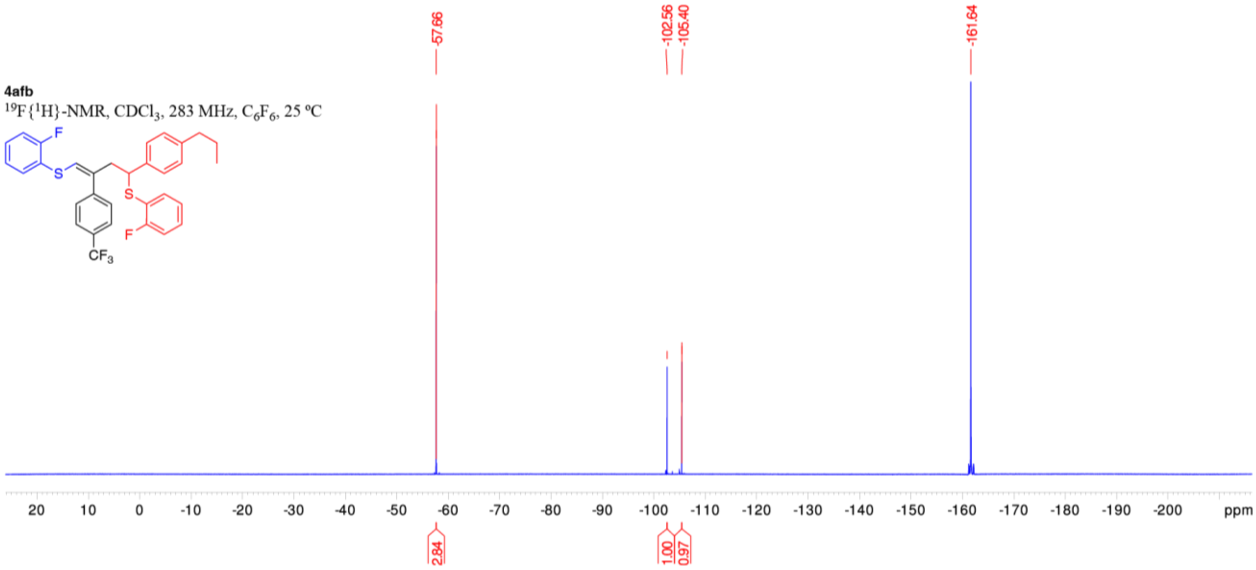
4afb
^{19}F{^{1}H}-NMR, $CDCl_3$, 283 MHz, C_6F_6, 25 ºC
ppm

4aac

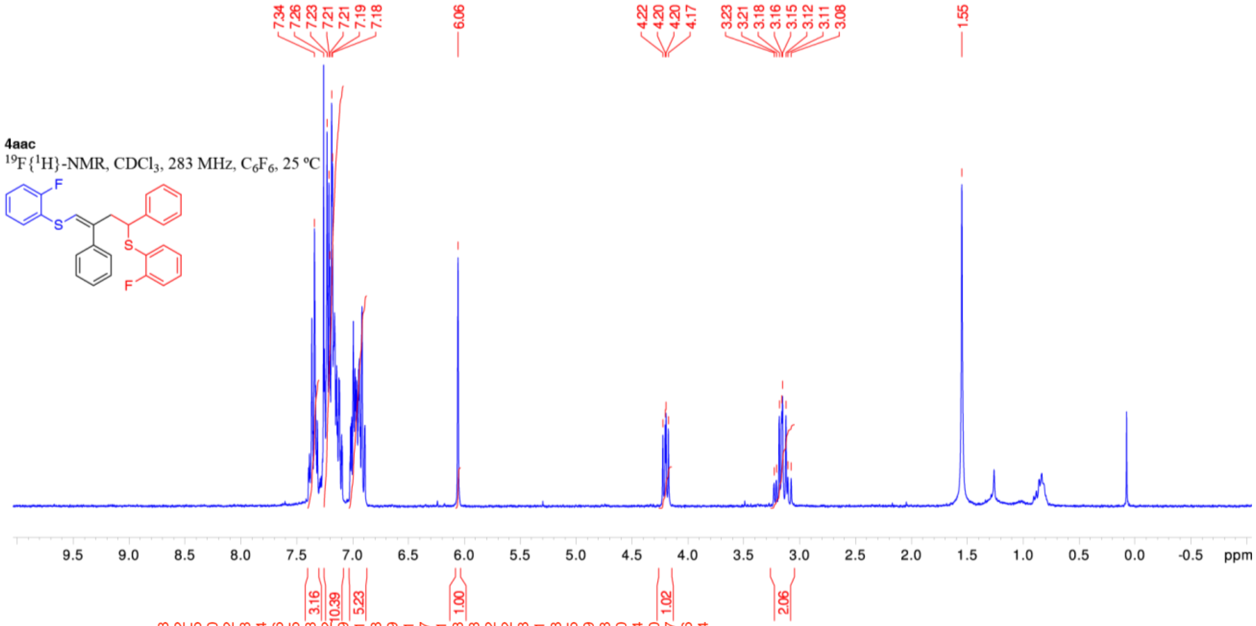
7.34
7.26
7.23
7.21
7.21
7.19
7.18
6.06
4.22
4.20
4.20
4.17
3.23
3.21
3.18
3.16
3.15
3.12
3.11
3.08
1.55
4aac
^{19}F{^{1}H}-NMR, CDCl$_3$, 283 MHz, C$_6$F$_6$, 25 ºC
F
S
S
F
9.5
9.0
8.5
8.0
7.5
7.0
6.5
6.0
5.5
5.0
4.5
4.0
3.5
3.0
2.5
2.0
1.5
1.0
0.5
0.0
-0.5
ppm
3.16
10.39
5.23
1.00
1.02
2.06

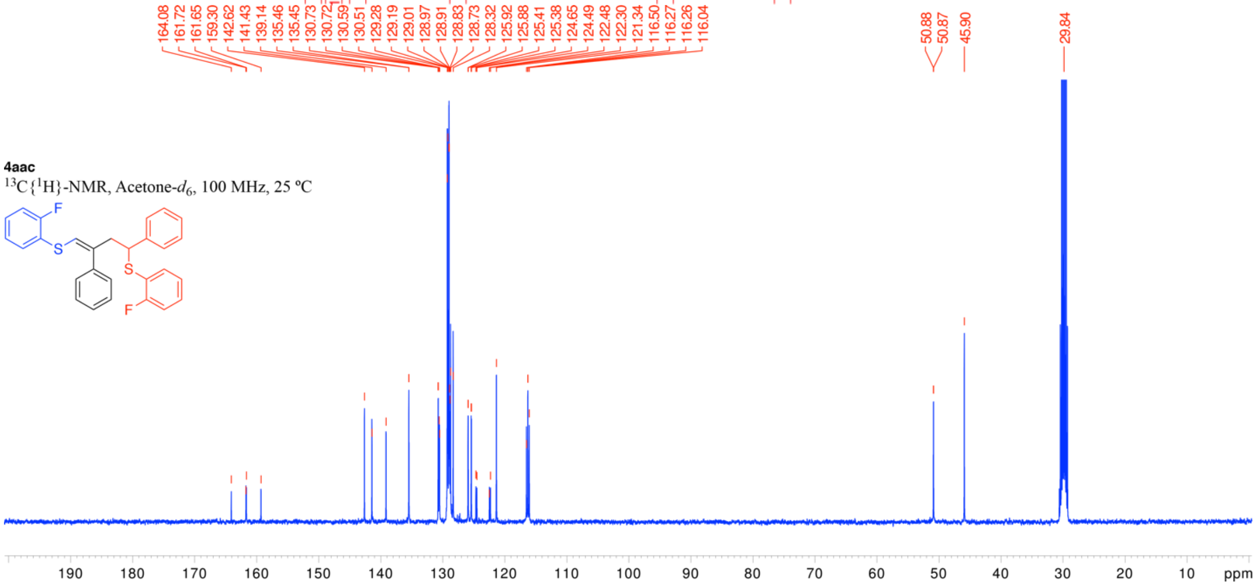
164.08
161.72
161.65
159.30
142.62
141.43
139.14
135.46
135.45
130.73
130.72
130.59
130.51
129.28
129.19
129.01
128.97
128.91
128.83
128.73
128.32
125.92
125.88
125.41
125.38
124.65
124.49
122.48
122.30
121.34
116.50
116.27
116.26
116.04
50.88
50.87
45.90
29.84
4aac
^{13}C{^{1}H}-NMR, Acetone-d_6, 100 MHz, 25 ºC
F
S
S
F
190
180
170
160
150
140
130
120
110
100
90
80
70
60
50
40
30
20
10
ppm

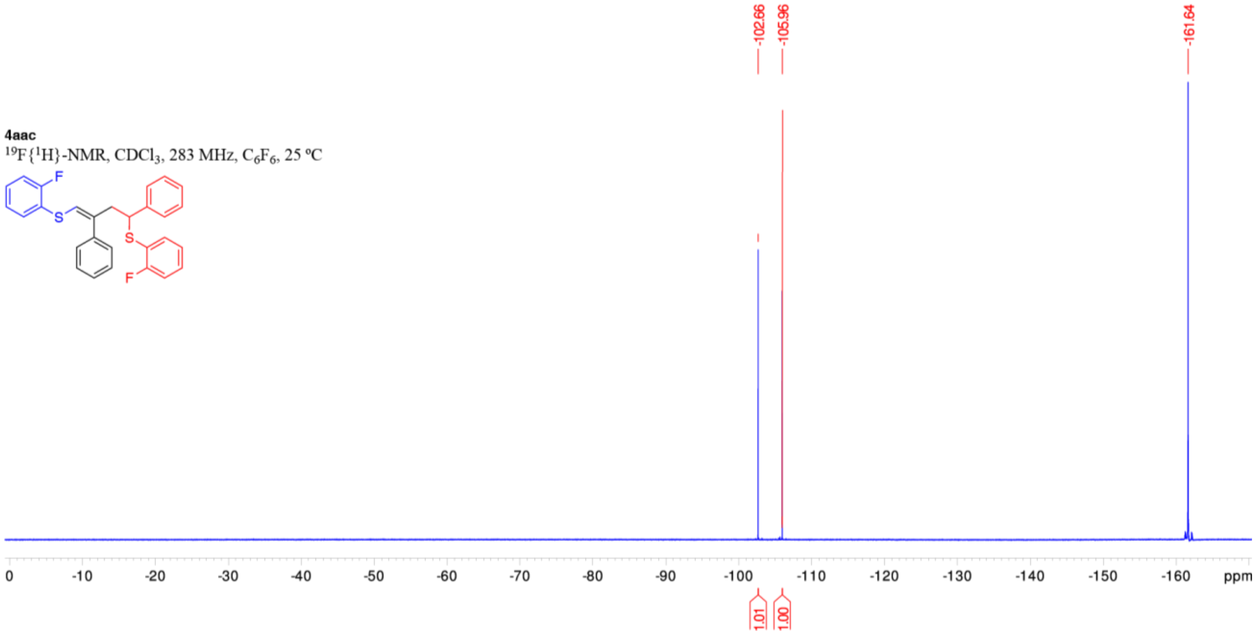
-102.66
-105.96
-161.64
4aac
^{19}F{^{1}H}-NMR, CDCl$_3$, 283 MHz, C$_6$F$_6$, 25 ºC
F
S
S
F
0
-10
-20
-30
-40
-50
-60
-70
-80
-90
-100
-110
-120
-130
-140
-150
-160
ppm
1.01
1.00

4abc

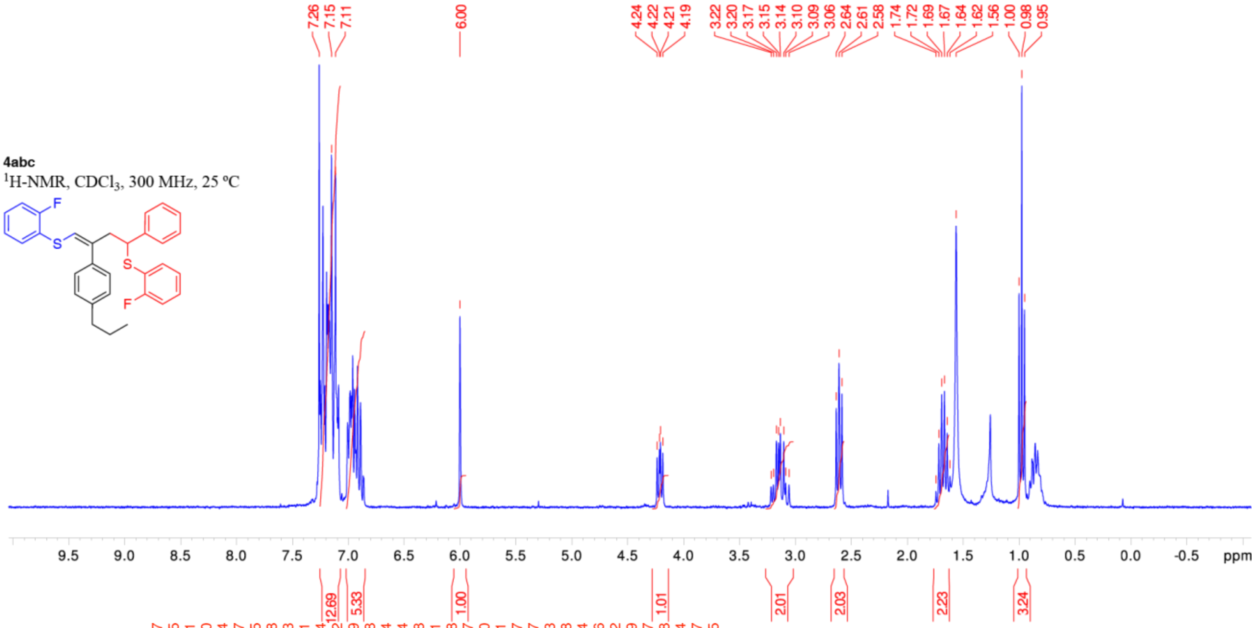
4abc
1H-NMR, CDCl3, 300 MHz, 25 ºC
F
S
S
F
7.26 7.15 7.11
6.00
4.24 4.22 4.21 4.19
3.22 3.20 3.17 3.15 3.14 3.10 3.09 3.06 2.64 2.61 2.58 1.74 1.72 1.69 1.67 1.64 1.62 1.56 1.00 0.98 0.95
12.69 5.33 1.00 1.01 2.01 2.03 2.23 3.24
9.5 9.0 8.5 8.0 7.5 7.0 6.5 6.0 5.5 5.0 4.5 4.0 3.5 3.0 2.5 2.0 1.5 1.0 0.5 0.0 -0.5 ppm

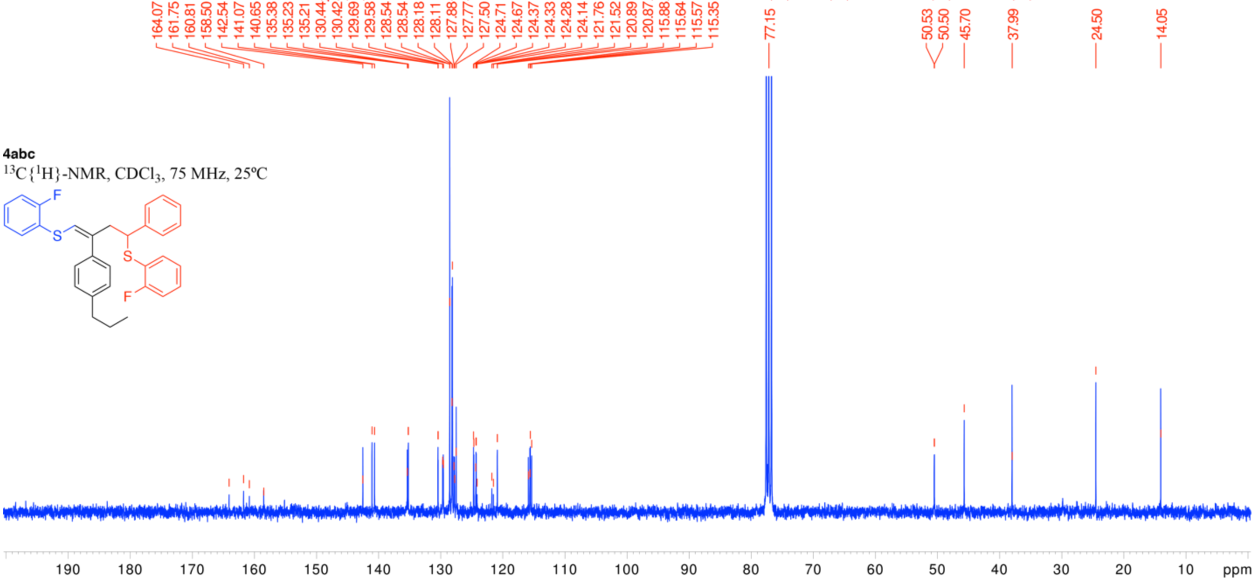
4abc
13C{1H}-NMR, CDCl3, 75 MHz, 25ºC
F
S
S
F
164.07 161.75 160.81 158.50 142.54 141.07 140.65 135.38 135.23 135.21 130.44 130.42 129.69 129.58 128.54 128.54 128.18 128.11 127.88 127.77 127.50 124.71 124.67 124.37 124.33 124.28 124.14 121.76 121.52 120.89 120.87 115.88 115.64 115.57 115.35
77.15
50.53 50.50 45.70 37.99 24.50 14.05
190 180 170 160 150 140 130 120 110 100 90 80 70 60 50 40 30 20 10 ppm

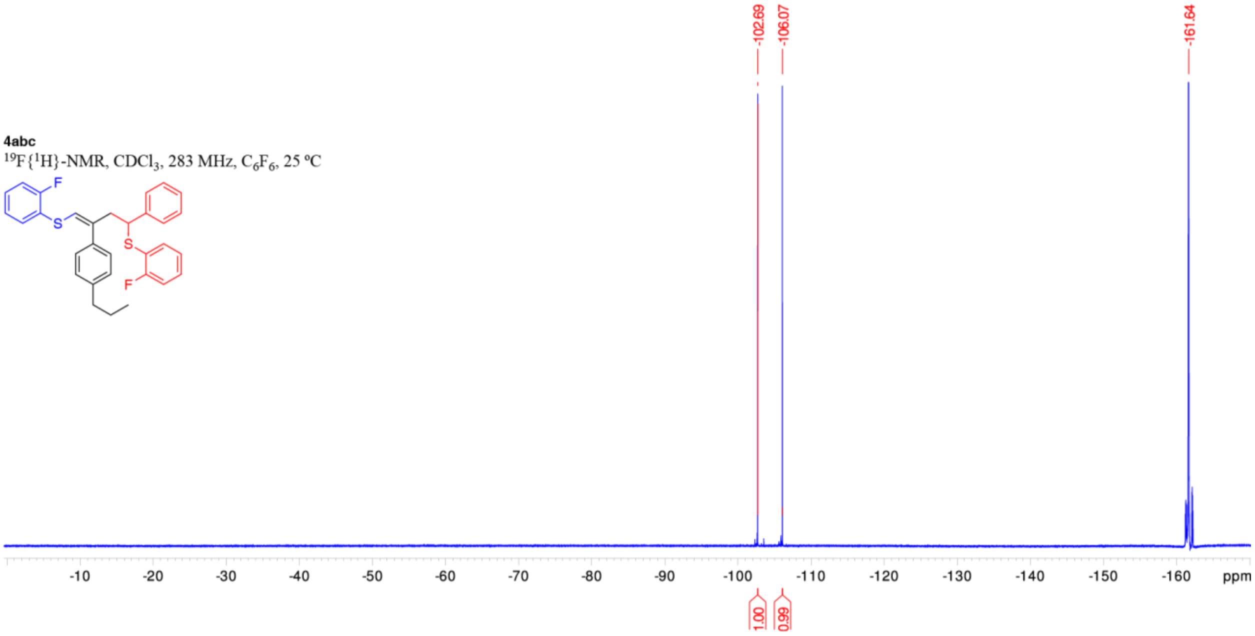
4abc
19F{1H}-NMR, CDCl3, 283 MHz, C6F6, 25 ºC
F
S
S
F
-102.69 -106.07 -161.64
-10 -20 -30 -40 -50 -60 -70 -80 -90 -100 -110 -120 -130 -140 -150 -160 ppm
1.00 0.99

4acc

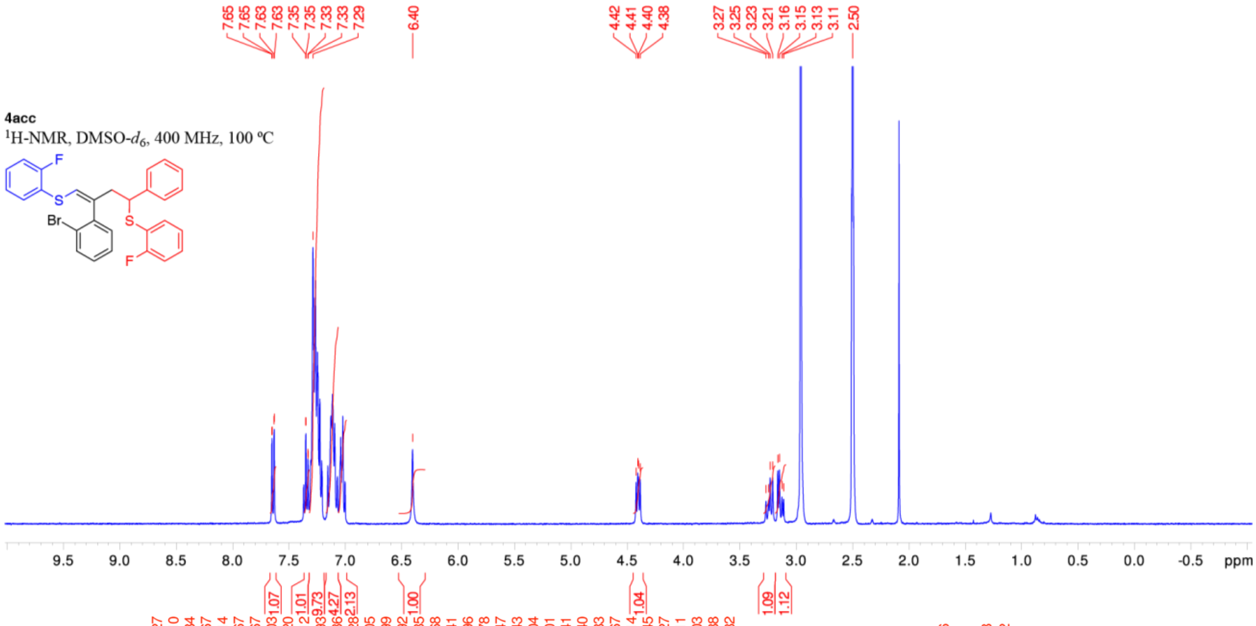

4acc
1H-NMR, DMSO-d6, 400 MHz, 100 ºC
ppm

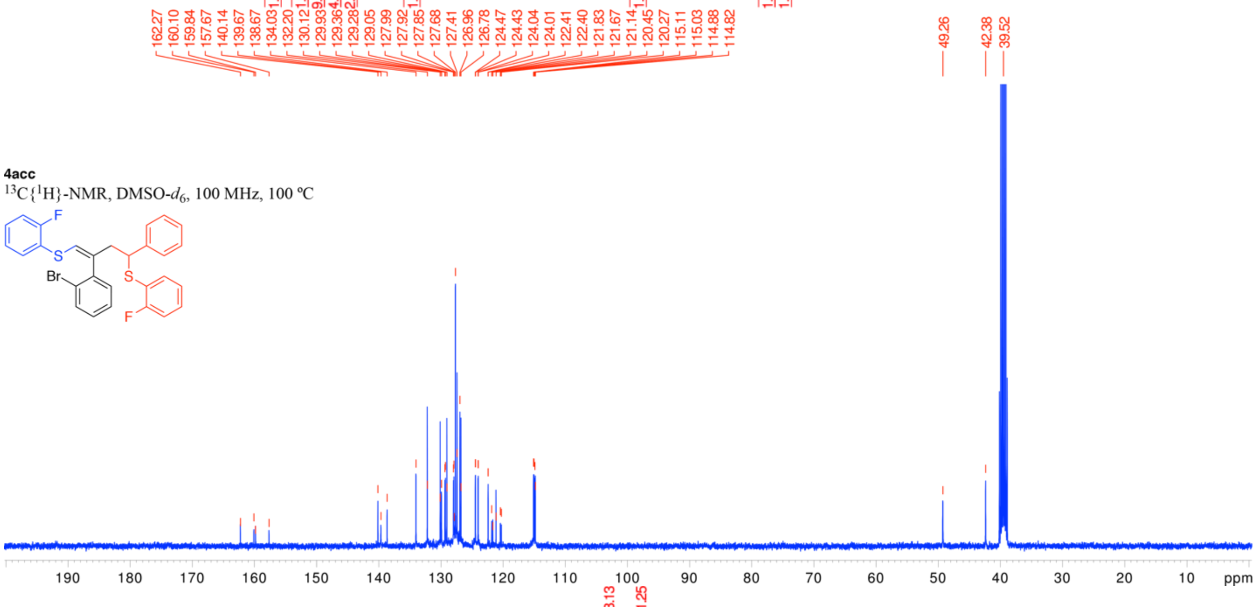

4acc
13C{1H}-NMR, DMSO-d6, 100 MHz, 100 ºC
ppm

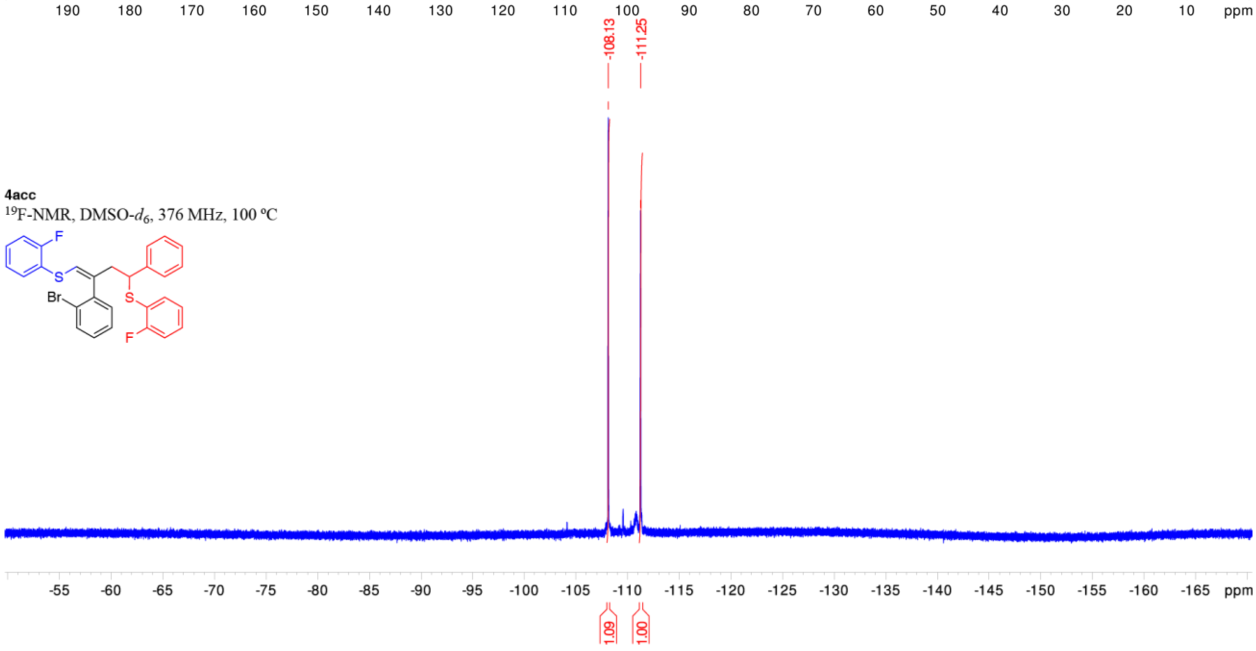

4acc
19F-NMR, DMSO-d6, 376 MHz, 100 ºC
-108.13
-111.25
ppm

4adc

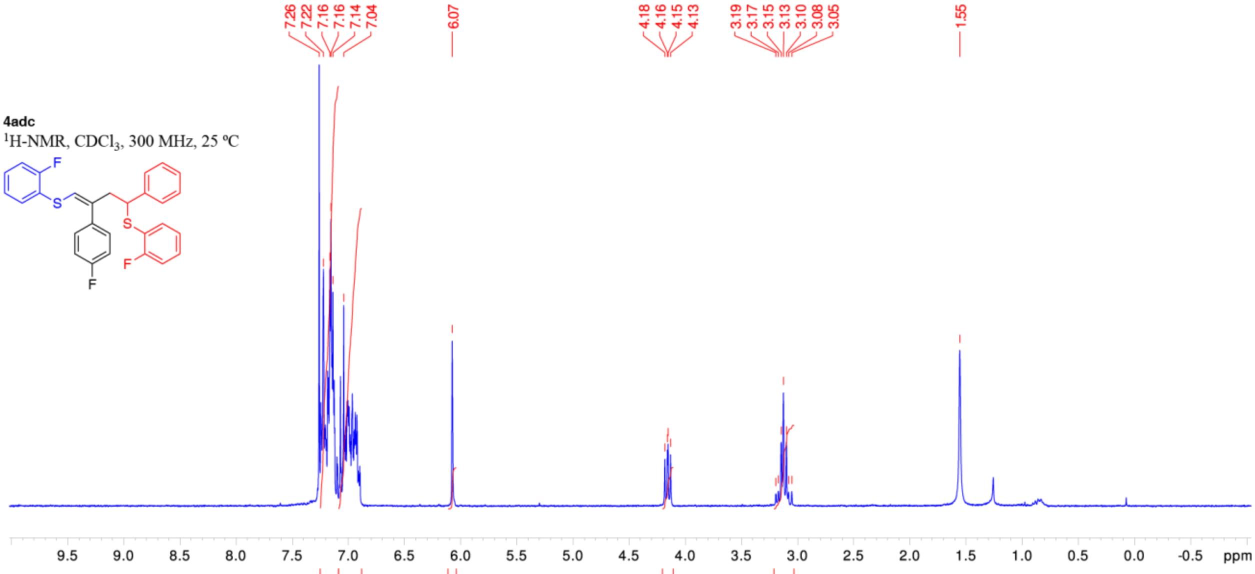
4adc
1H-NMR, CDCl3, 300 MHz, 25 ºC
7.26
7.22
7.16
7.16
7.14
7.04
6.07
4.18
4.16
4.15
4.13
3.19
3.17
3.15
3.13
3.10
3.08
3.05
1.55
10.30
7.32
1.00
1.01
2.04
ppm

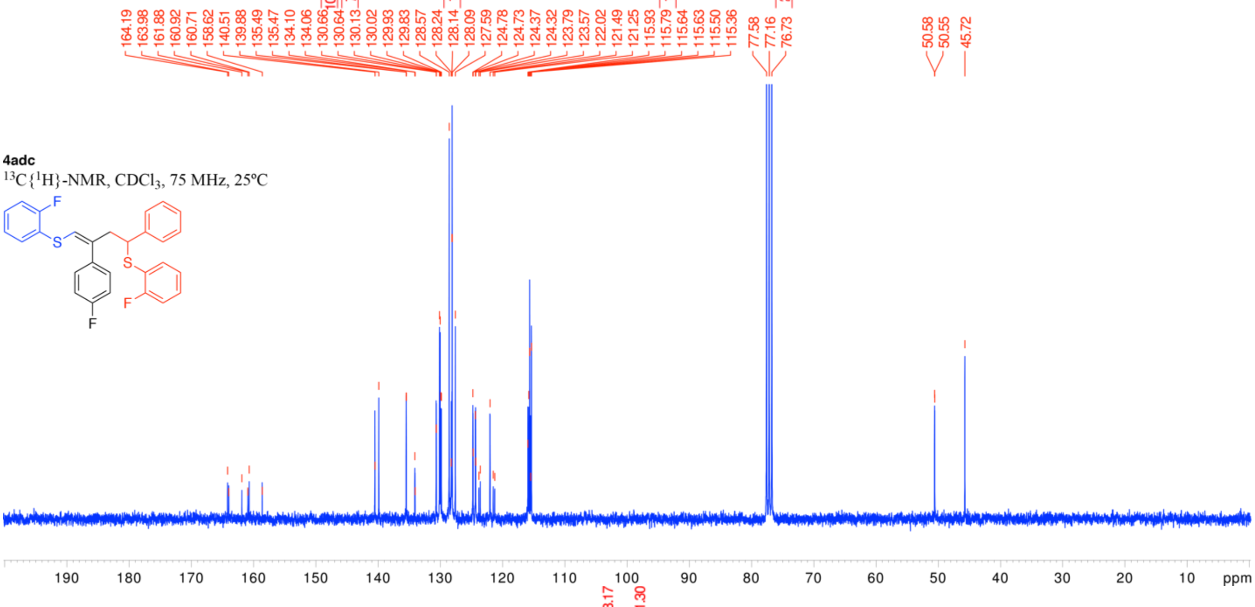
4adc
13C{1H}-NMR, CDCl3, 75 MHz, 25ºC
164.19
163.98
161.88
160.92
160.71
158.62
140.51
139.88
135.49
135.47
134.10
134.06
130.66
130.64
130.13
130.02
129.93
129.83
128.57
128.24
128.14
128.09
127.59
124.78
124.73
124.37
124.32
123.79
123.57
122.02
121.49
121.25
115.93
115.79
115.64
115.63
115.50
115.36
77.58
77.16
76.73
50.58
50.55
45.72
ppm

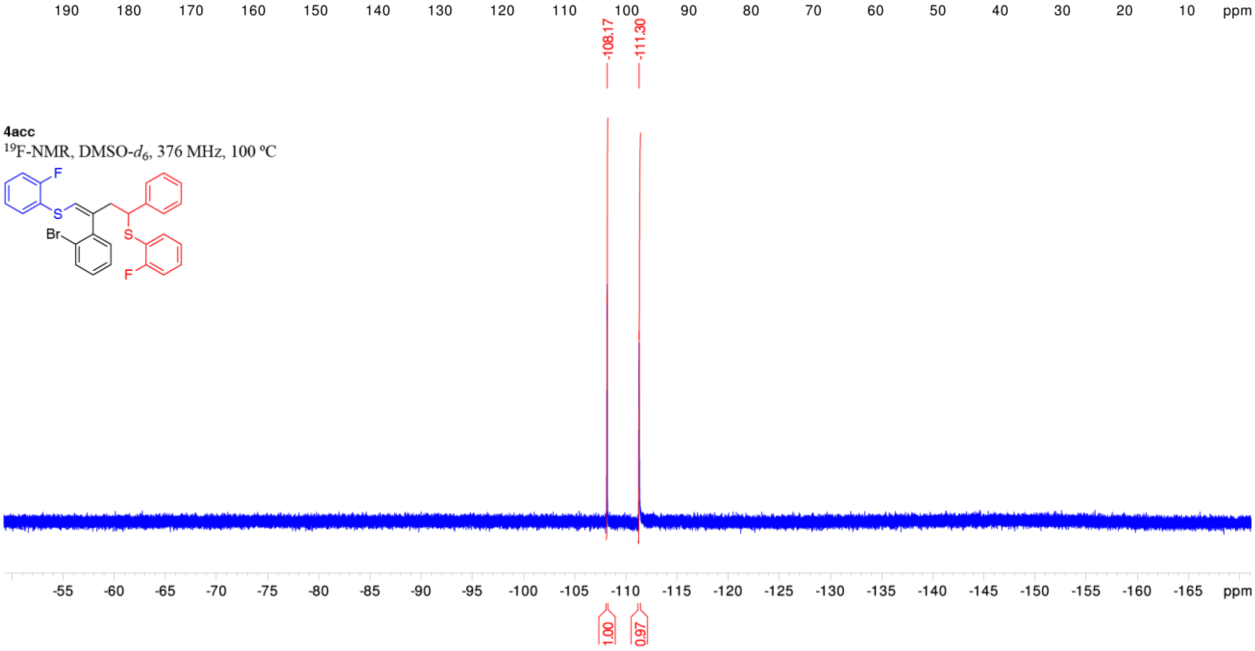
4acc
19F-NMR, DMSO-d6, 376 MHz, 100 ºC
-108.17
-111.30
1.00
0.97
ppm

4aec

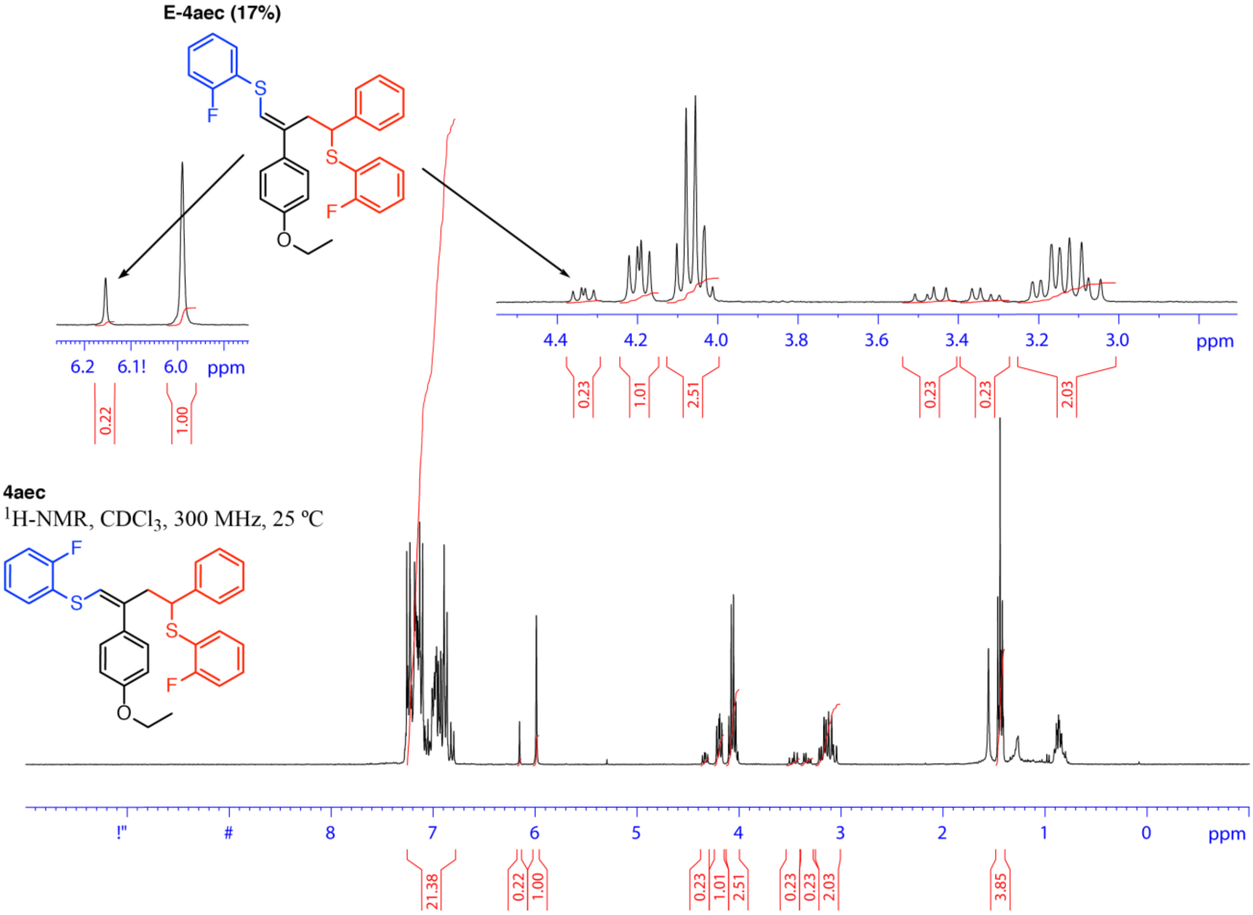

E-4aec (17%)
4aec
1H-NMR, CDCl3, 300 MHz, 25 ºC

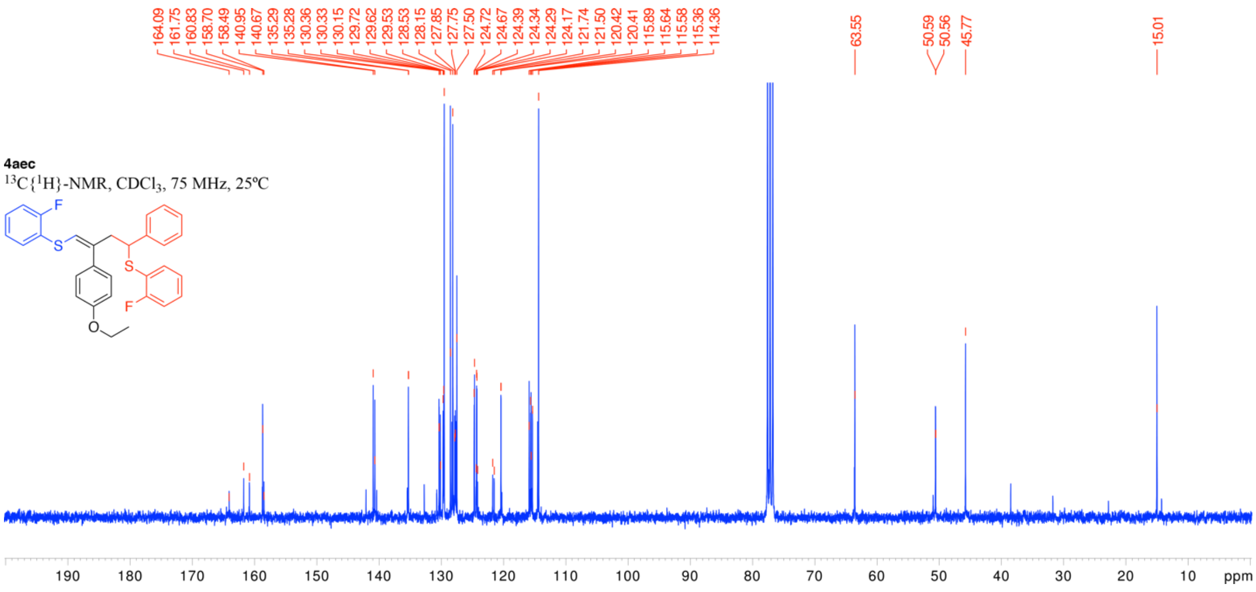

4aec
13C{1H}-NMR, CDCl3, 75 MHz, 25ºC

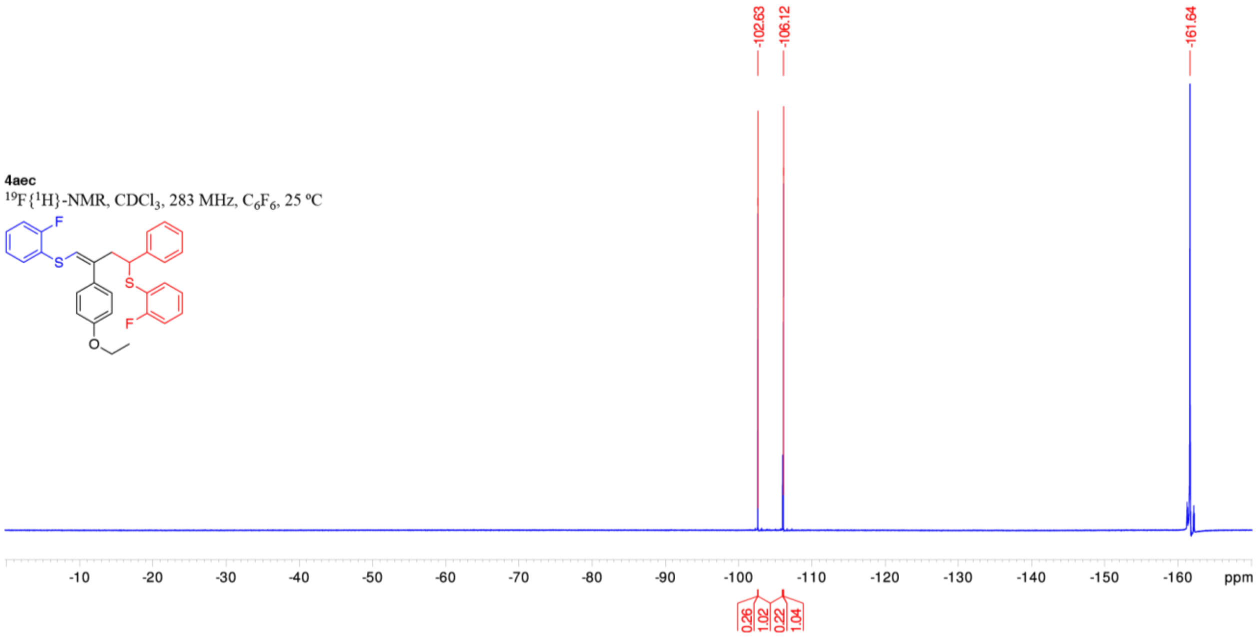
4aec
19F{1H}-NMR, CDCl3, 283 MHz, C6F6, 25 ºC
F
S
S
F
O
-102.63
-106.12
-161.64
-10
-20
-30
-40
-50
-60
-70
-80
-90
-100
-110
-120
-130
-140
-150
-160
ppm
0.26
1.02
0.22
1.04

4aad

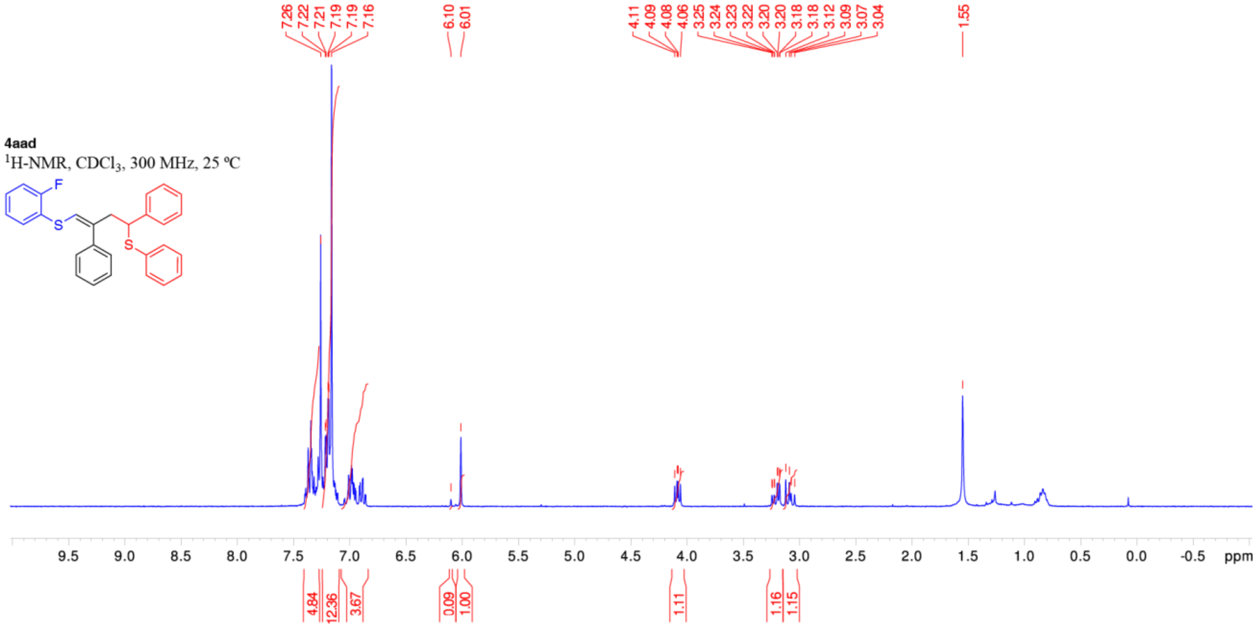
4aad
^{1}H-NMR, $CDCl_3$, 300 MHz, 25 ºC
F
S
S
ppm

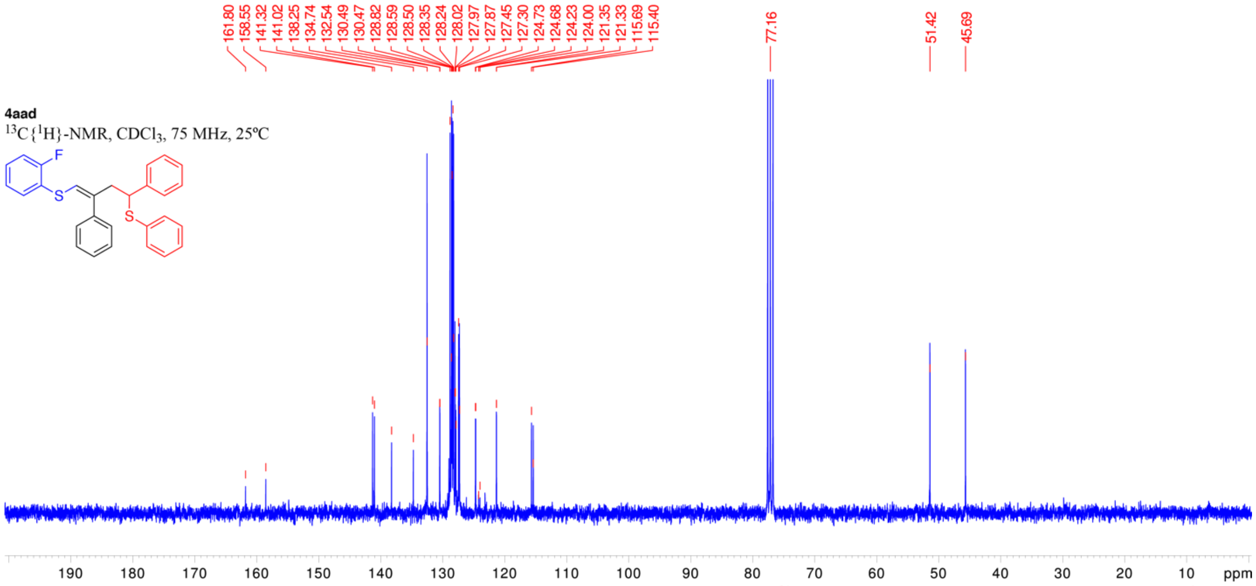
4aad
$^{13}C\{^1H\}$-NMR, $CDCl_3$, 75 MHz, 25ºC
F
S
S
ppm

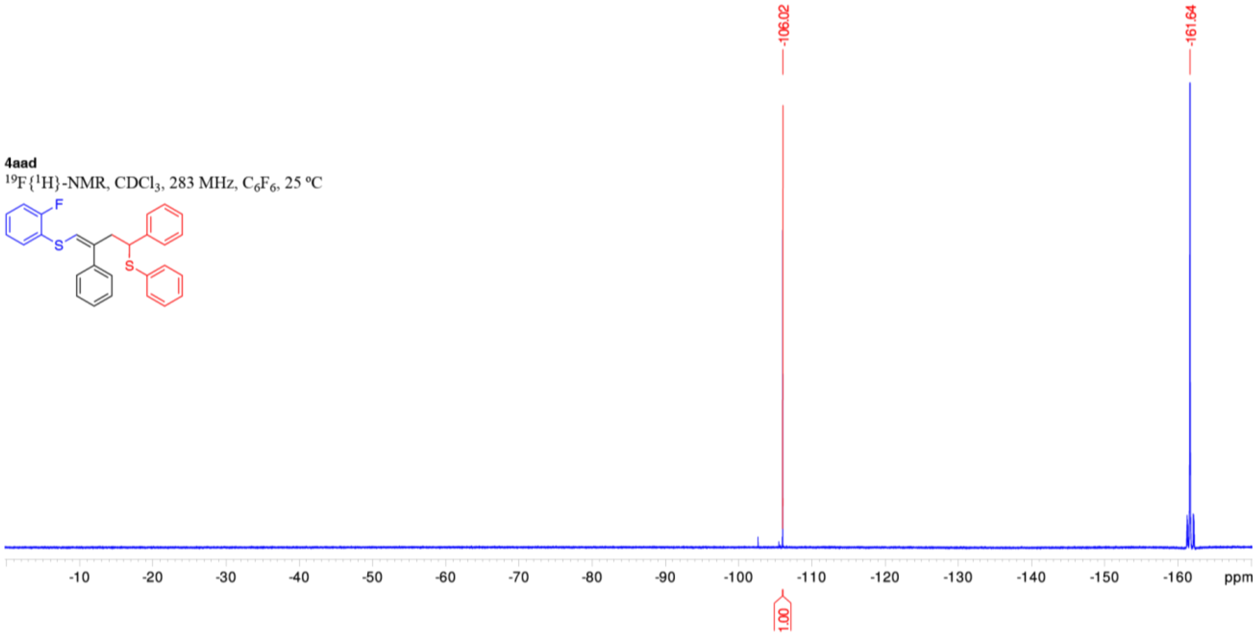
4aad
$^{19}F\{^1H\}$-NMR, $CDCl_3$, 283 MHz, C_6F_6, 25 ºC
F
S
S
-106.02
-161.64
ppm

4abd

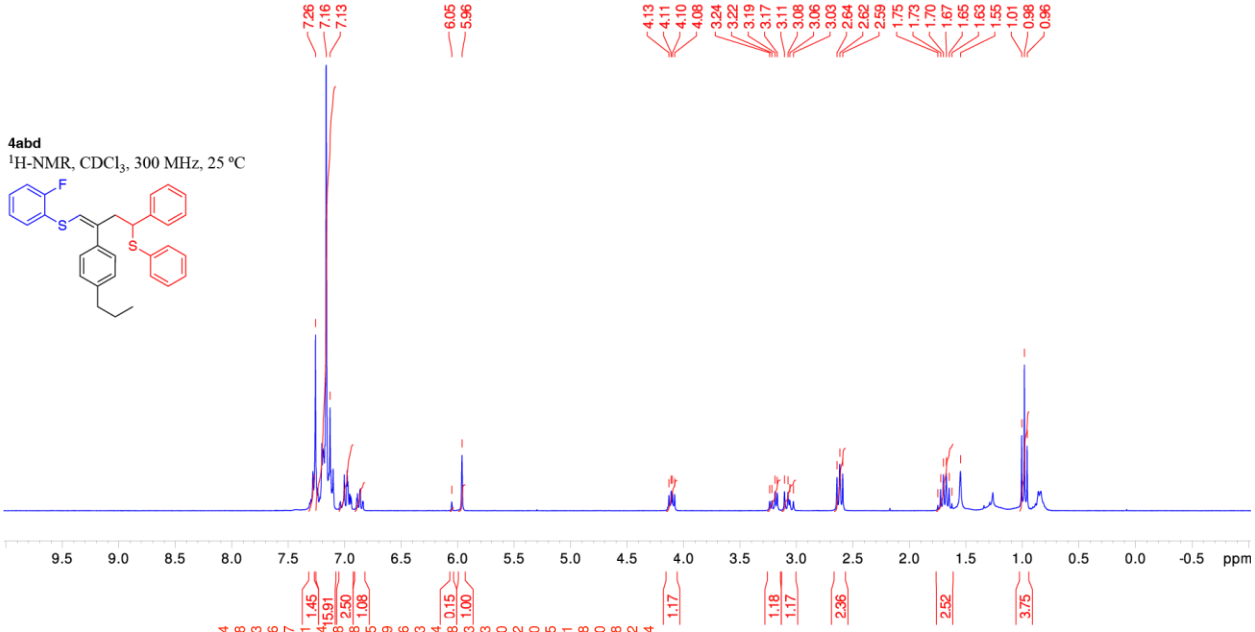

4abd
^{1}H-NMR, $CDCl_3$, 300 MHz, 25 ºC

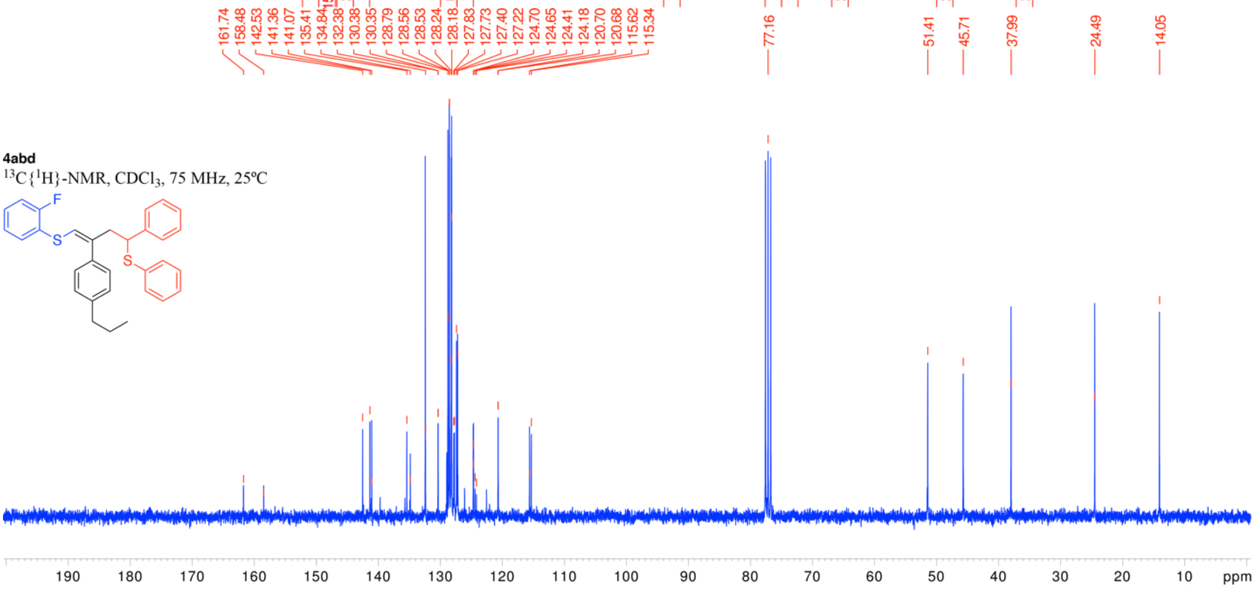

4abd
^{13}C{^{1}H}-NMR, $CDCl_3$, 75 MHz, 25ºC

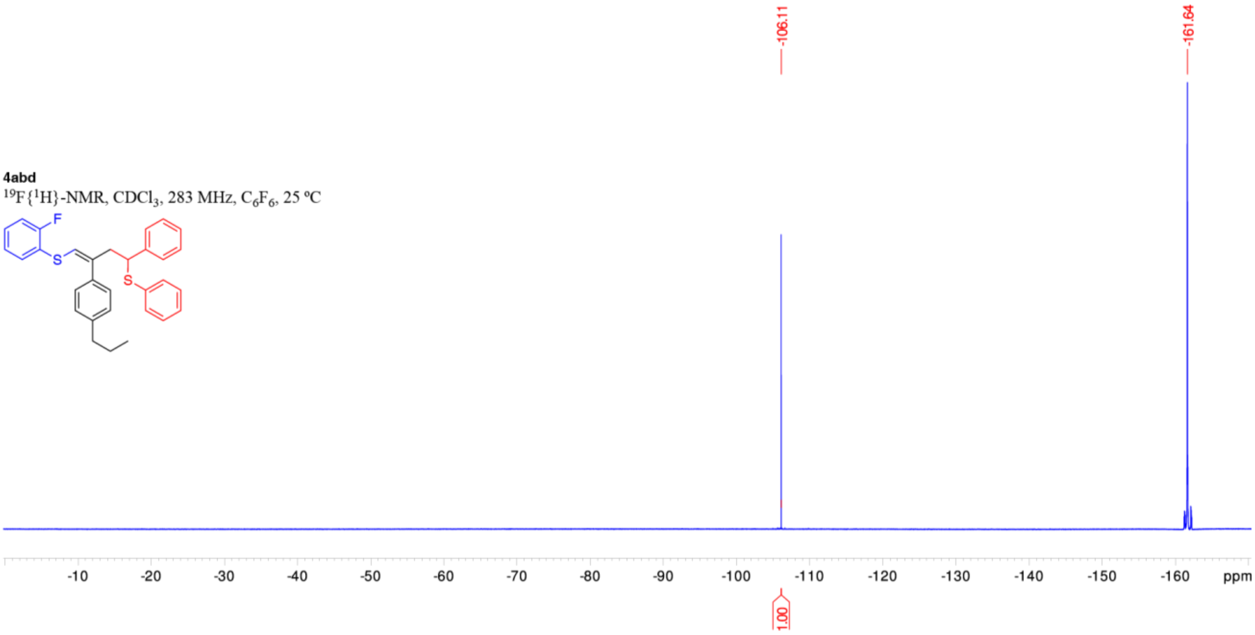

4abd
^{19}F{^{1}H}-NMR, $CDCl_3$, 283 MHz, C_6F_6, 25 ºC

4acd

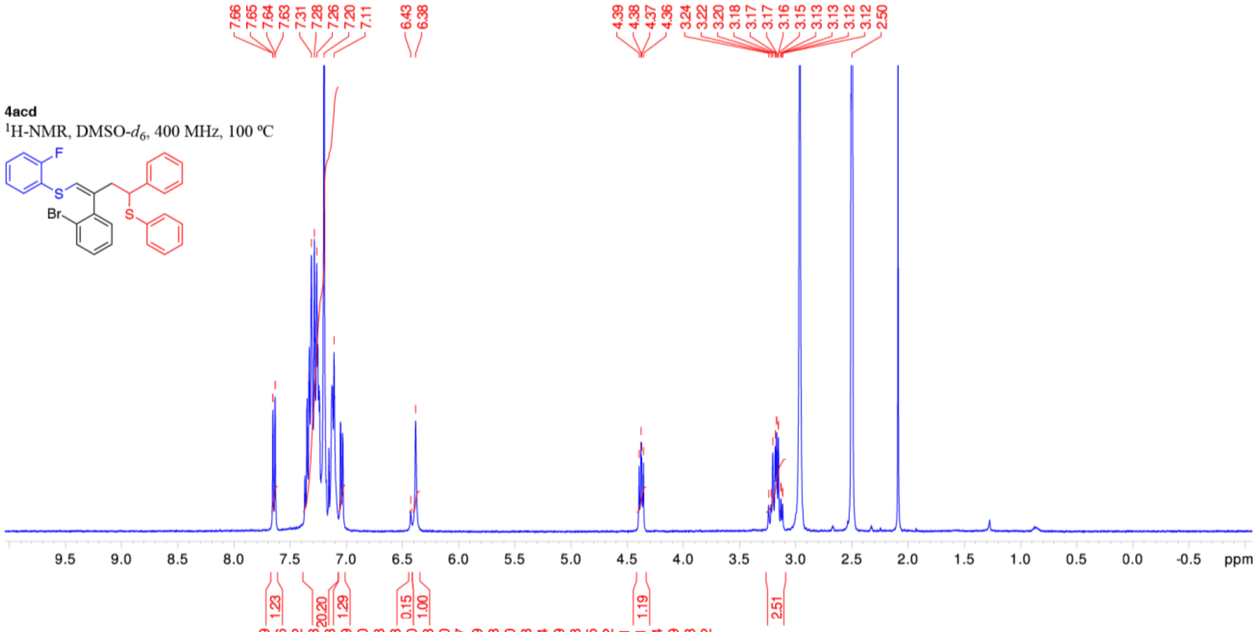
4acd
1H-NMR, DMSO-d6, 400 MHz, 100 ºC
ppm

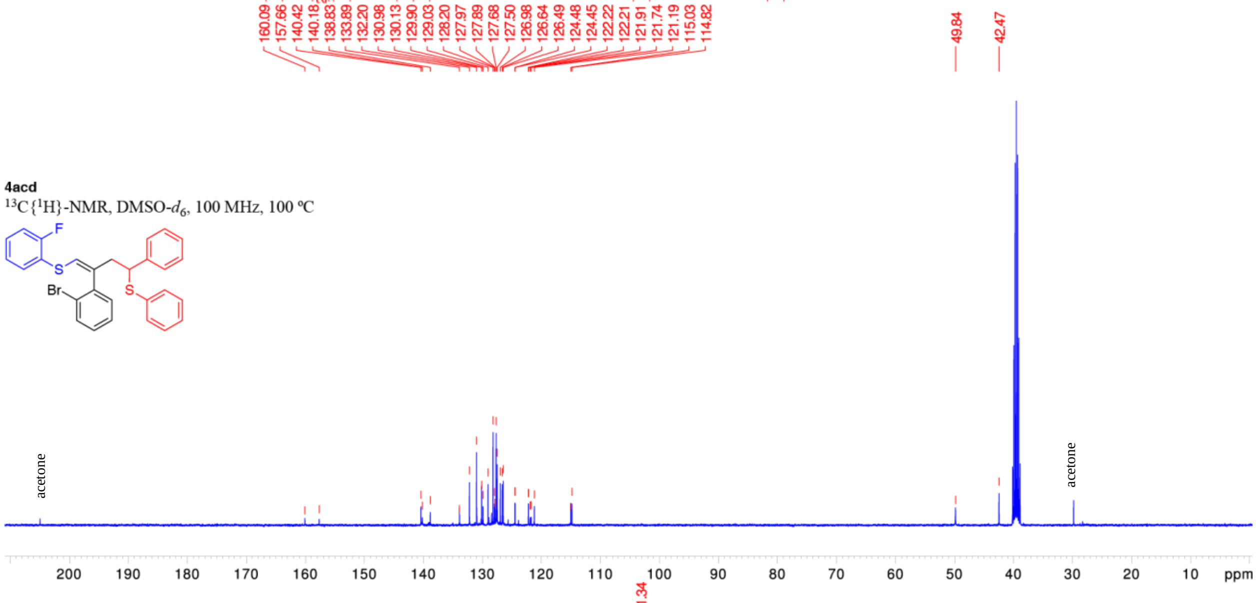
4acd
13C{1H}-NMR, DMSO-d6, 100 MHz, 100 ºC
acetone
acetone
ppm

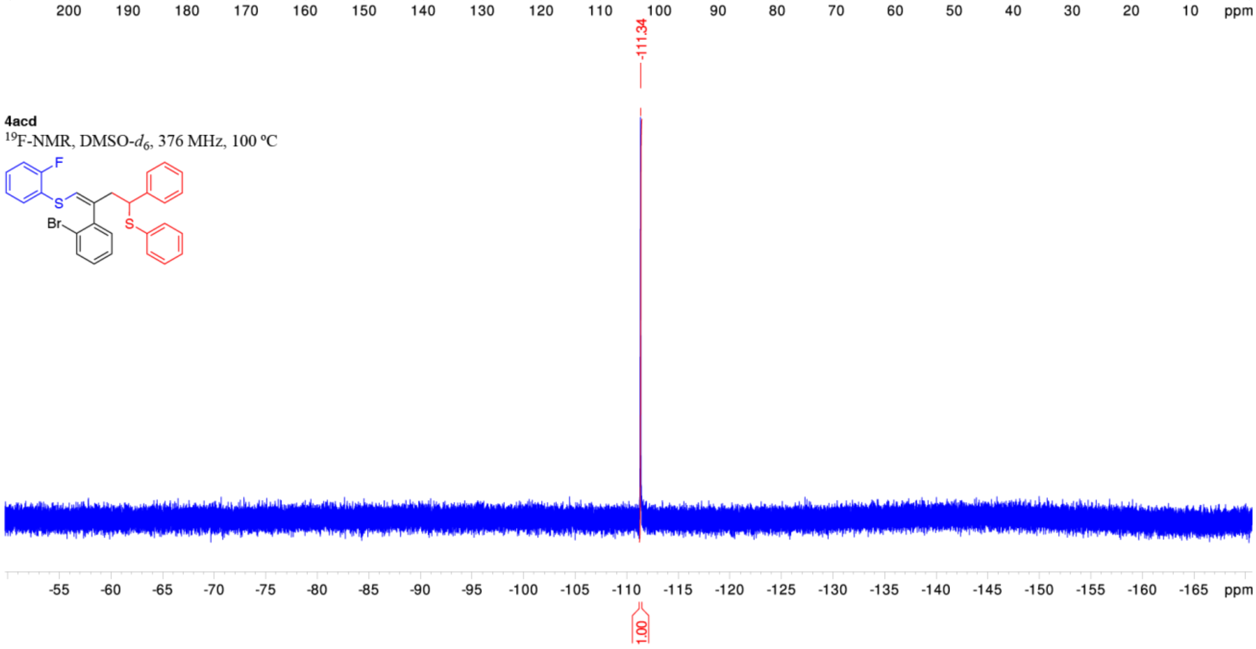
4acd
19F-NMR, DMSO-d6, 376 MHz, 100 ºC
-111.34
ppm

4add

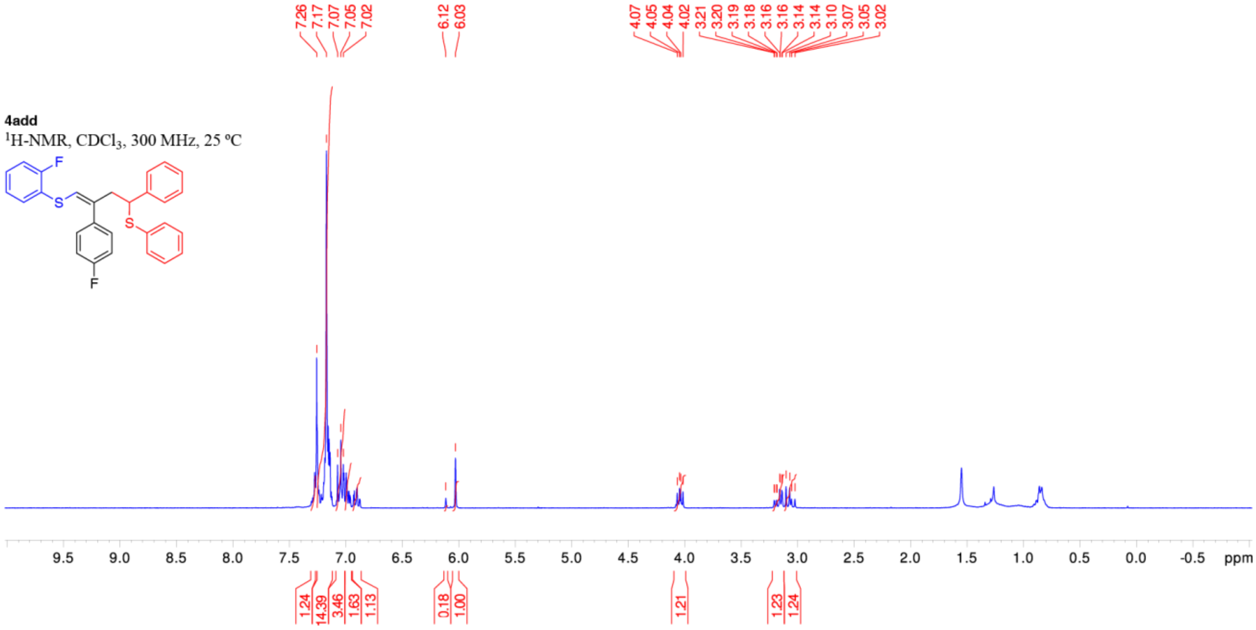

4add
^{1}H-NMR, CDCl$_3$, 300 MHz, 25 ºC

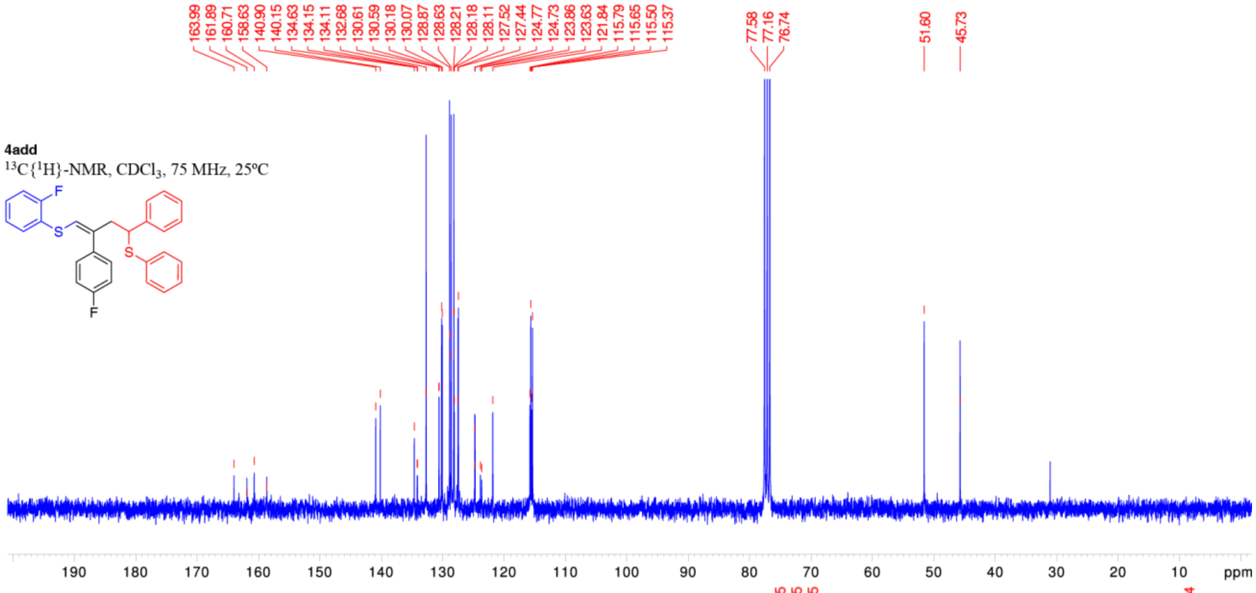

4add
^{13}C{^{1}H}-NMR, CDCl$_3$, 75 MHz, 25ºC

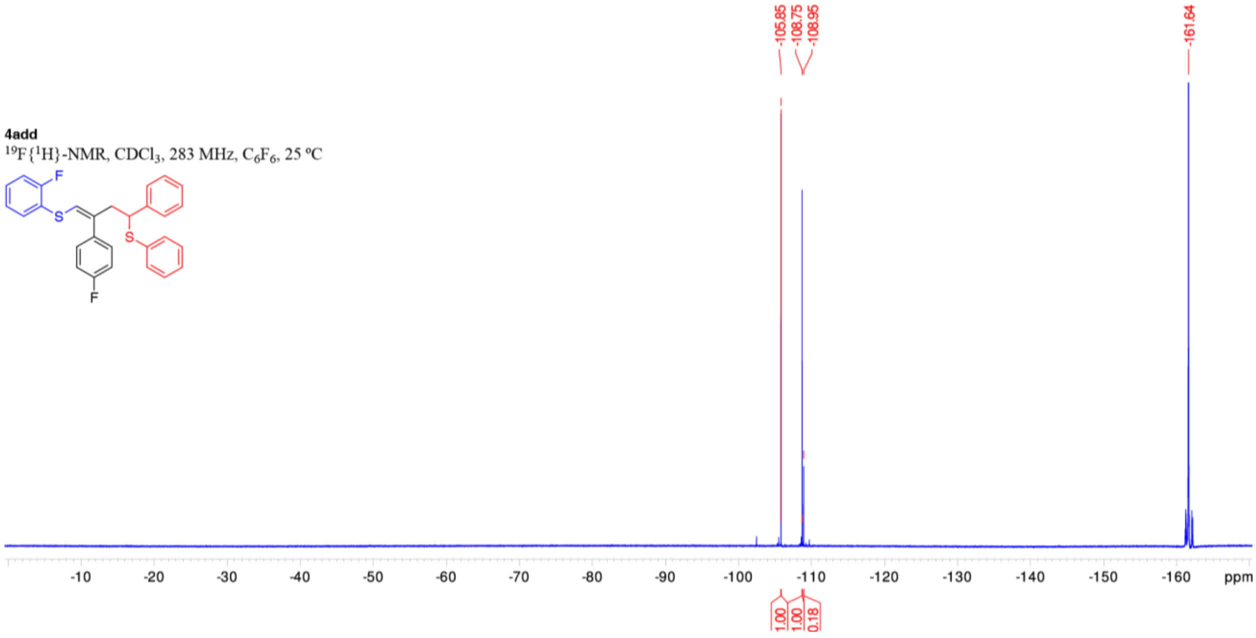

4add
^{19}F{^{1}H}-NMR, CDCl$_3$, 283 MHz, C$_6$F$_6$, 25 ºC

4aed

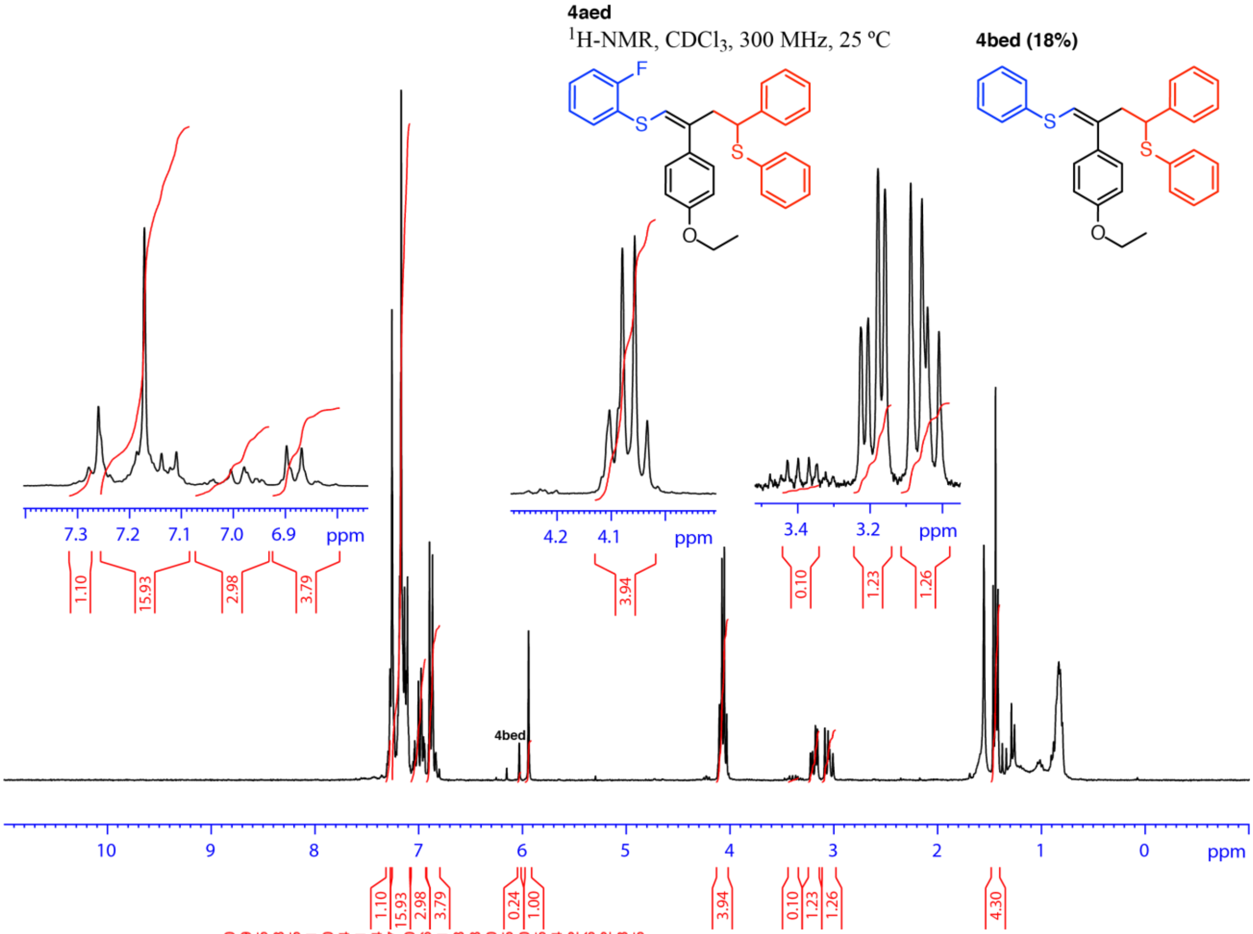

4aed
^{1}H-NMR, $CDCl_3$, 300 MHz, 25 ºC
4bed (18%)
F
S
O
4bed
ppm

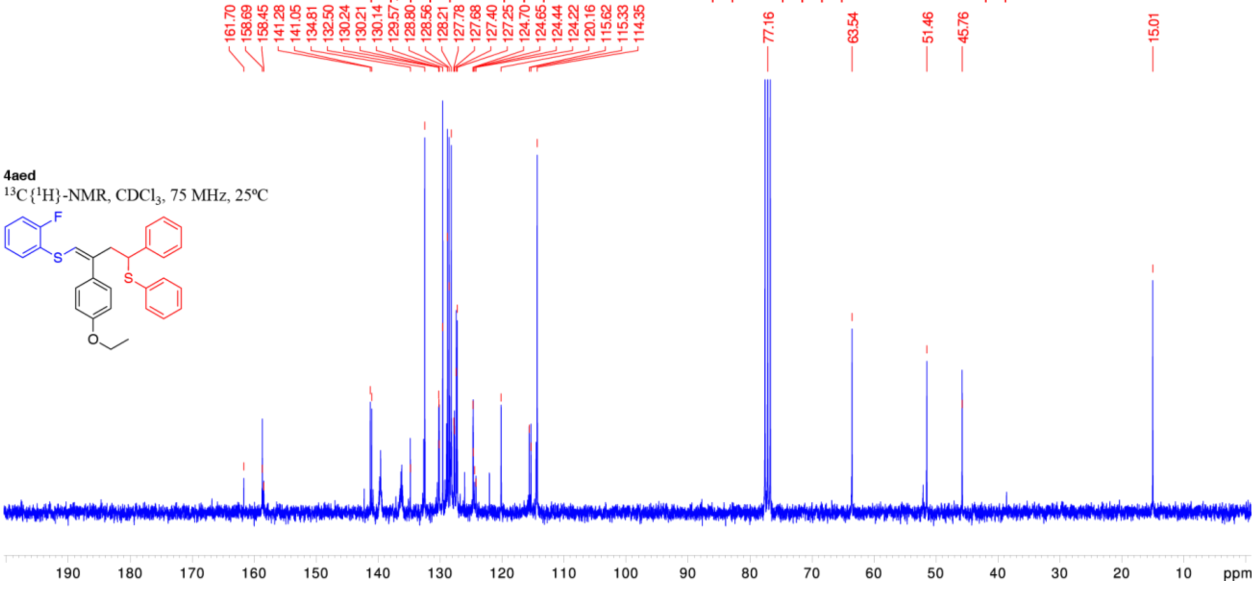

4aed
$^{13}C\{^1H\}$-NMR, $CDCl_3$, 75 MHz, 25ºC
F
S
O
ppm

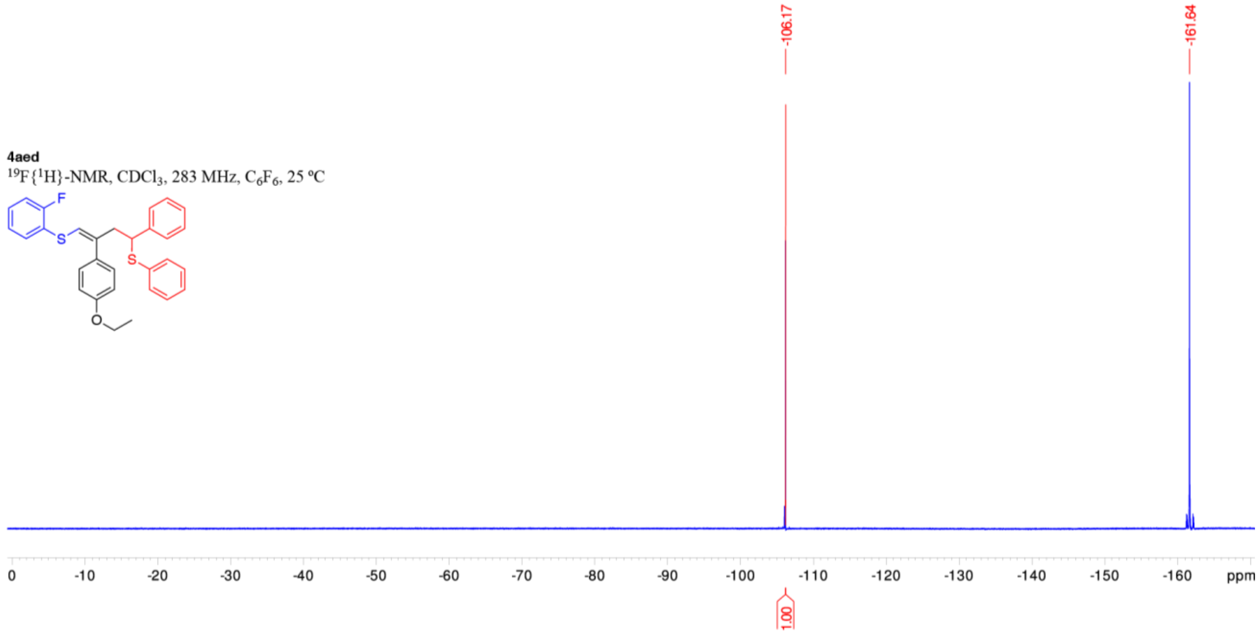
4aed
$^{19}F\{^{1}H\}$-NMR, $CDCl_3$, 283 MHz, C_6F_6, 25 ºC
F
S
S
O
-106.17
-161.64
0
-10
-20
-30
-40
-50
-60
-70
-80
-90
-100
-110
-120
-130
-140
-150
-160
ppm
1.00

4afd

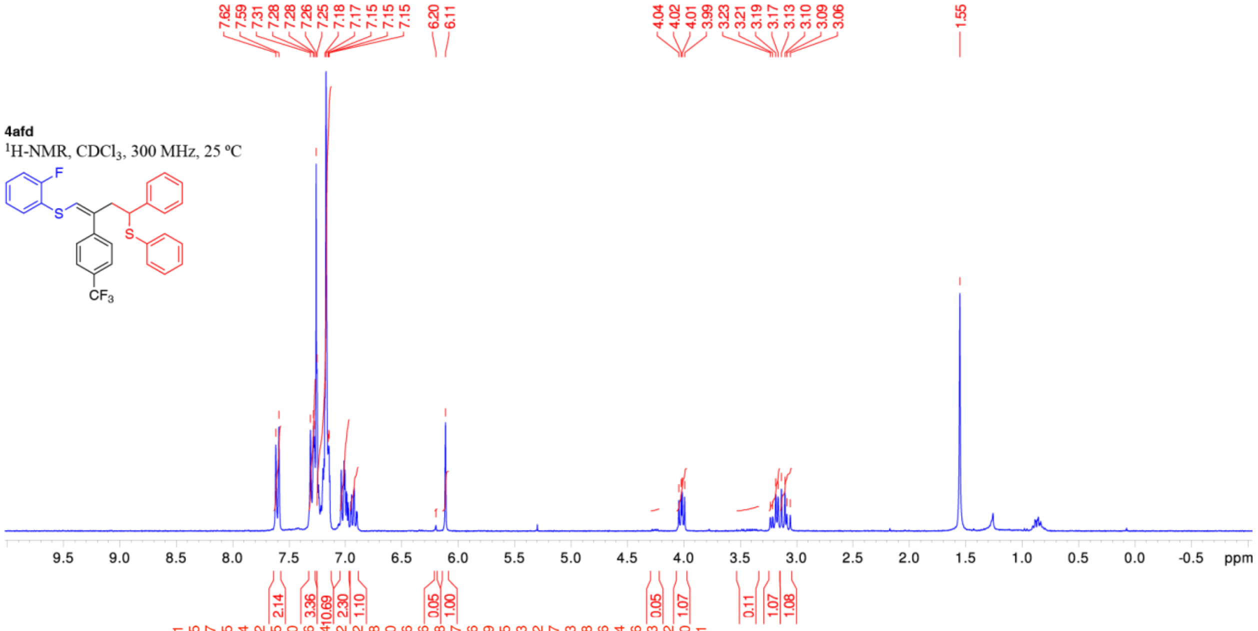
4afd
^{1}H-NMR, $CDCl_3$, 300 MHz, 25 ºC
F
S
S
CF_3
7.62 7.59 7.31 7.28 7.28 7.26 7.25 7.18 7.17 7.15 7.15 7.15 6.20 6.11
4.04 4.02 4.01 3.99 3.23 3.21 3.19 3.17 3.13 3.10 3.09 3.06
1.55
2.14 3.36 10.69 2.30 1.10 0.05 1.00 0.05 1.07 0.11 1.07 1.08
9.5 9.0 8.5 8.0 7.5 7.0 6.5 6.0 5.5 5.0 4.5 4.0 3.5 3.0 2.5 2.0 1.5 1.0 0.5 0.0 -0.5 ppm

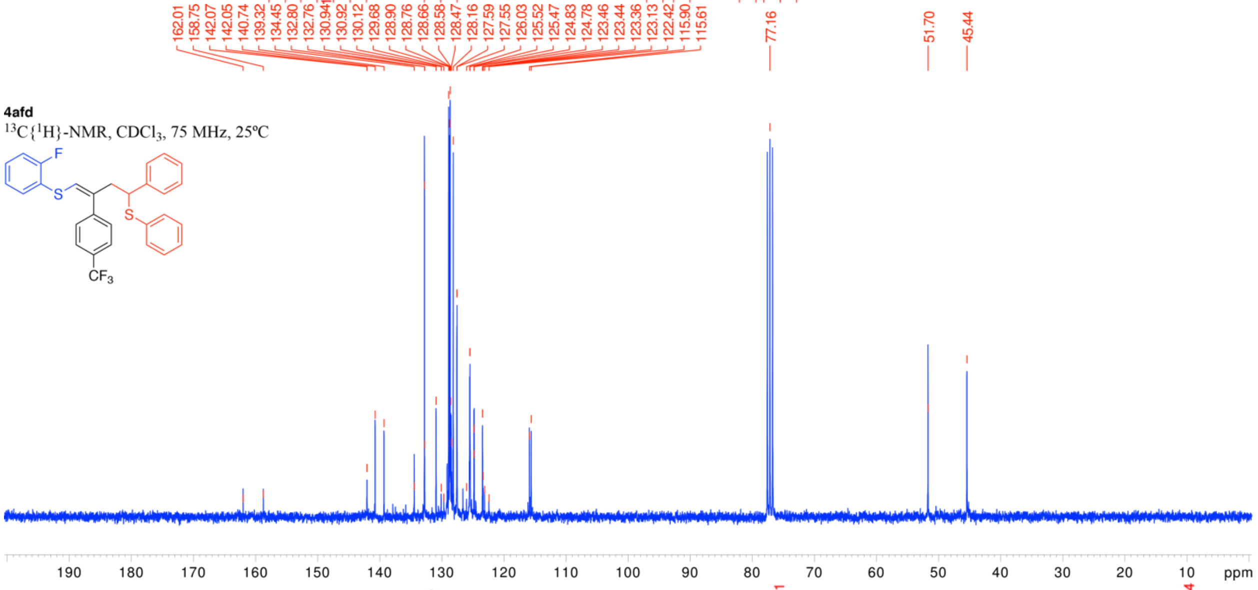
4afd
^{13}C{^{1}H}-NMR, $CDCl_3$, 75 MHz, 25ºC
F
S
S
CF_3
162.01 158.75 142.07 142.05 140.74 139.32 134.45 132.80 132.76 130.94 130.92 130.12 129.68 128.90 128.76 128.66 128.58 128.47 128.16 127.59 127.55 126.03 125.52 125.47 124.83 124.78 123.46 123.44 123.36 123.13 122.42 115.90 115.61
77.16
51.70
45.44
190 180 170 160 150 140 130 120 110 100 90 80 70 60 50 40 30 20 10 ppm

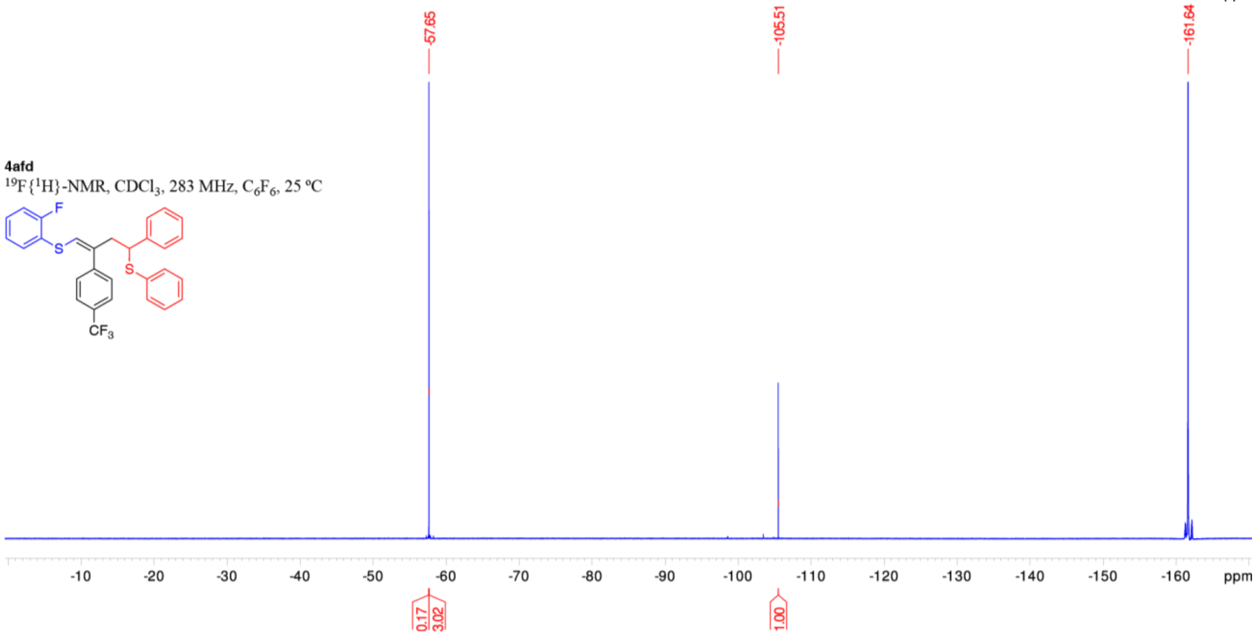
-57.65
-105.51
-161.64
4afd
^{19}F{^{1}H}-NMR, $CDCl_3$, 283 MHz, C_6F_6, 25 ºC
F
S
S
CF_3
-10 -20 -30 -40 -50 -60 -70 -80 -90 -100 -110 -120 -130 -140 -150 -160 ppm
0.17 3.02
1.00

4bbd

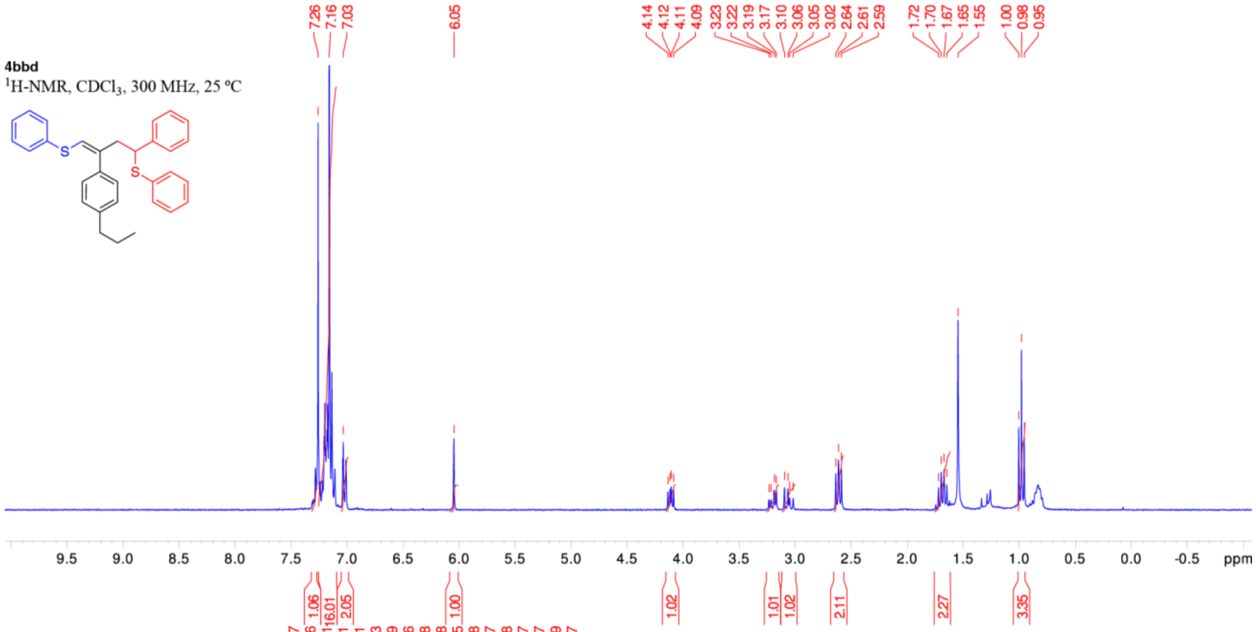

4bbd
¹H-NMR, CDCl₃, 300 MHz, 25 ºC
S
S
7.26
7.16
7.03
6.05
4.14
4.12
4.11
4.09
3.23
3.22
3.19
3.17
3.10
3.06
3.05
3.02
2.64
2.61
2.59
1.72
1.70
1.67
1.65
1.55
1.00
0.98
0.95
1.06
16.01
2.05
1.00
1.02
1.01
1.02
2.11
2.27
3.35
9.5
9.0
8.5
8.0
7.5
7.0
6.5
6.0
5.5
5.0
4.5
4.0
3.5
3.0
2.5
2.0
1.5
1.0
0.5
0.0
-0.5
ppm

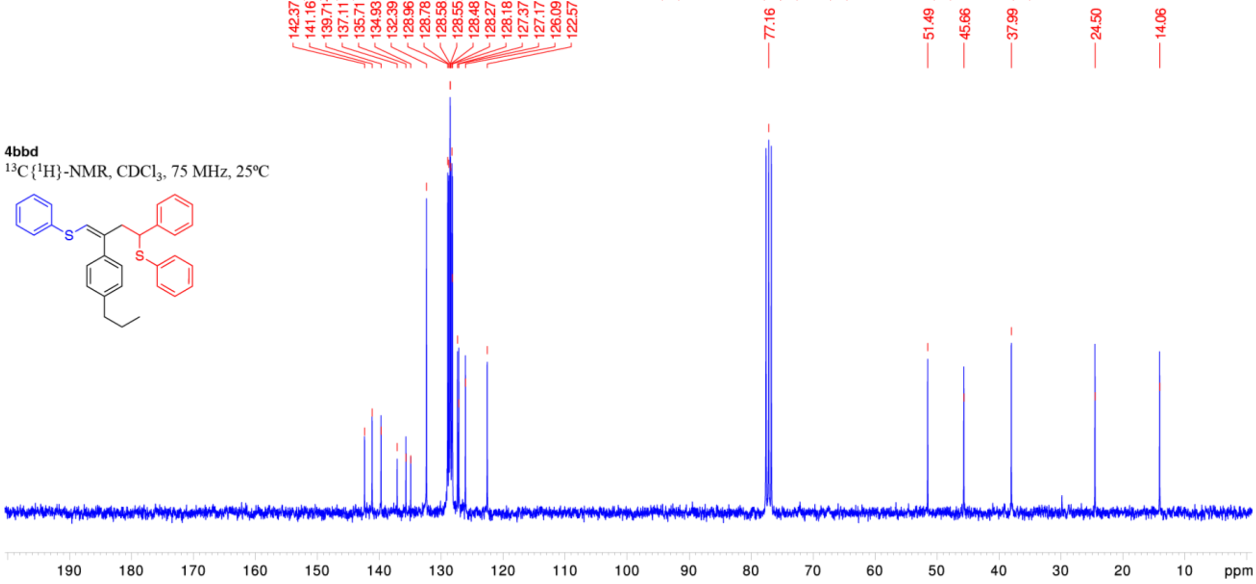

4bbd
¹³C{¹H}-NMR, CDCl₃, 75 MHz, 25ºC
S
S
142.37
141.16
139.71
137.11
135.71
134.93
132.39
128.96
128.78
128.58
128.55
128.48
128.27
128.18
127.37
127.17
126.09
122.57
77.16
51.49
45.66
37.99
24.50
14.06
190
180
170
160
150
140
130
120
110
100
90
80
70
60
50
40
30
20
10
ppm

E-4bbd

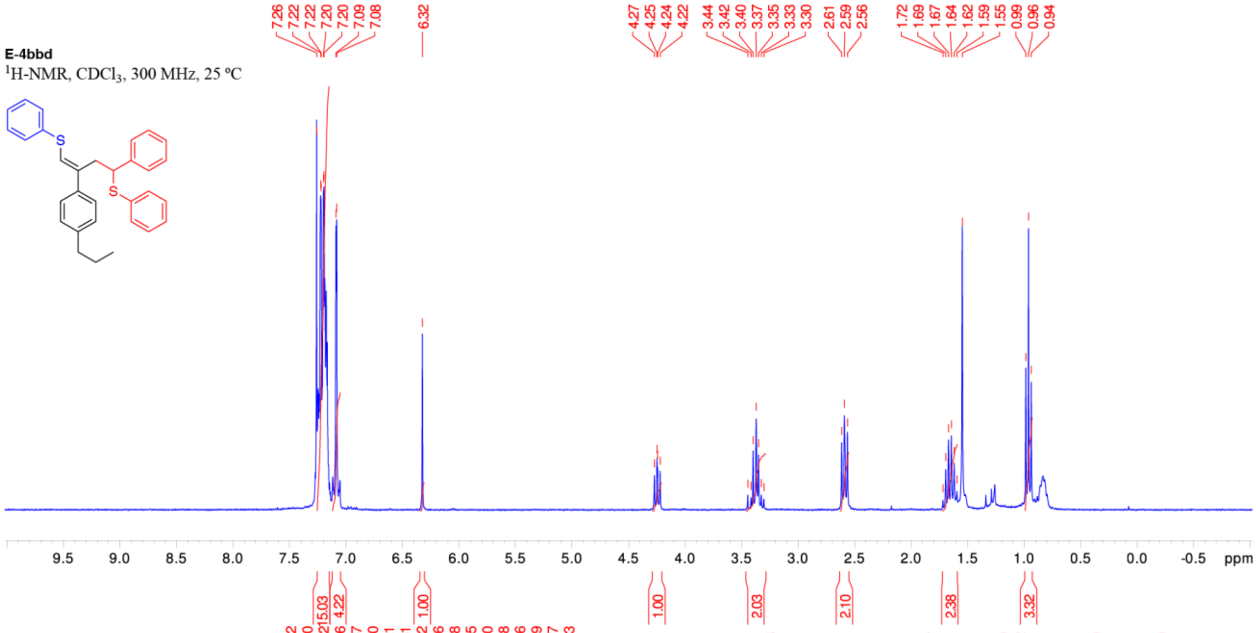

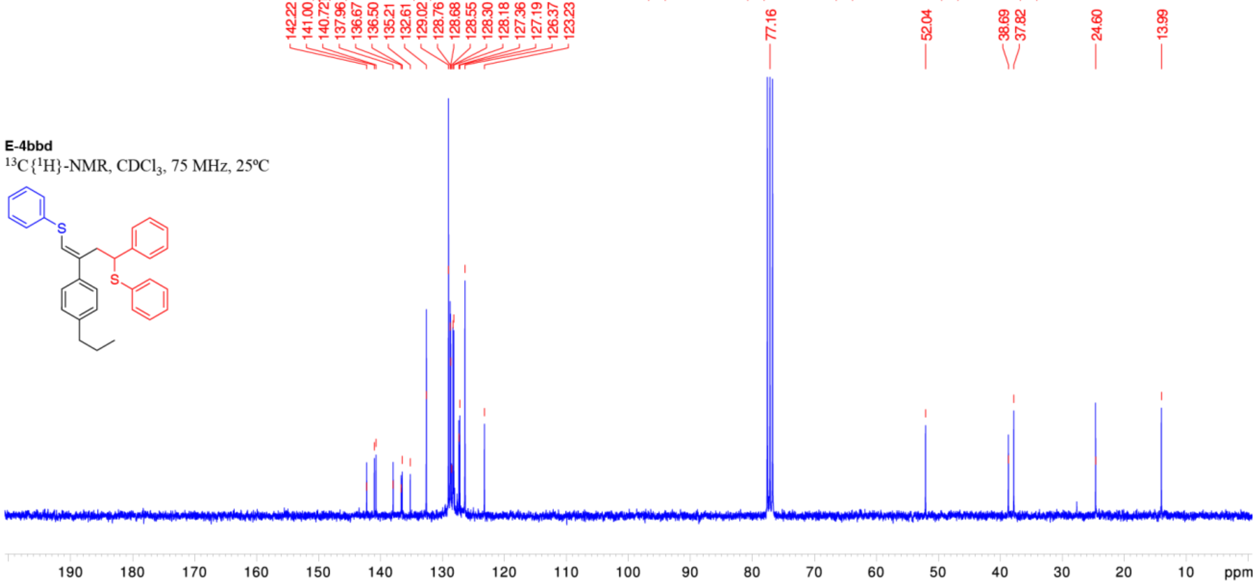

4cbd

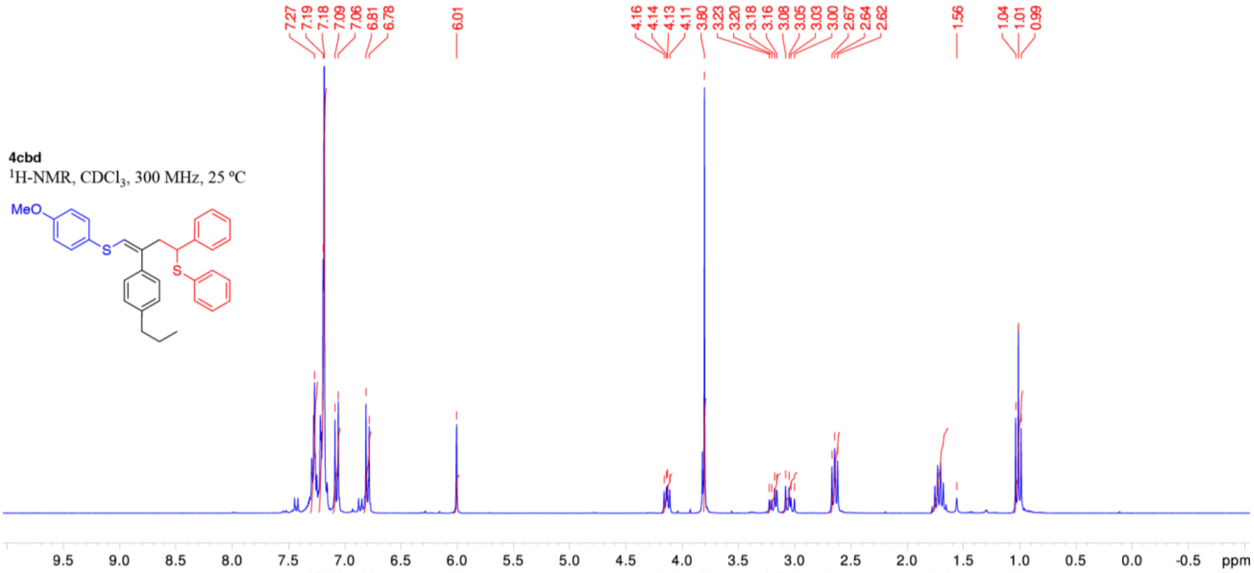
4cbd
1H-NMR, CDCl3, 300 MHz, 25 ºC
MeO
S
S
ppm

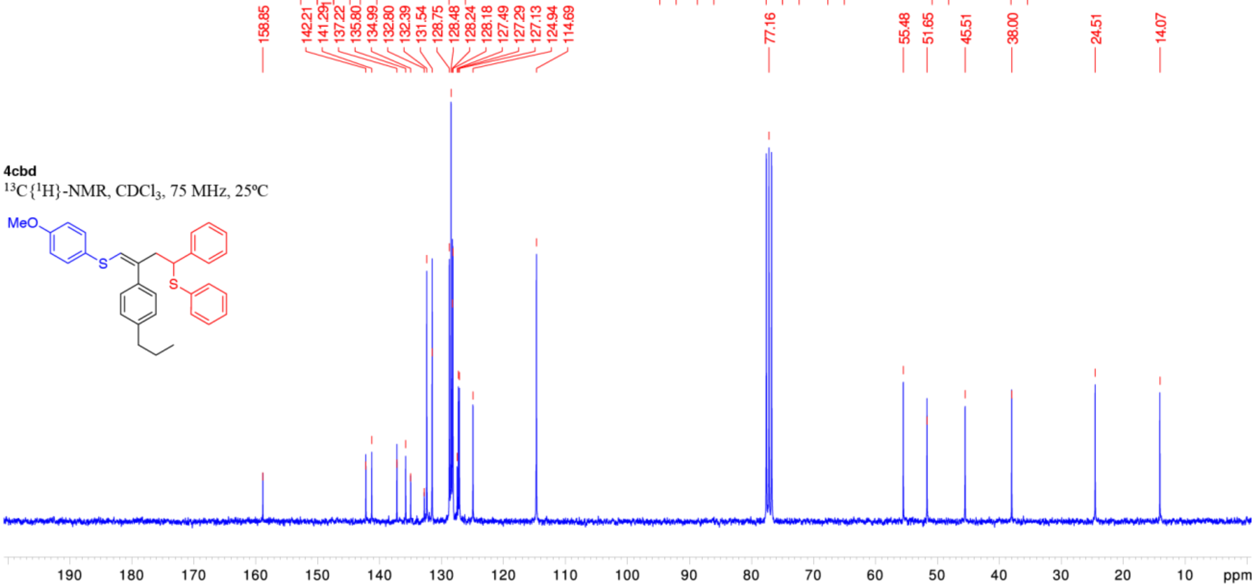
4cbd
13C{1H}-NMR, CDCl3, 75 MHz, 25ºC
MeO
S
S
ppm

4dbd

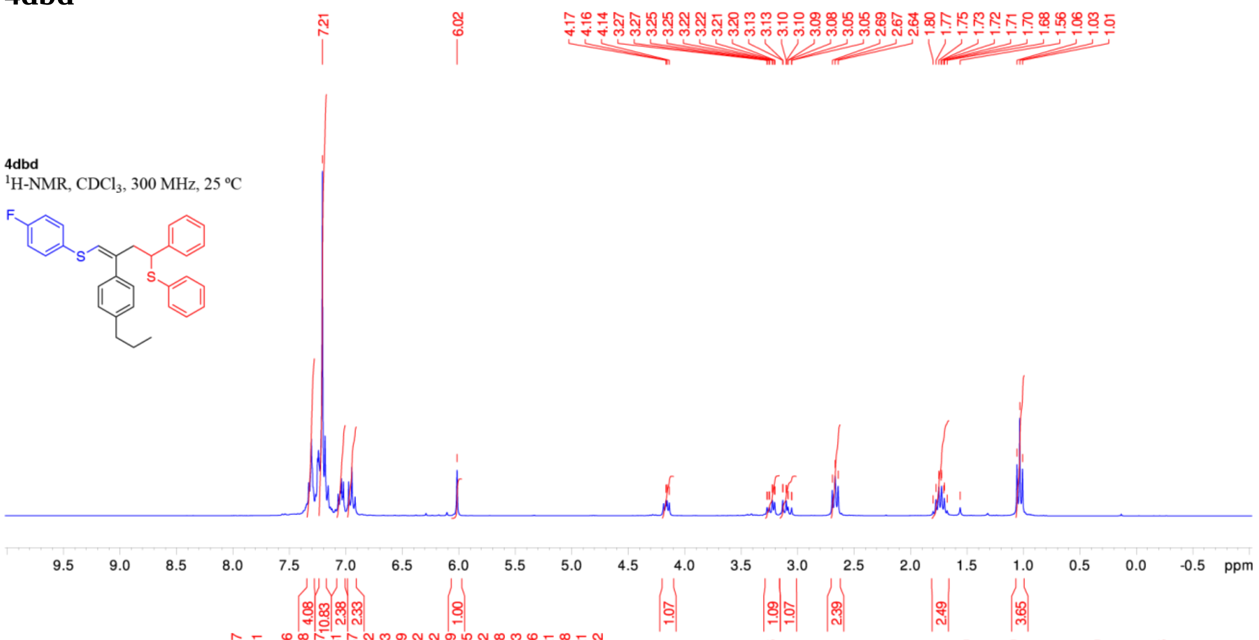
4dbd
1H-NMR, CDCl3, 300 MHz, 25 ºC
F
S
S
7.21
6.02
4.17 4.16 4.14 3.27 3.27 3.25 3.25 3.22 3.22 3.21 3.20 3.13 3.13 3.10 3.10 3.09 3.08 3.05 3.05 2.69 2.67 2.64 1.80 1.77 1.75 1.73 1.72 1.71 1.70 1.68 1.56 1.06 1.03 1.01
9.5 9.0 8.5 8.0 7.5 7.0 6.5 6.0 5.5 5.0 4.5 4.0 3.5 3.0 2.5 2.0 1.5 1.0 0.5 0.0 -0.5 ppm
4.08 10.83 2.38 2.33 1.00 1.07 1.09 1.07 2.39 2.49 3.65

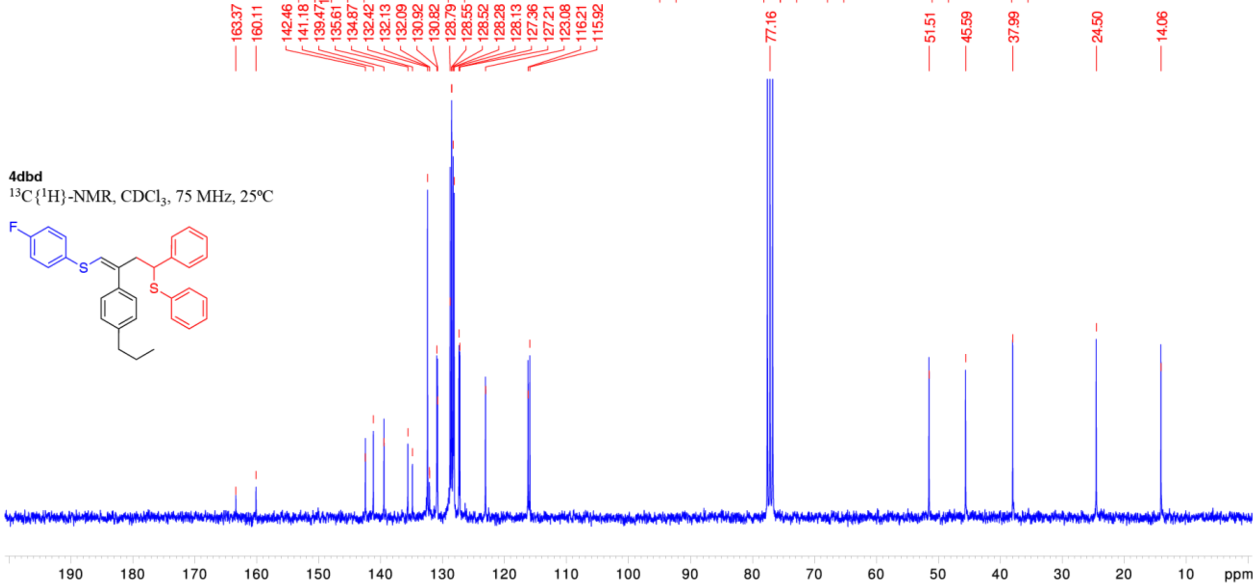
4dbd
13C{1H}-NMR, CDCl3, 75 MHz, 25ºC
F
S
S
163.37 160.11
142.46 141.18 139.47 135.61 134.87 132.42 132.13 132.09 130.92 130.82 128.79 128.55 128.52 128.28 128.13 127.36 127.21 123.08 116.21 115.92
77.16
51.51 45.59 37.99 24.50 14.06
190 180 170 160 150 140 130 120 110 100 90 80 70 60 50 40 30 20 10 ppm

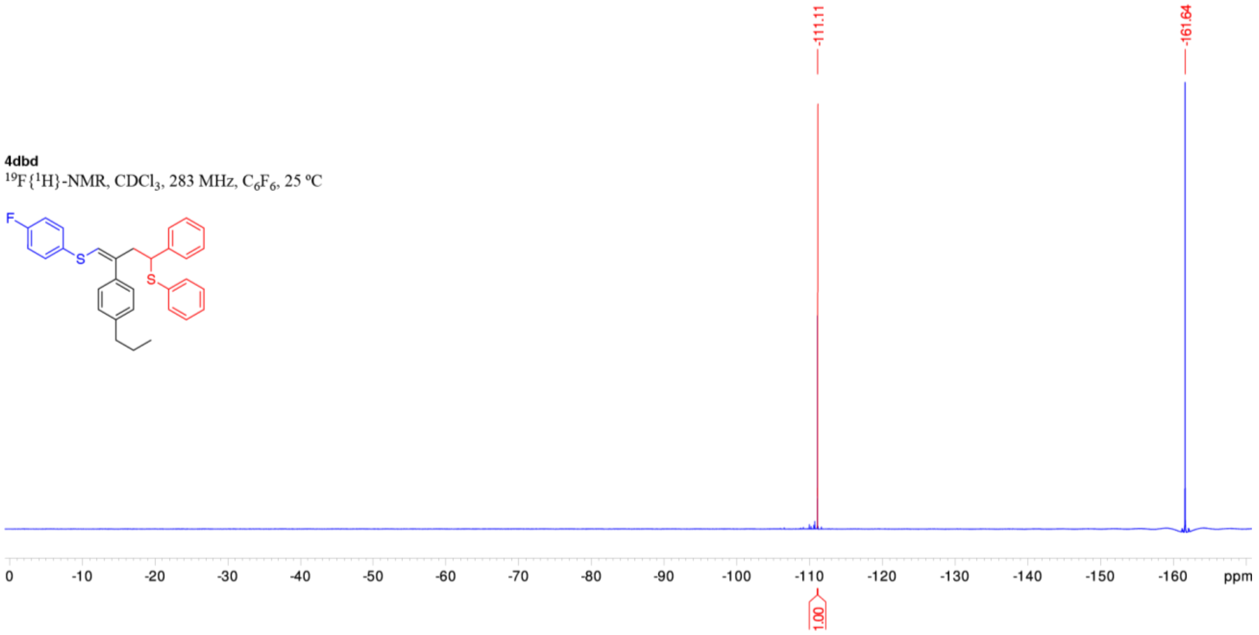
-111.11
-161.64
4dbd
19F{1H}-NMR, CDCl3, 283 MHz, C6F6, 25 ºC
F
S
S
0 -10 -20 -30 -40 -50 -60 -70 -80 -90 -100 -110 -120 -130 -140 -150 -160 ppm
1.00

4baa

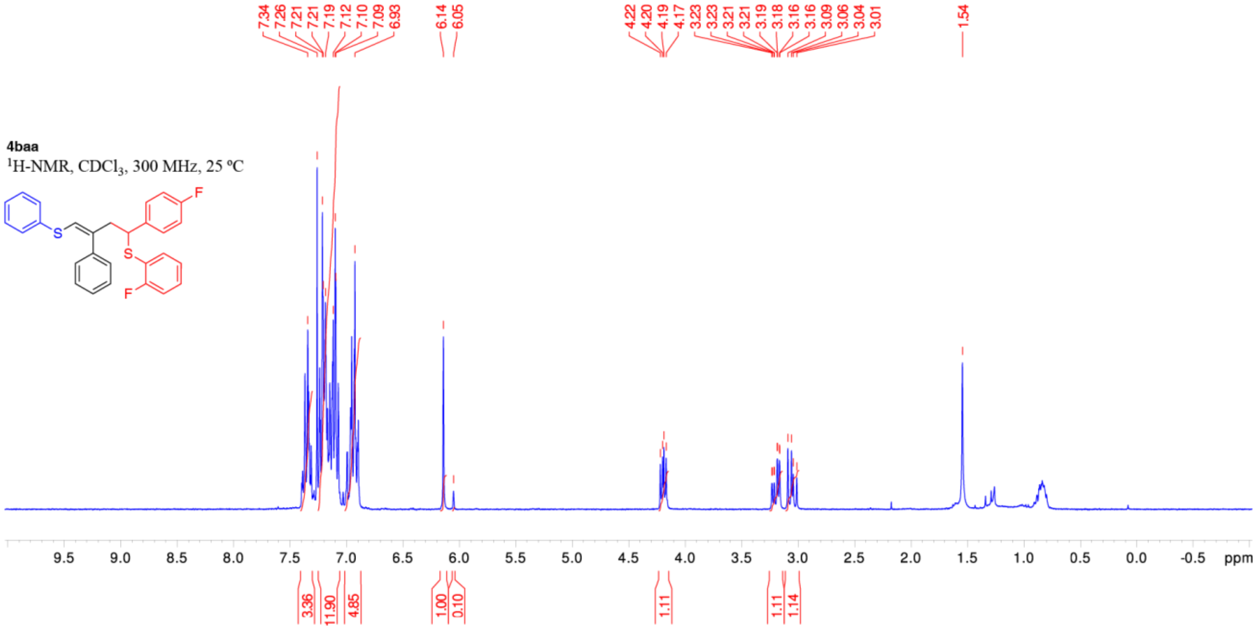
4baa
¹H-NMR, CDCl3, 300 MHz, 25 °C
7.34 7.26 7.21 7.21 7.19 7.12 7.10 7.09 6.93
6.14 6.05
4.22 4.20 4.19 4.17 3.23 3.23 3.21 3.21 3.19 3.18 3.16 3.16 3.09 3.06 3.04 3.01
1.54
3.36 11.90 4.85 1.00 0.10 1.11 1.11 1.14
ppm

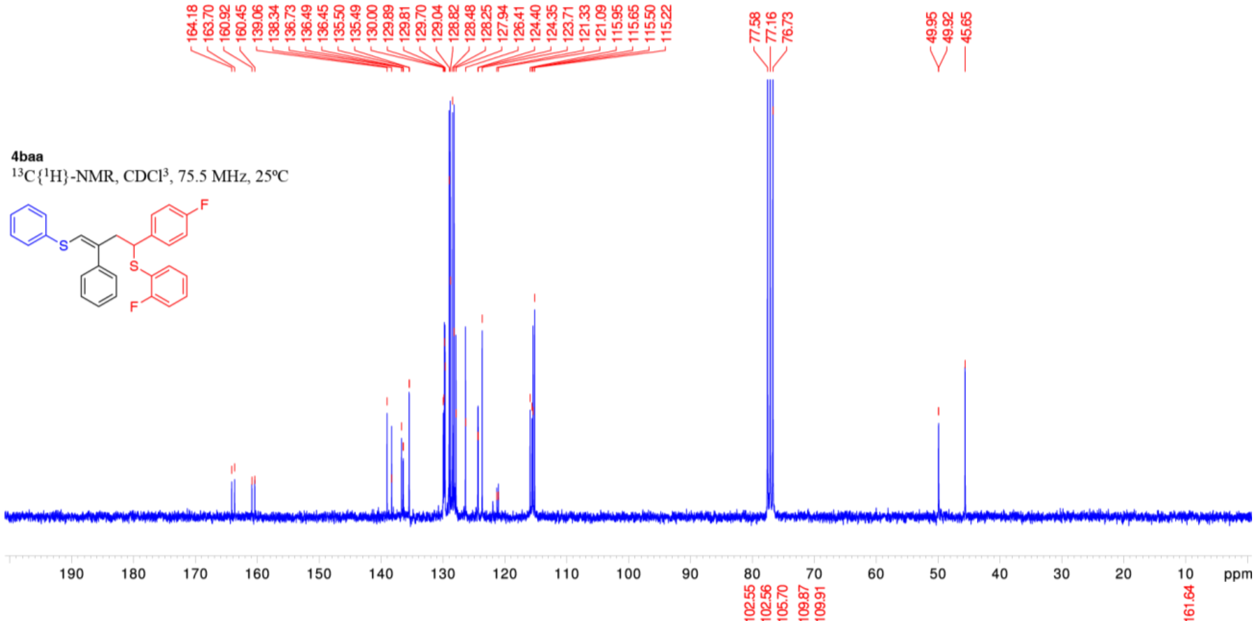
4baa
¹³C{¹H}-NMR, CDCl³, 75.5 MHz, 25ºC
164.18 163.70 160.92 160.45 139.06 138.34 136.73 136.49 136.45 135.50 135.49 130.00 129.89 129.81 129.70 129.04 128.82 128.48 128.25 127.94 126.41 124.40 124.35 123.71 121.33 121.09 115.95 115.65 115.50 115.22
77.58 77.16 76.73
49.95 49.92 45.65
ppm

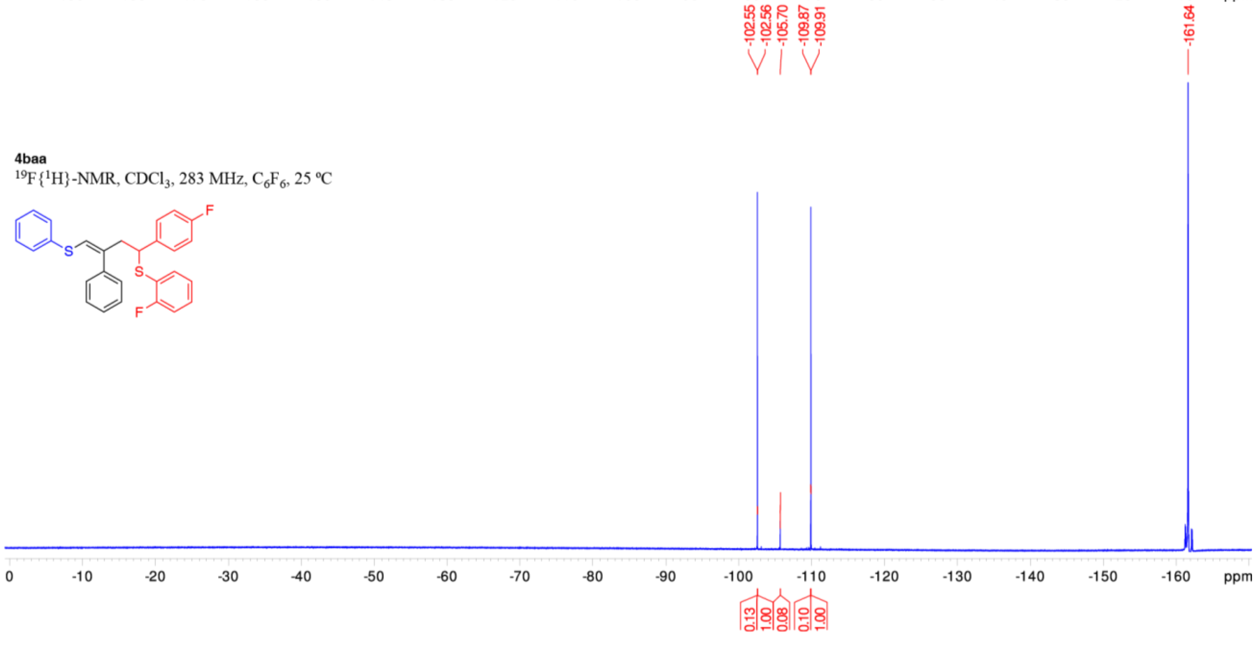
4baa
¹⁹F{¹H}-NMR, CDCl3, 283 MHz, C6F6, 25 °C
-102.55 -102.56 -105.70 -109.87 -109.91
-161.64
0.13 1.00 0.08 0.10 1.00
ppm

4bab

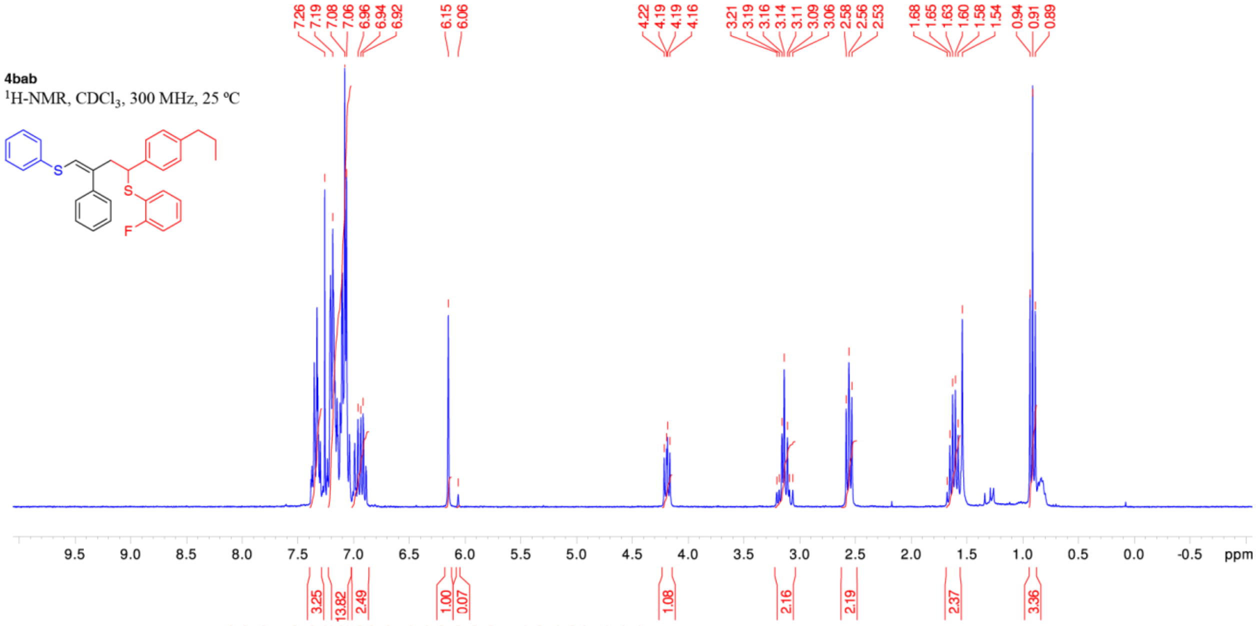
4bab
^{1}H-NMR, CDCl$_3$, 300 MHz, 25 °C
S
S
F
7.26 7.19 7.08 7.06 6.96 6.94 6.92
6.15 6.06
4.22 4.19 4.19 4.16
3.21 3.19 3.16 3.14 3.11 3.09 3.06 2.58 2.56 2.53
1.68 1.65 1.63 1.60 1.58 1.54
0.94 0.91 0.89
3.25 13.82 2.49 1.00 0.07 1.08 2.16 2.19 2.37 3.36
ppm

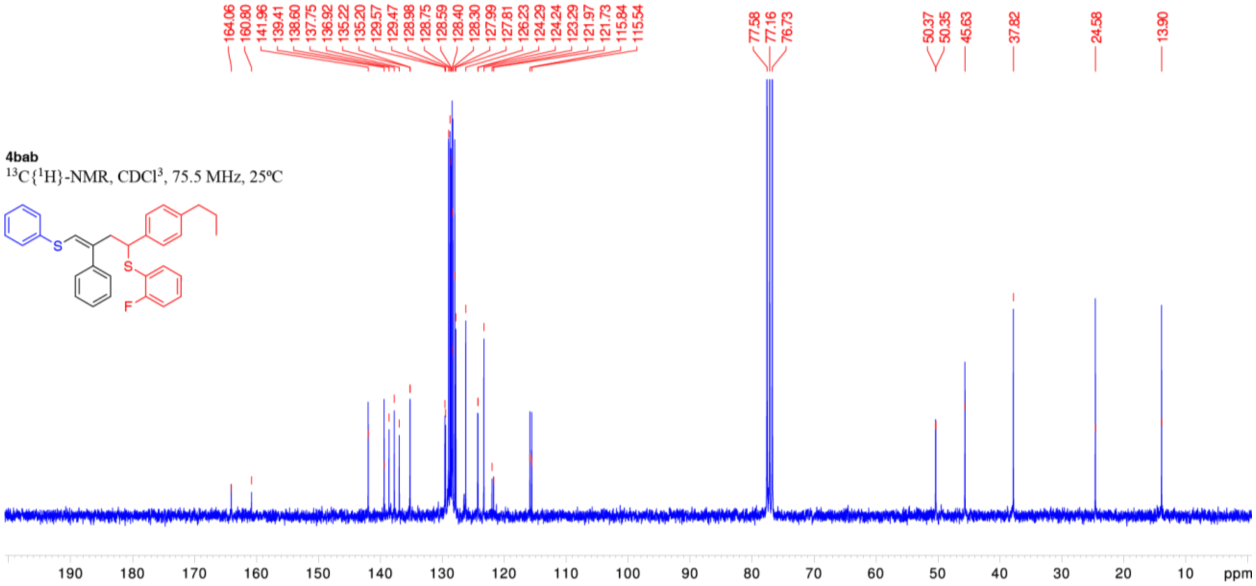
4bab
^{13}C{^{1}H}-NMR, CDCl^3, 75.5 MHz, 25ºC
S
S
F
164.06 160.80 141.96 139.41 138.60 137.75 136.92 135.22 135.20 129.57 129.47 128.98 128.75 128.59 128.40 128.30 127.99 127.81 126.23 124.29 124.24 123.29 121.97 121.73 115.84 115.54
77.58 77.16 76.73
50.37 50.35 45.63 37.82 24.58 13.90
ppm

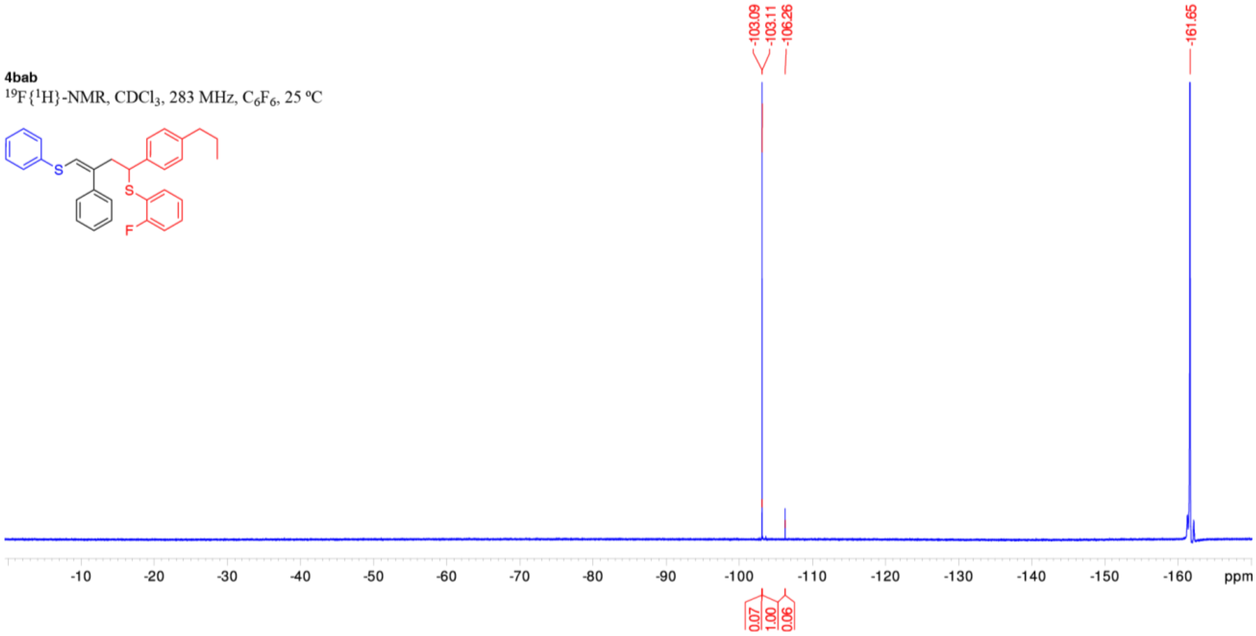
4bab
^{19}F{^{1}H}-NMR, CDCl$_3$, 283 MHz, C$_6$F$_6$, 25 °C
S
S
F
-103.09 -103.11 -106.26
-161.65
0.07 1.00 0.06
ppm

4bad

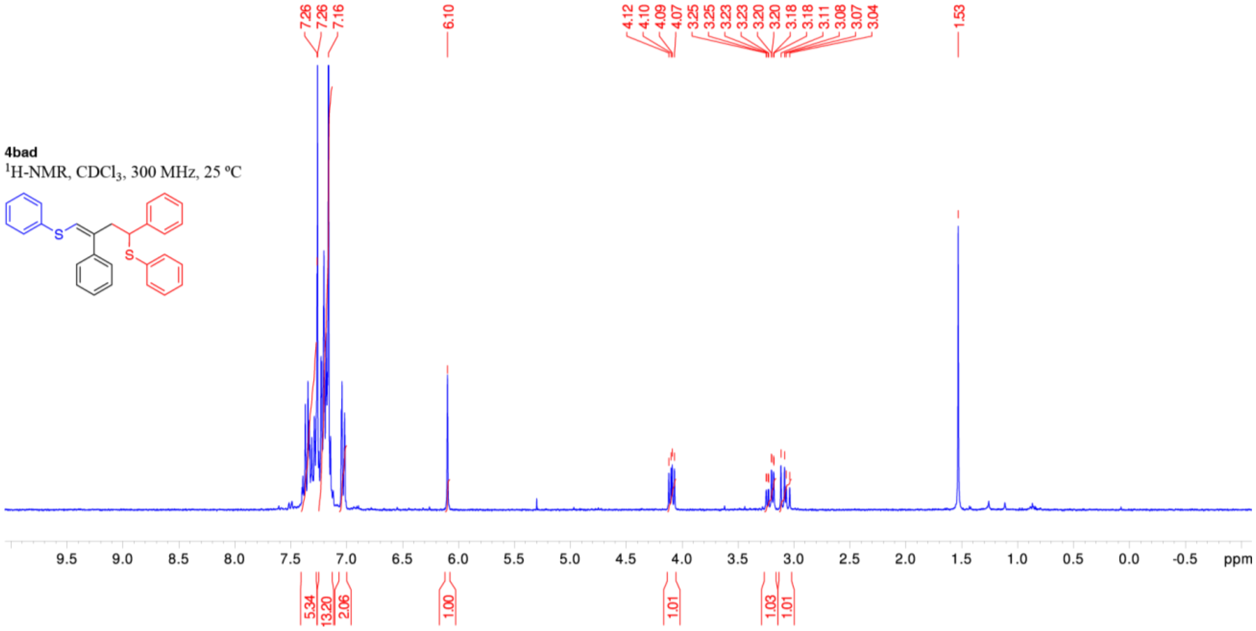

7.26
7.26
7.16
6.10
4.12
4.10
4.09
4.07
3.25
3.25
3.23
3.23
3.20
3.20
3.18
3.18
3.11
3.08
3.07
3.04
1.53
4bad
1H-NMR, CDCl3, 300 MHz, 25 ºC
S
S
9.5 9.0 8.5 8.0 7.5 7.0 6.5 6.0 5.5 5.0 4.5 4.0 3.5 3.0 2.5 2.0 1.5 1.0 0.5 0.0 -0.5 ppm
5.34
13.20
2.06
1.00
1.01
1.03
1.01

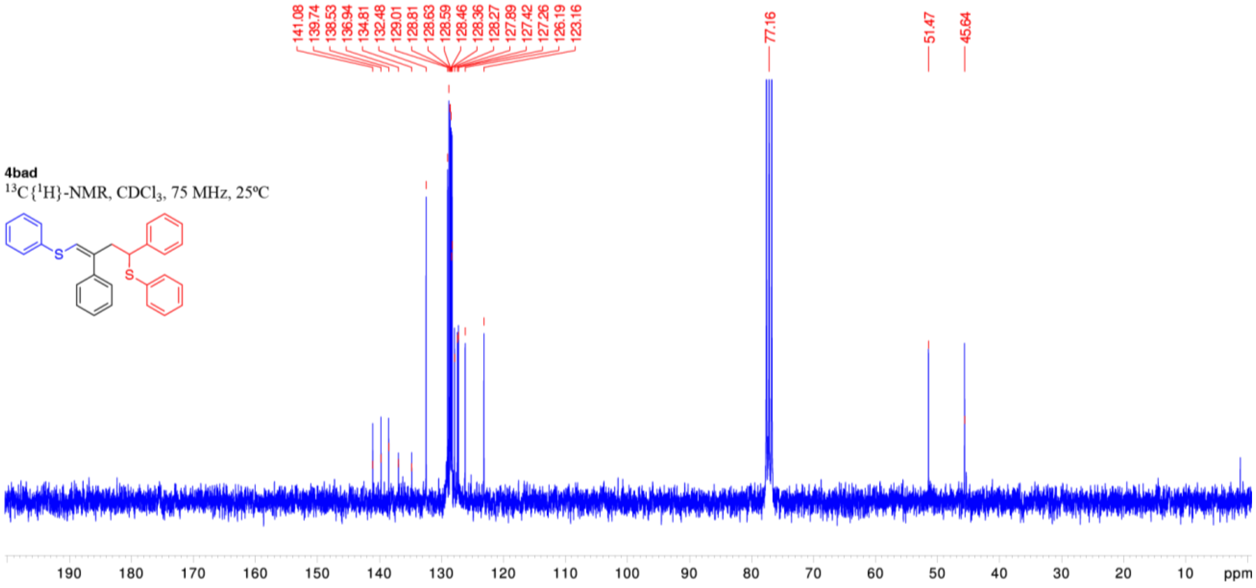

141.08
139.74
138.53
136.94
134.81
132.48
129.01
128.81
128.63
128.59
128.46
128.36
128.27
127.89
127.42
127.26
126.19
123.16
77.16
51.47
45.64
4bad
13C{1H}-NMR, CDCl3, 75 MHz, 25ºC
S
S
190 180 170 160 150 140 130 120 110 100 90 80 70 60 50 40 30 20 10 ppm

4bag

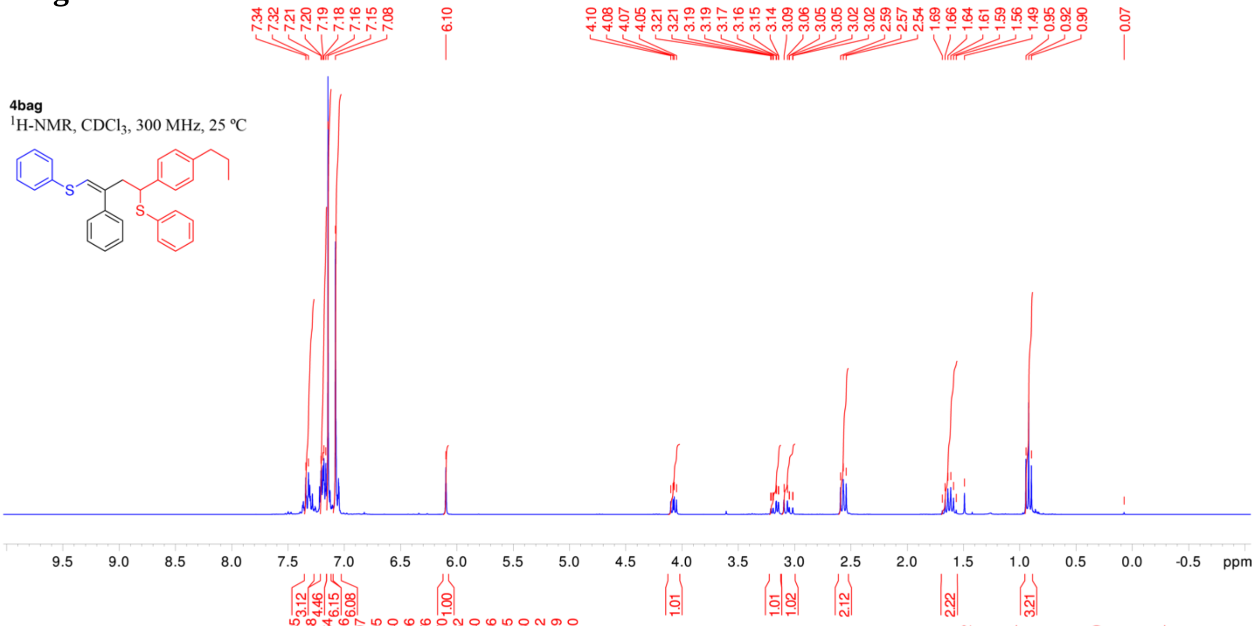

4bag
1H-NMR, CDCl3, 300 MHz, 25 ºC
S
S
ppm

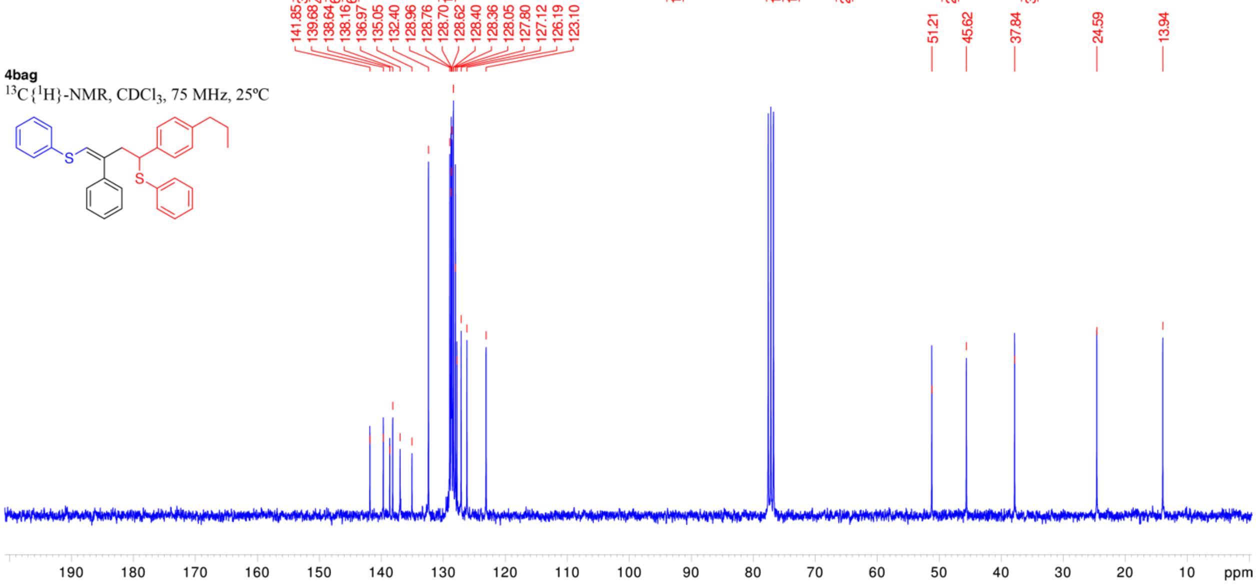

4bag
13C{1H}-NMR, CDCl3, 75 MHz, 25ºC
S
S
ppm

4aae

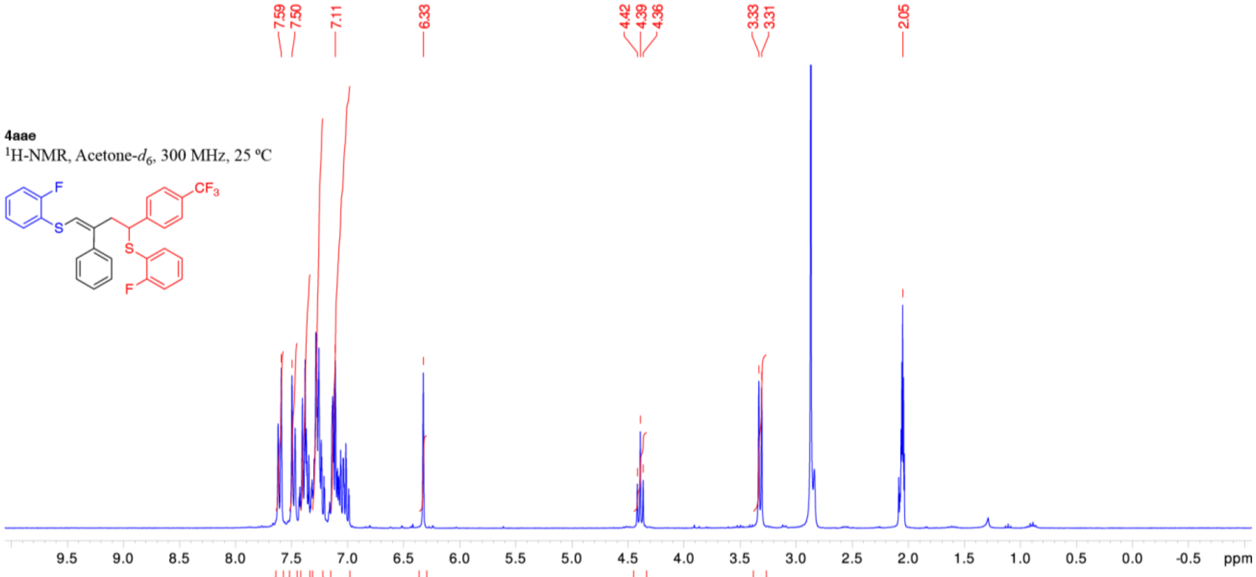
4aae
¹H-NMR, Acetone-d6, 300 MHz, 25 ºC

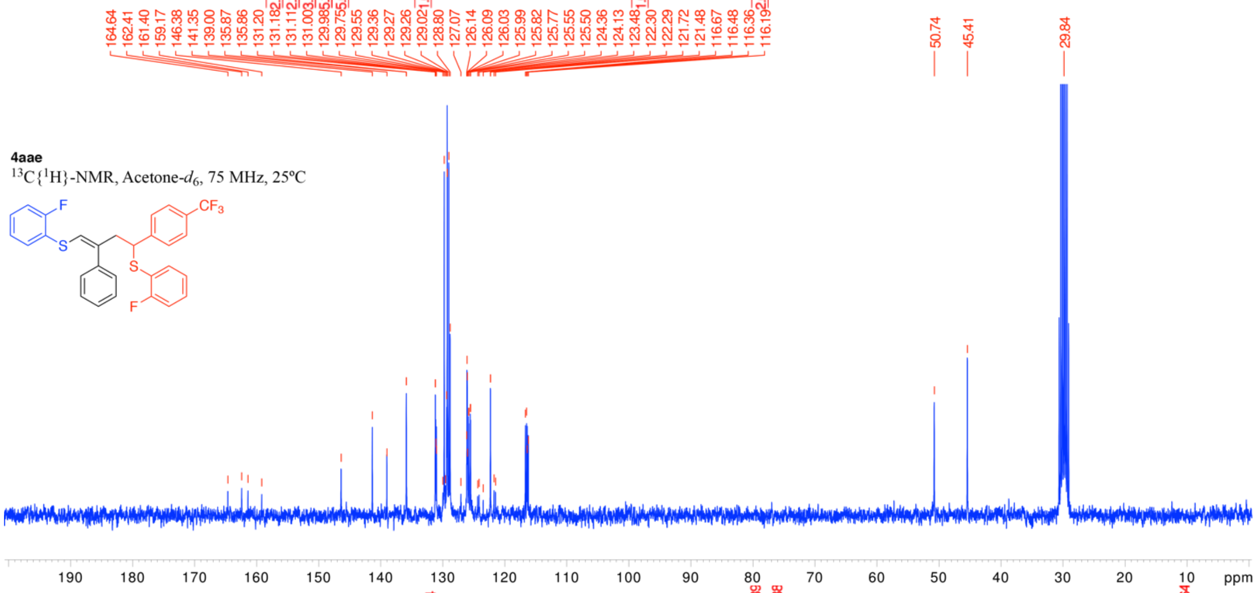
4aae
¹³C{¹H}-NMR, Acetone-d6, 75 MHz, 25ºC

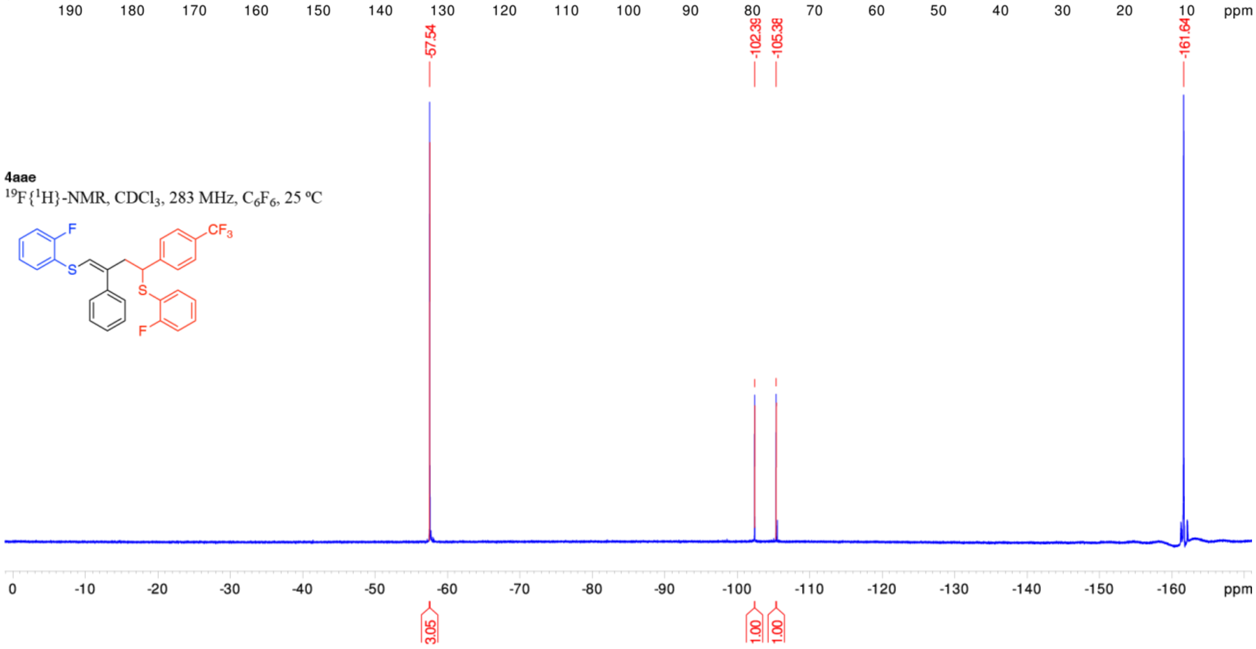
4aae
¹⁹F{¹H}-NMR, CDCl3, 283 MHz, C6F6, 25 ºC

4aaf

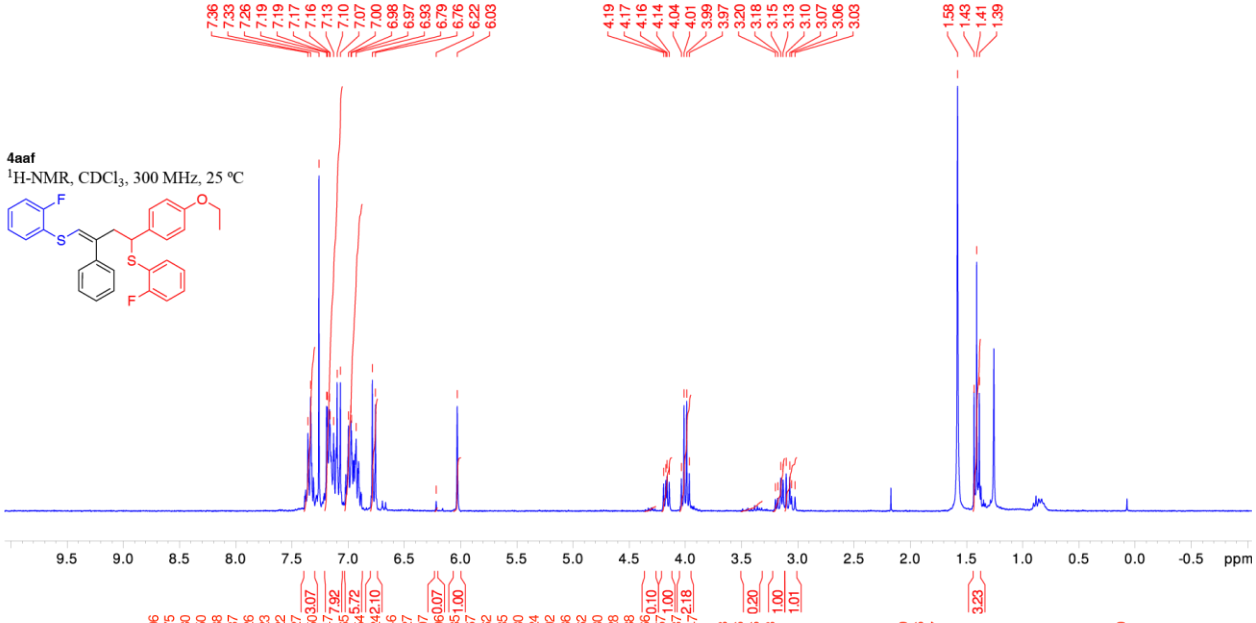
4aaf
^{1}H-NMR, $CDCl_3$, 300 MHz, 25 ºC
ppm

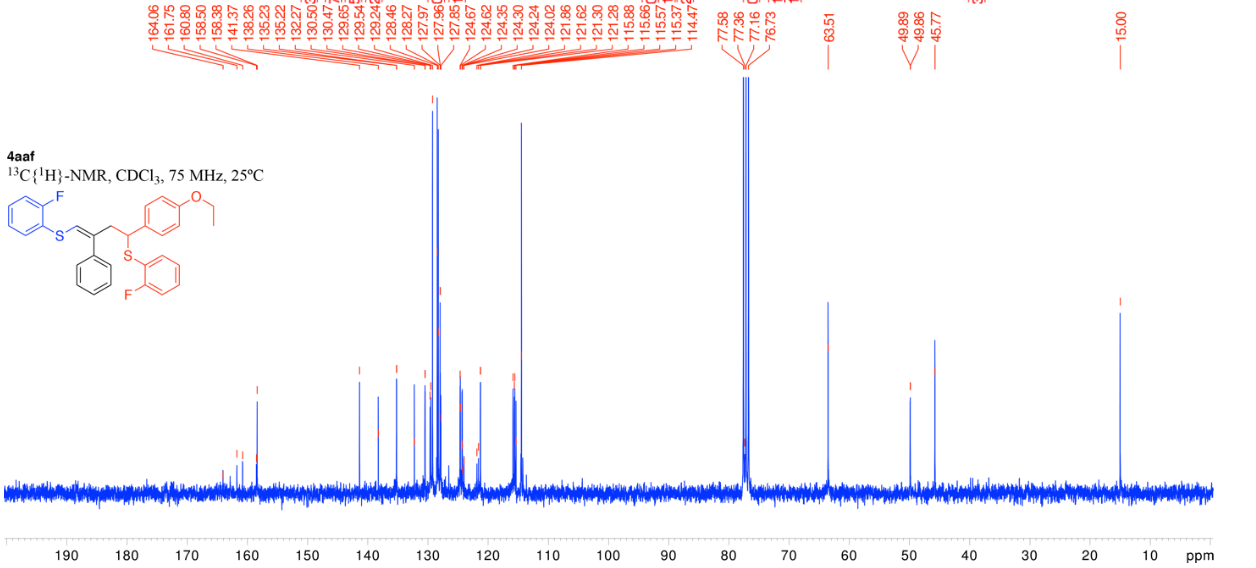
4aaf
$^{13}C\{^{1}H\}$-NMR, $CDCl_3$, 75 MHz, 25ºC
ppm

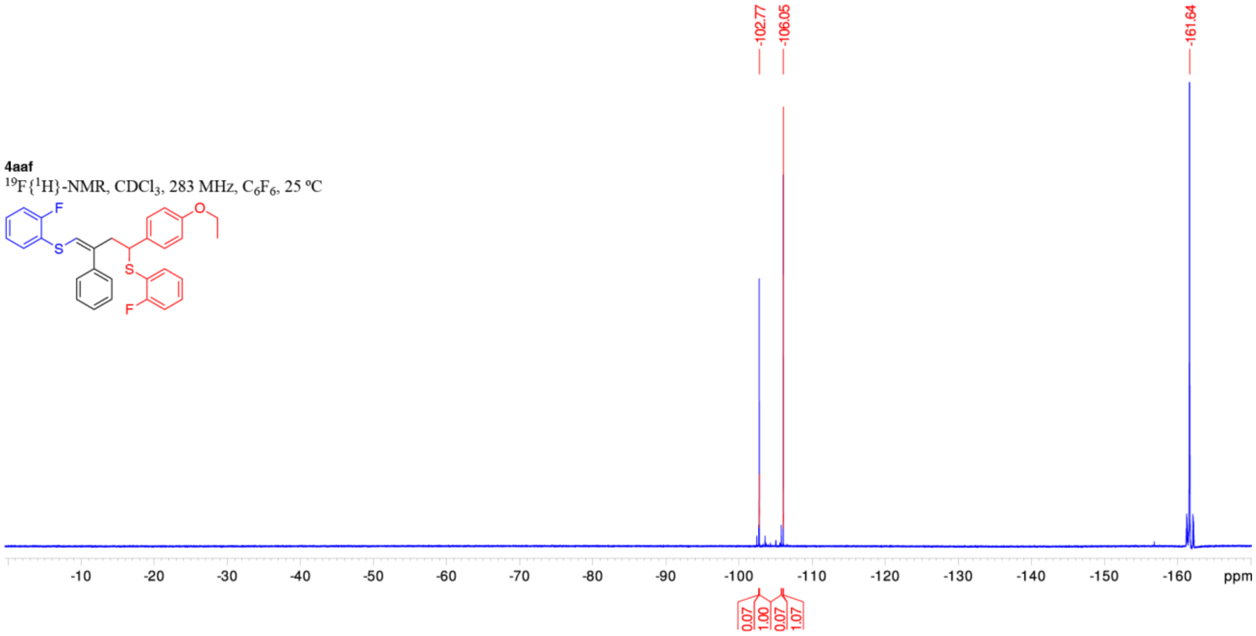
4aaf
$^{19}F\{^{1}H\}$-NMR, $CDCl_3$, 283 MHz, C_6F_6, 25 ºC
-102.77
-106.05
-161.64
ppm

4bhd

4bhd
^{1}H-NMR, DMSO-d_6, 400 MHz, 100 ºC

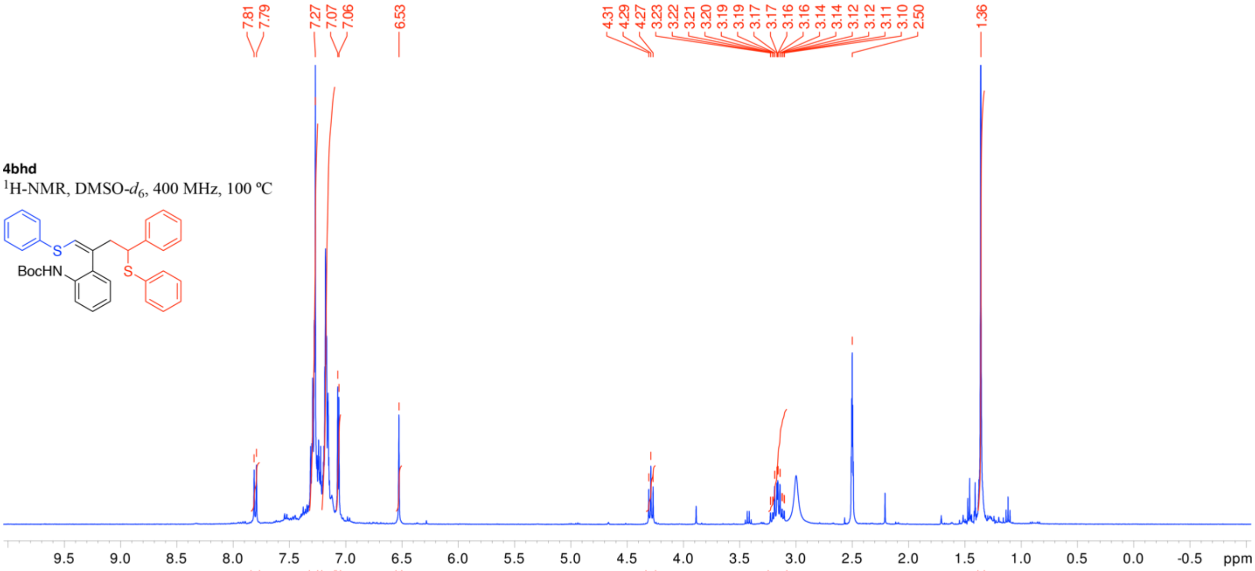

4bhd
^{13}C{^{1}H}-NMR, DMSO-d_6, 100 MHz, 100 ºC

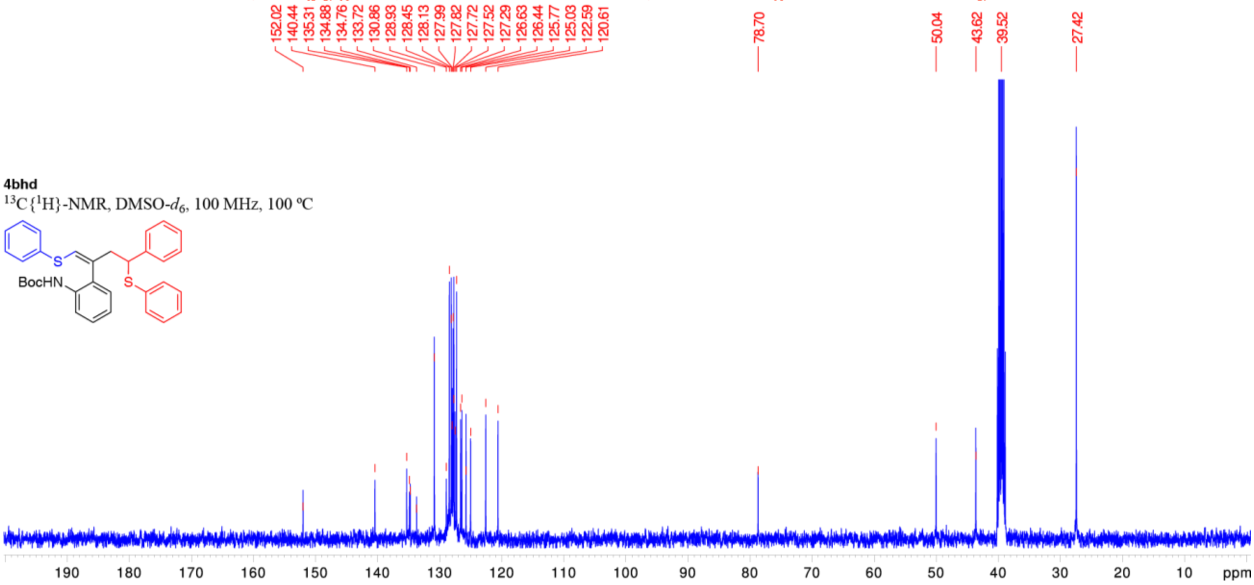

D-4bdd

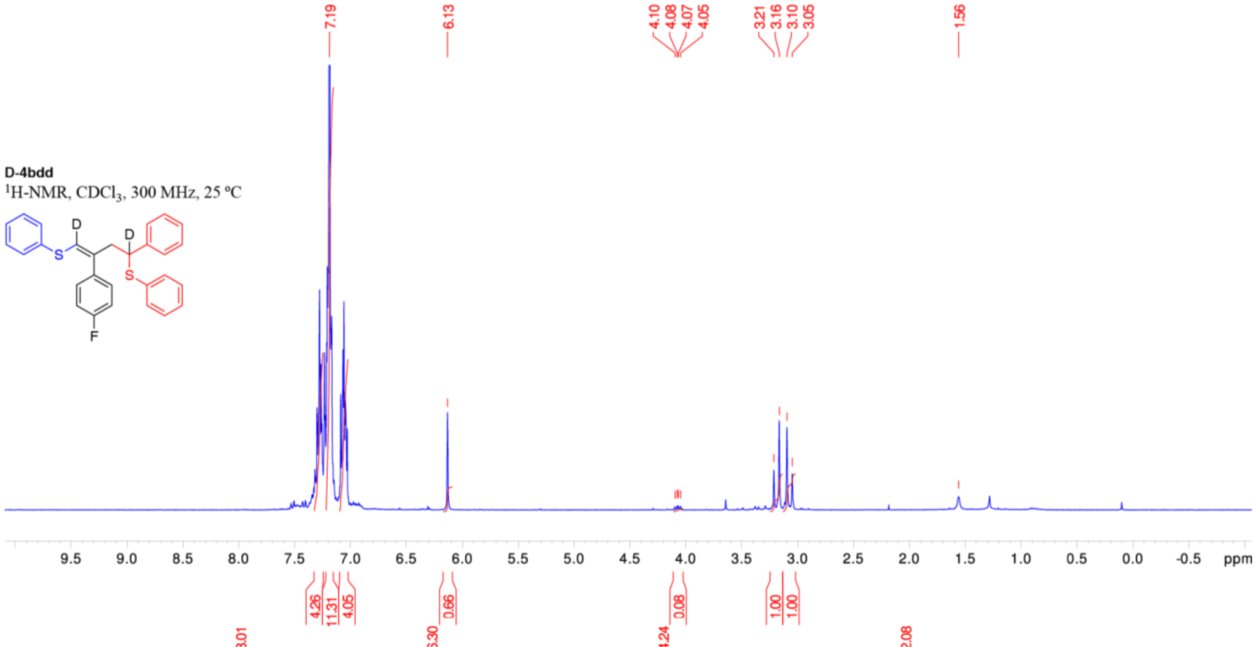

D-4bdd
¹H-NMR, CDCl3, 300 MHz, 25 ºC
7.19
6.13
4.10
4.08
4.07
4.05
3.21
3.16
3.10
3.05
1.56
4.26
11.31
4.05
0.66
0.08
1.00
1.00
9.5 9.0 8.5 8.0 7.5 7.0 6.5 6.0 5.5 5.0 4.5 4.0 3.5 3.0 2.5 2.0 1.5 1.0 0.5 0.0 -0.5 ppm

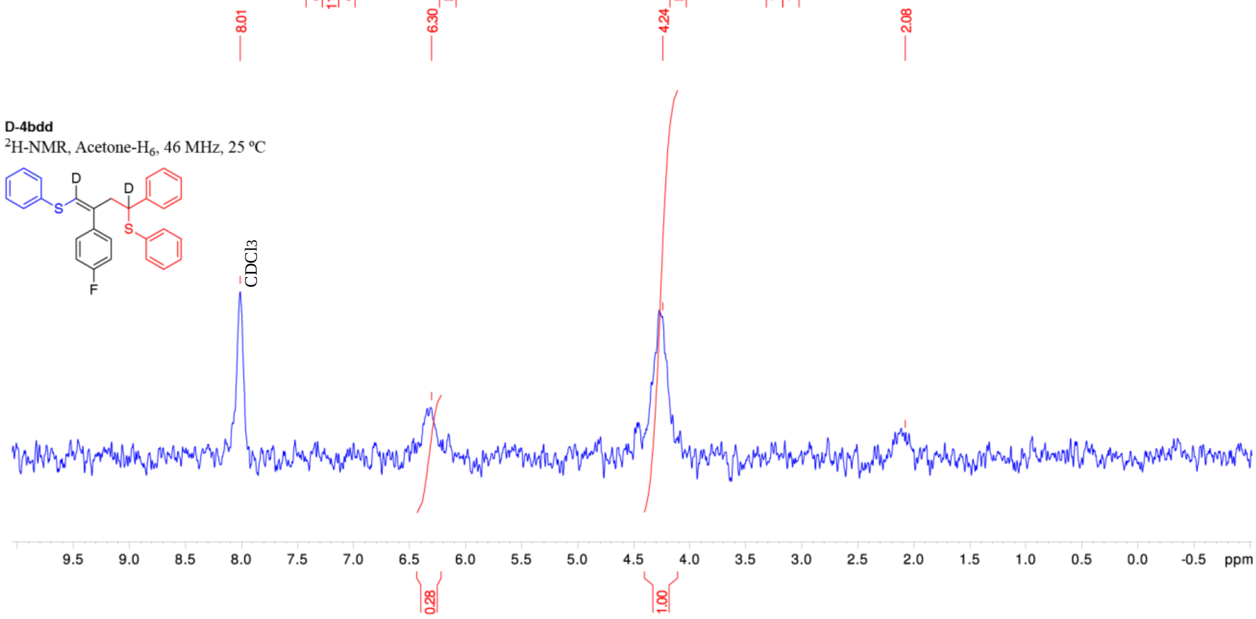

D-4bdd
²H-NMR, Acetone-H6, 46 MHz, 25 ºC
8.01
6.30
4.24
2.08
CDCl3
0.28
1.00
9.5 9.0 8.5 8.0 7.5 7.0 6.5 6.0 5.5 5.0 4.5 4.0 3.5 3.0 2.5 2.0 1.5 1.0 0.5 0.0 -0.5 ppm

D-4bdd

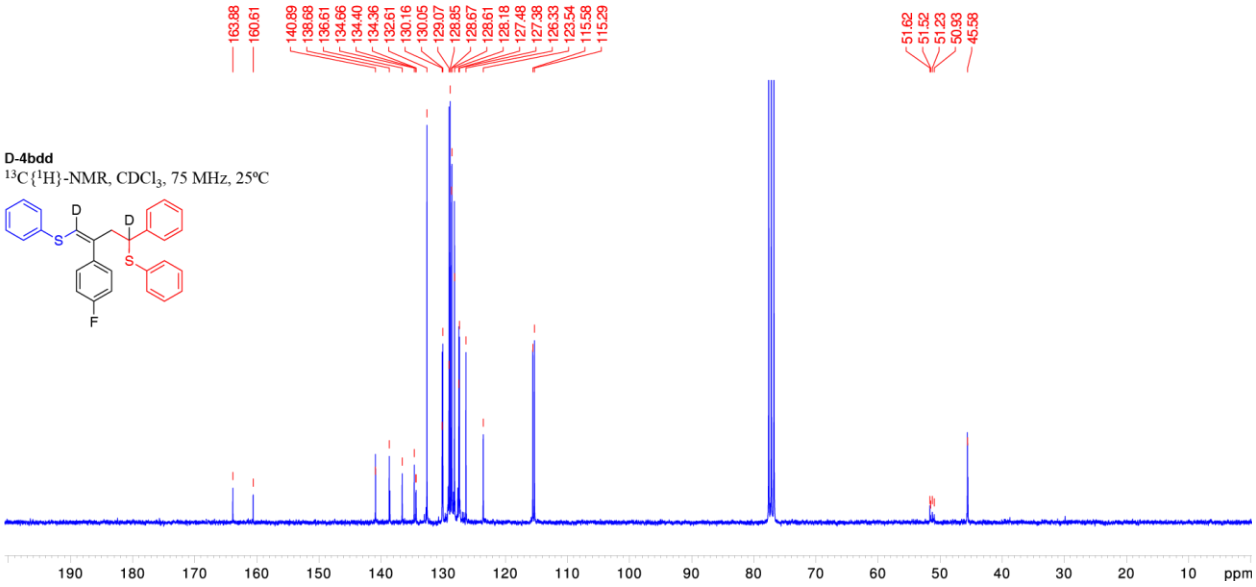
D-4bdd
$^{13}C\{^1H\}$-NMR, $CDCl_3$, 75 MHz, 25ºC
163.88
160.61
140.89
138.68
136.61
134.66
134.40
134.36
132.61
130.16
130.05
129.07
128.85
128.67
128.61
128.18
127.48
127.38
126.33
123.54
115.58
115.29
51.62
51.52
51.23
50.93
45.58
ppm

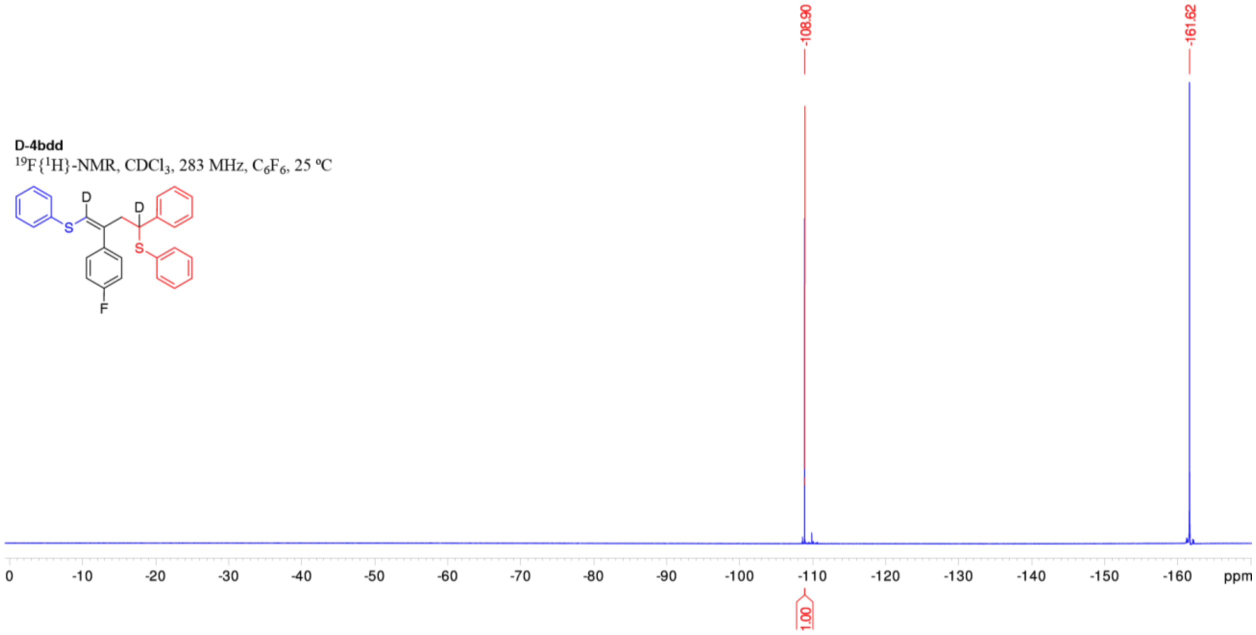
D-4bdd
$^{19}F\{^1H\}$-NMR, $CDCl_3$, 283 MHz, C_6F_6, 25 ºC
-108.90
-161.62
1.00
ppm

4bid

4bid
^{1}H-NMR, $CDCl_3$, 300 MHz, 25 ºC

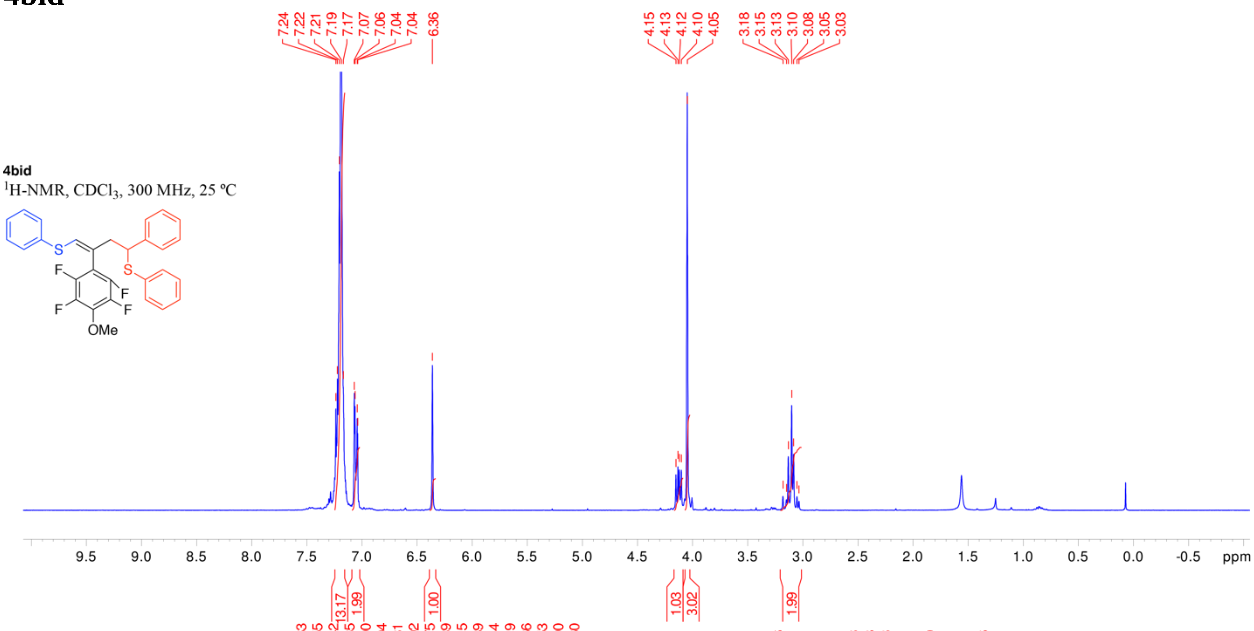

4bid
^{13}C{^{1}H}-NMR, $CDCl_3$, 75 MHz, 25ºC

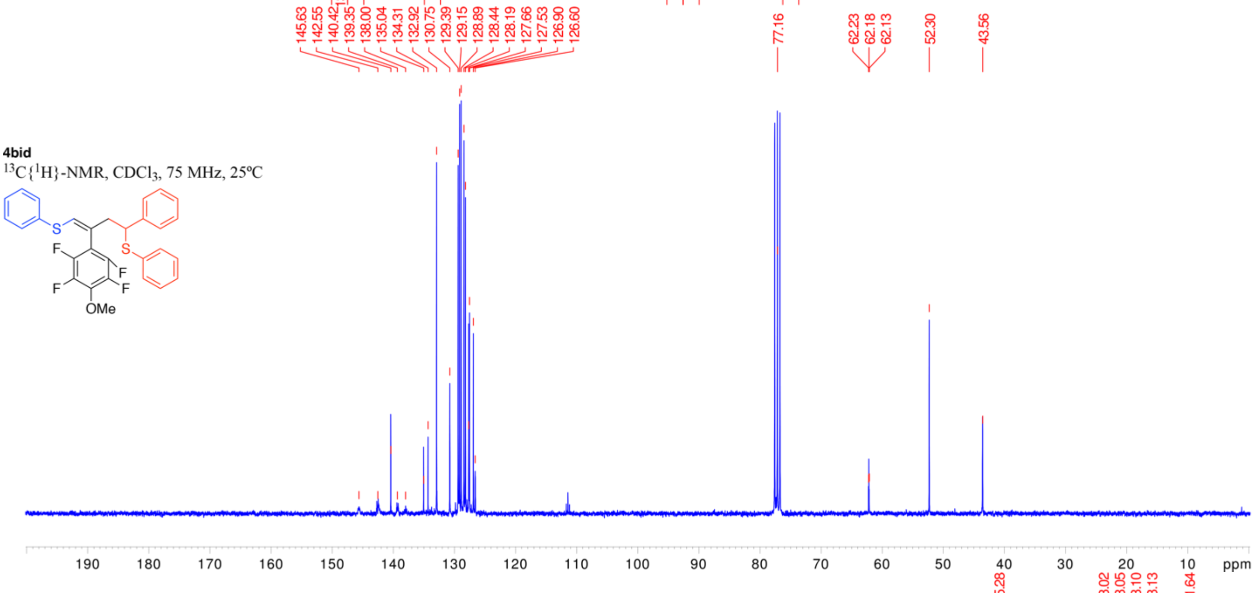

4bid
^{19}F{^{1}H}-NMR, $CDCl_3$, 283 MHz, C_6F_6, 25 ºC

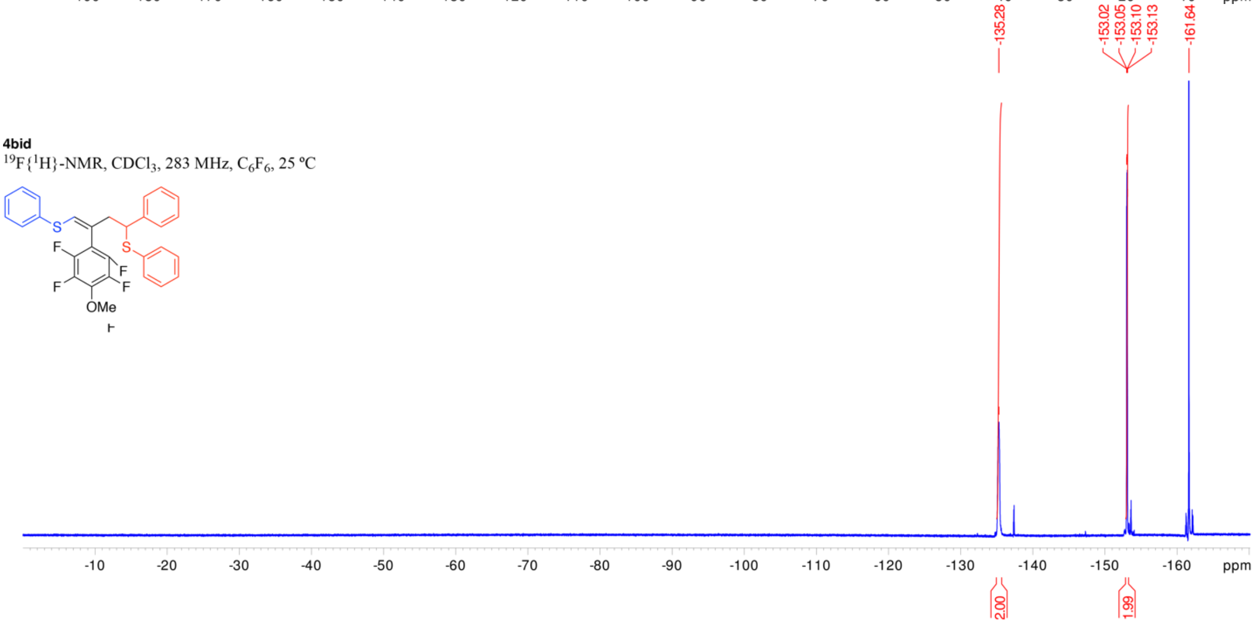

4bjd

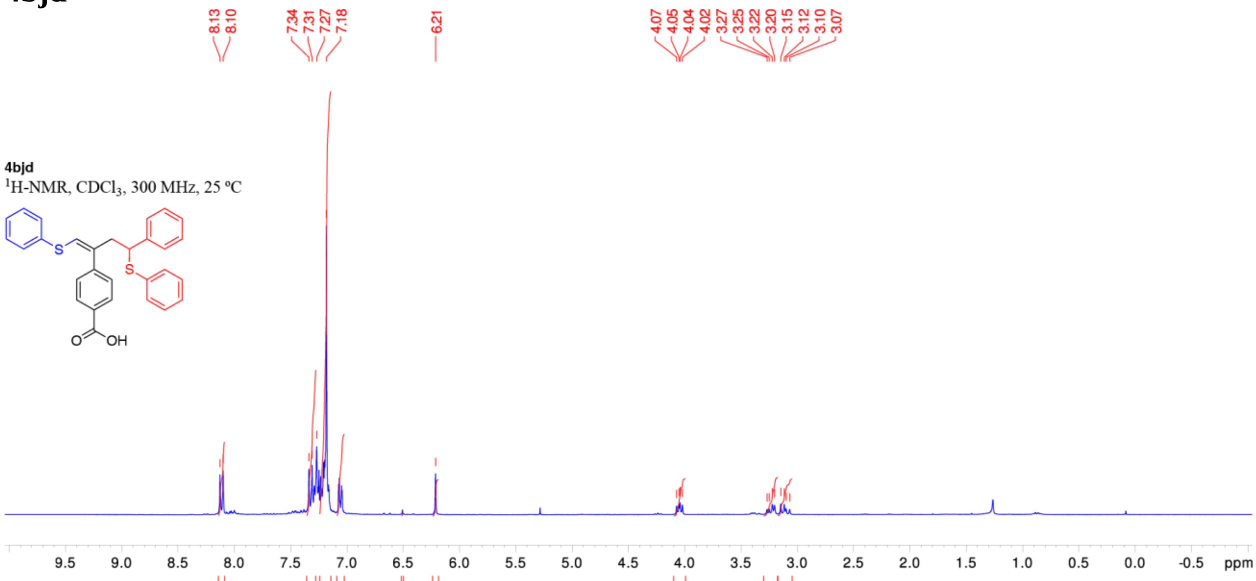
4bjd
^{1}H-NMR, $CDCl_3$, 300 MHz, 25 ºC
8.13
8.10
7.34
7.31
7.27
7.18
6.21
4.07
4.05
4.04
4.02
3.27
3.25
3.22
3.20
3.15
3.12
3.10
3.07
2.04
4.08
11.69
2.25
0.08
1.00
1.02
1.06
1.01
ppm

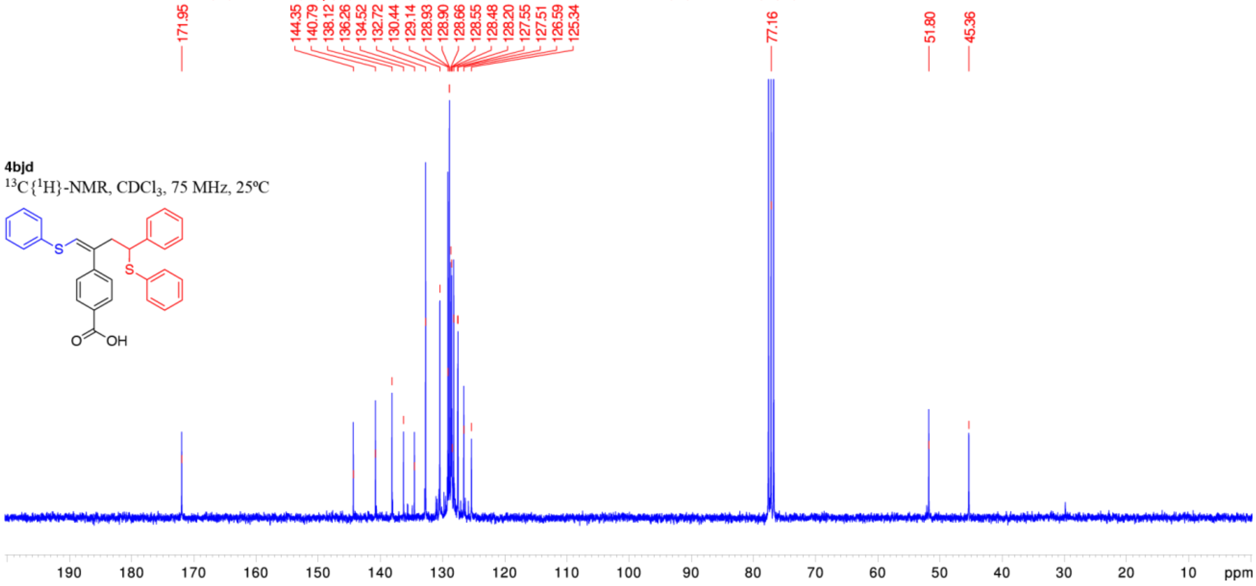
4bjd
$^{13}C\{^1H\}$-NMR, $CDCl_3$, 75 MHz, 25ºC
171.95
144.35
140.79
138.12
136.26
134.52
132.72
130.44
129.14
128.93
128.90
128.66
128.55
128.48
128.20
127.55
127.51
126.59
125.34
77.16
51.80
45.36
ppm

4bkd

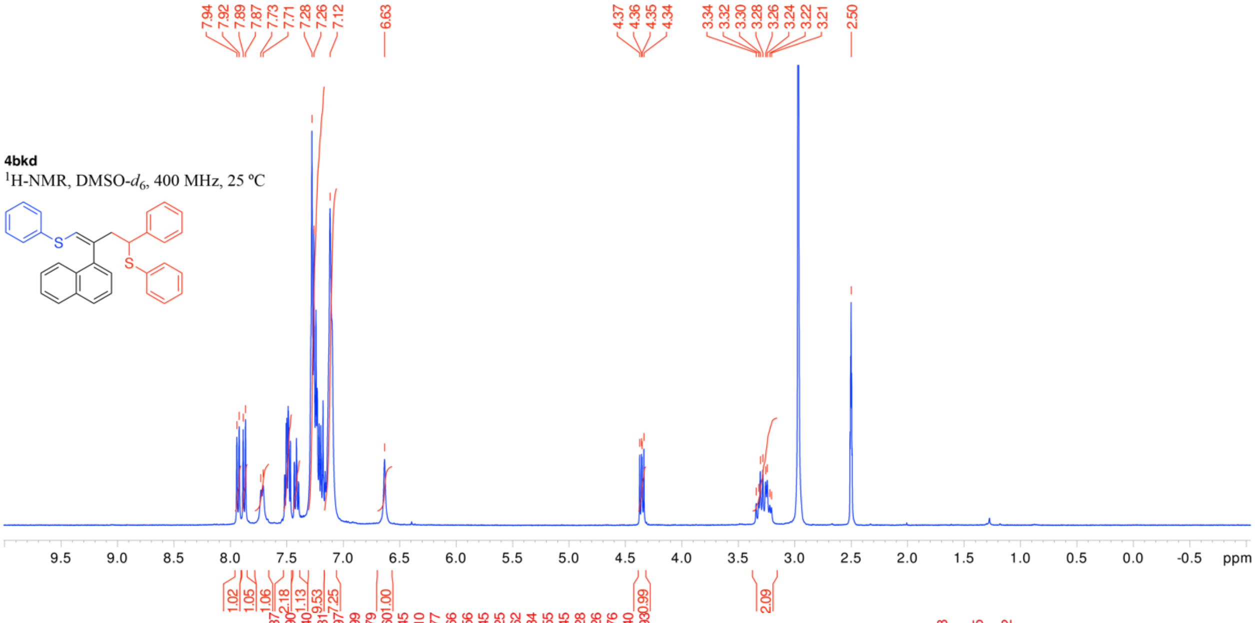

7.94 7.92 7.89 7.87 7.73 7.71 7.28 7.26 7.12
6.63
4.37 4.36 4.35 4.34
3.34 3.32 3.30 3.28 3.26 3.24 3.22 3.21
2.50
4bkd
^{1}H-NMR, DMSO-d_6, 400 MHz, 25 ºC
S
S
1.02 1.05 1.06 2.18 1.13 9.53 7.25 1.00
0.99
2.09
9.5 9.0 8.5 8.0 7.5 7.0 6.5 6.0 5.5 5.0 4.5 4.0 3.5 3.0 2.5 2.0 1.5 1.0 0.5 0.0 -0.5 ppm

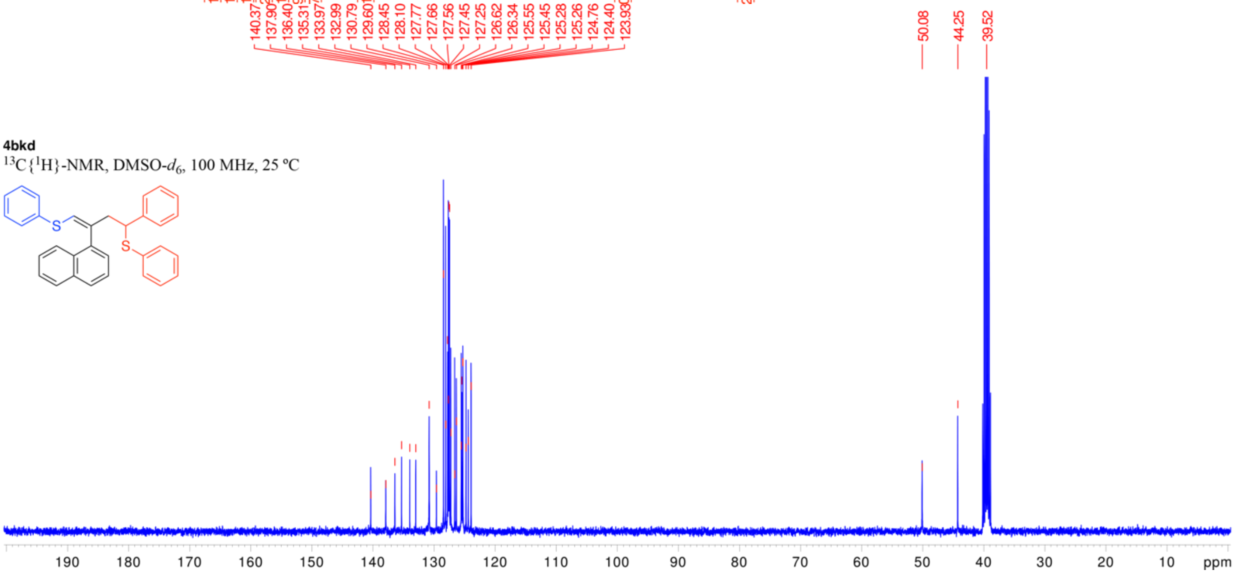

140.37 137.90 136.40 135.31 133.97 132.99 130.79 129.60 128.45 128.10 127.77 127.66 127.56 127.45 127.25 126.62 126.34 125.55 125.45 125.28 125.26 124.76 124.40 123.93
50.08
44.25
39.52
4bkd
^{13}C{^{1}H}-NMR, DMSO-d_6, 100 MHz, 25 ºC
S
S
190 180 170 160 150 140 130 120 110 100 90 80 70 60 50 40 30 20 10 ppm

6bbd

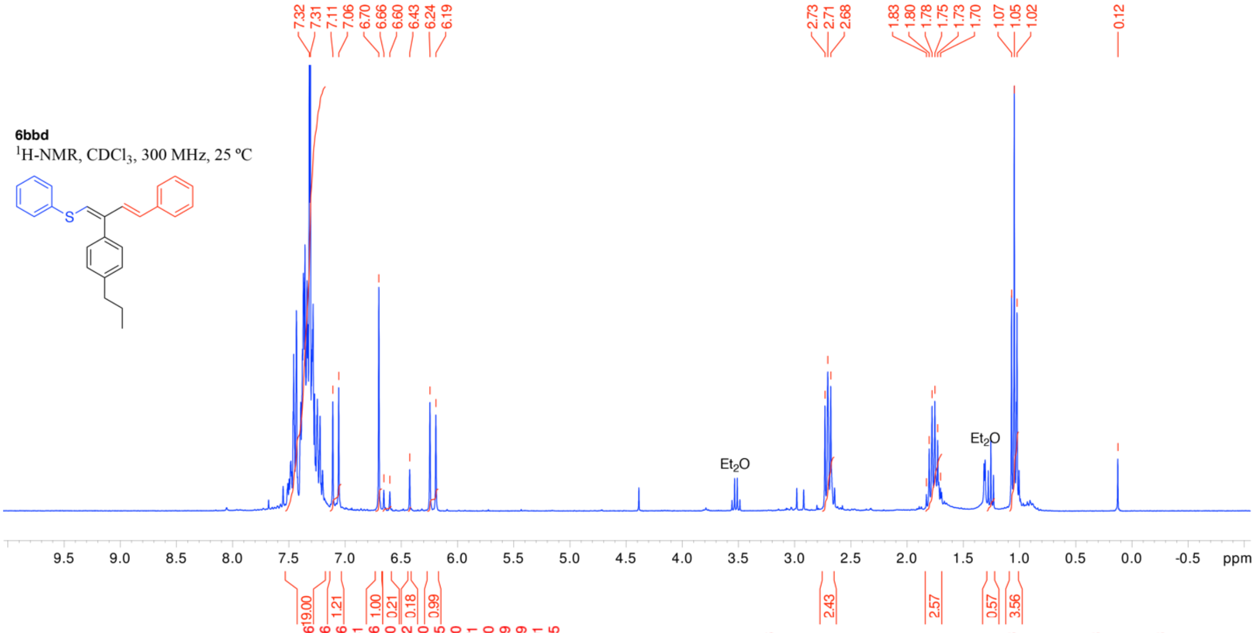
6bbd
1H-NMR, CDCl3, 300 MHz, 25 ºC
S
Et2O
Et2O
ppm

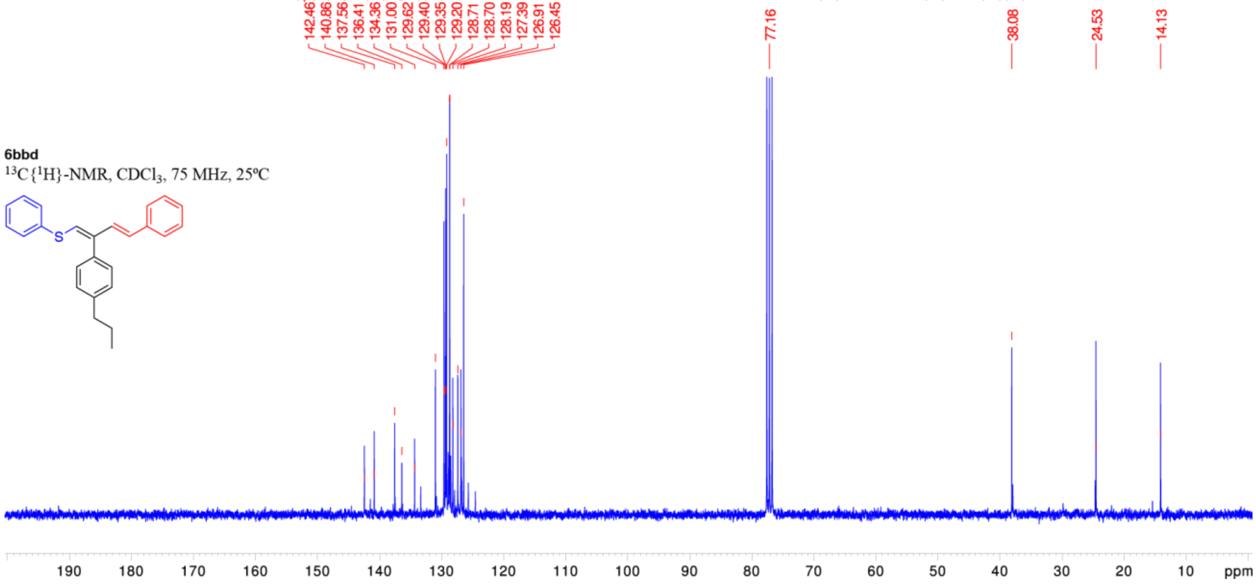
6bbd
13C{1H}-NMR, CDCl3, 75 MHz, 25ºC
S
ppm

6bad

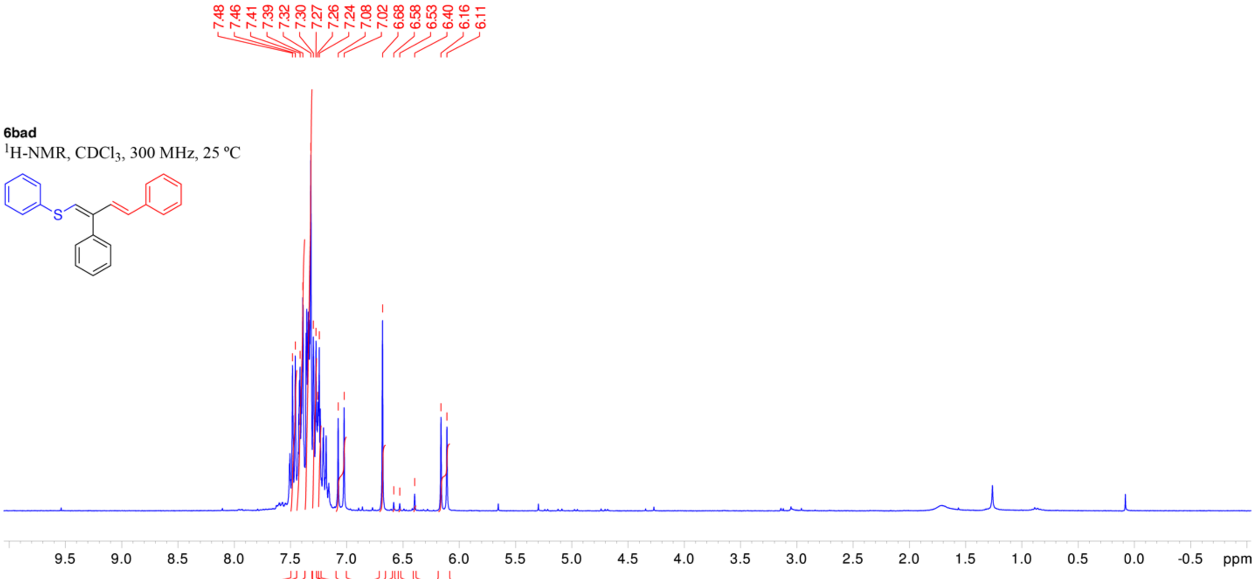
7.48
7.46
7.41
7.39
7.32
7.30
7.27
7.26
7.24
7.08
7.02
6.68
6.58
6.53
6.40
6.16
6.11
6bad
^{1}H-NMR, CDCl$_3$, 300 MHz, 25 ºC
S
2.12
4.11
6.37
2.35
1.30
1.12
1.00
0.05
0.04
0.09
1.02
ppm

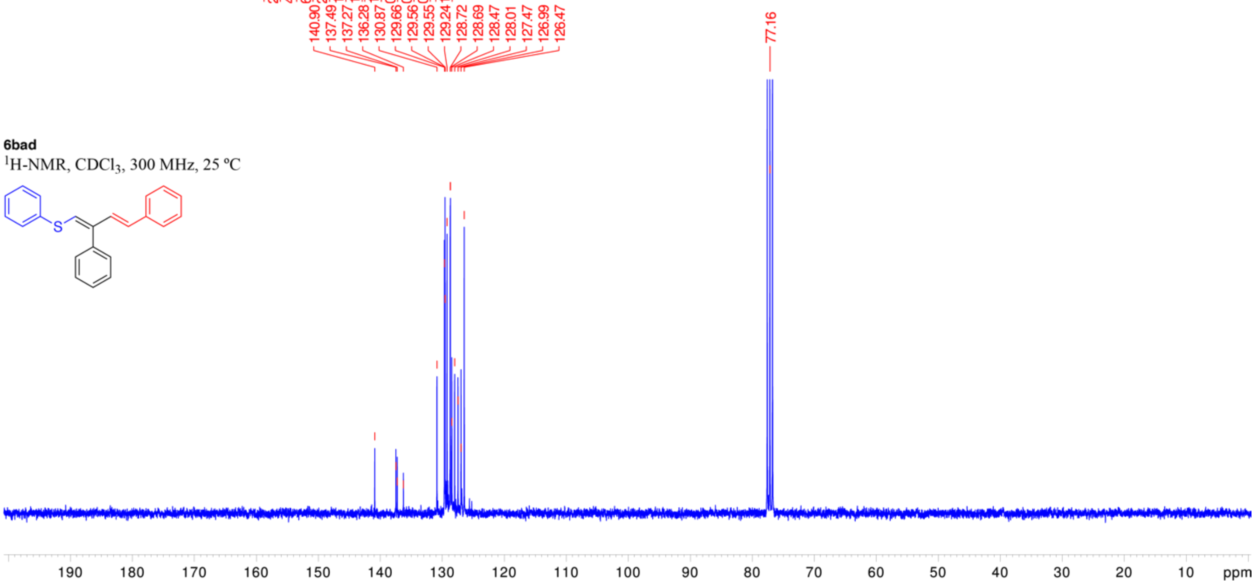
140.90
137.49
137.27
136.28
130.87
129.66
129.56
129.55
129.24
128.72
128.69
128.47
128.01
127.47
126.99
126.47
77.16
6bad
^{1}H-NMR, CDCl$_3$, 300 MHz, 25 ºC
S
ppm

6bag

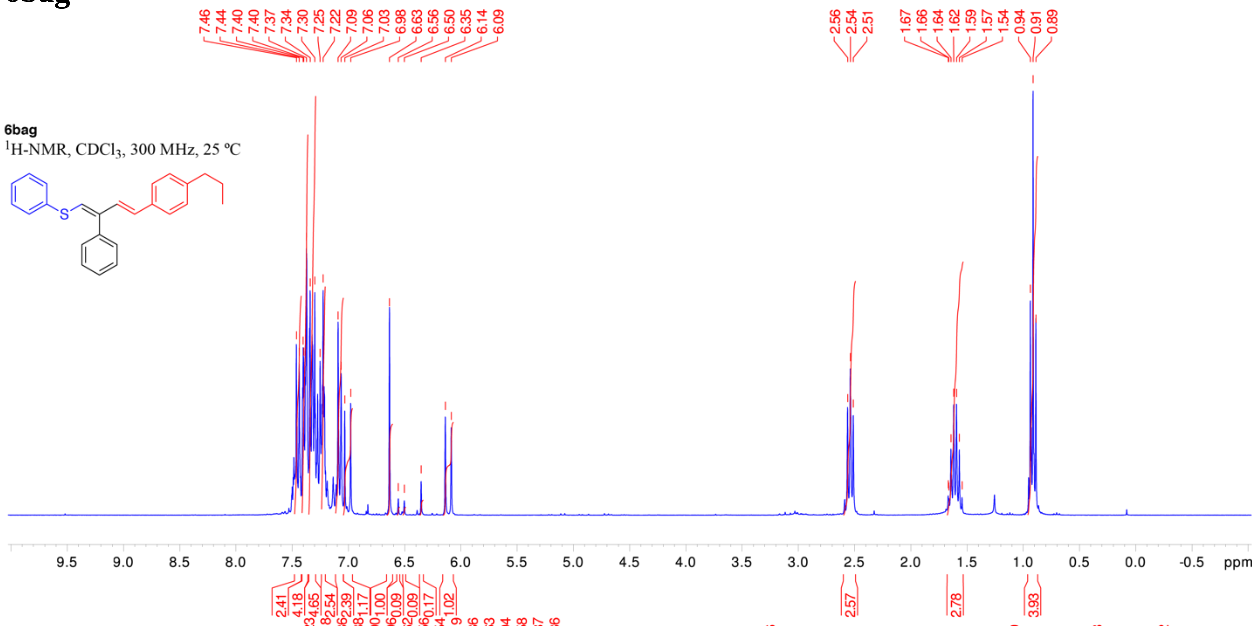

7.46 7.44 7.40 7.40 7.37 7.34 7.30 7.25 7.22 7.09 7.06 7.03 6.98 6.63 6.56 6.50 6.35 6.14 6.09
2.56 2.54 2.51
1.67 1.66 1.64 1.62 1.59 1.57 1.54 0.94 0.91 0.89
6bag
1H-NMR, CDCl3, 300 MHz, 25 ºC
S
9.5 9.0 8.5 8.0 7.5 7.0 6.5 6.0 5.5 5.0 4.5 4.0 3.5 3.0 2.5 2.0 1.5 1.0 0.5 0.0 -0.5 ppm
2.41 4.18 4.65 2.54 2.39 1.17 1.00 0.09 0.09 0.17 1.02
2.57
2.78
3.93

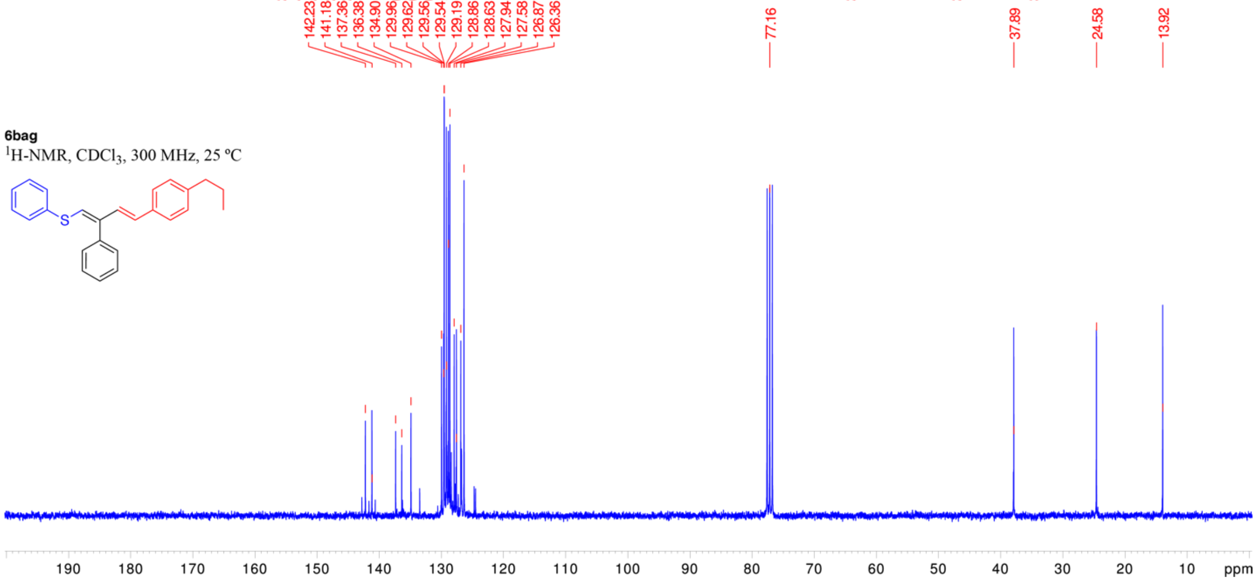

142.23 141.18 137.36 136.38 134.90 129.96 129.62 129.56 129.54 129.19 128.86 128.63 127.94 127.58 126.87 126.36
77.16
37.89
24.58
13.92
6bag
1H-NMR, CDCl3, 300 MHz, 25 ºC
S
190 180 170 160 150 140 130 120 110 100 90 80 70 60 50 40 30 20 10 ppm

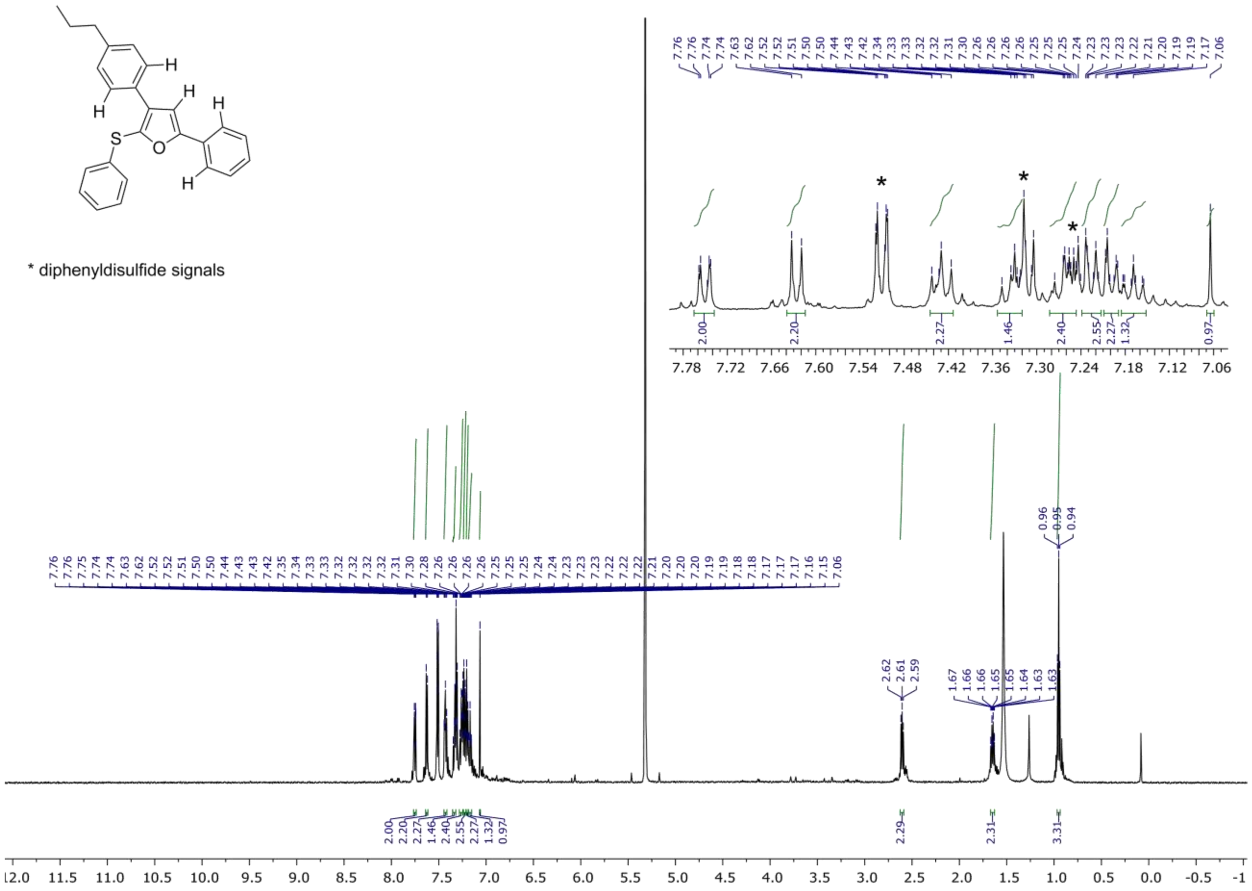

Figure S23. ^{1}H NMR spectrum for 5-phenyl-2-(phenylthio)-3-(4-propylphenyl)furan, CD_2Cl_2, 151 MHz. Signals from diphenyldisulfide are marked with asterisk.

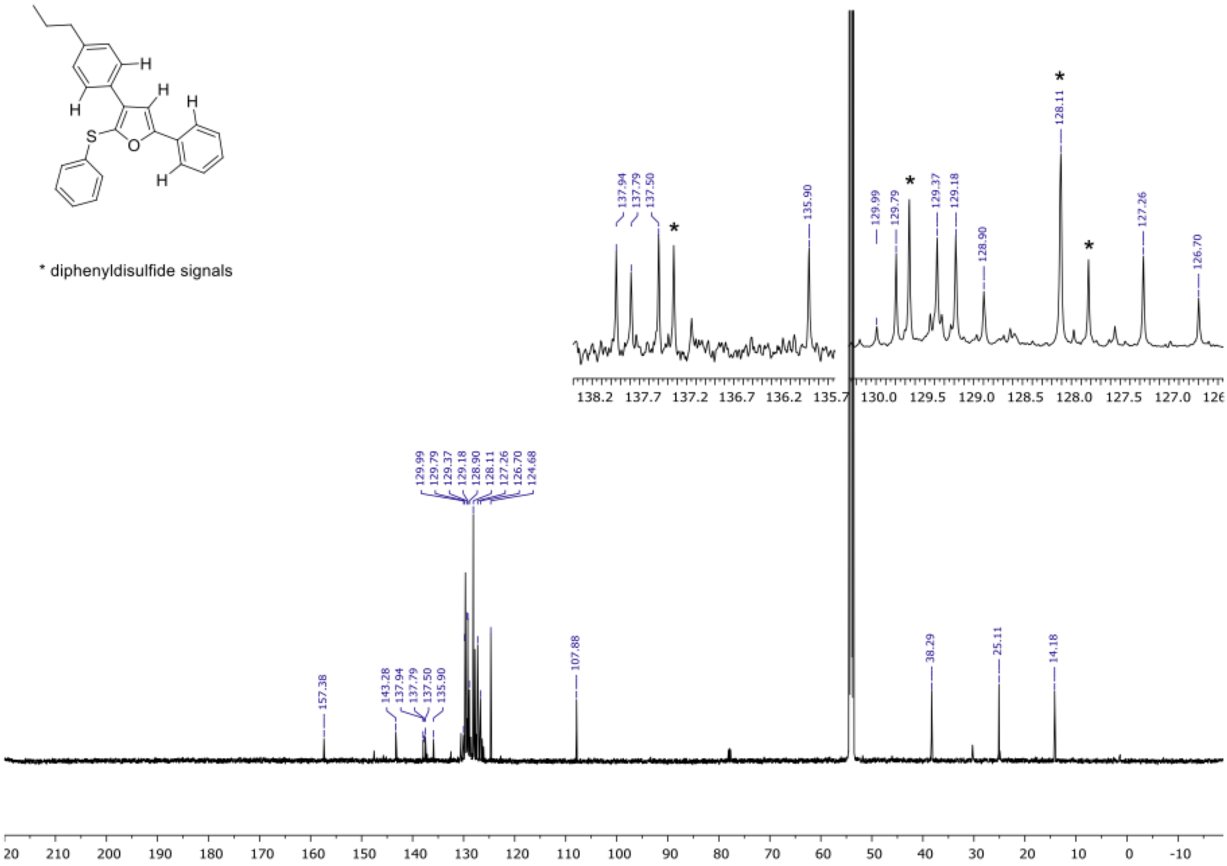

Figure S24. $^{13}C\{^{1}H\}$ NMR spectrum for 5-phenyl-2-(phenylthio)-3-(4-propylphenyl)furan, CD_2Cl_2, 151 MHz. Signals from diphenyldisulfide are marked with asterisk.

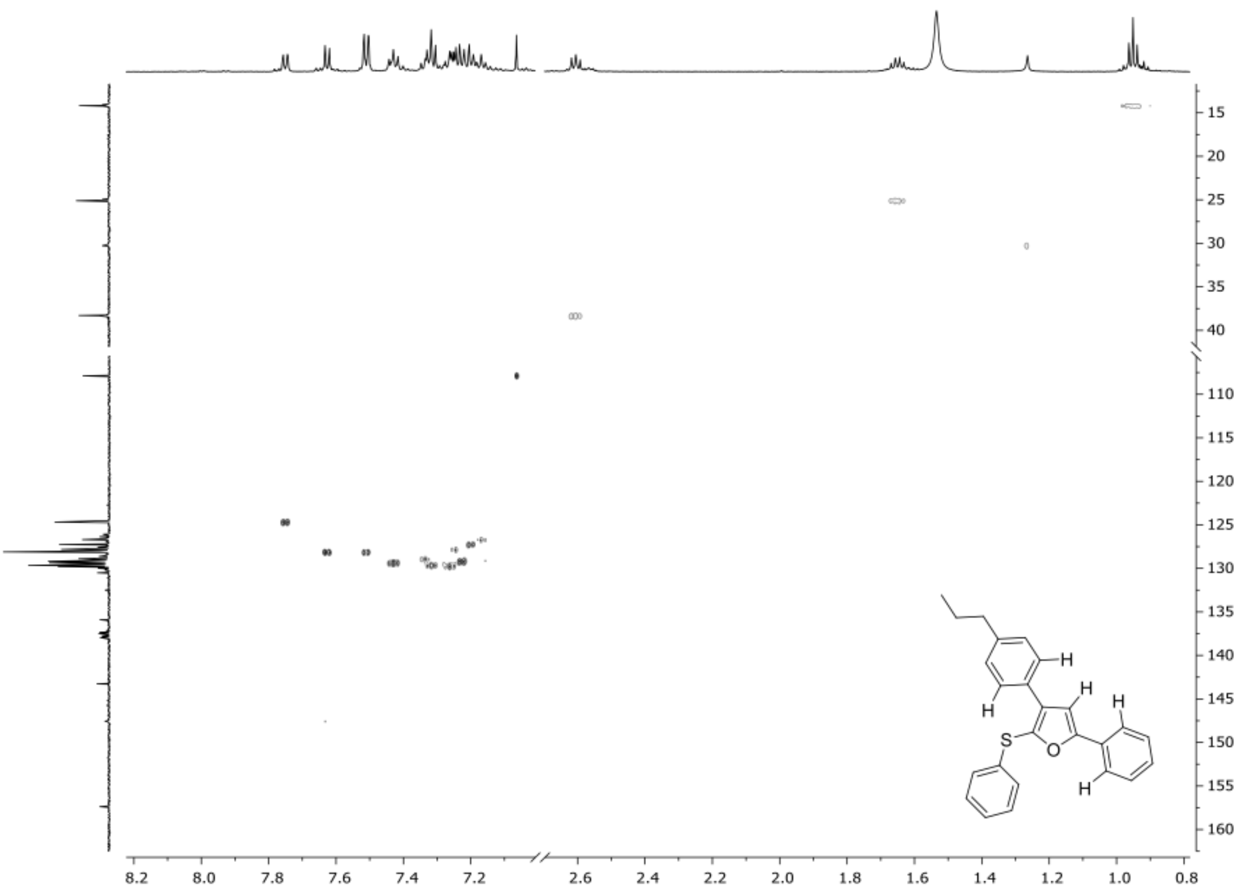

Figure S25. ^{1}H-^{13}C HSQC spectrum for the 5-phenyl-2-(phenylthio)-3-(4-propylphenyl)furan, CD_2Cl_2, 600 MHz. Region without significant signals is cut.

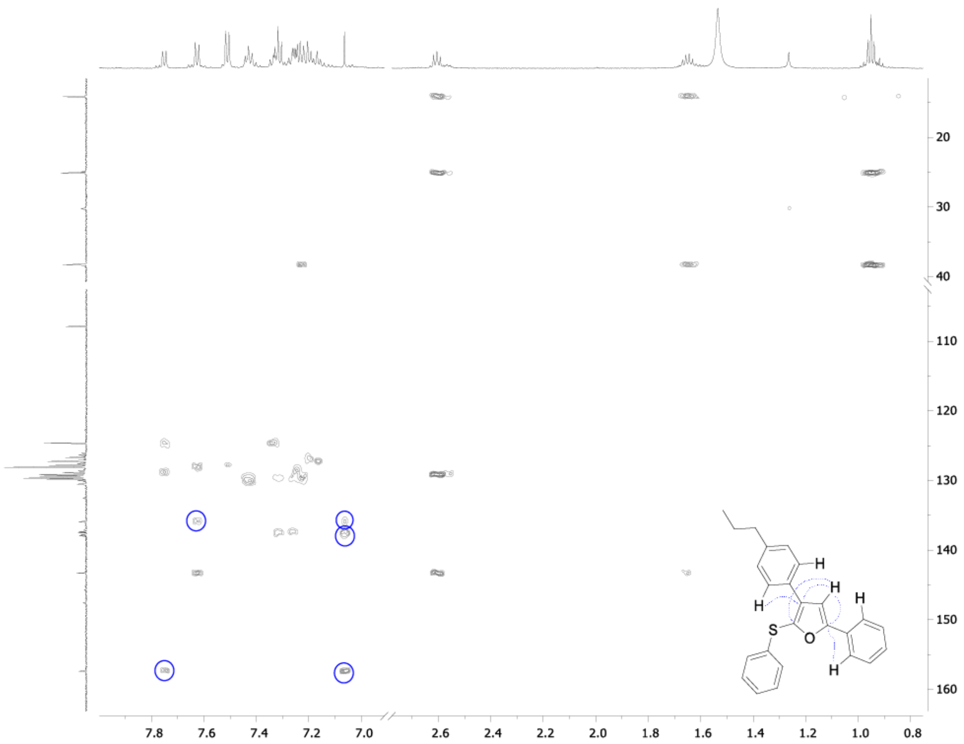

Figure S26. ^{1}H-^{13}C HMBC spectrum of the 5-phenyl-2-(phenylthio)-3-(4-propylphenyl)furan, CD_2Cl_2, 600 MHz. Key correlations are marked with blue. Region without significant signals is cut.

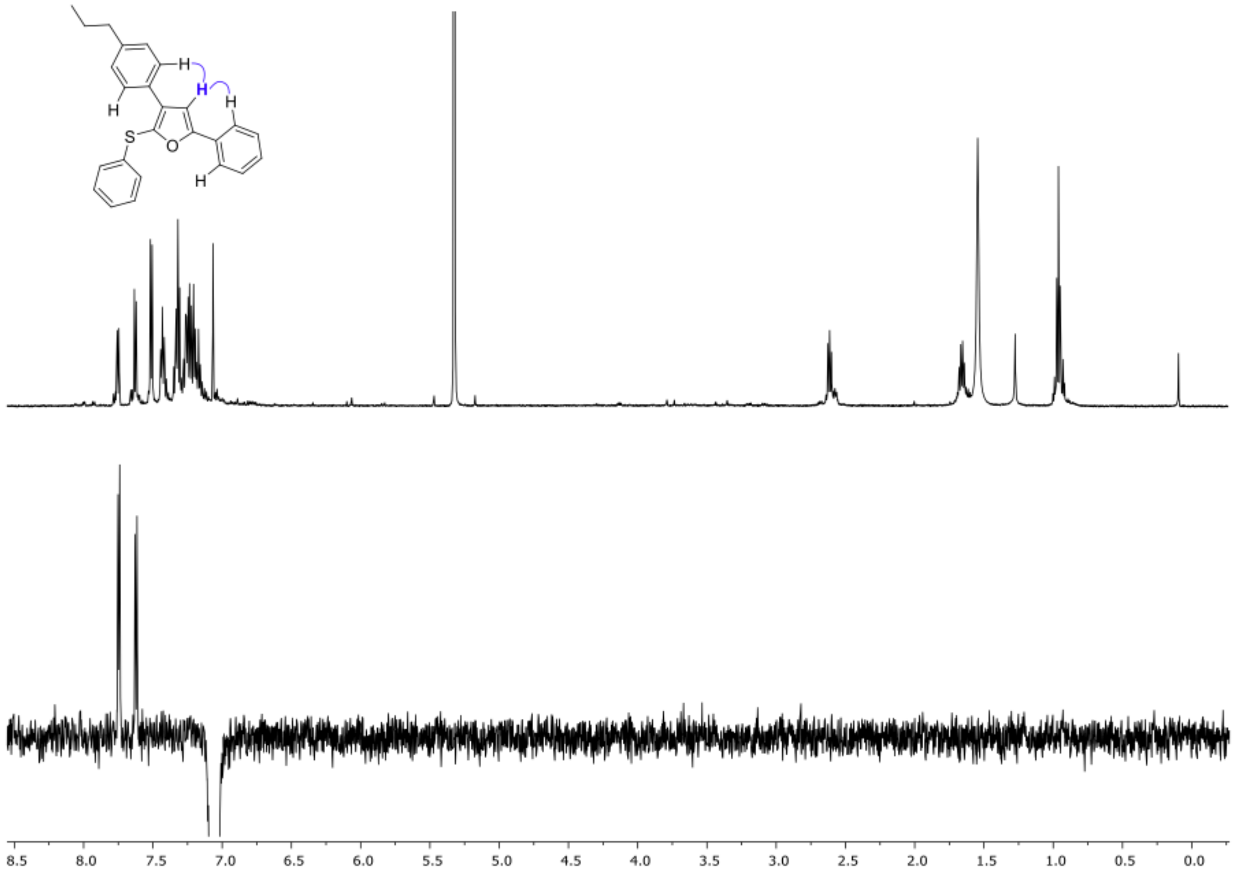

Figure S27. 1D selective NOE experiment (bottom) and usual ^{1}H NMR spectrum (top) for 5-phenyl-2-(phenylthio)-3-(4-propylphenyl)furan, CD_2Cl_2, 600 MHz. The detected interaction with the CH proton from the furan ring is shown.

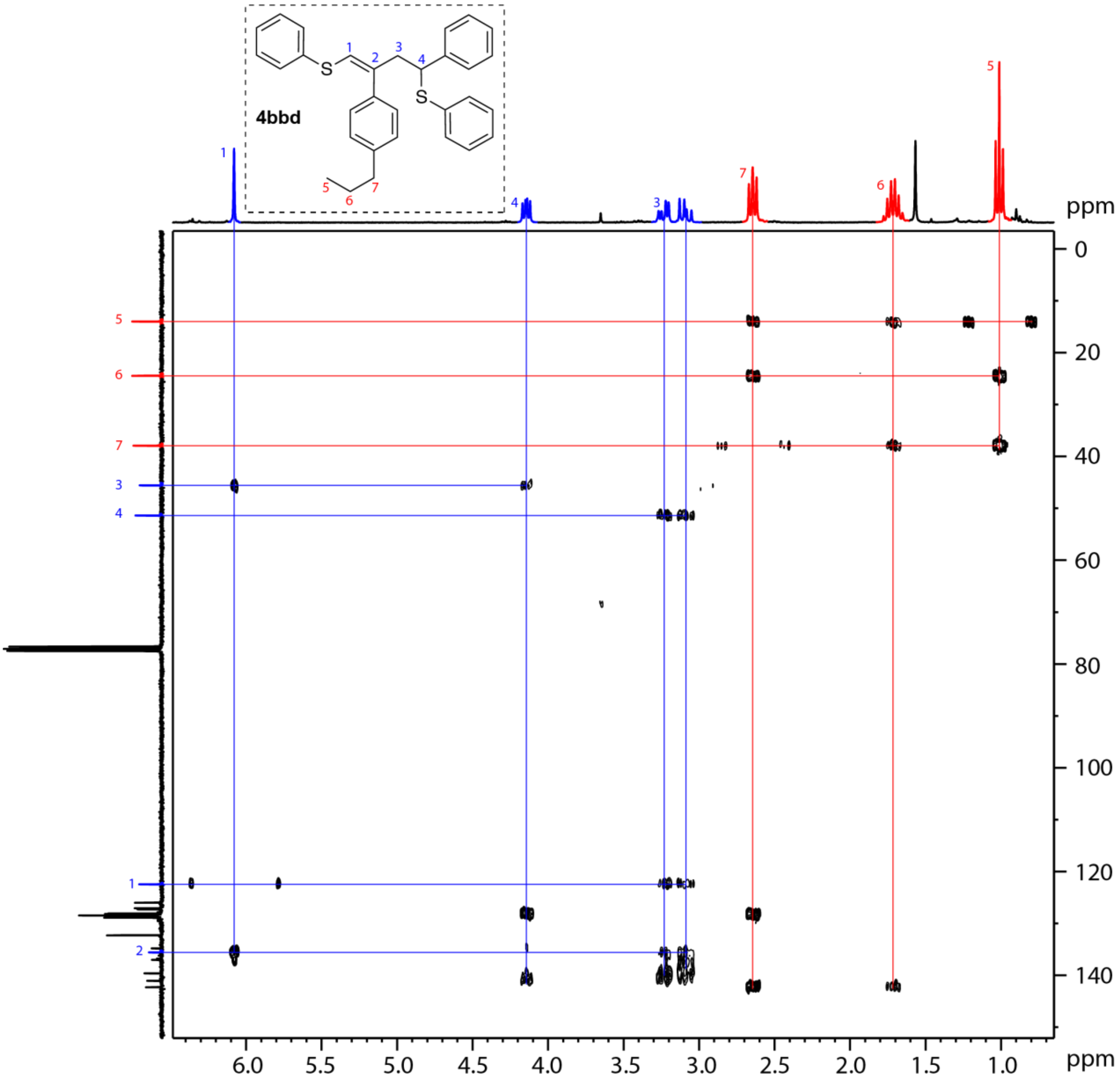

Figure S28. Fragment of ^{1}H-^{13}C HMBC spectrum of **4bbd**, $CDCl_3$, 300 MHz. Key correlations of the main carbon chain marked in blue. Correlations of *n*-Pr are marked in red.

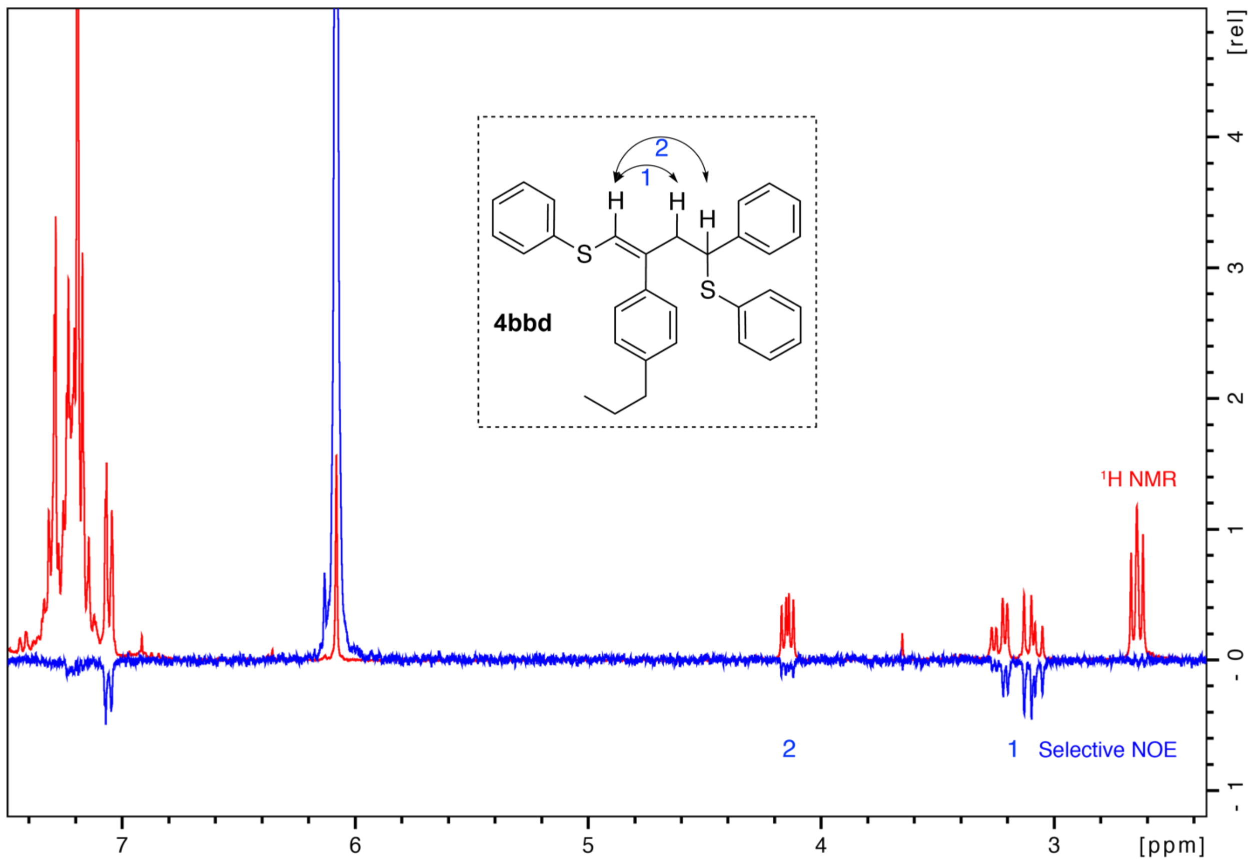

Figure S29. Fragments of the 1D selective NOE experiment (bottom) and usual ^{1}H NMR spectrum (top) of **4bbd**, $CDCl_3$, 300 MHz. The detected interactions of vinyl protons with aliphatic protons.

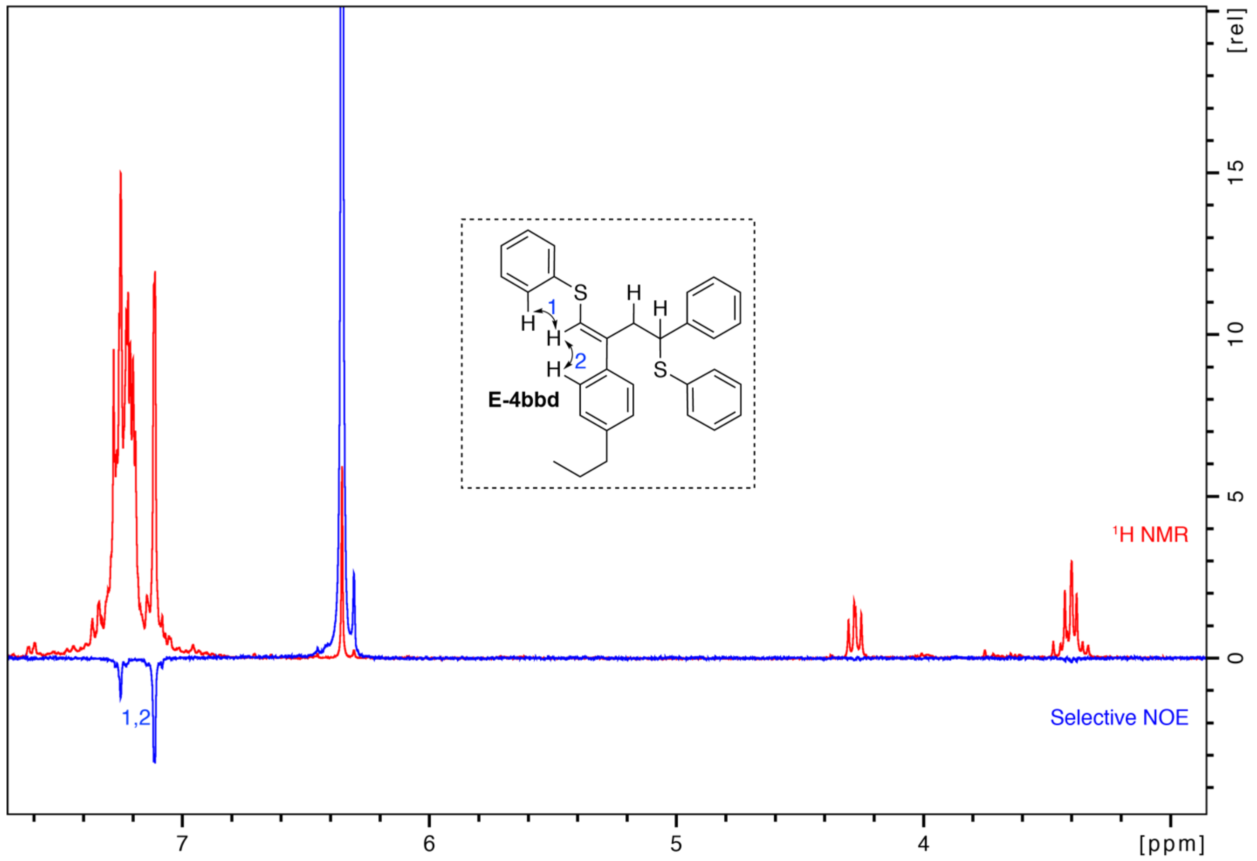

Figure S30. Fragments of the 1D selective NOE experiment (bottom) and usual ^{1}H NMR spectrum (top) of **E-4bbd**, $CDCl_3$, 300 MHz. No strong interaction of vinyl and aliphatic protons was detected.

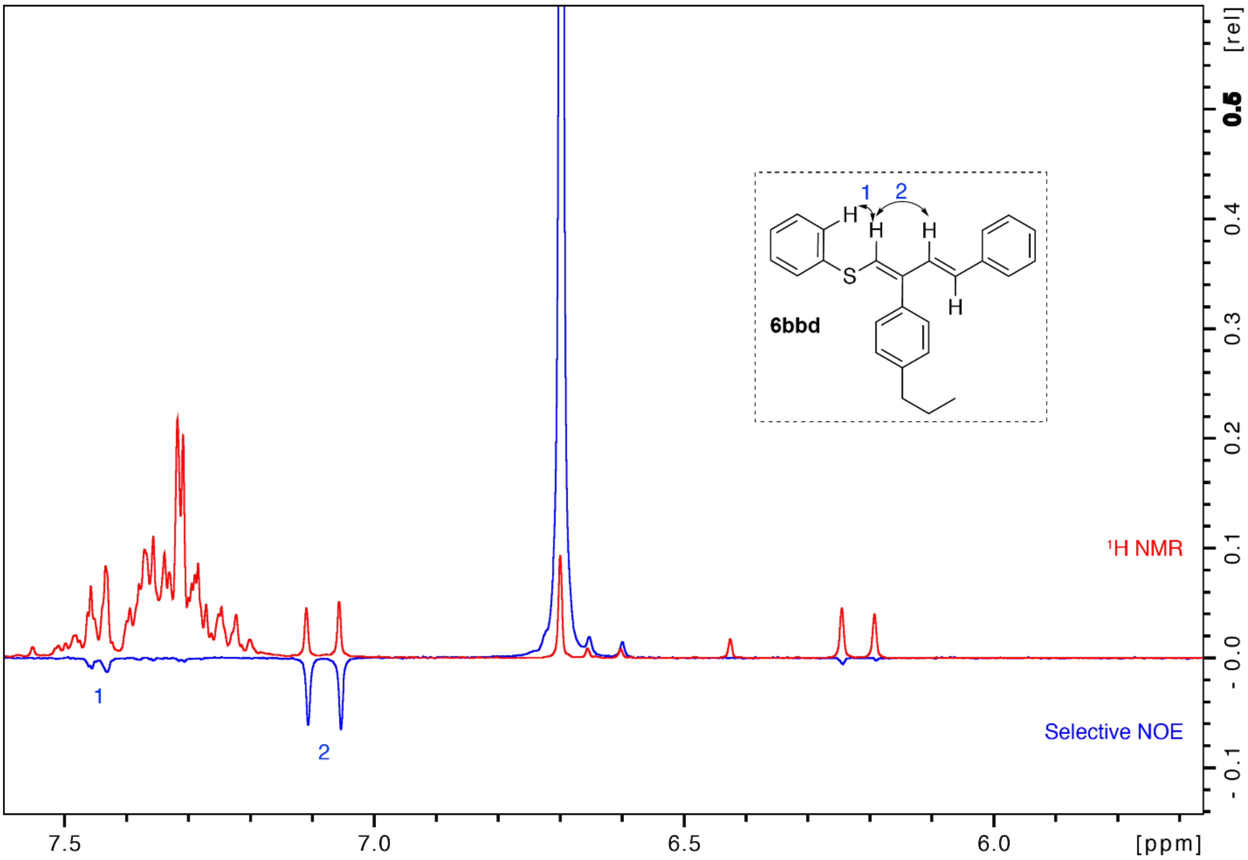

Figure S31. Fragments of 1D selective NOE experiment (bottom) and usual ^{1}H NMR spectrum (top) of **6bbd**, $CDCl_3$, 300 MHz.

References

[1] C. P. Rosenau, B. J. Jelier, A. D. Gossert, A. Togni, Angew. Chemie Int. Ed. 2018, 57, 9528–9533.
[2] L. Krause, R. Herbst-Irmer, G. M. Sheldrick, D. Stalke, J. Appl. Crystallogr. 2015, 48, 3–10.
[3] G. M. Sheldrick, Acta Crystallogr. Sect. A Found. Adv. 2015, 71, 3–8.
[4] M. J. Frisch, G. W. Trucks, H. B. Schlegel, G. E. Scuseria, M. A. Robb, J. R. Cheeseman, G. Scalmani, V. Barone, G. A. Petersson, H. Nakatsuji, X. Li, M. Caricato, A. V Marenich, J. Bloino, B. G. Janesko, R. Gomperts, B. Mennucci, H. P. Hratchian, J. V Ortiz, A. F. Izmaylov, J. L. Sonnenberg, D. Williams-Young, F. Ding, F. Lipparini, F. Egidi, J. Goings, B. Peng, A. Petrone, T. Henderson, D. Ranasinghe, V. G. Zakrzewski, J. Gao, N. Rega, G. Zheng, W. Liang, M. Hada, M. Ehara, K. Toyota, R. Fukuda, J. Hasegawa, M. Ishida, T. Nakajima, Y. Honda, O. Kitao, H. Nakai, T. Vreven, K. Throssell, J. A. Montgomery Jr., J. E. Peralta, F. Ogliaro, M. J. Bearpark, J. J. Heyd, E. N. Brothers, K. N. Kudin, V. N. Staroverov, T. A. Keith, R. Kobayashi, J. Normand, K. Raghavachari, A. P. Rendell, J. C. Burant, S. S. Iyengar, J. Tomasi, M. Cossi, J. M. Millam, M. Klene, C. Adamo, R. Cammi, J. W. Ochterski, R. L. Martin, K. Morokuma, O. Farkas, J. B. Foresman, D. J. Fox, Gaussian16 Revision C.01, 2016.
[5] J. P. Perdew, K. Burke, M. Ernzerhof, Phys. Rev. Lett. 1996, 77, 3865–3868.
[6] A. D. Becke, J. Chem. Phys. 1993, 98, 5648–5652.
[7] Y. Zhao, D. G. Truhlar, Theor. Chem. Acc. 2008, 120, 215–241.
[8] S. Grimme, J. Antony, S. Ehrlich, H. Krieg, J. Chem. Phys. 2010, 132, 154104.
[9] R. Krishnan, J. S. Binkley, R. Seeger, J. A. Pople, J. Chem. Phys. 1980, 72, 650–654.
[10] A. D. McLean, G. S. Chandler, J. Chem. Phys. 1980, 72, 5639–5648.
[11] S. Miertuš, E. Scrocco, J. Tomasi, Chem. Phys. 1981, 55, 117–129.
[12] K. Fukui, Acc. Chem. Res. 1981, 14, 363–368.
[13] Burykina, J. V.; Shlapakov, N. S.; Gordeev, E. G.; König, B.; Ananikov, V. P. *Chem. Sci.* **2020**, *11* (37), 10061–10070.
[14] D. W. Johnson and G. W. Rayner-Canham, *J. Chem. Educ.*, 1972, **49**, 211.
